# Overview of the Genetic Diversity of African *Macrotermes* (Termitidae: Macrotermitinae) and Implications for Taxonomy, Ecology and Food Science

**DOI:** 10.3390/insects12060518

**Published:** 2021-06-03

**Authors:** Bronwyn Egan, Zwannda Nethavhani, Barbara van Asch

**Affiliations:** 1 Department of Biodiversity, University of Limpopo, Private Bag X1106, Mankweng 0727, South Africa; bronwyn.egan@ul.ac.za; 2 Department of Genetics, University of Stellenbosch, Private Bag X1, Matieland 7602, South Africa; 20998880@sun.ac.za

**Keywords:** cryptic species, genetic divergence, mitochondrial markers, termites

## Abstract

**Simple Summary:**

*Macrotermes* are fungus-growing termites known as ecosystem engineers for their role in cellulose recycling and soil conditioning. *Macrotermes* termites are also important as edible insects and are widely consumed throughout Africa. Accurate identification of *Macrotermes* species is challenging because of few and unreliable morphological differences among taxonomic groups. Due to this limitation, the diversity of African *Macrotermes* is incompletely described. Genetic data has provided new insights, but vast geographic regions remain unsurveyed. We generated COI data for specimens collected in Limpopo, South Africa, where *Macrotermes* termites are commonly consumed by local populations, and assessed their diversity in the context of publicly available sequences. We identified 17 genetic groups that most likely represented distinct species, in contrast with the 13 *Macrotermes* species currently described in Africa. The specimens collected in Limpopo belonged to four genetic groups, suggesting a high diversity of *Macrotermes* in the region. Our results also showed that *Macrotermes* species names and genetic groups do not match in most cases, likely due to inaccurate identification of specimens. We propose that the genetic groups that are identified here be used as a background to guide future studies of African *Macrotermes* with a positive impact on food science, ecology, taxonomy, phylogeography and phylogenetics.

**Abstract:**

*Macrotermes* termites play important ecological roles and are consumed by many communities as a delicacy and dietary complement throughout Africa. However, lack of reliable morphological characters has hampered studies of *Macrotermes* diversity in a wide range of scientific fields including ecology, phylogenetics and food science. In order to place our preliminary assessment of the diversity of *Macrotermes* in South Africa in context, we analysed a comprehensive dataset of COI sequences for African species including new and publicly available data. Phylogenetic reconstruction and estimates of genetic divergence showed a high level of incongruity between species names and genetic groups, as well as several instances of cryptic diversity. We identified three main clades and 17 genetic groups in the dataset. We propose that this structure be used as a background for future surveys of *Macrotermes* diversity in Africa, thus mitigating the negative impact of the present taxonomic uncertainties in the genus. The new specimens collected in Limpopo fell into four distinct genetic groups, suggesting that the region harbours remarkable *Macrotermes* diversity relative to other African regions surveyed in previous studies. This work shows that African *Macrotermes* have been understudied across the continent, and that the genus contains cryptic diversity undetectable by classic taxonomy. Furthermore, these results may inform future taxonomic revisions in *Macrotermes*, thus contributing to advances in termitology.

## 1. Introduction

Termites (Blattodea) are the second most consumed insect group in the world, surpassed only by grasshoppers, crickets and locusts [1]. More than 1000 termite species have been recorded in Africa, all of which make vital environmental contributions by maintaining long-term soil yields and restoring unproductive soil [2]. Africa is also the hotspot of global termite consumption, with reported utilization of termites as food, medicine, livestock feed and for spiritual practices in 19 countries across the continent, in contrast with only five countries in both America and Asia, the closest competitors for edible termite diversity [1].

The family Termitidae is divided into five subfamilies, and the subfamily Macrotermitinae consists of approximately 330 species in 14 genera [3]. All Macrotermitinae have an obligate mutualism with *Termitomyces* fungi [4]. *Macrotermes* is the most preferred and well-known of the edible termite genera, likely due to their comparatively large size and high protein and lipid content [1]. The genus *Macrotermes* is presently divided into 47 species, of which 13 exist in the Afrotropical region [3]. However, this taxonomic structure may not reflect the real diversity of *Macrotermes* species because termites are notoriously difficult to identify due to their lack of reliable morphological characters [5]. Alates of *M. falciger* [6], *M. subhyalinus* [7], *M. michaelseni* [8], *M. bellicosus* [9], *M. natalensis* [10], *M. vitrialatus* [11] and *M. nigeriensis* [12] have been reported to be widely consumed in Africa [1,13,14]. Consumption of non-reproductive termite castes (i.e., workers and soldiers) is less extensively documented, and the most recorded species on the continent are *M. falciger*, *M. michaelseni* and *M. nigeriensis* [15].

Termites are increasingly threatened by changes in land use from communal pastureland to organized orchards and intensification of agricultural practices [16]. Due to their role as ecosystem engineers, which includes the ability to modify the physical and chemical distribution of soils and to influence their nutrient and moisture profiles, termite decline can have deleterious effects on the landscape and on organisms throughout the food cycle [17]. Termite colonies can persist in areas richly populated by people, albeit in lower densities [17], and are therefore an important component of functional ecosystems within an anthropogenically influenced arena. The role of non-reproductive termite castes in food security is noteworthy, as many other edible insects rely on particular host plant species or a narrow habitat range and are not as robust in their population size and persistence [18,19].

In South Africa, termites are the second most consumed insect, particularly in the KwaZulu-Natal and Limpopo provinces, where they are considered by local communities as a delicacy [20]. In the Venda region, alate termites are the most harvested, but the soldier and worker castes are also well appreciated and commonly found for sale [15]. Soldiers and workers can be harvested throughout the year, making them a more consistent food resource than alates [15]. Indeed, many villagers pour water over the dry mounds in winter, which results in easier capture of the soldiers and workers due to the mimicking of spring rains when termites are more active [21]. Due to their year-round availability, soldiers and workers are a promising source of insect protein. In Limpopo, soldier and worker termites are available in numbers, often within people’s yards in more rural areas, and even in towns they can be found on sidewalks.

Ethnospecies (i.e., vernacular names used by people in their locality) do not strictly converge with taxonomic classifications [22], although there can be strong links between vernacular and scientific names [13]. Generally, termites are named according to their utilitarian use or known by the damage they cause [22]. Thus, different castes of the same scientific taxon may be known by different vernacular names [22,23]. Although ethnospecies and scientific names do not overlap precisely, the knowledge of local termite experts is vitally important in the recognition of edible species, as well as their collection, preparation for consumption and processing for storage [22]. The species consumed depend on localized indigenous knowledge and personal taste and therefore not all edible termite species are utilized over the entire distribution range of each species [13].

Despite the ecological and nutritional relevance of termites, food science and other scientific fields have been negatively impacted by taxonomic uncertainties due to a lack of reliable morphological characters for species identification. This work provides the most comprehensive overview to date of African *Macrotermes* diversity using new and publicly available COI sequences. Furthermore, we performed a preliminary assessment of the genetic diversity of termites called “Majeje” or “Makeke”, a traditional food resource in Limpopo, South Africa.

## 2. Materials and Methods

### 2.1. Specimen Collection

**Figure 1:**
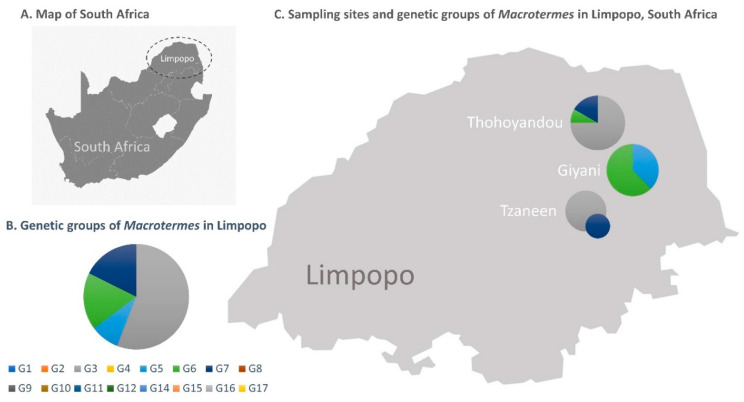
(**A**) Map of South Africa. (**B**) Genetic groups of African *Macrotermes* species. (**C**) Approximate location of the collection sites of *Macrotermes* termites in the Limpopo Province of South Africa, and their mitochondrial genetic groups. The size of the circles is proportional to the number of sequences.

Soldiers, workers and alate termites were haphazardly collected at four sites in the Limpopo Province of South Africa between February and December of 2020 (Table 1, Figure 1C). Alates (*n* = 15) were found in house yards, and soldiers and workers (*n* = 19) were collected from a single mound at each site. Specimens were euthanized by freezing within a few hours of collection from the field, and individually stored in 100% ethanol at room temperature until downstream analyses. DNA was extracted from termite legs using a standard phenol-chloroform method [24]. 

### 2.2. DNA Extraction, PCR Amplification and Sequencing 

A total of 34 specimens were sequenced for the standard COI barcoding region (702 bp). PCR amplifications were performed using a pair of species-specific primers designed in this study, based on the complete mitochondrial genome of *M. falciger* (NC_034050). The new PCR primers (Mfal-F 5′-TCTCAACTAATCATAAAGACATTG-3′ and Mfal-R 5′- TATACTTCGGGGTGTCCGAAG-3′) were manually designed to anneal to the same COI regions as the universal arthropod primers HLO/LCO [25] whilst avoiding the production of non-specific amplicons. PCR amplifications were performed in a total volume of 5 μL containing 1x of QIAGEN Multiplex PCR Kit (QIAGEN), 0.2 μM of each primer, 1.0 μL of MilliQ H2O and 0.5 μL of template DNA. The thermal cycling program was as follows: 15 min at 95 °C; 35 cycles of 30 s at 94 °C; 90 s at 56 °C; 90 s at 72 °C; and 10 min at 72 °C. PCR products were sequenced unidirectionally with the Mfal-F primer, using the BigDye Terminator v3.1 Cycle Sequencing Kit (Applied Biosystems, Waltham, MA, USA) as per the manufacturer’s protocol. Capillary electrophoresis was performed at the Central Analytical Facilities of Stellenbosch University, South Africa.

### 2.3. DNA Sequence Analyses

Genetic clustering and genetic divergences were calculated using the new sequences generated in this study and COI sequences taxonomically assigned to *Macrotermes* species available on GenBank as of 1 April 2021. The initial dataset (*n* = 278) was subsequently filtered for (a) sequences identified to the species level, (b) species recorded in Africa, and (c) sequences longer than 500 bp and with maximum overlap with the standard COI barcoding region. The final dataset included 229 sequences of African *Macrotermes* retrieved from GenBank representing 11 species, and 34 new sequences generated in this study. Multiple sequence alignments were performed using the MAFFT algorithm [26] in Geneious Prime v2021.1 (https://www.geneious.com) (accessed on 2 May 2021). Genetic clustering of sequences was assessed using a maximum likelihood (ML) tree, with *Microtermes obesi* (NC_034072) as the outgroup. The tree was run on the PHYML online server (http://www.atgc-montpellier.fr/phyml/) (accessed on 2 May 2021) [27], using the Smart Model Selection [28] and the Fast likelihood-based test (aLERT) [29]. The final tree was drawn using FigTree v1.4.4 (http://tree.bio.ed.ac.uk/) (accessed on 2 May 2021). The dataset was also used to build a Neighbour-joining (NJ) tree in MEGAX [30], under the Kimura 2-parameter (K2P) model [31].

Genetic divergences were estimated as percentage of maximum pairwise distances (maximum p-distance, %) in MEGA X, under the K2P model. Intraspecific maximum p-distances were calculated for species groups (i.e., sequences were grouped according to species names), and intragroup maximum p-distances were calculated according to genetic groups (i.e., sequences were grouped according to the clusters recovered on the ML tree, disregarding the names of the sequences). 

Polymorphic mitochondrial regions were identified on an alignment of 12 *Macrotermes* mitogenomes (*M. annandalei*, NC034078; *M. barneyi*, NC018599; *M. carbonarius*, NC034046; *M. falciger*, NC034050; *M. gilvus*, NC034110; *M. malaccensis*, NC034030; *M. muelleri*, NC034127; *M. natalensis*, NC025522; *Macrotermes* sp. A TB-2017, KY224531; *Macrotermes* sp. B TB-2017, KY224525; *M. subhyalinus*, NC018128; and *M. vitrialatus*, NC034054) using the DNA Polymorphism function of DNAsP6 with a 50-bp sliding window and 25-bp step size [32]. The alignment excluded tRNA and rRNA genes, and the AT-rich region.

## 3. Results and Discussion

### 3.1. Overview of the Genetic Diversity of African *Macrotermes*

Morphological identification of termites is notoriously challenging, and taxonomic revisions in Blattodea are necessary to solve the high prevalence of uncertainties [33]. In the face of the limitations of classic taxonomy in this group, DNA sequences may provide a useful tool to solve long-standing issues of species identification in *Macrotermes*. Several studies have reported incongruencies between genetic data and species names, and it is possible that incorrect identification of *Macrotermes* species has been affecting results and conclusions in a wide range of scientific fields. In an effort to contribute to solving this problem, we performed a naïve DNA-based approach for inferring genetic groups that potentially correspond to species, using new and publicly available COI sequences for African *Macrotermes*.

#### 3.1.1. Sequence Data for African *Macrotermes* Are Sparse and Unevenly Distributed

**Figure 2:**
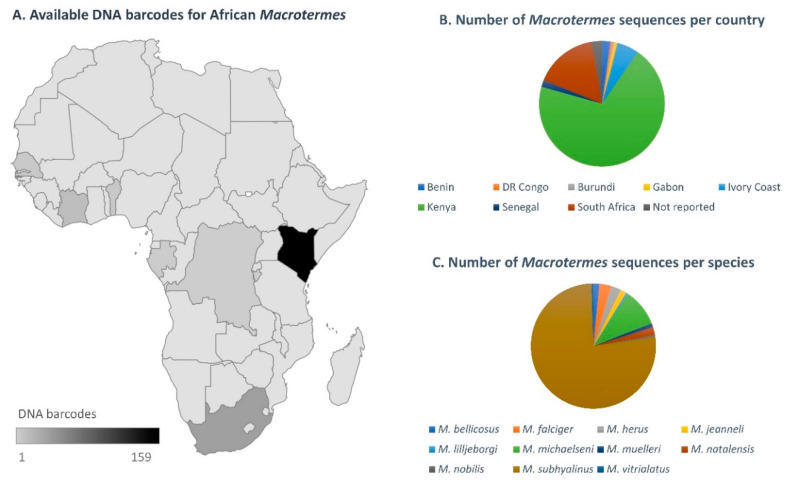
(**A**) Distribution of DNA barcodes for *Macrotermes* species in African countries, as determined using the complete dataset of sequences available on GenBank as of April 2021, and the new sequences generated in this study. (**B**) Number of DNA barcodes available per species, as of April 2021 (including the new sequences generated in this study). (**C**) Number of sequences available per species (not including new data generated in this study).

In spite of the wide distribution of *Macrotermes* in Africa, available DNA barcodes showed limited geographic coverage for the genus as sequences were only available for eight countries (Figure 2A). The coverage was also geographically biased: 72% of the sequences originated from Kenya, two countries (Burundi and Benin) were represented by a single sequence (Figure 2B) and South Africa was only represented by three sequences prior to this study. A total of eleven species were represented in the dataset, which comprised mostly sequences identified as *M. subhyalinus* (77%), followed by *M. michaelseni* (10%). Other species were represented by a minority of sequences, namely *M. falciger* and *M. herus* (3%); *M. bellicosus*, *M. jeanneli* and *M. natalensis* (2%); and *M. lilljeborgi*, *M. muelleri*, *M. nobilis* and *M. vitrialatus* (1%) (Figure 2C).

#### 3.1.2. Sequences of African *Macrotermes* Fall into Three Main Clades and Seventeen Genetic Groups

**Figure 3:**
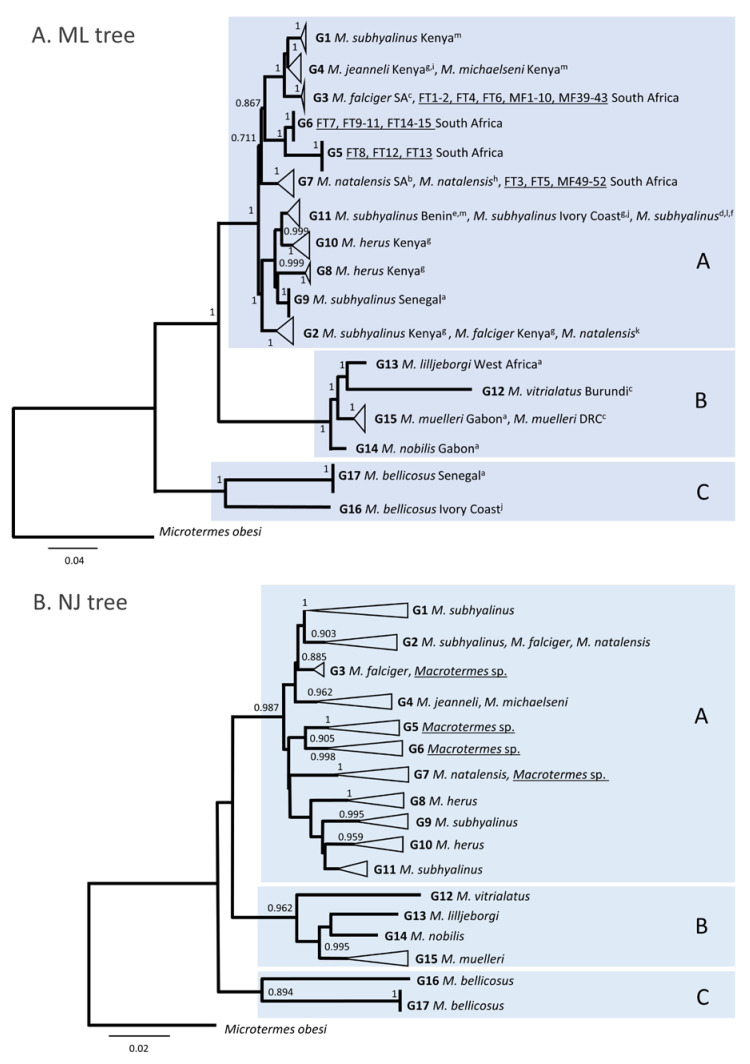
Phylogenetic trees of 228 COI sequences (536 bp) of African *Macrotermes*. The letters A, B and C on the ML and NJ trees represent the main clades in the phylogeny. G1 to G17 represent the different genetic groups in the dataset (intragroup maximum p-distances <3%). Sequences generated in this study are shown underlined. (**A**) Maximum likelihood tree. ^a^ Aanen et al., 2002, ^b^ Aanen et al., 2005, ^c^ Bourguignon et al., 2017, ^d^ Cameron et al., 2012, ^e^ Hausberger et al., 2011, ^f^ Legendre et al., 2008, ^g^ Marten et al., 2009, ^h^ Meng et al., 2014, ^i^ Nobre et al., 2010, ^j^ Nobre et al., 2011, ^k^ Pekar et al., 2020, ^l^ Svenson et al., 2009, ^m^ Vesala et al., 2017. (**B**) Neighbour-joining tree. The main clades and genetic groups are consistent with the topology recovered in the ML tree.

The ML tree recovered three main phylogenetic clades (A, B and C), which is in agreement with previous works [34,35,36,37], although a shorter 536-bp of the COI gene, representing the maximum sequence dataset overlap, was analysed in our study (Figure 3A). This broad division into three main clades is also in agreement with the broad topology obtained using a region of the COII gene [38]. The majority of the nodes in the ML tree had high statistical support except for the deep split between clade C and the other two clades. Poor recovery of deep phylogenies using short mitochondrial DNA regions is expected but this limitation does not affect the conclusions of our work, which aimed to identify genetic groups within the dataset and not to reconstruct the phylogeny of *Macrotermes*. Sequences were optimally grouped based on the topology of the tree, and on the level of divergence (maximum p-distance) within the groups (Table 2), i.e., groups of sequences that were closely related on the tree and for which the intragroup maximum p-distance was lower than 3% were considered as a genetic group. This approach allowed for the identification of 17 genetic groups (G1 to G17) across the three main clades after testing for all possible groups (Table S1).

##### Clade A: *M. subhyalinus*, *M. jeanneli*, *M. michaelseni*, *M. natalensis*, *M. herus*, and *M. falciger*

Clade A represented 96% of the total dataset and had the highest number of species (six), and the highest number of genetic groups (G1 to G11). Clade A also had a high level of incongruity between species groups and genetic groups involving four species (*M. falciger*, *M. herus*, *M. natalensis* and *M. subhyalinus*) and 84.5% of the sequences in the clade (Figure 3). The most striking case was that of *M. subhyalinus*, the species that represented 77% of the GenBank sequence dataset. *Macrotermes subhyalinus* had an intraspecific maximum p-distance of 6.59% (Table 2), and the sequences were distributed in four genetic groups (G1, G2, G9 and G11) in clade A. The sequences in G1 reportedly represent a tentative identification of specimens collected in Kenya [39]. *Macrotermes subhyalinus* also was part of three other genetic groups: G9 (intragroup maximum p-distance = 0.00%), which included *M. subhyalinus* sequences from Senegal [34]; G11 (intragroup maximum p-distance = 1.50%), which included one *M. subhyalinus* from the Ivory Coast [37,40] and Benin (Hausberger et al., 2011); in contrast, G2 (intragroup maximum p distance = 0.51%) included *M. subhyalinus* and *M. falciger* sampled from Kenya in the same study [40], and one *M. natalensis* of unreported geographic origin [41]. Therefore, it is likely that the sequences of *M. subhyalinus* represent four distinct species, in addition to the known differences between *M. subhyalinus* from East Africa and West Africa [38,40].

*Macrotermes jeanneli* and *M. michaelseni* formed a group of very similar sequences (G4; intragroup maximum p-distance = 1.76%) suggesting that G4 is composed of conspecific individuals. G4 was closely related to G1 (*M. subhyalinus* Kenya), which is similar to previous findings using a COII-based tree [38]. *Macrotermes michaelseni* is a tentative identification of specimens from Kenya based on the assumption that *M. michaelseni* build mounds with closed ventilation shafts, in contrast with the open ventilation shafts built by *M. subhyalinus* [39]. *Macrotermes jeanneli* was reported from Kenya in two different studies [36,40], suggesting consistent identification of specimens.

*Macrotermes falciger* had an intraspecific maximum p-distance of 4.44% suggesting non-conspecificity of the sequences. *Macrotermes falciger* from South Africa [42] clustered with 20 of the new sequences from South Africa in G3 (intragroup maximum p-distance = 0.33%). In G2 (intragroup maximum p-distance = 0.51%), *M. falciger* from Kenya [40] clustered with *M. subhyalinus* from Kenya [40] and one *M. natalensis* of unknown geographic origin [41].

*Macrotermes natalensis* had a maximum intraspecific p-distance of 3.72% and clustered in two genetic groups. As described above, G7 included *M. natalensis* from South Africa [34], *M. natalensis* from unreported geographic origin [43] and six of the new sequences from South Africa, and G2 included *M. natalensis* of unreported geographic origin [41] and *M. falciger* and *M. subhyalinus* from Kenya [40]. The existence of two species in *M. natalensis* has been noted previously in specimens collected in South Africa and Malawi [38].

*Macrotermes herus* was sampled in Kenya [40] and had an intraspecific maximum p-distance of 4.46%. The sequences clustered in two groups: G8 (intragroup maximum p-distance = 0.15%) and G10 (intragroup maximum p-distance = 1.71%), indicating that they represent two distinct species. G8 and G10 represent respectively *M. herus* from west of the Rift Valley in Kenya and *M. herus* within the Rift Valley that were hypothesized to represent distinct species in a previous study [40].

##### Clade B: *M. lilljeborgi*, *M. muelleri*, *M. nobilis* and *M. vitrialatus*

Clade B included four species (*n* = 5) sampled in West Africa and Burundi [34,42], three of which were represented by a single sequence (*M. lilljeborgi*, *M. muelleri* and *M. nobilis*). The sequences in G15 were reported as *M. muelleri* in two independent studies, and likely represent the same species (maximum intragroup p-distance = 1.08%).

##### Clade C: *M. bellicosus*

Clade C was highly diverged from clades A and B, as noted in previous works [34,35,36,37,38] and included only *M. bellicosus* (*n* = 3). The high intraspecific maximum p-distance (8.17%) of *M. bellicosus* indicated that a division of the sequences into two groups was appropriate; therefore, G17 (maximum intragroup p-distance = 0.11%) and G16 likely represent distinct species under the name *M. bellicosus*. High sequence divergence within specimens identified as *M. bellicosus* has been previously noted and hypothesized to reflect geographic variation or cryptic species diversity [44]. G16 was sampled in the Ivory Coast [37] and G17 in Senegal [34], and most likely belong to a genus other than *Macrotermes*. *Macrotermes bellicosus* was formerly assigned to *Bellicositermes* [45], and Brandl et al. (2017) have noted that the high genetic divergence between *M. bellicosus* and the other African *Macrotermes* was correlated with biological differences: minor workers were the main mound builders in *M. bellicosus*, whereas this task was performed by major workers in all other species surveyed in the Ethiopian region.

### 3.2. Inference of *Macrotermes* Species in the New Sequences from South Africa

**Figure 4:**
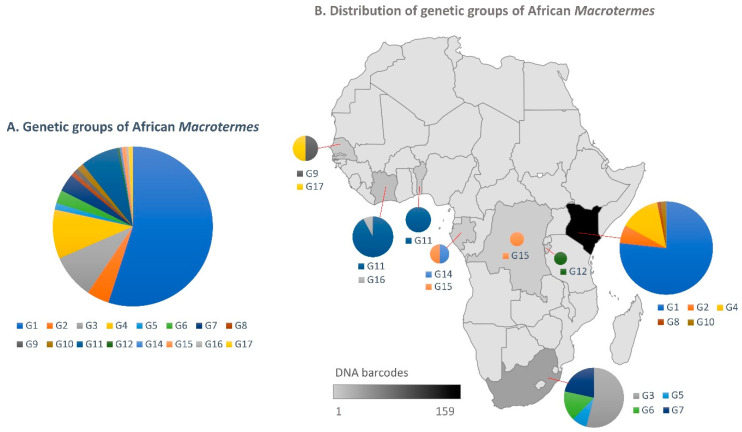
(**A**) Relative proportions of the genetic groups of African *Macrotermes* found in this study. (**B**) Distribution of the genetic groups in African countries. The size of the circles is proportional to the number of sequences for each genetic group.

In the light of the current taxonomic challenges, we did not attempt to seek expert assistance for morphological identification of the specimens analysed in this study. Hence, the following results are exclusively based on the genetic groups inferred from the total dataset. Our sequences indicated a high diversity of *Macrotermes* species in South Africa relative to surveys conducted in other countries (Figure 4). The haphazard sampling of 34 individuals at four sites recovered four distinct genetic groups (G3, G5, G6 and G7), two of which (G5 and G6) are reported here for the first time. The soldier and worker termites fell in two genetic groups (G3 and G7), and specimens from the same mound belonged to the same group, as expected. Alates collected at two sites (Giyani and Thohoyandou) belonged to four genetic groups (G3, G5, G6, G7). Remarkably, the three studies that contributed 72% of the sequences in the total dataset yielded only five genetic groups, with limited overlap with species names. A survey of *Macrotermes* diversity in the Tsavo Ecosystem in southern Kenya at eight different savanna and shrubland habitats recovered two sympatric species in a total of 141 individuals, which were tentatively identified as *M. subhyalinus* (G1) and *M. michaelseni* (G4) based on patterns of COI sequence similarity [39]. Another survey of *Macrotermes* colonies at 11 Kenyan sites reported four species that can be divided into four genetic groups with imperfect overlap with species names: *M. herus* (G8 and G10), *M. jeanneli* (G4, which includes *M. michaelseni* sequences) and *M. falciger* and *M. subhyalinus* (G2 in both cases) [40]. Interestingly, the same study also surveyed one site in the Ivory Coast where they also identified *M. subhyalinus*, but these sequences fall in a different genetic group (G11). Another example of taxonomic inconsistency is *M. bellicosus* sampled in Senegal (G16) [34] and *M. bellicosus* in the Ivory Coast (G17) [37], which likely belong to different species. 

**Figure 5:**
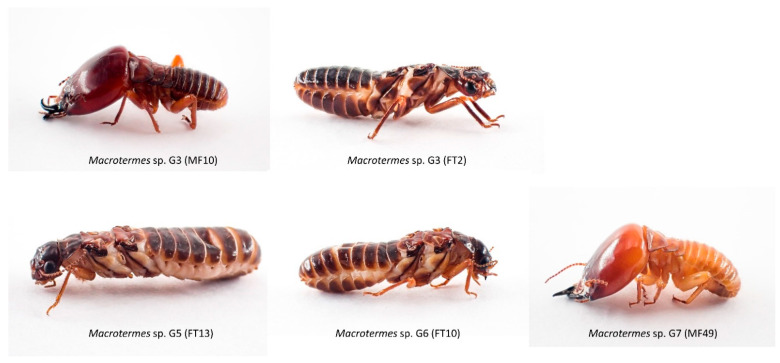
Representative specimens of *Macrotermes* termites collected in Limpopo, South Africa. G3, G5, G6 and G7 represent the genetic groups found in the region (specimen code in parenthesis).

Considering the overall results for the total dataset, i.e., the high level of incongruity between species names and genetic groups, the species identity of our specimens could not be inferred from the sequences (Figure 5). Our G7 sequences could represent *M. natalensis*, if the specimens at the origin of the sequences named *M. natalensis* (MK591923, AY818067 and AY818088) [35,41,43] were correctly identified by the authors of those studies. Similarly, our G3 sequences could represent specimens of *M. falciger* [42]. In contrast, G5 and G6 are here reported for the first time as they form unique genetic groups that may represent two closely related species (maximum p-distance G5 + G6 = 2.89%) (Table S1).

### 3.3. Mitochondrial Genetic Groups as A Background for Future Studies in African *Macrotermes*

The high level of incongruency between genetic clustering and species names, and between intraspecific and intragroup maximum p-distances indicates a high level of taxonomic inconsistencies and/or misidentifications in the *Macrotermes* sequences available on GenBank. This result is not surprising because the taxonomy and phylogeny of the genus *Macrotermes* have posed significant challenges and few characters can be used for species identification [45,46]. It is widely acknowledged that a paucity of informative morphological characters hampers termite studies, and termitologists working on West African species recently convened to start addressing the problem [33]. The results were staggering as only 10% of specimens were unambiguously identified to the species level, a proportion that increased to 25% by the end of the workshop, after long deliberations. Interestingly, *M. bellicosus* and *M. subhyalinus* were identified identically across experts, a result that was only achieved for five species out of 70. It is possible that the experts did not analyse specimens of the different known genetic lineages of each of those two species. However, the possibility remains that the morphological markers employed by the experts failed to detect differences that are evident at the DNA level [36,38,40]. As a result, *M. bellicosus* and *M. subhyalinus* were classified as unambiguous species and were not listed for taxonomic revision [33].

We propose that the structure of genetic diversity of *Macrotermes* presented here is a useful scaffold for framing sequence data generated in future studies. This baseline structure can be reassessed and expanded to accommodate additional genetic groups as the geographic coverage of *Macrotermes* diversity and the availability of sequence data increases. A possible first-line approach for analysing future COI datasets in African *Macrotermes* could consist of (1) retrieving the sequence dataset compiled in this study (Tables S2 and S3), (2) aligning this dataset with the new sequences, (3) building an NJ tree, (4) identifying the position of the new sequences on the NJ tree, which should fall within a known genetic group or appear as a new group, and (5) using maximum p-distances to reassess the genetic divergence within groups that include new sequences. NJ trees are not adequate for recovering deeper nodes but are much less computationally demanding than ML trees. In our analyses, the three main clades and the 17 genetic groups identified in the ML tree were correctly recovered in the NJ tree, albeit with low statistical support and a different order of the deeper nodes, as expected (Figure 3B). Thus, this methodological pipeline may be useful in future surveys of *Macrotermes*, as it will allow for a first overview of the total dataset that can inform subsequent analyses.

The use of mitochondrial DNA, particularly the COI gene for phylogenetic reconstruction and identification of new species has raised legitimate concerns [47]. However, phylogenetic reconstructions in termites using solely mitochondrial markers or combinations of mitochondrial and nuclear markers have been largely consistent, with no overlaps of intra- and intergroup sequence variability, and sequence clusters could be defined unambiguously [44]. Patterns of COI sequence clustering and divergence are useful to detect cryptic species diversity, but average p-distance values should be interpreted with caution. For example, a DNA barcode library was generated for *Trioza erytreae*, the psyllid vector for African citrus greening disease, under the assumption that all specimens analysed belonged to the same species [48]. Re-analyses of that sequence dataset using a fine-scale phylogenetic approach and maximum p-distances showed the presence of four genetically distinct groups likely representing different species that were not detected in the original study, which relied on broad-scale phylogenetic reconstruction and average p-distances [49].

### 3.4. Mitochondrial Markers for African *Macrotermes*

**Figure 6:**
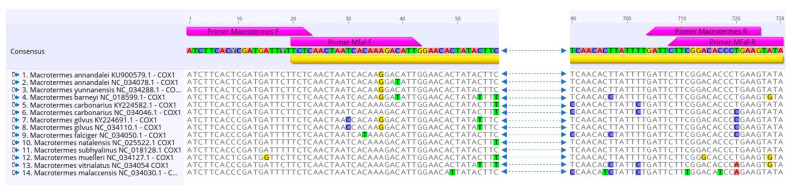
Alignment of COI sequences extracted from 14 complete mitochondrial genomes of *Macrotermes* available on GenBank. The primers Mfal-F and Mfal-R are specific for *M. falciger* and were designed for PCR amplification and sequencing of the standard DNA barcoding region (709 bp) in African *Macrotermes*. Macrotermes-F and Macrotermes-R represent theoretical universal primers for DNA barcoding (724 bp) of African and Asian *Macrotermes*.

Difficulties in obtaining consistent PCR amplification of COI in termites has been noted as a reason to favour COII for species identification [33]. However, the increasing availability of complete mitogenomes greatly facilitates the design of optimal primers for PCR amplification and Sanger sequencing. Utilizing taxon-specific primers can also contribute to reducing the generation of non-specific PCR products and nuclear mitochondrial sequences (NUMTs) [50]. In the beginning of this study, we designed primers specifically for *M. falciger* (Mfal-F and Mfal-R), which proved to be robust in the other genetic groups of African *Macrotermes* present in our samples (Figure 6). This primer pair seems to have potential for use in other termite groups, as we also used it to generate high quality sequences for *Hodotermes* sp. (data not shown). In hindsight, it would have been preferable to utilize generic primers with potential to amplify a wider range of *Macrotermes*, including the Asian species, such as the theoretical primer pair Macrotermes-F and Macrotermes-R shown in Figure 6.

**Figure 7:**
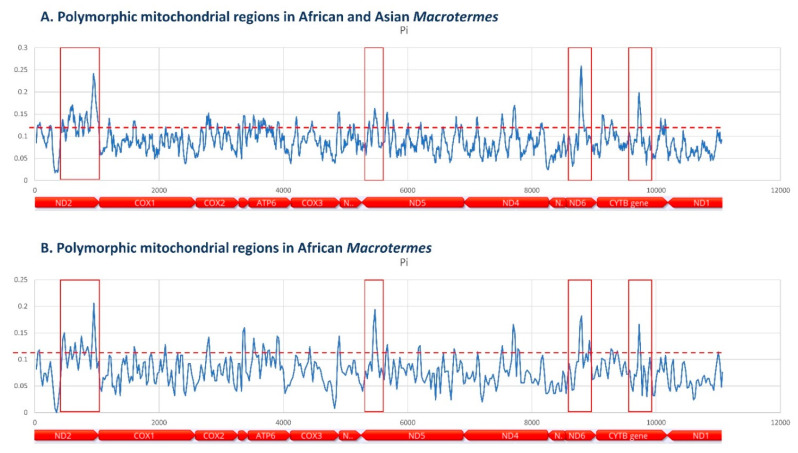
Polymorphic regions with potential utility as genetic markers for phylogenetic and species diversity studies in *Macrotermes* identified in the alignments of mitochondrial genomes of (**A**) African (*n* = 5) and (**B**) African and Asian species (*n* = 12).

Generating informative data in addition to the commonly used COI sequence may be necessary for higher resolution of genetic groups, and it has been suggested that COII could be an adequate candidate. This recommendation seems to stem from the perception that the PCR amplification of COII is more successful than that of COI, and that COII provides the best taxonomic resolution [33]. As explained above, optimization of primers that perform robustly across taxa is attainable because of the current availability of many mitochondrial genomes in practically all insect orders and families. This availability also allows for informed decisions on the selection of informative markers (i.e., mitochondrial regions with high levels of polymorphism) by comparing sequences among taxa of interest [51]. Informative mitochondrial regions may vary between taxa and taxonomic levels, i.e., they may fall on different genes for different taxonomic groups. Therefore, it is advisable to perform preliminary assessments of potential target regions in the taxa of interest. Protein-coding regions should be preferred over transfer and ribosomal RNA genes and the AT-rich region. This is justifiable because these regions contain repetitive stretches of nucleotides and are prone to generating sequencing artifacts that cannot be detected by translation during sequence quality control. The alignment of all available mitogenome for African and Asian *Macrotermes* showed that the most informative mitochondrial regions that can be used to complement standard COI barcoding sequences are located in the ND1, ND5, ND6 and CYTB genes (Figure 7).

## 4. Conclusions

The absence of reliable morphological characters has hampered termite studies for a long time, and it is now apparent that DNA sequence data can provide much needed clarifications [44]. The high level of taxonomic incongruity evidenced in our study is concerning and casts doubt on the reliability of the identification of *Macrotermes* species reported in a wide range of research fields including phylogenetics, ecology and food science. Our results confirm that DNA-based analyses should complement the current methods of species identification in *Macrotermes* as classic taxonomy and ecological information are insufficient for accurately cataloguing species diversity. The structure of genetic groups of African *Macrotermes* that we propose was based on the analysis of the most comprehensive dataset currently available. This depiction is likely incomplete and the identification of new genetic groups and further cryptic diversity in African *Macrotermes* may challenge our conclusions. Nonetheless, the low geographic coverage and small amount of data generated so far provide a good opportunity to establish a background for future studies.

## Figures and Tables

**Table 1:** Specimens of African *Macrotermes* collected in the Limpopo Province of South Africa for preliminary assessment of species diversity in the region based on COI sequences.

Specimen	Caste	Site	Collection Date	Municipality	Village	GPS Coordinates	
FT01	Alate	House yard	28-November-2020	Thohoyandou	Lufule 2	−22.96386	30.51733
FT02	Alate	House yard	28-November-2020	Thohoyandou	Lufule 2	−22.96386	30.51733
FT03	Alate	House yard	28-November-2020	Thohoyandou	Lufule 2	−22.96386	30.51733
FT04	Alate	House yard	28-November-2020	Thohoyandou	Lufule 2	−22.96386	30.51733
FT05	Alate	House yard	28-November-2020	Thohoyandou	Lufule 2	−22.96386	30.51733
FT06	Alate	House yard	28-November-2020	Thohoyandou	Lufule 2	−22.96386	30.51733
FT07	Alate	House yard	28-November-2020	Thohoyandou	Lufule 2	−22.96386	30.51733
FT08	Alate	House yard	10-December-2020	Giyani	Ka-Homu	−23.33400	30.77300
FT09	Alate	House yard	10-December-2020	Giyani	Ka-Homu	−23.33400	30.77300
FT10	Alate	House yard	10-December-2020	Giyani	Ka-Homu	−23.33400	30.77300
FT11	Alate	House yard	10-December-2020	Giyani	Ka-Homu	−23.33400	30.77300
FT12	Alate	House yard	10-December-2020	Giyani	Ka-Homu	−23.33400	30.77300
FT13	Alate	House yard	10-December-2020	Giyani	Ka-Homu	−23.33400	30.77300
FT14	Alate	House yard	10-December-2020	Giyani	Ka-Homu	−23.33400	30.77300
FT15	Alate	House yard	10-December-2020	Giyani	Ka-Homu	−23.33400	30.77300
MF01	Soldier/worker	Termite mound	19-August-2020	Tzaneen	Moleketla	−23.68000	30.28000
MF02	Soldier/worker	Termite mound	19-August-2020	Tzaneen	Moleketla	−23.68000	30.28000
MF03	Soldier/worker	Termite mound	19-August-2020	Tzaneen	Moleketla	−23.68000	30.28000
MF04	Soldier/worker	Termite mound	19-August-2020	Tzaneen	Moleketla	−23.68000	30.28000
MF05	Soldier/worker	Termite mound	19-August-2020	Tzaneen	Moleketla	−23.68000	30.28000
MF06	Soldier/worker	Termite mound	19-August-2020	Tzaneen	Moleketla	−23.68000	30.28000
MF07	Soldier/worker	Termite mound	19-August-2020	Tzaneen	Moleketla	−23.68000	30.28000
MF08	Soldier/worker	Termite mound	19-August-2020	Tzaneen	Moleketla	−23.68000	30.28000
MF09	Soldier/worker	Termite mound	19-August-2020	Tzaneen	Moleketla	−23.68000	30.28000
MF10	Soldier/worker	Termite mound	19-August-2020	Tzaneen	Moleketla	−23.68000	30.28000
MF39	Soldier/worker	Termite mound	30-November-2020	Thohoyandou	Lufule 2	−22.96386	30.51733
MF40	Soldier/worker	Termite mound	30-November-2020	Thohoyandou	Lufule 2	−22.96386	30.51733
MF41	Soldier/worker	Termite mound	30-November-2020	Thohoyandou	Lufule 2	−22.96386	30.51733
MF42	Soldier/worker	Termite mound	30-November-2020	Thohoyandou	Lufule 2	−22.96386	30.51733
MF43	Soldier/worker	Termite mound	30-November-2020	Thohoyandou	Lufule 2	−22.96386	30.51733
MF49	Soldier/worker	Termite mound	14-February-2020	Tzaneen	Ga-Mmamatsha	−24.012259	29.81153
MF50	Soldier/worker	Termite mound	14-February-2020	Tzaneen	Ga-Mmamatsha	−24.012259	29.81153
MF51	Soldier/worker	Termite mound	14-February-2020	Tzaneen	Ga-Mmamatsha	−24.012259	29.81153
MF52	Soldier	Termite mound	14-February-2020	Tzaneen	Ga-Mmamatsha	−24.012259	29.81153

**Table 2:** Pairwise distances among different genetic groups of African *Macrotermes*. Species—sequences were grouped according to species names. Genetic groups—sequences were grouped according to phylogenetic clusters.

Species	Maximum p-Distance (%)	Mean p-Distance (%)	SE
*Macrotermes bellicosus*	8.17	6.17	0.98
*Macrotermes falciger*	4.44	0.54	0.20
*Macrotermes herus*	4.46	2.04	0.47
*Macrotermes jeanneli*	0.00	0.00	0.00
*Macrotermes lilljeborgi*	n.a.	n.a.	n.a.
*Macrotermes michaelseni*	0.22	0.00	0.00
*Macrotermes muelleri*	1.08	1.41	0.54
*Macrotermes natalensis*	3.72	2.04	0.54
*Macrotermes nobilis*	n.a.	n.a.	n.a.
*Macrotermes subhyalinus*	6.59	0.95	0.22
*Macrotermes vitrialatus*	n.a.	n.a.	n.a.
**Genetic group**			
G1	0.55	0.03	0.01
G2	0.51	0.18	0.13
G3	0.33	0.04	0.03
G4	1.19	0.20	0.09
G5	0.00	0.00	0.00
G6	0.00	0.00	0.00
G7	2.15	0.67	0.25
G8	0.15	0.00	0.00
G9	0.00	0.00	0.00
G10	1.71	1.07	0.39
G11	1.50	0.40	0.12
G12	n.a.	n.a.	n.a.
G13	n.a.	n.a.	n.a.
G15	1.08	1.41	0.49
G14	n.a.	n.a.	n.a.
G16	n.a.	n.a.	n.a.
G17	0.11	0.00	0.00

## Data Availability

The sequences generated in this study were deposited on GenBank (MZ323635 to MZ323668).

## Supplementary Materials

The following are available online at https://www.mdpi.com/article/10.3390/insects12060518/s1, Table S1: Pairwise distances, Table S2: Sequence list, Table S3: Multiple sequence alignment of total dataset.
