# Supplementary material for: Overview of the Genetic Diversity of African Macrotermes (Termitidae: Macrotermitinae) and Implications for Taxonomy, Ecology and Food Science
Source: Insects. 2021 Jun 3;12(6):518. doi: 10.3390/insects12060518 (PMC8228397; doi:10.3390/insects12060518)
Supplement: Supplementary file 1 [file insects-12-00518-s001.zip › insects-1231601-supplementary.pdf]

Table S1. Maximum pairwise distances calculated for genetic groups of African *Macrotermes* identified in a COI-based phylogeny. G1 to G7 – genetic groups for which max p-distance < 3%. Other possible combinations of genetic groups closely related in the phylogenetic tree were also tested for max p-distances.

**Clade**	**Genetic group**	**max p-distance (%)**	**Species**	**Country**	**Reference**
A	G1	0.55	*M. subhyalinus*	Kenya	Vesala et al. 2017
A	G3	0.33	*M. falciger,* FT1-2, FT4, FT6, MF1-10, MF39-43	South Africa	Bourguignon et al. 2017; This study
A	G4	1.19	*M. jeanneli, M. michaelseni*	Kenya	Marten et al. 2009; Nobre et al. 2010; Vesala et al. 2017
A	G5	0.00	FT8, FT12-13	South Africa	This study
A	G6	0.00	FT7, FT9-11, FT14-15	South Africa	This study
A	G7	2.15	*M. natalensis,* FT3, FT5, MF49-52	South Africa	Aanen et al. 2005; Meng et al. 2014; This study
A	G1+G3+G4	3.01	*M. subhyalinus, M. falciger, M. jeanelli, M. natalensis,* FT1-2, FT4, FT6, MF1-10, MF39-43		Marten et al. 2009; Nobre et al. 2010; Vesala et al. 2017; Bourguignon et al. 2017; This study
A	G5+G6	2.89	FT7-15	South Africa	This study
A	G1 to G7	5.82	*M. subhyalinus, M. falciger, M. jeanelli, M. natalensis,* FT1-15, MF1-10, MF39-43, MF49-52	South Africa	Marten et al. 2009; Nobre et al. 2010; Vesala et al. 2017; Bourguignon et al. 2017; This study
A	G8	0.15	*M. herus*	Kenya	Marten et al. 2009
A	G9	0.00	*M. subhyalinus*	Senegal	Aanen et al. 2002
A	G10	1.71	*M. herus*	Kenya	Marten et al. 2009
A	G11	1.50	*M. subhyalinus*	Benin, Ivory Coast	Legendre et al. 2008; Marten et al. 2009; Svenson et al. 2009; Nobre et al. 2011; Hausberger et al. 2011; Cameron et al. 2012
A	G2	0.51	*M. subhyalinus, M. falciger, M. natalensis*	Kenya	Marten et al. 2009; Pekar et al. 2020

A	G10+G11	3.44	*M. herus, M. subhyalinus*		Legendre et al. 2008; Marten et al. 2009; Svenson et al. 2009; Nobre et al. 2011; Hausberger et al. 2011; Cameron et al. 2012
A	G8+G9	2.89	*M. herus, M. subhyalinus*		Marten et al. 2009; Aanen et al. 2002
A	G8 to G11	4.24	*M. herus, M. subhyalinus*		Aanen et al. 2002; Legendre et al. 2008; Marten et al. 2009; Svenson et al. 2009; Nobre et al. 2011; Hausberger et al. 2011; Cameron et al. 2012
A	G2 to G11	4.86	*M. subhyalinus, M. herus , M. falciger, M. natalensis*		Aanen et al. 2002; Legendre et al. 2008; Marten et al. 2009; Svenson et al. 2009; Nobre et al. 2011; Hausberger et al. 2011; Cameron et al. 2012; Pekar et al. 2020
A	G1 to G11	6.55	*M. subhyalinus, M. herus, M. natalensis, M. falciger, M. jeanneli, M. michaelseni*	Kenya, South Africa, Senegal, Benin, Ivory Coast	Aanen et al. 2002; Aanen 2005; Legendre et al. 2008; Marten et al. 2009; Svenson et al. 2009; Nobre et al. 2010; Nobre et al. 2011; Hausberger et al. 2011; Cameron et al. 2012; Meng et al. 2014; Bourguignon et al. 2017; Vesala et al. 2017; Pekar et al. 2020; This study
B	G12	n.a.	*M. vitrialatus*	Burundi	Bourguignon et al. 2017
B	G13	n.a.	*M. lilljeborgi*	Not reported	Aanen et al. 2002
B	G14	n.a.	*M. nobilis*	Gabon	Aanen et al. 2002
B	G15	1.08	*M. muelleri*	Gabon, DRC	Aanen et al. 2002; Bourguignon et al. 2017
B	G12 to G15	9.52	*M. vitrialatus, M. lilljeborgi, M. nobilis, M. muelleri*	Burundi, Gabon, DRC	Aanen et al. 2002; Bourguignon et al. 2017
C	G16	n.a.	*M. bellicosus*	Ivory Coast	Nobre et al. 2011
C	G17	0.11	*M. bellicosus*	Senegal	Aanen et al. 2002
C	G16+G17	8.17	*M. bellicosus*	Ivory Coast, Senegal	Aanen et al. 2002; Nobre et al. 2011

Table S2. List of COI sequences of African *Macrotermes* species used in this study, including previously published and new data. Genetic groups were determined by clustering analysis and pairwise distances.

Sequence	GenBank	Reference	Country	Clade	Genetic group
Macrotermes bellicosus 1	AY127702	Aanen et al. 2002	Senegal	C	G17
Macrotermes bellicosus 2	AY127711	Aanen et al. 2002	Senegal	C	G17
Macrotermes bellicosus 3	JF302840	Nobre et al. 2011	Ivory Coast	C	G16
Macrotermes falciger 1	FJ207434	Marten et al. 2009	Kenya	A	G02
Macrotermes falciger 2	FJ207437	Marten et al. 2009	Kenya	A	G02
Macrotermes falciger 3	FJ207435	Marten et al. 2009	Kenya	A	G02
Macrotermes falciger 4	FJ207436	Marten et al. 2009	Kenya	A	G02
Macrotermes falciger 5	FJ207438	Marten et al. 2009	Kenya	A	G02
Macrotermes falciger mitogenome	NC_034050	Bourguignon et al. 2017	South Africa	A	G03
Macrotermes herus 1	FJ207439	Marten et al. 2009	Kenya	A	G10
Macrotermes herus 2	FJ207441	Marten et al. 2009	Kenya	A	G10
Macrotermes herus 3	FJ207440	Marten et al. 2009	Kenya	A	G10
Macrotermes herus 4	FJ207442	Marten et al. 2009	Kenya	A	G08
Macrotermes herus 5	FJ207443	Marten et al. 2009	Kenya	A	G08
Macrotermes jeanneli 1	FJ207449	Marten et al. 2009	Kenya	A	G04
Macrotermes jeanneli 2	FJ207450	Marten et al. 2009	Kenya	A	G04
Macrotermes jeanneli 3	GQ922749	Nobre et al. 2010	Kenya	A	G04
Macrotermes lilljeborgi	AY127734	Aanen et al. 2002	Not reported	B	G13
Macrotermes michaelseni 1	KY197607	Vesala et al. 2017	Kenya	A	G04
Macrotermes michaelseni 2	KY197609	Vesala et al. 2017	Kenya	A	G04
Macrotermes michaelseni 3	KY197610	Vesala et al. 2017	Kenya	A	G04
Macrotermes michaelseni 4	KY197611	Vesala et al. 2017	Kenya	A	G04
Macrotermes michaelseni 5	KY197612	Vesala et al. 2017	Kenya	A	G04

Macrotermes michaelseni 6	KY197613	Vesala et al. 2017	Kenya	A	G04
Macrotermes michaelseni 7	KY197615	Vesala et al. 2017	Kenya	A	G04
Macrotermes michaelseni 8	KY197616	Vesala et al. 2017	Kenya	A	G04
Macrotermes michaelseni 9	KY197618	Vesala et al. 2017	Kenya	A	G04
Macrotermes michaelseni 10	KY197620	Vesala et al. 2017	Kenya	A	G04
Macrotermes michaelseni 11	KY197622	Vesala et al. 2017	Kenya	A	G04
Macrotermes michaelseni 12	KY197623	Vesala et al. 2017	Kenya	A	G04
Macrotermes michaelseni 13	KY197625	Vesala et al. 2017	Kenya	A	G04
Macrotermes michaelseni 14	KY197608	Vesala et al. 2017	Kenya	A	G04
Macrotermes michaelseni 15	KY197617	Vesala et al. 2017	Kenya	A	G04
Macrotermes michaelseni 16	KY197619	Vesala et al. 2017	Kenya	A	G04
Macrotermes michaelseni 17	KY197621	Vesala et al. 2017	Kenya	A	G04
Macrotermes michaelseni 18	KY197624	Vesala et al. 2017	Kenya	A	G04
Macrotermes michaelseni 19	KY197614	Vesala et al. 2017	Kenya	A	G04
Macrotermes muelleri	AY127703	Aanen et al. 2002	Gabon	B	G15
Macrotermes muelleri mitogenome	NC_034127	Bourguignon et al. 2017	DR Congo	B	G15
Macrotermes natalensis 1	MK591923	Pekar et al. 2020	Not reported	A	G02
Macrotermes natalensis 2	AY818067	Aanen et al. 2005	South Africa	A	G07
Macrotermes natalensis 3	AY818088	Aanen et al. 2005	South Africa	A	G07
Macrotermes natalensis mitogenome	NC_025522	Meng et al. 2014	Not reported	A	G07
Macrotermes nobilis	AY127705	Aanen et al. 2002	Gabon	B	G14
Macrotermes subhyalinus 1	FJ207447	Marten et al. 2009	Kenya	A	G02
Macrotermes subhyalinus 2	FJ207444	Marten et al. 2009	Kenya	A	G02
Macrotermes subhyalinus 3	FJ207446	Marten et al. 2009	Kenya	A	G02
Macrotermes subhyalinus 4	FJ207448	Marten et al. 2009	Kenya	A	G02
Macrotermes subhyalinus 5	FJ207445	Marten et al. 2009	Kenya	A	G02
Macrotermes subhyalinus 6	JF923288	Hausberger et al. 2011	Benin	A	G11
Macrotermes subhyalinus 7	JF923290	Hausberger et al. 2011	Benin	A	G11
Macrotermes subhyalinus 8	JF923292	Hausberger et al. 2011	Benin	A	G11
Macrotermes subhyalinus 9	JF923291	Hausberger et al. 2011	Benin	A	G11

Macrotermes subhyalinus 10	AY127708	Aanen et al. 2002	Senegal	A	G09
Macrotermes subhyalinus 11	AY127709	Aanen et al. 2002	Senegal	A	G09
Macrotermes subhyalinus 12	EU253856	Legendre et al. 2008	Not reported	A	G11
Macrotermes subhyalinus 13	FJ207424	Marten et al. 2009	Ivory Coast	A	G11
Macrotermes subhyalinus 14	FJ207425	Marten et al. 2009	Ivory Coast	A	G11
Macrotermes subhyalinus 15	FJ207426	Marten et al. 2009	Ivory Coast	A	G11
Macrotermes subhyalinus 16	FJ207428	Marten et al. 2009	Ivory Coast	A	G11
Macrotermes subhyalinus 17	FJ207429	Marten et al. 2009	Ivory Coast	A	G11
Macrotermes subhyalinus 18	FJ207430	Marten et al. 2009	Ivory Coast	A	G11
Macrotermes subhyalinus 19	FJ207432	Marten et al. 2009	Ivory Coast	A	G11
Macrotermes subhyalinus 20	FJ207431	Marten et al. 2009	Ivory Coast	A	G11
Macrotermes subhyalinus 21	FJ207427	Marten et al. 2009	Ivory Coast	A	G11
Macrotermes subhyalinus 22	FJ207433	Marten et al. 2009	Ivory Coast	A	G11
Macrotermes subhyalinus 23	JF923342	Hausberger et al. 2011	Benin	A	G11
Macrotermes subhyalinus 24	JF302838	Nobre et al. 2011	Ivory Coast	A	G11
Macrotermes subhyalinus 25	JF302839	Nobre et al. 2011	Ivory Coast	A	G11
Macrotermes subhyalinus 26	FJ802752	Svenson et al. 2009	Not reported	A	G11
Macrotermes subhyalinus 27	KY197592	Vesala et al. 2017	Kenya	A	G01
Macrotermes subhyalinus 28	KY197593	Vesala et al. 2017	Kenya	A	G01
Macrotermes subhyalinus 29	KY197596	Vesala et al. 2017	Kenya	A	G01
Macrotermes subhyalinus 30	KY197597	Vesala et al. 2017	Kenya	A	G01
Macrotermes subhyalinus 31	KY197599	Vesala et al. 2017	Kenya	A	G01
Macrotermes subhyalinus 32	KY197600	Vesala et al. 2017	Kenya	A	G01
Macrotermes subhyalinus 33	KY197601	Vesala et al. 2017	Kenya	A	G01
Macrotermes subhyalinus 34	KY197602	Vesala et al. 2017	Kenya	A	G01
Macrotermes subhyalinus 35	KY197603	Vesala et al. 2017	Kenya	A	G01
Macrotermes subhyalinus 36	KY197604	Vesala et al. 2017	Kenya	A	G01
Macrotermes subhyalinus 37	KY197605	Vesala et al. 2017	Kenya	A	G01
Macrotermes subhyalinus 38	KY197606	Vesala et al. 2017	Kenya	A	G01
Macrotermes subhyalinus 39	KY197485	Vesala et al. 2017	Kenya	A	G01

Macrotermes subhyalinus 40	KY197487	Vesala et al. 2017	Kenya	A	G01
Macrotermes subhyalinus 41	KY197490	Vesala et al. 2017	Kenya	A	G01
Macrotermes subhyalinus 42	KY197491	Vesala et al. 2017	Kenya	A	G01
Macrotermes subhyalinus 43	KY197495	Vesala et al. 2017	Kenya	A	G01
Macrotermes subhyalinus 44	KY197500	Vesala et al. 2017	Kenya	A	G01
Macrotermes subhyalinus 45	KY197501	Vesala et al. 2017	Kenya	A	G01
Macrotermes subhyalinus 46	KY197502	Vesala et al. 2017	Kenya	A	G01
Macrotermes subhyalinus 47	KY197503	Vesala et al. 2017	Kenya	A	G01
Macrotermes subhyalinus 48	KY197504	Vesala et al. 2017	Kenya	A	G01
Macrotermes subhyalinus 49	KY197505	Vesala et al. 2017	Kenya	A	G01
Macrotermes subhyalinus 50	KY197506	Vesala et al. 2017	Kenya	A	G01
Macrotermes subhyalinus 51	KY197507	Vesala et al. 2017	Kenya	A	G01
Macrotermes subhyalinus 52	KY197514	Vesala et al. 2017	Kenya	A	G01
Macrotermes subhyalinus 53	KY197516	Vesala et al. 2017	Kenya	A	G01
Macrotermes subhyalinus 54	KY197518	Vesala et al. 2017	Kenya	A	G01
Macrotermes subhyalinus 55	KY197520	Vesala et al. 2017	Kenya	A	G01
Macrotermes subhyalinus 56	KY197521	Vesala et al. 2017	Kenya	A	G01
Macrotermes subhyalinus 57	KY197524	Vesala et al. 2017	Kenya	A	G01
Macrotermes subhyalinus 58	KY197526	Vesala et al. 2017	Kenya	A	G01
Macrotermes subhyalinus 59	KY197528	Vesala et al. 2017	Kenya	A	G01
Macrotermes subhyalinus 60	KY197529	Vesala et al. 2017	Kenya	A	G01
Macrotermes subhyalinus 61	KY197530	Vesala et al. 2017	Kenya	A	G01
Macrotermes subhyalinus 62	KY197531	Vesala et al. 2017	Kenya	A	G01
Macrotermes subhyalinus 63	KY197532	Vesala et al. 2017	Kenya	A	G01
Macrotermes subhyalinus 64	KY197533	Vesala et al. 2017	Kenya	A	G01
Macrotermes subhyalinus 65	KY197534	Vesala et al. 2017	Kenya	A	G01
Macrotermes subhyalinus 66	KY197535	Vesala et al. 2017	Kenya	A	G01
Macrotermes subhyalinus 67	KY197536	Vesala et al. 2017	Kenya	A	G01
Macrotermes subhyalinus 68	KY197537	Vesala et al. 2017	Kenya	A	G01
Macrotermes subhyalinus 69	KY197538	Vesala et al. 2017	Kenya	A	G01

Macrotermes subhyalinus 70	KY197540	Vesala et al. 2017	Kenya	A	G01
Macrotermes subhyalinus 71	KY197541	Vesala et al. 2017	Kenya	A	G01
Macrotermes subhyalinus 72	KY197542	Vesala et al. 2017	Kenya	A	G01
Macrotermes subhyalinus 73	KY197543	Vesala et al. 2017	Kenya	A	G01
Macrotermes subhyalinus 74	KY197544	Vesala et al. 2017	Kenya	A	G01
Macrotermes subhyalinus 75	KY197548	Vesala et al. 2017	Kenya	A	G01
Macrotermes subhyalinus 76	KY197550	Vesala et al. 2017	Kenya	A	G01
Macrotermes subhyalinus 77	KY197551	Vesala et al. 2017	Kenya	A	G01
Macrotermes subhyalinus 78	KY197552	Vesala et al. 2017	Kenya	A	G01
Macrotermes subhyalinus 79	KY197553	Vesala et al. 2017	Kenya	A	G01
Macrotermes subhyalinus 80	KY197554	Vesala et al. 2017	Kenya	A	G01
Macrotermes subhyalinus 81	KY197556	Vesala et al. 2017	Kenya	A	G01
Macrotermes subhyalinus 82	KY197557	Vesala et al. 2017	Kenya	A	G01
Macrotermes subhyalinus 83	KY197558	Vesala et al. 2017	Kenya	A	G01
Macrotermes subhyalinus 84	KY197559	Vesala et al. 2017	Kenya	A	G01
Macrotermes subhyalinus 85	KY197560	Vesala et al. 2017	Kenya	A	G01
Macrotermes subhyalinus 86	KY197561	Vesala et al. 2017	Kenya	A	G01
Macrotermes subhyalinus 87	KY197562	Vesala et al. 2017	Kenya	A	G01
Macrotermes subhyalinus 88	KY197563	Vesala et al. 2017	Kenya	A	G01
Macrotermes subhyalinus 89	KY197565	Vesala et al. 2017	Kenya	A	G01
Macrotermes subhyalinus 90	KY197566	Vesala et al. 2017	Kenya	A	G01
Macrotermes subhyalinus 91	KY197567	Vesala et al. 2017	Kenya	A	G01
Macrotermes subhyalinus 92	KY197570	Vesala et al. 2017	Kenya	A	G01
Macrotermes subhyalinus 93	KY197571	Vesala et al. 2017	Kenya	A	G01
Macrotermes subhyalinus 94	KY197572	Vesala et al. 2017	Kenya	A	G01
Macrotermes subhyalinus 95	KY197573	Vesala et al. 2017	Kenya	A	G01
Macrotermes subhyalinus 96	KY197577	Vesala et al. 2017	Kenya	A	G01
Macrotermes subhyalinus 97	KY197578	Vesala et al. 2017	Kenya	A	G01
Macrotermes subhyalinus 98	KY197579	Vesala et al. 2017	Kenya	A	G01
Macrotermes subhyalinus 99	KY197580	Vesala et al. 2017	Kenya	A	G01

Macrotermes subhyalinus 100	KY197581	Vesala et al. 2017	Kenya	A	G01
Macrotermes subhyalinus 101	KY197584	Vesala et al. 2017	Kenya	A	G01
Macrotermes subhyalinus 102	KY197585	Vesala et al. 2017	Kenya	A	G01
Macrotermes subhyalinus 103	KY197587	Vesala et al. 2017	Kenya	A	G01
Macrotermes subhyalinus 104	KY197589	Vesala et al. 2017	Kenya	A	G01
Macrotermes subhyalinus 105	KY197590	Vesala et al. 2017	Kenya	A	G01
Macrotermes subhyalinus 106	KY197598	Vesala et al. 2017	Kenya	A	G01
Macrotermes subhyalinus 107	KY197575	Vesala et al. 2017	Kenya	A	G01
Macrotermes subhyalinus 108	KY197583	Vesala et al. 2017	Kenya	A	G01
Macrotermes subhyalinus 109	KY197486	Vesala et al. 2017	Kenya	A	G01
Macrotermes subhyalinus 110	KY197489	Vesala et al. 2017	Kenya	A	G01
Macrotermes subhyalinus 111	KY197493	Vesala et al. 2017	Kenya	A	G01
Macrotermes subhyalinus 112	Y197497	Vesala et al. 2017	Kenya	A	G01
Macrotermes subhyalinus 113	KY197519	Vesala et al. 2017	Kenya	A	G01
Macrotermes subhyalinus 114	KY197488	Vesala et al. 2017	Kenya	A	G01
Macrotermes subhyalinus 115	KY197492	Vesala et al. 2017	Kenya	A	G01
Macrotermes subhyalinus 116	KY197494	Vesala et al. 2017	Kenya	A	G01
Macrotermes subhyalinus 117	KY197508	Vesala et al. 2017	Kenya	A	G01
Macrotermes subhyalinus 118	KY197509	Vesala et al. 2017	Kenya	A	G01
Macrotermes subhyalinus 119	KY197510	Vesala et al. 2017	Kenya	A	G01
Macrotermes subhyalinus 120	KY197511	Vesala et al. 2017	Kenya	A	G01
Macrotermes subhyalinus 121	KY197512	Vesala et al. 2017	Kenya	A	G01
Macrotermes subhyalinus 122	KY197513	Vesala et al. 2017	Kenya	A	G01
Macrotermes subhyalinus 123	KY197515	Vesala et al. 2017	Kenya	A	G01
Macrotermes subhyalinus 124	KY197522	Vesala et al. 2017	Kenya	A	G01
Macrotermes subhyalinus 125	KY197525	Vesala et al. 2017	Kenya	A	G01
Macrotermes subhyalinus 126	KY197527	Vesala et al. 2017	Kenya	A	G01
Macrotermes subhyalinus 127	KY197539	Vesala et al. 2017	Kenya	A	G01
Macrotermes subhyalinus 128	KY197545	Vesala et al. 2017	Kenya	A	G01
Macrotermes subhyalinus 129	KY197546	Vesala et al. 2017	Kenya	A	G01

Macrotermes subhyalinus 130	KY197547	Vesala et al. 2017	Kenya	A	G01
Macrotermes subhyalinus 131	KY197549	Vesala et al. 2017	Kenya	A	G01
Macrotermes subhyalinus 132	KY197555	Vesala et al. 2017	Kenya	A	G01
Macrotermes subhyalinus 133	KY197564	Vesala et al. 2017	Kenya	A	G01
Macrotermes subhyalinus 134	KY197569	Vesala et al. 2017	Kenya	A	G01
Macrotermes subhyalinus 135	KY197496	Vesala et al. 2017	Kenya	A	G01
Macrotermes subhyalinus 136	KY197498	Vesala et al. 2017	Kenya	A	G01
Macrotermes subhyalinus 137	KY197499	Vesala et al. 2017	Kenya	A	G01
Macrotermes subhyalinus 138	KY197594	Vesala et al. 2017	Kenya	A	G01
Macrotermes subhyalinus 139	KY197595	Vesala et al. 2017	Kenya	A	G01
Macrotermes subhyalinus 140	KY197574	Vesala et al. 2017	Kenya	A	G01
Macrotermes subhyalinus 141	KY197576	Vesala et al. 2017	Kenya	A	G01
Macrotermes subhyalinus 142	KY197582	Vesala et al. 2017	Kenya	A	G01
Macrotermes subhyalinus 143	KY197586	Vesala et al. 2017	Kenya	A	G01
Macrotermes subhyalinus 144	KY197588	Vesala et al. 2017	Kenya	A	G01
Macrotermes subhyalinus 145	KY197591	Vesala et al. 2017	Kenya	A	G01
Macrotermes subhyalinus 146	Y197568	Vesala et al. 2017	Kenya	A	G01
Macrotermes subhyalinus 147	KY197517	Vesala et al. 2017	Kenya	A	G01
Macrotermes subhyalinus 148	KY197523	Vesala et al. 2017	Kenya	A	G01
Macrotermes subhyalinus mitogenome	NC_018128	Cameron et al. 2012	Not reported	A	G11
Macrotermes vitrialatus mitogenome	NC_034054	Bourguignon et al. 2017	Burundi	B	G12
FT01	MZ323635	This study	South Africa	A	G03
FT02	MZ323636	This study	South Africa	A	G03
FT03	MZ323637	This study	South Africa	A	G07
FT04	MZ323638	This study	South Africa	A	G03
FT05	MZ323639	This study	South Africa	A	G07
FT06	MZ323640	This study	South Africa	A	G03
FT07	MZ323641	This study	South Africa	A	G06
FT08	MZ323642	This study	South Africa	A	G05

FT09	MZ323643	This study	South Africa	A	G06
FT10	MZ323644	This study	South Africa	A	G06
FT11	MZ323645	This study	South Africa	A	G06
FT12	MZ323646	This study	South Africa	A	G05
FT13	MZ323647	This study	South Africa	A	G05
FT14	MZ323648	This study	South Africa	A	G06
FT15	MZ323649	This study	South Africa	A	G06
MF01	MZ323650	This study	South Africa	A	G03
MF02	MZ323651	This study	South Africa	A	G03
MF03	MZ323652	This study	South Africa	A	G03
MF04	MZ323653	This study	South Africa	A	G03
MF05	MZ323654	This study	South Africa	A	G03
MF06	MZ323655	This study	South Africa	A	G03
MF07	MZ323656	This study	South Africa	A	G03
MF08	MZ323657	This study	South Africa	A	G03
MF09	MZ323658	This study	South Africa	A	G03
MF10	MZ323659	This study	South Africa	A	G03
MF39	MZ323660	This study	South Africa	A	G03
MF40	MZ323661	This study	South Africa	A	G03
MF41	MZ323662	This study	South Africa	A	G03
MF42	MZ323663	This study	South Africa	A	G03
MF43	MZ323664	This study	South Africa	A	G03
MF49	MZ323665	This study	South Africa	A	G07
MF50	MZ323666	This study	South Africa	A	G07
MF51	MZ323667	This study	South Africa	A	G07
MF52	MZ323668	This study	South Africa	A	G07

Table S3. Multiple sequence alignment of 228 African *Macrotermes*, including new and publicly available COI sequences.

>G1_Macrotermes_subhyalinus_27 Macrotermes subhyalinus isolate TY06 cytochrome oxidase subunit I (COI) gene, partial cds; mitochondrial

----

AACAGAACTTGGACAACCAGGATCCTTAATCGGGGACGACCAAATCTACAACGTCATCGTCACAGCTCACGCTTTCGTAATGATCTTCTTCATAGTAATACCAATTATGATTGG

AGGATTCGGAAACTGACTAGTACCACTAATATTAGGAGCACCAGACATAGCATTCCCACGAATAAACAACATAAGATTCTGATTATTACCACCATCATTAACTCTTCTTCTCACT

AGTAGAACAGTAGAAAGTGGTGCAGGAACAGGATGAACAGTATACCCACCCCTTGCAAGAGGAATTGCCCACGCCGGAGCATCAGTAGATCTAGCCATCTTCTCATTACACTT

AGCAGGAGTATCATCCATCCTAGGAGCAGTAAACTTCATCTCAACAACAATCAACATGAAACCAAAAAACATAAAACCCGAACGAATCCCCTTATTCGTATGATCAGTTGCCAT

CACGGCTCTCCTACTCCTCCTATCACTACCAGTACTAGCAGGAGCAATCACAATACTATTAACTGACCGAAACCTAAACACATCCTTCTTTGATCCAGCAGGAGGTGGAGACCC

AATCCTATACCAACACTTATTCTGATTCTTCGGACACCCAGAAGTATATATTTTAATCCTACCAGGATTTGGTATAATTTCCCACATTATTTGTCACGAAAGAGGTAAAAAGGAA

GCCTTCGGAAACCTAGGAATAATTTTCGCCATATTAGCAATCGGATTACTAGGATTTGTAGTATGAGCACACCATATGTTCACAGTAGGAATAGACGTTGACACACGAGCTTAC

TTTACATCAGCAACAATAATCATTGCAGTACCTACGGGGATTAAAATCTTCAGATGACTTGCAACAATATACGGAACCCGAATAACTTATAGAGCAGCATGCCTATGAGCCCTA

GGATTTGTA--

>G1_Macrotermes_subhyalinus_28 Macrotermes subhyalinus isolate TY09 cytochrome oxidase subunit I (COI) gene, partial cds; mitochondrial

----

AACAGAACTTGGACAACCAGGATCCTTAATCGGGGACGACCAAATCTACAACGTCATCGTCACAGCTCACGCTTTCGTAATGATCTTCTTCATAGTAATACCAATTATGATTGG

AGGATTCGGAAACTGACTAGTACCACTAATATTAGGAGCACCAGACATAGCATTCCCACGAATAAACAACATAAGATTCTGATTATTACCACCATCATTAACTCTTCTTCTCACT

AGTAGAACAGTAGAAAGTGGTGCAGGAACAGGATGAACAGTATACCCACCCCTTGCAAGAGGAATTGCCCACGCCGGAGCATCAGTAGATCTAGCCATCTTCTCATTACACTT

AGCAGGAGTATCATCCATCCTAGGAGCAGTAAACTTCATCTCAACAACAATCAACATGAAACCAAAAAACATAAAACCCGAACGAATCCCCTTATTCGTATGATCAGTTGCCAT

CACGGCTCTCCTACTCCTCCTATCACTACCAGTACTAGCAGGAGCAATCACAATACTATTAACTGACCGAAACCTAAACACATCCTTCTTTGATCCAGCAGGAGGTGGAGACCC

AATCCTATACCAACACTTATTCTGATTCTTCGGACACCCAGAAGTATATATTTTAATCCTACCAGGATTTGGTATAATTTCCCACATTATTTGTCACGAAAGAGGTAAAAAGGAA

GCCTTCGGAAACCTAGGAATAATTTTCGCCATATTAGCAATCGGATTACTAGGATTTGTAGTATGAGCACACCATATGTTCACAGTAGGAATAGACGTTGACACACGAGCTTAC

TTTACATCAGCAACAATAATCATTGCAGTACCTACGGGGATTAAAATCTTCAGATGACTTGCAACAATATACGGAACCCGAATAACTTATAGAGCAGCATGCCTATGAGCCCTA

GGATTTGTA--

>G1_Macrotermes_subhyalinus_29 Macrotermes subhyalinus isolate TY13 cytochrome oxidase subunit I (COI) gene, partial cds; mitochondrial

----

AACAGAACTTGGACAACCAGGATCCTTAATCGGGGACGACCAAATCTACAACGTCATCGTCACAGCTCACGCTTTCGTAATGATCTTCTTCATAGTAATACCAATTATGATTGG

AGGATTCGGAAACTGACTAGTACCACTAATATTAGGAGCACCAGACATAGCATTCCCACGAATAAACAACATAAGATTCTGATTATTACCACCATCATTAACTCTTCTTCTCACT

AGTAGAACAGTAGAAAGTGGTGCAGGAACAGGATGAACAGTATACCCACCCCTTGCAAGAGGAATTGCCCACGCCGGAGCATCAGTAGATCTAGCCATCTTCTCATTACACTT

AGCAGGAGTATCATCCATCCTAGGAGCAGTAAACTTCATCTCAACAACAATCAACATGAAACCAAAAAACATAAAACCCGAACGAATCCCCTTATTCGTATGATCAGTTGCCAT

CACGGCTCTCCTACTCCTCCTATCACTACCAGTACTAGCAGGAGCAATCACAATACTATTAACTGACCGAAACCTAAACACATCCTTCTTTGATCCAGCAGGAGGTGGAGACCC

AATCCTATACCAACACTTATTCTGATTCTTCGGACACCCAGAAGTATATATTTTAATCCTACCAGGATTTGGTATAATTTCCCACATTATTTGTCACGAAAGAGGTAAAAAGGAA

GCCTTCGGAAACCTAGGAATAATTTTCGCCATATTAGCAATCGGATTACTAGGATTTGTAGTATGAGCACACCATATGTTCACAGTAGGAATAGACGTTGACACACGAGCTTAC

TTTACATCAGCAACAATAATCATTGCAGTACCTACGGGGATTAAAATCTTCAGATGACTTGCAACAATATACGGAACCCGAATAACTTATAGAGCAGCATGCCTATGAGCCCTA

GGATTTGTA--

>G1_Macrotermes_subhyalinus_30 Macrotermes subhyalinus isolate TY14 cytochrome oxidase subunit I (COI) gene, partial cds; mitochondrial

----

AACAGAACTTGGACAACCAGGATCCTTAATCGGGGACGACCAAATCTACAACGTCATCGTCACAGCTCACGCTTTCGTAATGATCTTCTTCATAGTAATACCAATTATGATTGG

AGGATTCGGAAACTGACTAGTACCACTAATATTAGGAGCACCAGACATAGCATTCCCACGAATAAACAACATAAGATTCTGATTATTACCACCATCATTAACTCTTCTTCTCACT

AGTAGAACAGTAGAAAGTGGTGCAGGAACAGGATGAACAGTATACCCACCCCTTGCAAGAGGAATTGCCCACGCCGGAGCATCAGTAGATCTAGCCATCTTCTCATTACACTT

AGCAGGAGTATCATCCATCCTAGGAGCAGTAAACTTCATCTCAACAACAATCAACATGAAACCAAAAAACATAAAACCCGAACGAATCCCCTTATTCGTATGATCAGTTGCCAT

CACGGCTCTCCTACTCCTCCTATCACTACCAGTACTAGCAGGAGCAATCACAATACTATTAACTGACCGAAACCTAAACACATCCTTCTTTGATCCAGCAGGAGGTGGAGACCC

AATCCTATACCAACACTTATTCTGATTCTTCGGACACCCAGAAGTATATATTTTAATCCTACCAGGATTTGGTATAATTTCCCACATTATTTGTCACGAAAGAGGTAAAAAGGAA

GCCTTCGGAAACCTAGGAATAATTTTCGCCATATTAGCAATCGGATTACTAGGATTTGTAGTATGAGCACACCATATGTTCACAGTAGGAATAGACGTTGACACACGAGCTTAC

TTTACATCAGCAACAATAATCATTGCAGTACCTACGGGGATTAAAATCTTCAGATGACTTGCAACAATATACGGAACCCGAATAACTTATAGAGCAGCATGCCTATGAGCCCTA

GGATTTGTA--

>G1_Macrotermes_subhyalinus_31 Macrotermes subhyalinus isolate TS05 cytochrome oxidase subunit I (COI) gene, partial cds; mitochondrial

----

AACAGAACTTGGACAACCAGGATCCTTAATCGGGGACGACCAAATCTACAACGTCATCGTCACAGCTCACGCTTTCGTAATGATCTTCTTCATAGTAATACCAATTATGATTGG

AGGATTCGGAAACTGACTAGTACCACTAATATTAGGAGCACCAGACATAGCATTCCCACGAATAAACAACATAAGATTCTGATTATTACCACCATCATTAACTCTTCTTCTCACT

AGTAGAACAGTAGAAAGTGGTGCAGGAACAGGATGAACAGTATACCCACCCCTTGCAAGAGGAATTGCCCACGCCGGAGCATCAGTAGATCTAGCCATCTTCTCATTACACTT

AGCAGGAGTATCATCCATCCTAGGAGCAGTAAACTTCATCTCAACAACAATCAACATGAAACCAAAAAACATAAAACCCGAACGAATCCCCTTATTCGTATGATCAGTTGCCAT

CACGGCTCTCCTACTCCTCCTATCACTACCAGTACTAGCAGGAGCAATCACAATACTATTAACTGACCGAAACCTAAACACATCCTTCTTTGATCCAGCAGGAGGTGGAGACCC

AATCCTATACCAACACTTATTCTGATTCTTCGGACACCCAGAAGTATATATTTTAATCCTACCAGGATTTGGTATAATTTCCCACATTATTTGTCACGAAAGAGGTAAAAAGGAA

GCCTTCGGAAACCTAGGAATAATTTTCGCCATATTAGCAATCGGATTACTAGGATTTGTAGTATGAGCACACCATATGTTCACAGTAGGAATAGACGTTGACACACGAGCTTAC

TTTACATCAGCAACAATAATCATTGCAGTACCTACGGGGATTAAAATCTTCAGATGACTTGCAACAATATACGGAACCCGAATAACTTATAGAGCAGCATGCCTATGAGCCCTA

GGATTTGTA---

>G1_Macrotermes_subhyalinus_32 Macrotermes subhyalinus isolate TS08 cytochrome oxidase subunit I (COI) gene, partial cds; mitochondrial

----

AACAGAACTTGGACAACCAGGATCCTTAATCGGGGACGACCAAATCTACAACGTCATCGTCACAGCTCACGCTTTCGTAATGATCTTCTTCATAGTAATACCAATTATGATTGG

AGGATTCGGAAACTGACTAGTACCACTAATATTAGGAGCACCAGACATAGCATTCCCACGAATAAACAACATAAGATTCTGATTATTACCACCATCATTAACTCTTCTTCTCACT

AGTAGAACAGTAGAAAGTGGTGCAGGAACAGGATGAACAGTATACCCACCCCTTGCAAGAGGAATTGCCCACGCCGGAGCATCAGTAGATCTAGCCATCTTCTCATTACACTT

AGCAGGAGTATCATCCATCCTAGGAGCAGTAAACTTCATCTCAACAACAATCAACATGAAACCAAAAAACATAAAACCCGAACGAATCCCCTTATTCGTATGATCAGTTGCCAT

CACGGCTCTCCTACTCCTCCTATCACTACCAGTACTAGCAGGAGCAATCACAATACTATTAACTGACCGAAACCTAAACACATCCTTCTTTGATCCAGCAGGAGGTGGAGACCC

AATCCTATACCAACACTTATTCTGATTCTTCGGACACCCAGAAGTATATATTTTAATCCTACCAGGATTTGGTATAATTTCCCACATTATTTGTCACGAAAGAGGTAAAAAGGAA

GCCTTCGGAAACCTAGGAATAATTTTCGCCATATTAGCAATCGGATTACTAGGATTTGTAGTATGAGCACACCATATGTTCACAGTAGGAATAGACGTTGACACACGAGCTTAC

TTTACATCAGCAACAATAATCATTGCAGTACCTACGGGGATTAAAATCTTCAGATGACTTGCAACAATATACGGAACCCGAATAACTTATAGAGCAGCATGCCTATGAGCCCTA

GGATTTGTA---

>G1_Macrotermes_subhyalinus_33 Macrotermes subhyalinus isolate TS09 cytochrome oxidase subunit I (COI) gene, partial cds; mitochondrial

----

AACAGAACTTGGACAACCAGGATCCTTAATCGGGGACGACCAAATCTACAACGTCATCGTCACAGCTCACGCTTTCGTAATGATCTTCTTCATAGTAATACCAATTATGATTGG

AGGATTCGGAAACTGACTAGTACCACTAATATTAGGAGCACCAGACATAGCATTCCCACGAATAAACAACATAAGATTCTGATTATTACCACCATCATTAACTCTTCTTCTCACT

AGTAGAACAGTAGAAAGTGGTGCAGGAACAGGATGAACAGTATACCCACCCCTTGCAAGAGGAATTGCCCACGCCGGAGCATCAGTAGATCTAGCCATCTTCTCATTACACTT

AGCAGGAGTATCATCCATCCTAGGAGCAGTAAACTTCATCTCAACAACAATCAACATGAAACCAAAAAACATAAAACCCGAACGAATCCCCTTATTCGTATGATCAGTTGCCAT

CACGGCTCTCCTACTCCTCCTATCACTACCAGTACTAGCAGGAGCAATCACAATACTATTAACTGACCGAAACCTAAACACATCCTTCTTTGATCCAGCAGGAGGTGGAGACCC

AATCCTATACCAACACTTATTCTGATTCTTCGGACACCCAGAAGTATATATTTTAATCCTACCAGGATTTGGTATAATTTCCCACATTATTTGTCACGAAAGAGGTAAAAAGGAA

GCCTTCGGAAACCTAGGAATAATTTTCGCCATATTAGCAATCGGATTACTAGGATTTGTAGTATGAGCACACCATATGTTCACAGTAGGAATAGACGTTGACACACGAGCTTAC

TTTACATCAGCAACAATAATCATTGCAGTACCTACGGGGATTAAAATCTTCAGATGACTTGCAACAATATACGGAACCCGAATAACTTATAGAGCAGCATGCCTATGAGCCCTA

GGATTTGTA--

>G1_Macrotermes_subhyalinus_34 Macrotermes subhyalinus isolate TS10 cytochrome oxidase subunit I (COI) gene, partial cds; mitochondrial

----

AACAGAACTTGGACAACCAGGATCCTTAATCGGGGACGACCAAATCTACAACGTCATCGTCACAGCTCACGCTTTCGTAATGATCTTCTTCATAGTAATACCAATTATGATTGG

AGGATTCGGAAACTGACTAGTACCACTAATATTAGGAGCACCAGACATAGCATTCCCACGAATAAACAACATAAGATTCTGATTATTACCACCATCATTAACTCTTCTTCTCACT

AGTAGAACAGTAGAAAGTGGTGCAGGAACAGGATGAACAGTATACCCACCCCTTGCAAGAGGAATTGCCCACGCCGGAGCATCAGTAGATCTAGCCATCTTCTCATTACACTT

AGCAGGAGTATCATCCATCCTAGGAGCAGTAAACTTCATCTCAACAACAATCAACATGAAACCAAAAAACATAAAACCCGAACGAATCCCCTTATTCGTATGATCAGTTGCCAT

CACGGCTCTCCTACTCCTCCTATCACTACCAGTACTAGCAGGAGCAATCACAATACTATTAACTGACCGAAACCTAAACACATCCTTCTTTGATCCAGCAGGAGGTGGAGACCC

AATCCTATACCAACACTTATTCTGATTCTTCGGACACCCAGAAGTATATATTTTAATCCTACCAGGATTTGGTATAATTTCCCACATTATTTGTCACGAAAGAGGTAAAAAGGAA

GCCTTCGGAAACCTAGGAATAATTTTCGCCATATTAGCAATCGGATTACTAGGATTTGTAGTATGAGCACACCATATGTTCACAGTAGGAATAGACGTTGACACACGAGCTTAC

TTTACATCAGCAACAATAATCATTGCAGTACCTACGGGGATTAAAATCTTCAGATGACTTGCAACAATATACGGAACCCGAATAACTTATAGAGCAGCATGCCTATGAGCCCTA

GGATTTGTA--

>G1_Macrotermes_subhyalinus_35 Macrotermes subhyalinus isolate TS13 cytochrome oxidase subunit I (COI) gene, partial cds; mitochondrial

----

AACAGAACTTGGACAACCAGGATCCTTAATCGGGGACGACCAAATCTACAACGTCATCGTCACAGCTCACGCTTTCGTAATGATCTTCTTCATAGTAATACCAATTATGATTGG

AGGATTCGGAAACTGACTAGTACCACTAATATTAGGAGCACCAGACATAGCATTCCCACGAATAAACAACATAAGATTCTGATTATTACCACCATCATTAACTCTTCTTCTCACT

AGTAGAACAGTAGAAAGTGGTGCAGGAACAGGATGAACAGTATACCCACCCCTTGCAAGAGGAATTGCCCACGCCGGAGCATCAGTAGATCTAGCCATCTTCTCATTACACTT

AGCAGGAGTATCATCCATCCTAGGAGCAGTAAACTTCATCTCAACAACAATCAACATGAAACCAAAAAACATAAAACCCGAACGAATCCCCTTATTCGTATGATCAGTTGCCAT

CACGGCTCTCCTACTCCTCCTATCACTACCAGTACTAGCAGGAGCAATCACAATACTATTAACTGACCGAAACCTAAACACATCCTTCTTTGATCCAGCAGGAGGTGGAGACCC

AATCCTATACCAACACTTATTCTGATTCTTCGGACACCCAGAAGTATATATTTTAATCCTACCAGGATTTGGTATAATTTCCCACATTATTTGTCACGAAAGAGGTAAAAAGGAA

GCCTTCGGAAACCTAGGAATAATTTTCGCCATATTAGCAATCGGATTACTAGGATTTGTAGTATGAGCACACCATATGTTCACAGTAGGAATAGACGTTGACACACGAGCTTAC

TTTACATCAGCAACAATAATCATTGCAGTACCTACGGGGATTAAAATCTTCAGATGACTTGCAACAATATACGGAACCCGAATAACTTATAGAGCAGCATGCCTATGAGCCCTA

GGATTTGTA--

>G1_Macrotermes_subhyalinus_36 Macrotermes subhyalinus isolate TS14 cytochrome oxidase subunit I (COI) gene, partial cds; mitochondrial

----

AACAGAACTTGGACAACCAGGATCCTTAATCGGGGACGACCAAATCTACAACGTCATCGTCACAGCTCACGCTTTCGTAATGATCTTCTTCATAGTAATACCAATTATGATTGG

AGGATTCGGAAACTGACTAGTACCACTAATATTAGGAGCACCAGACATAGCATTCCCACGAATAAACAACATAAGATTCTGATTATTACCACCATCATTAACTCTTCTTCTCACT

AGTAGAACAGTAGAAAGTGGTGCAGGAACAGGATGAACAGTATACCCACCCCTTGCAAGAGGAATTGCCCACGCCGGAGCATCAGTAGATCTAGCCATCTTCTCATTACACTT

AGCAGGAGTATCATCCATCCTAGGAGCAGTAAACTTCATCTCAACAACAATCAACATGAAACCAAAAAACATAAAACCCGAACGAATCCCCTTATTCGTATGATCAGTTGCCAT

CACGGCTCTCCTACTCCTCCTATCACTACCAGTACTAGCAGGAGCAATCACAATACTATTAACTGACCGAAACCTAAACACATCCTTCTTTGATCCAGCAGGAGGTGGAGACCC

AATCCTATACCAACACTTATTCTGATTCTTCGGACACCCAGAAGTATATATTTTAATCCTACCAGGATTTGGTATAATTTCCCACATTATTTGTCACGAAAGAGGTAAAAAGGAA

GCCTTCGGAAACCTAGGAATAATTTTCGCCATATTAGCAATCGGATTACTAGGATTTGTAGTATGAGCACACCATATGTTCACAGTAGGAATAGACGTTGACACACGAGCTTAC

TTTACATCAGCAACAATAATCATTGCAGTACCTACGGGGATTAAAATCTTCAGATGACTTGCAACAATATACGGAACCCGAATAACTTATAGAGCAGCATGCCTATGAGCCCTA

GGATTTGTA--

>G1_Macrotermes_subhyalinus_37 Macrotermes subhyalinus isolate TS20 cytochrome oxidase subunit I (COI) gene, partial cds; mitochondrial

----

AACAGAACTTGGACAACCAGGATCCTTAATCGGGGACGACCAAATCTACAACGTCATCGTCACAGCTCACGCTTTCGTAATGATCTTCTTCATAGTAATACCAATTATGATTGG

AGGATTCGGAAACTGACTAGTACCACTAATATTAGGAGCACCAGACATAGCATTCCCACGAATAAACAACATAAGATTCTGATTATTACCACCATCATTAACTCTTCTTCTCACT

AGTAGAACAGTAGAAAGTGGTGCAGGAACAGGATGAACAGTATACCCACCCCTTGCAAGAGGAATTGCCCACGCCGGAGCATCAGTAGATCTAGCCATCTTCTCATTACACTT

AGCAGGAGTATCATCCATCCTAGGAGCAGTAAACTTCATCTCAACAACAATCAACATGAAACCAAAAAACATAAAACCCGAACGAATCCCCTTATTCGTATGATCAGTTGCCAT

CACGGCTCTCCTACTCCTCCTATCACTACCAGTACTAGCAGGAGCAATCACAATACTATTAACTGACCGAAACCTAAACACATCCTTCTTTGATCCAGCAGGAGGTGGAGACCC

AATCCTATACCAACACTTATTCTGATTCTTCGGACACCCAGAAGTATATATTTTAATCCTACCAGGATTTGGTATAATTTCCCACATTATTTGTCACGAAAGAGGTAAAAAGGAA

GCCTTCGGAAACCTAGGAATAATTTTCGCCATATTAGCAATCGGATTACTAGGATTTGTAGTATGAGCACACCATATGTTCACAGTAGGAATAGACGTTGACACACGAGCTTAC

TTTACATCAGCAACAATAATCATTGCAGTACCTACGGGGATTAAAATCTTCAGATGACTTGCAACAATATACGGAACCCGAATAACTTATAGAGCAGCATGCCTATGAGCCCTA

GGATTTGTA--

>G1_Macrotermes_subhyalinus_38 Macrotermes subhyalinus isolate TS56 cytochrome oxidase subunit I (COI) gene, partial cds; mitochondrial

----

AACAGAACTTGGACAACCAGGATCCTTAATCGGGGACGACCAAATCTACAACGTCATCGTCACAGCTCACGCTTTCGTAATGATCTTCTTCATAGTAATACCAATTATGATTGG

AGGATTCGGAAACTGACTAGTACCACTAATATTAGGAGCACCAGACATAGCATTCCCACGAATAAACAACATAAGATTCTGATTATTACCACCATCATTAACTCTTCTTCTCACT

AGTAGAACAGTAGAAAGTGGTGCAGGAACAGGATGAACAGTATACCCACCCCTTGCAAGAGGAATTGCCCACGCCGGAGCATCAGTAGATCTAGCCATCTTCTCATTACACTT

AGCAGGAGTATCATCCATCCTAGGAGCAGTAAACTTCATCTCAACAACAATCAACATGAAACCAAAAAACATAAAACCCGAACGAATCCCCTTATTCGTATGATCAGTTGCCAT

CACGGCTCTCCTACTCCTCCTATCACTACCAGTACTAGCAGGAGCAATCACAATACTATTAACTGACCGAAACCTAAACACATCCTTCTTTGATCCAGCAGGAGGTGGAGACCC

AATCCTATACCAACACTTATTCTGATTCTTCGGACACCCAGAAGTATATATTTTAATCCTACCAGGATTTGGTATAATTTCCCACATTATTTGTCACGAAAGAGGTAAAAAGGAA

GCCTTCGGAAACCTAGGAATAATTTTCGCCATATTAGCAATCGGATTACTAGGATTTGTAGTATGAGCACACCATATGTTCACAGTAGGAATAGACGTTGACACACGAGCTTAC

TTTACATCAGCAACAATAATCATTGCAGTACCTACGGGGATTAAAATCTTCAGATGACTTGCAACAATATACGGAACCCGAATAACTTATAGAGCAGCATGCCTATGAGCCCTA

GGATTTGTA--

>G1_Macrotermes_subhyalinus_39 Macrotermes subhyalinus isolate TK01 cytochrome oxidase subunit I (COI) gene, partial cds; mitochondrial

----

AACAGAACTTGGACAACCAGGATCCTTAATCGGGGACGACCAAATCTACAACGTCATCGTCACAGCTCACGCTTTCGTAATGATCTTCTTCATAGTAATACCAATTATGATTGG

AGGATTCGGAAACTGACTAGTACCACTAATATTAGGAGCACCAGACATAGCATTCCCACGAATAAACAACATAAGATTCTGATTATTACCACCATCATTAACTCTTCTTCTCACT

AGTAGAACAGTAGAAAGTGGTGCAGGAACAGGATGAACAGTATACCCACCCCTTGCAAGAGGAATTGCCCACGCCGGAGCATCAGTAGATCTAGCCATCTTCTCATTACACTT

AGCAGGAGTATCATCCATCCTAGGAGCAGTAAACTTCATCTCAACAACAATCAACATGAAACCAAAAAACATAAAACCCGAACGAATCCCCTTATTCGTATGATCAGTTGCCAT

CACGGCTCTCCTACTCCTCCTATCACTACCAGTACTAGCAGGAGCAATCACAATACTATTAACTGACCGAAACCTAAACACATCCTTCTTTGATCCAGCAGGAGGTGGAGACCC

AATCCTATACCAACACTTATTCTGATTCTTCGGACACCCAGAAGTATATATTTTAATCCTACCAGGATTTGGTATAATTTCCCACATTATTTGTCACGAAAGAGGTAAAAAGGAA

GCCTTCGGAAACCTAGGAATAATTTTCGCCATATTAGCAATCGGATTACTAGGATTTGTAGTATGAGCACACCATATGTTCACAGTAGGAATAGACGTTGACACACGAGCTTAC

TTTACATCAGCAACAATAATCATTGCAGTACCTACGGGGATTAAAATCTTCAGATGACTTGCAACAATATACGGAACCCGAATAACTTATAGAGCAGCATGCCTATGAGCCCTA

GGATTTGTA---

>G1_Macrotermes_subhyalinus_40 Macrotermes subhyalinus isolate TK04 cytochrome oxidase subunit I (COI) gene, partial cds; mitochondrial

----

AACAGAACTTGGACAACCAGGATCCTTAATCGGGGACGACCAAATCTACAACGTCATCGTCACAGCTCACGCTTTCGTAATGATCTTCTTCATAGTAATACCAATTATGATTGG

AGGATTCGGAAACTGACTAGTACCACTAATATTAGGAGCACCAGACATAGCATTCCCACGAATAAACAACATAAGATTCTGATTATTACCACCATCATTAACTCTTCTTCTCACT

AGTAGAACAGTAGAAAGTGGTGCAGGAACAGGATGAACAGTATACCCACCCCTTGCAAGAGGAATTGCCCACGCCGGAGCATCAGTAGATCTAGCCATCTTCTCATTACACTT

AGCAGGAGTATCATCCATCCTAGGAGCAGTAAACTTCATCTCAACAACAATCAACATGAAACCAAAAAACATAAAACCCGAACGAATCCCCTTATTCGTATGATCAGTTGCCAT

CACGGCTCTCCTACTCCTCCTATCACTACCAGTACTAGCAGGAGCAATCACAATACTATTAACTGACCGAAACCTAAACACATCCTTCTTTGATCCAGCAGGAGGTGGAGACCC

AATCCTATACCAACACTTATTCTGATTCTTCGGACACCCAGAAGTATATATTTTAATCCTACCAGGATTTGGTATAATTTCCCACATTATTTGTCACGAAAGAGGTAAAAAGGAA

GCCTTCGGAAACCTAGGAATAATTTTCGCCATATTAGCAATCGGATTACTAGGATTTGTAGTATGAGCACACCATATGTTCACAGTAGGAATAGACGTTGACACACGAGCTTAC

TTTACATCAGCAACAATAATCATTGCAGTACCTACGGGGATTAAAATCTTCAGATGACTTGCAACAATATACGGAACCCGAATAACTTATAGAGCAGCATGCCTATGAGCCCTA
GGATTTGTA---

>G1_Macrotermes_subhyalinus_41 Macrotermes subhyalinus isolate TK07 cytochrome oxidase subunit I (COI) gene, partial cds; mitochondrial

----
AACAGAACTTGGACAACCAGGATCCTTAATCGGGGACGACCAAATCTACAACGTCATCGTCACAGCTCACGCTTTCGTAATGATCTTCTTCATAGTAATACCAATTATGATTGG
AGGATTCGGAAACTGACTAGTACCACTAATATTAGGAGCACCAGACATAGCATTCCCACGAATAAACAACATAAGATTCTGATTATTACCACCATCATTAACTCTTCTTCTCACT
AGTAGAACAGTAGAAAGTGGTGCAGGAACAGGATGAACAGTATACCCACCCCTTGCAAGAGGAATTGCCCACGCCGGAGCATCAGTAGATCTAGCCATCTTCTCATTACACTT
AGCAGGAGTATCATCCATCCTAGGAGCAGTAAACTTCATCTCAACAACAATCAACATGAAACCAAAAAACATAAAACCCGAACGAATCCCCTTATTCGTATGATCAGTTGCCAT
CACGGCTCTCCTACTCCTCCTATCACTACCAGTACTAGCAGGAGCAATCACAATACTATTAACTGACCGAAACCTAAACACATCCTTCTTTGATCCAGCAGGAGGTGGAGACCC
AATCCTATACCAACACTTATTCTGATTCTTCGGACACCCAGAAGTATATATTTTAATCCTACCAGGATTTGGTATAATTTCCCACATTATTTGTCACGAAAGAGGTAAAAAGGAA
GCCTTCGGAAACCTAGGAATAATTTTCGCCATATTAGCAATCGGATTACTAGGATTTGTAGTATGAGCACACCATATGTTCACAGTAGGAATAGACGTTGACACACGAGCTTAC
TTTACATCAGCAACAATAATCATTGCAGTACCTACGGGGATTAAAATCTTCAGATGACTTGCAACAATATACGGAACCCGAATAACTTATAGAGCAGCATGCCTATGAGCCCTA
GGATTTGTA---

>G1_Macrotermes_subhyalinus_42 Macrotermes subhyalinus isolate TK08 cytochrome oxidase subunit I (COI) gene, partial cds; mitochondrial

----

AACAGAACTTGGACAACCAGGATCCTTAATCGGGGACGACCAAATCTACAACGTCATCGTCACAGCTCACGCTTTCGTAATGATCTTCTTCATAGTAATACCAATTATGATTGG

AGGATTCGGAAACTGACTAGTACCACTAATATTAGGAGCACCAGACATAGCATTCCCACGAATAAACAACATAAGATTCTGATTATTACCACCATCATTAACTCTTCTTCTCACT

AGTAGAACAGTAGAAAGTGGTGCAGGAACAGGATGAACAGTATACCCACCCCTTGCAAGAGGAATTGCCCACGCCGGAGCATCAGTAGATCTAGCCATCTTCTCATTACACTT

AGCAGGAGTATCATCCATCCTAGGAGCAGTAAACTTCATCTCAACAACAATCAACATGAAACCAAAAAACATAAAACCCGAACGAATCCCCTTATTCGTATGATCAGTTGCCAT

CACGGCTCTCCTACTCCTCCTATCACTACCAGTACTAGCAGGAGCAATCACAATACTATTAACTGACCGAAACCTAAACACATCCTTCTTTGATCCAGCAGGAGGTGGAGACCC

AATCCTATACCAACACTTATTCTGATTCTTCGGACACCCAGAAGTATATATTTTAATCCTACCAGGATTTGGTATAATTTCCCACATTATTTGTCACGAAAGAGGTAAAAAGGAA

GCCTTCGGAAACCTAGGAATAATTTTCGCCATATTAGCAATCGGATTACTAGGATTTGTAGTATGAGCACACCATATGTTCACAGTAGGAATAGACGTTGACACACGAGCTTAC

TTTACATCAGCAACAATAATCATTGCAGTACCTACGGGGATTAAAATCTTCAGATGACTTGCAACAATATACGGAACCCGAATAACTTATAGAGCAGCATGCCTATGAGCCCTA

GGATTTGTA--

>G1_Macrotermes_subhyalinus_43 Macrotermes subhyalinus isolate TK12 cytochrome oxidase subunit I (COI) gene, partial cds; mitochondrial

----

AACAGAACTTGGACAACCAGGATCCTTAATCGGGGACGACCAAATCTACAACGTCATCGTCACAGCTCACGCTTTCGTAATGATCTTCTTCATAGTAATACCAATTATGATTGG

AGGATTCGGAAACTGACTAGTACCACTAATATTAGGAGCACCAGACATAGCATTCCCACGAATAAACAACATAAGATTCTGATTATTACCACCATCATTAACTCTTCTTCTCACT

AGTAGAACAGTAGAAAGTGGTGCAGGAACAGGATGAACAGTATACCCACCCCTTGCAAGAGGAATTGCCCACGCCGGAGCATCAGTAGATCTAGCCATCTTCTCATTACACTT

AGCAGGAGTATCATCCATCCTAGGAGCAGTAAACTTCATCTCAACAACAATCAACATGAAACCAAAAAACATAAAACCCGAACGAATCCCCTTATTCGTATGATCAGTTGCCAT

CACGGCTCTCCTACTCCTCCTATCACTACCAGTACTAGCAGGAGCAATCACAATACTATTAACTGACCGAAACCTAAACACATCCTTCTTTGATCCAGCAGGAGGTGGAGACCC

AATCCTATACCAACACTTATTCTGATTCTTCGGACACCCAGAAGTATATATTTTAATCCTACCAGGATTTGGTATAATTTCCCACATTATTTGTCACGAAAGAGGTAAAAAGGAA

GCCTTCGGAAACCTAGGAATAATTTTCGCCATATTAGCAATCGGATTACTAGGATTTGTAGTATGAGCACACCATATGTTCACAGTAGGAATAGACGTTGACACACGAGCTTAC

TTTACATCAGCAACAATAATCATTGCAGTACCTACGGGGATTAAAATCTTCAGATGACTTGCAACAATATACGGAACCCGAATAACTTATAGAGCAGCATGCCTATGAGCCCTA
GGATTTGTA--

>G1_Macrotermes_subhyalinus_44 Macrotermes subhyalinus isolate TK17 cytochrome oxidase subunit I (COI) gene, partial cds; mitochondrial

----
AACAGAACTTGGACAACCAGGATCCTTAATCGGGGACGACCAAATCTACAACGTCATCGTCACAGCTCACGCTTTCGTAATGATCTTCTTCATAGTAATACCAATTATGATTGG
AGGATTCGGAAACTGACTAGTACCACTAATATTAGGAGCACCAGACATAGCATTCCCACGAATAAACAACATAAGATTCTGATTATTACCACCATCATTAACTCTTCTTCTCACT
AGTAGAACAGTAGAAAGTGGTGCAGGAACAGGATGAACAGTATACCCACCCCTTGCAAGAGGAATTGCCCACGCCGGAGCATCAGTAGATCTAGCCATCTTCTCATTACACTT
AGCAGGAGTATCATCCATCCTAGGAGCAGTAAACTTCATCTCAACAACAATCAACATGAAACCAAAAAACATAAAACCCGAACGAATCCCCTTATTCGTATGATCAGTTGCCAT
CACGGCTCTCCTACTCCTCCTATCACTACCAGTACTAGCAGGAGCAATCACAATACTATTAACTGACCGAAACCTAAACACATCCTTCTTTGATCCAGCAGGAGGTGGAGACCC
AATCCTATACCAACACTTATTCTGATTCTTCGGACACCCAGAAGTATATATTTTAATCCTACCAGGATTTGGTATAATTTCCCACATTATTTGTCACGAAAGAGGTAAAAAGGAA
GCCTTCGGAAACCTAGGAATAATTTTCGCCATATTAGCAATCGGATTACTAGGATTTGTAGTATGAGCACACCATATGTTCACAGTAGGAATAGACGTTGACACACGAGCTTAC
TTTACATCAGCAACAATAATCATTGCAGTACCTACGGGGATTAAAATCTTCAGATGACTTGCAACAATATACGGAACCCGAATAACTTATAGAGCAGCATGCCTATGAGCCCTA
GGATTTGTA--

>G1_Macrotermes_subhyalinus_45 Macrotermes subhyalinus isolate TK18 cytochrome oxidase subunit I (COI) gene, partial cds; mitochondrial

----

AACAGAACTTGGACAACCAGGATCCTTAATCGGGGACGACCAAATCTACAACGTCATCGTCACAGCTCACGCTTTCGTAATGATCTTCTTCATAGTAATACCAATTATGATTGG

AGGATTCGGAAACTGACTAGTACCACTAATATTAGGAGCACCAGACATAGCATTCCCACGAATAAACAACATAAGATTCTGATTATTACCACCATCATTAACTCTTCTTCTCACT

AGTAGAACAGTAGAAAGTGGTGCAGGAACAGGATGAACAGTATACCCACCCCTTGCAAGAGGAATTGCCCACGCCGGAGCATCAGTAGATCTAGCCATCTTCTCATTACACTT

AGCAGGAGTATCATCCATCCTAGGAGCAGTAAACTTCATCTCAACAACAATCAACATGAAACCAAAAAACATAAAACCCGAACGAATCCCCTTATTCGTATGATCAGTTGCCAT

CACGGCTCTCCTACTCCTCCTATCACTACCAGTACTAGCAGGAGCAATCACAATACTATTAACTGACCGAAACCTAAACACATCCTTCTTTGATCCAGCAGGAGGTGGAGACCC

AATCCTATACCAACACTTATTCTGATTCTTCGGACACCCAGAAGTATATATTTTAATCCTACCAGGATTTGGTATAATTTCCCACATTATTTGTCACGAAAGAGGTAAAAAGGAA

GCCTTCGGAAACCTAGGAATAATTTTCGCCATATTAGCAATCGGATTACTAGGATTTGTAGTATGAGCACACCATATGTTCACAGTAGGAATAGACGTTGACACACGAGCTTAC

TTTACATCAGCAACAATAATCATTGCAGTACCTACGGGGATTAAAATCTTCAGATGACTTGCAACAATATACGGAACCCGAATAACTTATAGAGCAGCATGCCTATGAGCCCTA

GGATTTGTA---

>G1_Macrotermes_subhyalinus_46 Macrotermes subhyalinus isolate TK19 cytochrome oxidase subunit I (COI) gene, partial cds; mitochondrial

----

AACAGAACTTGGACAACCAGGATCCTTAATCGGGGACGACCAAATCTACAACGTCATCGTCACAGCTCACGCTTTCGTAATGATCTTCTTCATAGTAATACCAATTATGATTGG

AGGATTCGGAAACTGACTAGTACCACTAATATTAGGAGCACCAGACATAGCATTCCCACGAATAAACAACATAAGATTCTGATTATTACCACCATCATTAACTCTTCTTCTCACT

AGTAGAACAGTAGAAAGTGGTGCAGGAACAGGATGAACAGTATACCCACCCCTTGCAAGAGGAATTGCCCACGCCGGAGCATCAGTAGATCTAGCCATCTTCTCATTACACTT

AGCAGGAGTATCATCCATCCTAGGAGCAGTAAACTTCATCTCAACAACAATCAACATGAAACCAAAAAACATAAAACCCGAACGAATCCCCTTATTCGTATGATCAGTTGCCAT

CACGGCTCTCCTACTCCTCCTATCACTACCAGTACTAGCAGGAGCAATCACAATACTATTAACTGACCGAAACCTAAACACATCCTTCTTTGATCCAGCAGGAGGTGGAGACCC

AATCCTATACCAACACTTATTCTGATTCTTCGGACACCCAGAAGTATATATTTTAATCCTACCAGGATTTGGTATAATTTCCCACATTATTTGTCACGAAAGAGGTAAAAAGGAA

GCCTTCGGAAACCTAGGAATAATTTTCGCCATATTAGCAATCGGATTACTAGGATTTGTAGTATGAGCACACCATATGTTCACAGTAGGAATAGACGTTGACACACGAGCTTAC

TTTACATCAGCAACAATAATCATTGCAGTACCTACGGGGATTAAAATCTTCAGATGACTTGCAACAATATACGGAACCCGAATAACTTATAGAGCAGCATGCCTATGAGCCCTA

GGATTTGTA---

>G1_Macrotermes_subhyalinus_47 Macrotermes subhyalinus isolate TK20 cytochrome oxidase subunit I (COI) gene, partial cds; mitochondrial

----

AACAGAACTTGGACAACCAGGATCCTTAATCGGGGACGACCAAATCTACAACGTCATCGTCACAGCTCACGCTTTCGTAATGATCTTCTTCATAGTAATACCAATTATGATTGG

AGGATTCGGAAACTGACTAGTACCACTAATATTAGGAGCACCAGACATAGCATTCCCACGAATAAACAACATAAGATTCTGATTATTACCACCATCATTAACTCTTCTTCTCACT

AGTAGAACAGTAGAAAGTGGTGCAGGAACAGGATGAACAGTATACCCACCCCTTGCAAGAGGAATTGCCCACGCCGGAGCATCAGTAGATCTAGCCATCTTCTCATTACACTT

AGCAGGAGTATCATCCATCCTAGGAGCAGTAAACTTCATCTCAACAACAATCAACATGAAACCAAAAAACATAAAACCCGAACGAATCCCCTTATTCGTATGATCAGTTGCCAT

CACGGCTCTCCTACTCCTCCTATCACTACCAGTACTAGCAGGAGCAATCACAATACTATTAACTGACCGAAACCTAAACACATCCTTCTTTGATCCAGCAGGAGGTGGAGACCC

AATCCTATACCAACACTTATTCTGATTCTTCGGACACCCAGAAGTATATATTTTAATCCTACCAGGATTTGGTATAATTTCCCACATTATTTGTCACGAAAGAGGTAAAAAGGAA

GCCTTCGGAAACCTAGGAATAATTTTCGCCATATTAGCAATCGGATTACTAGGATTTGTAGTATGAGCACACCATATGTTCACAGTAGGAATAGACGTTGACACACGAGCTTAC

TTTACATCAGCAACAATAATCATTGCAGTACCTACGGGGATTAAAATCTTCAGATGACTTGCAACAATATACGGAACCCGAATAACTTATAGAGCAGCATGCCTATGAGCCCTA

GGATTTGTA---

>G1_Macrotermes_subhyalinus_48 Macrotermes subhyalinus isolate TK21 cytochrome oxidase subunit I (COI) gene, partial cds; mitochondrial

----

AACAGAACTTGGACAACCAGGATCCTTAATCGGGGACGACCAAATCTACAACGTCATCGTCACAGCTCACGCTTTCGTAATGATCTTCTTCATAGTAATACCAATTATGATTGG
AGGATTCGGAAACTGACTAGTACCACTAATATTAGGAGCACCAGACATAGCATTCCCACGAATAAACAACATAAGATTCTGATTATTACCACCATCATTAACTCTTCTTCTCACT
AGTAGAACAGTAGAAAGTGGTGCAGGAACAGGATGAACAGTATACCCACCCCTTGCAAGAGGAATTGCCCACGCCGGAGCATCAGTAGATCTAGCCATCTTCTCATTACACTT
AGCAGGAGTATCATCCATCCTAGGAGCAGTAAACTTCATCTCAACAACAATCAACATGAAACCAAAAAACATAAAACCCGAACGAATCCCCTTATTCGTATGATCAGTTGCCAT
CACGGCTCTCCTACTCCTCCTATCACTACCAGTACTAGCAGGAGCAATCACAATACTATTAACTGACCGAAACCTAAACACATCCTTCTTTGATCCAGCAGGAGGTGGAGACCC
AATCCTATACCAACACTTATTCTGATTCTTCGGACACCCAGAAGTATATATTTTAATCCTACCAGGATTTGGTATAATTTCCCACATTATTTGTCACGAAAGAGGTAAAAAGGAA
GCCTTCGGAAACCTAGGAATAATTTTCGCCATATTAGCAATCGGATTACTAGGATTTGTAGTATGAGCACACCATATGTTCACAGTAGGAATAGACGTTGACACACGAGCTTAC
TTTACATCAGCAACAATAATCATTGCAGTACCTACGGGGATTAAAATCTTCAGATGACTTGCAACAATATACGGAACCCGAATAACTTATAGAGCAGCATGCCTATGAGCCCTA
GGATTTGTA--

>G1_Macrotermes_subhyalinus_49 Macrotermes subhyalinus isolate TK22 cytochrome oxidase subunit I (COI) gene, partial cds; mitochondrial

----

AACAGAACTTGGACAACCAGGATCCTTAATCGGGGACGACCAAATCTACAACGTCATCGTCACAGCTCACGCTTTCGTAATGATCTTCTTCATAGTAATACCAATTATGATTGG
AGGATTCGGAAACTGACTAGTACCACTAATATTAGGAGCACCAGACATAGCATTCCCACGAATAAACAACATAAGATTCTGATTATTACCACCATCATTAACTCTTCTTCTCACT
AGTAGAACAGTAGAAAGTGGTGCAGGAACAGGATGAACAGTATACCCACCCCTTGCAAGAGGAATTGCCCACGCCGGAGCATCAGTAGATCTAGCCATCTTCTCATTACACTT
AGCAGGAGTATCATCCATCCTAGGAGCAGTAAACTTCATCTCAACAACAATCAACATGAAACCAAAAAACATAAAACCCGAACGAATCCCCTTATTCGTATGATCAGTTGCCAT
CACGGCTCTCCTACTCCTCCTATCACTACCAGTACTAGCAGGAGCAATCACAATACTATTAACTGACCGAAACCTAAACACATCCTTCTTTGATCCAGCAGGAGGTGGAGACCC
AATCCTATACCAACACTTATTCTGATTCTTCGGACACCCAGAAGTATATATTTTAATCCTACCAGGATTTGGTATAATTTCCCACATTATTTGTCACGAAAGAGGTAAAAAGGAA
GCCTTCGGAAACCTAGGAATAATTTTCGCCATATTAGCAATCGGATTACTAGGATTTGTAGTATGAGCACACCATATGTTCACAGTAGGAATAGACGTTGACACACGAGCTTAC

TTTACATCAGCAACAATAATCATTGCAGTACCTACGGGGATTAAAATCTTCAGATGACTTGCAACAATATACGGAACCCGAATAACTTATAGAGCAGCATGCCTATGAGCCCTA

GGATTTGTA---

>G1_Macrotermes_subhyalinus_50 Macrotermes subhyalinus isolate TK23 cytochrome oxidase subunit I (COI) gene, partial cds; mitochondrial

----

AACAGAACTTGGACAACCAGGATCCTTAATCGGGGACGACCAAATCTACAACGTCATCGTCACAGCTCACGCTTTCGTAATGATCTTCTTCATAGTAATACCAATTATGATTGG

AGGATTCGGAAACTGACTAGTACCACTAATATTAGGAGCACCAGACATAGCATTCCCACGAATAAACAACATAAGATTCTGATTATTACCACCATCATTAACTCTTCTTCTCACT

AGTAGAACAGTAGAAAGTGGTGCAGGAACAGGATGAACAGTATACCCACCCCTTGCAAGAGGAATTGCCCACGCCGGAGCATCAGTAGATCTAGCCATCTTCTCATTACACTT

AGCAGGAGTATCATCCATCCTAGGAGCAGTAAACTTCATCTCAACAACAATCAACATGAAACCAAAAAACATAAAACCCGAACGAATCCCCTTATTCGTATGATCAGTTGCCAT

CACGGCTCTCCTACTCCTCCTATCACTACCAGTACTAGCAGGAGCAATCACAATACTATTAACTGACCGAAACCTAAACACATCCTTCTTTGATCCAGCAGGAGGTGGAGACCC

AATCCTATACCAACACTTATTCTGATTCTTCGGACACCCAGAAGTATATATTTTAATCCTACCAGGATTTGGTATAATTTCCCACATTATTTGTCACGAAAGAGGTAAAAAGGAA

GCCTTCGGAAACCTAGGAATAATTTTCGCCATATTAGCAATCGGATTACTAGGATTTGTAGTATGAGCACACCATATGTTCACAGTAGGAATAGACGTTGACACACGAGCTTAC

TTTACATCAGCAACAATAATCATTGCAGTACCTACGGGGATTAAAATCTTCAGATGACTTGCAACAATATACGGAACCCGAATAACTTATAGAGCAGCATGCCTATGAGCCCTA

GGATTTGTA---

>G1_Macrotermes_subhyalinus_51 Macrotermes subhyalinus isolate TR09 cytochrome oxidase subunit I (COI) gene, partial cds; mitochondrial

----

AACAGAACTTGGACAACCAGGATCCTTAATCGGGGACGACCAAATCTACAACGTCATCGTCACAGCTCACGCTTTCGTAATGATCTTCTTCATAGTAATACCAATTATGATTGG

AGGATTCGGAAACTGACTAGTACCACTAATATTAGGAGCACCAGACATAGCATTCCCACGAATAAACAACATAAGATTCTGATTATTACCACCATCATTAACTCTTCTTCTCACT

AGTAGAACAGTAGAAAGTGGTGCAGGAACAGGATGAACAGTATACCCACCCCTTGCAAGAGGAATTGCCCACGCCGGAGCATCAGTAGATCTAGCCATCTTCTCATTACACTT

AGCAGGAGTATCATCCATCCTAGGAGCAGTAAACTTCATCTCAACAACAATCAACATGAAACCAAAAAACATAAAACCCGAACGAATCCCCTTATTCGTATGATCAGTTGCCAT

CACGGCTCTCCTACTCCTCCTATCACTACCAGTACTAGCAGGAGCAATCACAATACTATTAACTGACCGAAACCTAAACACATCCTTCTTTGATCCAGCAGGAGGTGGAGACCC

AATCCTATACCAACACTTATTCTGATTCTTCGGACACCCAGAAGTATATATTTTAATCCTACCAGGATTTGGTATAATTTCCCACATTATTTGTCACGAAAGAGGTAAAAAGGAA

GCCTTCGGAAACCTAGGAATAATTTTCGCCATATTAGCAATCGGATTACTAGGATTTGTAGTATGAGCACACCATATGTTCACAGTAGGAATAGACGTTGACACACGAGCTTAC

TTTACATCAGCAACAATAATCATTGCAGTACCTACGGGGATTAAAATCTTCAGATGACTTGCAACAATATACGGAACCCGAATAACTTATAGAGCAGCATGCCTATGAGCCCTA

GGATTTGTA--

>G1_Macrotermes_subhyalinus_52 Macrotermes subhyalinus isolate TR172 cytochrome oxidase subunit I (COI) gene, partial cds; mitochondrial

----

AACAGAACTTGGACAACCAGGATCCTTAATCGGGGACGACCAAATCTACAACGTCATCGTCACAGCTCACGCTTTCGTAATGATCTTCTTCATAGTAATACCAATTATGATTGG

AGGATTCGGAAACTGACTAGTACCACTAATATTAGGAGCACCAGACATAGCATTCCCACGAATAAACAACATAAGATTCTGATTATTACCACCATCATTAACTCTTCTTCTCACT

AGTAGAACAGTAGAAAGTGGTGCAGGAACAGGATGAACAGTATACCCACCCCTTGCAAGAGGAATTGCCCACGCCGGAGCATCAGTAGATCTAGCCATCTTCTCATTACACTT

AGCAGGAGTATCATCCATCCTAGGAGCAGTAAACTTCATCTCAACAACAATCAACATGAAACCAAAAAACATAAAACCCGAACGAATCCCCTTATTCGTATGATCAGTTGCCAT

CACGGCTCTCCTACTCCTCCTATCACTACCAGTACTAGCAGGAGCAATCACAATACTATTAACTGACCGAAACCTAAACACATCCTTCTTTGATCCAGCAGGAGGTGGAGACCC

AATCCTATACCAACACTTATTCTGATTCTTCGGACACCCAGAAGTATATATTTTAATCCTACCAGGATTTGGTATAATTTCCCACATTATTTGTCACGAAAGAGGTAAAAAGGAA

GCCTTCGGAAACCTAGGAATAATTTTCGCCATATTAGCAATCGGATTACTAGGATTTGTAGTATGAGCACACCATATGTTCACAGTAGGAATAGACGTTGACACACGAGCTTAC

TTTACATCAGCAACAATAATCATTGCAGTACCTACGGGGATTAAAATCTTCAGATGACTTGCAACAATATACGGAACCCGAATAACTTATAGAGCAGCATGCCTATGAGCCCTA

GGATTTGTA--

>G1_Macrotermes_subhyalinus_53 Macrotermes subhyalinus isolate TM02 cytochrome oxidase subunit I (COI) gene, partial cds; mitochondrial

----

AACAGAACTTGGACAACCAGGATCCTTAATCGGGGACGACCAAATCTACAACGTCATCGTCACAGCTCACGCTTTCGTAATGATCTTCTTCATAGTAATACCAATTATGATTGG

AGGATTCGGAAACTGACTAGTACCACTAATATTAGGAGCACCAGACATAGCATTCCCACGAATAAACAACATAAGATTCTGATTATTACCACCATCATTAACTCTTCTTCTCACT

AGTAGAACAGTAGAAAGTGGTGCAGGAACAGGATGAACAGTATACCCACCCCTTGCAAGAGGAATTGCCCACGCCGGAGCATCAGTAGATCTAGCCATCTTCTCATTACACTT

AGCAGGAGTATCATCCATCCTAGGAGCAGTAAACTTCATCTCAACAACAATCAACATGAAACCAAAAAACATAAAACCCGAACGAATCCCCTTATTCGTATGATCAGTTGCCAT

CACGGCTCTCCTACTCCTCCTATCACTACCAGTACTAGCAGGAGCAATCACAATACTATTAACTGACCGAAACCTAAACACATCCTTCTTTGATCCAGCAGGAGGTGGAGACCC

AATCCTATACCAACACTTATTCTGATTCTTCGGACACCCAGAAGTATATATTTTAATCCTACCAGGATTTGGTATAATTTCCCACATTATTTGTCACGAAAGAGGTAAAAAGGAA

GCCTTCGGAAACCTAGGAATAATTTTCGCCATATTAGCAATCGGATTACTAGGATTTGTAGTATGAGCACACCATATGTTCACAGTAGGAATAGACGTTGACACACGAGCTTAC

TTTACATCAGCAACAATAATCATTGCAGTACCTACGGGGATTAAAATCTTCAGATGACTTGCAACAATATACGGAACCCGAATAACTTATAGAGCAGCATGCCTATGAGCCCTA

GGATTTGTA--

>G1_Macrotermes_subhyalinus_54 Macrotermes subhyalinus isolate TM05 cytochrome oxidase subunit I (COI) gene, partial cds; mitochondrial

----

AACAGAACTTGGACAACCAGGATCCTTAATCGGGGACGACCAAATCTACAACGTCATCGTCACAGCTCACGCTTTCGTAATGATCTTCTTCATAGTAATACCAATTATGATTGG

AGGATTCGGAAACTGACTAGTACCACTAATATTAGGAGCACCAGACATAGCATTCCCACGAATAAACAACATAAGATTCTGATTATTACCACCATCATTAACTCTTCTTCTCACT

AGTAGAACAGTAGAAAGTGGTGCAGGAACAGGATGAACAGTATACCCACCCCTTGCAAGAGGAATTGCCCACGCCGGAGCATCAGTAGATCTAGCCATCTTCTCATTACACTT

AGCAGGAGTATCATCCATCCTAGGAGCAGTAAACTTCATCTCAACAACAATCAACATGAAACCAAAAAACATAAAACCCGAACGAATCCCCTTATTCGTATGATCAGTTGCCAT

CACGGCTCTCCTACTCCTCCTATCACTACCAGTACTAGCAGGAGCAATCACAATACTATTAACTGACCGAAACCTAAACACATCCTTCTTTGATCCAGCAGGAGGTGGAGACCC

AATCCTATACCAACACTTATTCTGATTCTTCGGACACCCAGAAGTATATATTTTAATCCTACCAGGATTTGGTATAATTTCCCACATTATTTGTCACGAAAGAGGTAAAAAGGAA

GCCTTCGGAAACCTAGGAATAATTTTCGCCATATTAGCAATCGGATTACTAGGATTTGTAGTATGAGCACACCATATGTTCACAGTAGGAATAGACGTTGACACACGAGCTTAC

TTTACATCAGCAACAATAATCATTGCAGTACCTACGGGGATTAAAATCTTCAGATGACTTGCAACAATATACGGAACCCGAATAACTTATAGAGCAGCATGCCTATGAGCCCTA

GGATTTGTA---

>G1_Macrotermes_subhyalinus_55 Macrotermes subhyalinus isolate TM07 cytochrome oxidase subunit I (COI) gene, partial cds; mitochondrial

----

AACAGAACTTGGACAACCAGGATCCTTAATCGGGGACGACCAAATCTACAACGTCATCGTCACAGCTCACGCTTTCGTAATGATCTTCTTCATAGTAATACCAATTATGATTGG

AGGATTCGGAAACTGACTAGTACCACTAATATTAGGAGCACCAGACATAGCATTCCCACGAATAAACAACATAAGATTCTGATTATTACCACCATCATTAACTCTTCTTCTCACT

AGTAGAACAGTAGAAAGTGGTGCAGGAACAGGATGAACAGTATACCCACCCCTTGCAAGAGGAATTGCCCACGCCGGAGCATCAGTAGATCTAGCCATCTTCTCATTACACTT

AGCAGGAGTATCATCCATCCTAGGAGCAGTAAACTTCATCTCAACAACAATCAACATGAAACCAAAAAACATAAAACCCGAACGAATCCCCTTATTCGTATGATCAGTTGCCAT

CACGGCTCTCCTACTCCTCCTATCACTACCAGTACTAGCAGGAGCAATCACAATACTATTAACTGACCGAAACCTAAACACATCCTTCTTTGATCCAGCAGGAGGTGGAGACCC

AATCCTATACCAACACTTATTCTGATTCTTCGGACACCCAGAAGTATATATTTTAATCCTACCAGGATTTGGTATAATTTCCCACATTATTTGTCACGAAAGAGGTAAAAAGGAA

GCCTTCGGAAACCTAGGAATAATTTTCGCCATATTAGCAATCGGATTACTAGGATTTGTAGTATGAGCACACCATATGTTCACAGTAGGAATAGACGTTGACACACGAGCTTAC

TTTACATCAGCAACAATAATCATTGCAGTACCTACGGGGATTAAAATCTTCAGATGACTTGCAACAATATACGGAACCCGAATAACTTATAGAGCAGCATGCCTATGAGCCCTA

GGATTTGTA--

>G1_Macrotermes_subhyalinus_56 Macrotermes subhyalinus isolate TM08 cytochrome oxidase subunit I (COI) gene, partial cds; mitochondrial

----

AACAGAACTTGGACAACCAGGATCCTTAATCGGGGACGACCAAATCTACAACGTCATCGTCACAGCTCACGCTTTCGTAATGATCTTCTTCATAGTAATACCAATTATGATTGG

AGGATTCGGAAACTGACTAGTACCACTAATATTAGGAGCACCAGACATAGCATTCCCACGAATAAACAACATAAGATTCTGATTATTACCACCATCATTAACTCTTCTTCTCACT

AGTAGAACAGTAGAAAGTGGTGCAGGAACAGGATGAACAGTATACCCACCCCTTGCAAGAGGAATTGCCCACGCCGGAGCATCAGTAGATCTAGCCATCTTCTCATTACACTT

AGCAGGAGTATCATCCATCCTAGGAGCAGTAAACTTCATCTCAACAACAATCAACATGAAACCAAAAAACATAAAACCCGAACGAATCCCCTTATTCGTATGATCAGTTGCCAT

CACGGCTCTCCTACTCCTCCTATCACTACCAGTACTAGCAGGAGCAATCACAATACTATTAACTGACCGAAACCTAAACACATCCTTCTTTGATCCAGCAGGAGGTGGAGACCC

AATCCTATACCAACACTTATTCTGATTCTTCGGACACCCAGAAGTATATATTTTAATCCTACCAGGATTTGGTATAATTTCCCACATTATTTGTCACGAAAGAGGTAAAAAGGAA

GCCTTCGGAAACCTAGGAATAATTTTCGCCATATTAGCAATCGGATTACTAGGATTTGTAGTATGAGCACACCATATGTTCACAGTAGGAATAGACGTTGACACACGAGCTTAC

TTTACATCAGCAACAATAATCATTGCAGTACCTACGGGGATTAAAATCTTCAGATGACTTGCAACAATATACGGAACCCGAATAACTTATAGAGCAGCATGCCTATGAGCCCTA

GGATTTGTA--

>G1_Macrotermes_subhyalinus_57 Macrotermes subhyalinus isolate TM15 cytochrome oxidase subunit I (COI) gene, partial cds; mitochondrial

----

AACAGAACTTGGACAACCAGGATCCTTAATCGGGGACGACCAAATCTACAACGTCATCGTCACAGCTCACGCTTTCGTAATGATCTTCTTCATAGTAATACCAATTATGATTGG

AGGATTCGGAAACTGACTAGTACCACTAATATTAGGAGCACCAGACATAGCATTCCCACGAATAAACAACATAAGATTCTGATTATTACCACCATCATTAACTCTTCTTCTCACT

AGTAGAACAGTAGAAAGTGGTGCAGGAACAGGATGAACAGTATACCCACCCCTTGCAAGAGGAATTGCCCACGCCGGAGCATCAGTAGATCTAGCCATCTTCTCATTACACTT

AGCAGGAGTATCATCCATCCTAGGAGCAGTAAACTTCATCTCAACAACAATCAACATGAAACCAAAAAACATAAAACCCGAACGAATCCCCTTATTCGTATGATCAGTTGCCAT

CACGGCTCTCCTACTCCTCCTATCACTACCAGTACTAGCAGGAGCAATCACAATACTATTAACTGACCGAAACCTAAACACATCCTTCTTTGATCCAGCAGGAGGTGGAGACCC

AATCCTATACCAACACTTATTCTGATTCTTCGGACACCCAGAAGTATATATTTTAATCCTACCAGGATTTGGTATAATTTCCCACATTATTTGTCACGAAAGAGGTAAAAAGGAA

GCCTTCGGAAACCTAGGAATAATTTTCGCCATATTAGCAATCGGATTACTAGGATTTGTAGTATGAGCACACCATATGTTCACAGTAGGAATAGACGTTGACACACGAGCTTAC

TTTACATCAGCAACAATAATCATTGCAGTACCTACGGGGATTAAAATCTTCAGATGACTTGCAACAATATACGGAACCCGAATAACTTATAGAGCAGCATGCCTATGAGCCCTA

GGATTTGTA---

>G1_Macrotermes_subhyalinus_58 Macrotermes subhyalinus isolate TM17 cytochrome oxidase subunit I (COI) gene, partial cds; mitochondrial

----

AACAGAACTTGGACAACCAGGATCCTTAATCGGGGACGACCAAATCTACAACGTCATCGTCACAGCTCACGCTTTCGTAATGATCTTCTTCATAGTAATACCAATTATGATTGG

AGGATTCGGAAACTGACTAGTACCACTAATATTAGGAGCACCAGACATAGCATTCCCACGAATAAACAACATAAGATTCTGATTATTACCACCATCATTAACTCTTCTTCTCACT

AGTAGAACAGTAGAAAGTGGTGCAGGAACAGGATGAACAGTATACCCACCCCTTGCAAGAGGAATTGCCCACGCCGGAGCATCAGTAGATCTAGCCATCTTCTCATTACACTT

AGCAGGAGTATCATCCATCCTAGGAGCAGTAAACTTCATCTCAACAACAATCAACATGAAACCAAAAAACATAAAACCCGAACGAATCCCCTTATTCGTATGATCAGTTGCCAT

CACGGCTCTCCTACTCCTCCTATCACTACCAGTACTAGCAGGAGCAATCACAATACTATTAACTGACCGAAACCTAAACACATCCTTCTTTGATCCAGCAGGAGGTGGAGACCC

AATCCTATACCAACACTTATTCTGATTCTTCGGACACCCAGAAGTATATATTTTAATCCTACCAGGATTTGGTATAATTTCCCACATTATTTGTCACGAAAGAGGTAAAAAGGAA

GCCTTCGGAAACCTAGGAATAATTTTCGCCATATTAGCAATCGGATTACTAGGATTTGTAGTATGAGCACACCATATGTTCACAGTAGGAATAGACGTTGACACACGAGCTTAC

TTTACATCAGCAACAATAATCATTGCAGTACCTACGGGGATTAAAATCTTCAGATGACTTGCAACAATATACGGAACCCGAATAACTTATAGAGCAGCATGCCTATGAGCCCTA
GGATTTGTA---

>G1_Macrotermes_subhyalinus_59 Macrotermes subhyalinus isolate TM22 cytochrome oxidase subunit I (COI) gene, partial cds; mitochondrial

----
AACAGAACTTGGACAACCAGGATCCTTAATCGGGGACGACCAAATCTACAACGTCATCGTCACAGCTCACGCTTTCGTAATGATCTTCTTCATAGTAATACCAATTATGATTGG
AGGATTCGGAAACTGACTAGTACCACTAATATTAGGAGCACCAGACATAGCATTCCCACGAATAAACAACATAAGATTCTGATTATTACCACCATCATTAACTCTTCTTCTCACT
AGTAGAACAGTAGAAAGTGGTGCAGGAACAGGATGAACAGTATACCCACCCCTTGCAAGAGGAATTGCCCACGCCGGAGCATCAGTAGATCTAGCCATCTTCTCATTACACTT
AGCAGGAGTATCATCCATCCTAGGAGCAGTAAACTTCATCTCAACAACAATCAACATGAAACCAAAAAACATAAAACCCGAACGAATCCCCTTATTCGTATGATCAGTTGCCAT
CACGGCTCTCCTACTCCTCCTATCACTACCAGTACTAGCAGGAGCAATCACAATACTATTAACTGACCGAAACCTAAACACATCCTTCTTTGATCCAGCAGGAGGTGGAGACCC
AATCCTATACCAACACTTATTCTGATTCTTCGGACACCCAGAAGTATATATTTTAATCCTACCAGGATTTGGTATAATTTCCCACATTATTTGTCACGAAAGAGGTAAAAAGGAA
GCCTTCGGAAACCTAGGAATAATTTTCGCCATATTAGCAATCGGATTACTAGGATTTGTAGTATGAGCACACCATATGTTCACAGTAGGAATAGACGTTGACACACGAGCTTAC
TTTACATCAGCAACAATAATCATTGCAGTACCTACGGGGATTAAAATCTTCAGATGACTTGCAACAATATACGGAACCCGAATAACTTATAGAGCAGCATGCCTATGAGCCCTA
GGATTTGTA---

>G1_Macrotermes_subhyalinus_60 Macrotermes subhyalinus isolate TM23 cytochrome oxidase subunit I (COI) gene, partial cds; mitochondrial

----

AACAGAACTTGGACAACCAGGATCCTTAATCGGGGACGACCAAATCTACAACGTCATCGTCACAGCTCACGCTTTCGTAATGATCTTCTTCATAGTAATACCAATTATGATTGG

AGGATTCGGAAACTGACTAGTACCACTAATATTAGGAGCACCAGACATAGCATTCCCACGAATAAACAACATAAGATTCTGATTATTACCACCATCATTAACTCTTCTTCTCACT

AGTAGAACAGTAGAAAGTGGTGCAGGAACAGGATGAACAGTATACCCACCCCTTGCAAGAGGAATTGCCCACGCCGGAGCATCAGTAGATCTAGCCATCTTCTCATTACACTT

AGCAGGAGTATCATCCATCCTAGGAGCAGTAAACTTCATCTCAACAACAATCAACATGAAACCAAAAAACATAAAACCCGAACGAATCCCCTTATTCGTATGATCAGTTGCCAT

CACGGCTCTCCTACTCCTCCTATCACTACCAGTACTAGCAGGAGCAATCACAATACTATTAACTGACCGAAACCTAAACACATCCTTCTTTGATCCAGCAGGAGGTGGAGACCC

AATCCTATACCAACACTTATTCTGATTCTTCGGACACCCAGAAGTATATATTTTAATCCTACCAGGATTTGGTATAATTTCCCACATTATTTGTCACGAAAGAGGTAAAAAGGAA

GCCTTCGGAAACCTAGGAATAATTTTCGCCATATTAGCAATCGGATTACTAGGATTTGTAGTATGAGCACACCATATGTTCACAGTAGGAATAGACGTTGACACACGAGCTTAC

TTTACATCAGCAACAATAATCATTGCAGTACCTACGGGGATTAAAATCTTCAGATGACTTGCAACAATATACGGAACCCGAATAACTTATAGAGCAGCATGCCTATGAGCCCTA

GGATTTGTA--

>G1_Macrotermes_subhyalinus_61 Macrotermes subhyalinus isolate TM25 cytochrome oxidase subunit I (COI) gene, partial cds; mitochondrial

----

AACAGAACTTGGACAACCAGGATCCTTAATCGGGGACGACCAAATCTACAACGTCATCGTCACAGCTCACGCTTTCGTAATGATCTTCTTCATAGTAATACCAATTATGATTGG

AGGATTCGGAAACTGACTAGTACCACTAATATTAGGAGCACCAGACATAGCATTCCCACGAATAAACAACATAAGATTCTGATTATTACCACCATCATTAACTCTTCTTCTCACT

AGTAGAACAGTAGAAAGTGGTGCAGGAACAGGATGAACAGTATACCCACCCCTTGCAAGAGGAATTGCCCACGCCGGAGCATCAGTAGATCTAGCCATCTTCTCATTACACTT

AGCAGGAGTATCATCCATCCTAGGAGCAGTAAACTTCATCTCAACAACAATCAACATGAAACCAAAAAACATAAAACCCGAACGAATCCCCTTATTCGTATGATCAGTTGCCAT

CACGGCTCTCCTACTCCTCCTATCACTACCAGTACTAGCAGGAGCAATCACAATACTATTAACTGACCGAAACCTAAACACATCCTTCTTTGATCCAGCAGGAGGTGGAGACCC

AATCCTATACCAACACTTATTCTGATTCTTCGGACACCCAGAAGTATATATTTTAATCCTACCAGGATTTGGTATAATTTCCCACATTATTTGTCACGAAAGAGGTAAAAAGGAA

GCCTTCGGAAACCTAGGAATAATTTTCGCCATATTAGCAATCGGATTACTAGGATTTGTAGTATGAGCACACCATATGTTCACAGTAGGAATAGACGTTGACACACGAGCTTAC

TTTACATCAGCAACAATAATCATTGCAGTACCTACGGGGATTAAAATCTTCAGATGACTTGCAACAATATACGGAACCCGAATAACTTATAGAGCAGCATGCCTATGAGCCCTA
GGATTTGTA---

>G1_Macrotermes_subhyalinus_62 Macrotermes subhyalinus isolate TM26 cytochrome oxidase subunit I (COI) gene, partial cds; mitochondrial

----
AACAGAACTTGGACAACCAGGATCCTTAATCGGGGACGACCAAATCTACAACGTCATCGTCACAGCTCACGCTTTCGTAATGATCTTCTTCATAGTAATACCAATTATGATTGG
AGGATTCGGAAACTGACTAGTACCACTAATATTAGGAGCACCAGACATAGCATTCCCACGAATAAACAACATAAGATTCTGATTATTACCACCATCATTAACTCTTCTTCTCACT
AGTAGAACAGTAGAAAGTGGTGCAGGAACAGGATGAACAGTATACCCACCCCTTGCAAGAGGAATTGCCCACGCCGGAGCATCAGTAGATCTAGCCATCTTCTCATTACACTT
AGCAGGAGTATCATCCATCCTAGGAGCAGTAAACTTCATCTCAACAACAATCAACATGAAACCAAAAAACATAAAACCCGAACGAATCCCCTTATTCGTATGATCAGTTGCCAT
CACGGCTCTCCTACTCCTCCTATCACTACCAGTACTAGCAGGAGCAATCACAATACTATTAACTGACCGAAACCTAAACACATCCTTCTTTGATCCAGCAGGAGGTGGAGACCC
AATCCTATACCAACACTTATTCTGATTCTTCGGACACCCAGAAGTATATATTTTAATCCTACCAGGATTTGGTATAATTTCCCACATTATTTGTCACGAAAGAGGTAAAAAGGAA
GCCTTCGGAAACCTAGGAATAATTTTCGCCATATTAGCAATCGGATTACTAGGATTTGTAGTATGAGCACACCATATGTTCACAGTAGGAATAGACGTTGACACACGAGCTTAC
TTTACATCAGCAACAATAATCATTGCAGTACCTACGGGGATTAAAATCTTCAGATGACTTGCAACAATATACGGAACCCGAATAACTTATAGAGCAGCATGCCTATGAGCCCTA
GGATTTGTA---

>G1_Macrotermes_subhyalinus_63 Macrotermes subhyalinus isolate TM28 cytochrome oxidase subunit I (COI) gene, partial cds; mitochondrial

----

AACAGAACTTGGACAACCAGGATCCTTAATCGGGGACGACCAAATCTACAACGTCATCGTCACAGCTCACGCTTTCGTAATGATCTTCTTCATAGTAATACCAATTATGATTGG

AGGATTCGGAAACTGACTAGTACCACTAATATTAGGAGCACCAGACATAGCATTCCCACGAATAAACAACATAAGATTCTGATTATTACCACCATCATTAACTCTTCTTCTCACT

AGTAGAACAGTAGAAAGTGGTGCAGGAACAGGATGAACAGTATACCCACCCCTTGCAAGAGGAATTGCCCACGCCGGAGCATCAGTAGATCTAGCCATCTTCTCATTACACTT

AGCAGGAGTATCATCCATCCTAGGAGCAGTAAACTTCATCTCAACAACAATCAACATGAAACCAAAAAACATAAAACCCGAACGAATCCCCTTATTCGTATGATCAGTTGCCAT

CACGGCTCTCCTACTCCTCCTATCACTACCAGTACTAGCAGGAGCAATCACAATACTATTAACTGACCGAAACCTAAACACATCCTTCTTTGATCCAGCAGGAGGTGGAGACCC

AATCCTATACCAACACTTATTCTGATTCTTCGGACACCCAGAAGTATATATTTTAATCCTACCAGGATTTGGTATAATTTCCCACATTATTTGTCACGAAAGAGGTAAAAAGGAA

GCCTTCGGAAACCTAGGAATAATTTTCGCCATATTAGCAATCGGATTACTAGGATTTGTAGTATGAGCACACCATATGTTCACAGTAGGAATAGACGTTGACACACGAGCTTAC

TTTACATCAGCAACAATAATCATTGCAGTACCTACGGGGATTAAAATCTTCAGATGACTTGCAACAATATACGGAACCCGAATAACTTATAGAGCAGCATGCCTATGAGCCCTA

GGATTTGTA--

>G1_Macrotermes_subhyalinus_64 Macrotermes subhyalinus isolate TM33 cytochrome oxidase subunit I (COI) gene, partial cds; mitochondrial

----

AACAGAACTTGGACAACCAGGATCCTTAATCGGGGACGACCAAATCTACAACGTCATCGTCACAGCTCACGCTTTCGTAATGATCTTCTTCATAGTAATACCAATTATGATTGG

AGGATTCGGAAACTGACTAGTACCACTAATATTAGGAGCACCAGACATAGCATTCCCACGAATAAACAACATAAGATTCTGATTATTACCACCATCATTAACTCTTCTTCTCACT

AGTAGAACAGTAGAAAGTGGTGCAGGAACAGGATGAACAGTATACCCACCCCTTGCAAGAGGAATTGCCCACGCCGGAGCATCAGTAGATCTAGCCATCTTCTCATTACACTT

AGCAGGAGTATCATCCATCCTAGGAGCAGTAAACTTCATCTCAACAACAATCAACATGAAACCAAAAAACATAAAACCCGAACGAATCCCCTTATTCGTATGATCAGTTGCCAT

CACGGCTCTCCTACTCCTCCTATCACTACCAGTACTAGCAGGAGCAATCACAATACTATTAACTGACCGAAACCTAAACACATCCTTCTTTGATCCAGCAGGAGGTGGAGACCC

AATCCTATACCAACACTTATTCTGATTCTTCGGACACCCAGAAGTATATATTTTAATCCTACCAGGATTTGGTATAATTTCCCACATTATTTGTCACGAAAGAGGTAAAAAGGAA

GCCTTCGGAAACCTAGGAATAATTTTCGCCATATTAGCAATCGGATTACTAGGATTTGTAGTATGAGCACACCATATGTTCACAGTAGGAATAGACGTTGACACACGAGCTTAC

TTTACATCAGCAACAATAATCATTGCAGTACCTACGGGGATTAAAATCTTCAGATGACTTGCAACAATATACGGAACCCGAATAACTTATAGAGCAGCATGCCTATGAGCCCTA

GGATTTGTA--

>G1_Macrotermes_subhyalinus_65 Macrotermes subhyalinus isolate TM34 cytochrome oxidase subunit I (COI) gene, partial cds; mitochondrial

----

AACAGAACTTGGACAACCAGGATCCTTAATCGGGGACGACCAAATCTACAACGTCATCGTCACAGCTCACGCTTTCGTAATGATCTTCTTCATAGTAATACCAATTATGATTGG

AGGATTCGGAAACTGACTAGTACCACTAATATTAGGAGCACCAGACATAGCATTCCCACGAATAAACAACATAAGATTCTGATTATTACCACCATCATTAACTCTTCTTCTCACT

AGTAGAACAGTAGAAAGTGGTGCAGGAACAGGATGAACAGTATACCCACCCCTTGCAAGAGGAATTGCCCACGCCGGAGCATCAGTAGATCTAGCCATCTTCTCATTACACTT

AGCAGGAGTATCATCCATCCTAGGAGCAGTAAACTTCATCTCAACAACAATCAACATGAAACCAAAAAACATAAAACCCGAACGAATCCCCTTATTCGTATGATCAGTTGCCAT

CACGGCTCTCCTACTCCTCCTATCACTACCAGTACTAGCAGGAGCAATCACAATACTATTAACTGACCGAAACCTAAACACATCCTTCTTTGATCCAGCAGGAGGTGGAGACCC

AATCCTATACCAACACTTATTCTGATTCTTCGGACACCCAGAAGTATATATTTTAATCCTACCAGGATTTGGTATAATTTCCCACATTATTTGTCACGAAAGAGGTAAAAAGGAA

GCCTTCGGAAACCTAGGAATAATTTTCGCCATATTAGCAATCGGATTACTAGGATTTGTAGTATGAGCACACCATATGTTCACAGTAGGAATAGACGTTGACACACGAGCTTAC

TTTACATCAGCAACAATAATCATTGCAGTACCTACGGGGATTAAAATCTTCAGATGACTTGCAACAATATACGGAACCCGAATAACTTATAGAGCAGCATGCCTATGAGCCCTA

GGATTTGTA--

>G1_Macrotermes_subhyalinus_66 Macrotermes subhyalinus isolate TM35 cytochrome oxidase subunit I (COI) gene, partial cds; mitochondrial

----

AACAGAACTTGGACAACCAGGATCCTTAATCGGGGACGACCAAATCTACAACGTCATCGTCACAGCTCACGCTTTCGTAATGATCTTCTTCATAGTAATACCAATTATGATTGG

AGGATTCGGAAACTGACTAGTACCACTAATATTAGGAGCACCAGACATAGCATTCCCACGAATAAACAACATAAGATTCTGATTATTACCACCATCATTAACTCTTCTTCTCACT

AGTAGAACAGTAGAAAGTGGTGCAGGAACAGGATGAACAGTATACCCACCCCTTGCAAGAGGAATTGCCCACGCCGGAGCATCAGTAGATCTAGCCATCTTCTCATTACACTT

AGCAGGAGTATCATCCATCCTAGGAGCAGTAAACTTCATCTCAACAACAATCAACATGAAACCAAAAAACATAAAACCCGAACGAATCCCCTTATTCGTATGATCAGTTGCCAT

CACGGCTCTCCTACTCCTCCTATCACTACCAGTACTAGCAGGAGCAATCACAATACTATTAACTGACCGAAACCTAAACACATCCTTCTTTGATCCAGCAGGAGGTGGAGACCC

AATCCTATACCAACACTTATTCTGATTCTTCGGACACCCAGAAGTATATATTTTAATCCTACCAGGATTTGGTATAATTTCCCACATTATTTGTCACGAAAGAGGTAAAAAGGAA

GCCTTCGGAAACCTAGGAATAATTTTCGCCATATTAGCAATCGGATTACTAGGATTTGTAGTATGAGCACACCATATGTTCACAGTAGGAATAGACGTTGACACACGAGCTTAC

TTTACATCAGCAACAATAATCATTGCAGTACCTACGGGGATTAAAATCTTCAGATGACTTGCAACAATATACGGAACCCGAATAACTTATAGAGCAGCATGCCTATGAGCCCTA

GGATTTGTA---

>G1_Macrotermes_subhyalinus_67 Macrotermes subhyalinus isolate TM36 cytochrome oxidase subunit I (COI) gene, partial cds; mitochondrial

----

AACAGAACTTGGACAACCAGGATCCTTAATCGGGGACGACCAAATCTACAACGTCATCGTCACAGCTCACGCTTTCGTAATGATCTTCTTCATAGTAATACCAATTATGATTGG

AGGATTCGGAAACTGACTAGTACCACTAATATTAGGAGCACCAGACATAGCATTCCCACGAATAAACAACATAAGATTCTGATTATTACCACCATCATTAACTCTTCTTCTCACT

AGTAGAACAGTAGAAAGTGGTGCAGGAACAGGATGAACAGTATACCCACCCCTTGCAAGAGGAATTGCCCACGCCGGAGCATCAGTAGATCTAGCCATCTTCTCATTACACTT

AGCAGGAGTATCATCCATCCTAGGAGCAGTAAACTTCATCTCAACAACAATCAACATGAAACCAAAAAACATAAAACCCGAACGAATCCCCTTATTCGTATGATCAGTTGCCAT

CACGGCTCTCCTACTCCTCCTATCACTACCAGTACTAGCAGGAGCAATCACAATACTATTAACTGACCGAAACCTAAACACATCCTTCTTTGATCCAGCAGGAGGTGGAGACCC

AATCCTATACCAACACTTATTCTGATTCTTCGGACACCCAGAAGTATATATTTTAATCCTACCAGGATTTGGTATAATTTCCCACATTATTTGTCACGAAAGAGGTAAAAAGGAA

GCCTTCGGAAACCTAGGAATAATTTTCGCCATATTAGCAATCGGATTACTAGGATTTGTAGTATGAGCACACCATATGTTCACAGTAGGAATAGACGTTGACACACGAGCTTAC

TTTACATCAGCAACAATAATCATTGCAGTACCTACGGGGATTAAAATCTTCAGATGACTTGCAACAATATACGGAACCCGAATAACTTATAGAGCAGCATGCCTATGAGCCCTA
GGATTTGTA--

>G1_Macrotermes_subhyalinus_68 Macrotermes subhyalinus isolate TM37 cytochrome oxidase subunit I (COI) gene, partial cds; mitochondrial

----
AACAGAACTTGGACAACCAGGATCCTTAATCGGGGACGACCAAATCTACAACGTCATCGTCACAGCTCACGCTTTCGTAATGATCTTCTTCATAGTAATACCAATTATGATTGG
AGGATTCGGAAACTGACTAGTACCACTAATATTAGGAGCACCAGACATAGCATTCCCACGAATAAACAACATAAGATTCTGATTATTACCACCATCATTAACTCTTCTTCTCACT
AGTAGAACAGTAGAAAGTGGTGCAGGAACAGGATGAACAGTATACCCACCCCTTGCAAGAGGAATTGCCCACGCCGGAGCATCAGTAGATCTAGCCATCTTCTCATTACACTT
AGCAGGAGTATCATCCATCCTAGGAGCAGTAAACTTCATCTCAACAACAATCAACATGAAACCAAAAAACATAAAACCCGAACGAATCCCCTTATTCGTATGATCAGTTGCCAT
CACGGCTCTCCTACTCCTCCTATCACTACCAGTACTAGCAGGAGCAATCACAATACTATTAACTGACCGAAACCTAAACACATCCTTCTTTGATCCAGCAGGAGGTGGAGACCC
AATCCTATACCAACACTTATTCTGATTCTTCGGACACCCAGAAGTATATATTTTAATCCTACCAGGATTTGGTATAATTTCCCACATTATTTGTCACGAAAGAGGTAAAAAGGAA
GCCTTCGGAAACCTAGGAATAATTTTCGCCATATTAGCAATCGGATTACTAGGATTTGTAGTATGAGCACACCATATGTTCACAGTAGGAATAGACGTTGACACACGAGCTTAC
TTTACATCAGCAACAATAATCATTGCAGTACCTACGGGGATTAAAATCTTCAGATGACTTGCAACAATATACGGAACCCGAATAACTTATAGAGCAGCATGCCTATGAGCCCTA
GGATTTGTA--

>G1_Macrotermes_subhyalinus_69 Macrotermes subhyalinus isolate TM38 cytochrome oxidase subunit I (COI) gene, partial cds; mitochondrial

----

AACAGAACTTGGACAACCAGGATCCTTAATCGGGGACGACCAAATCTACAACGTCATCGTCACAGCTCACGCTTTCGTAATGATCTTCTTCATAGTAATACCAATTATGATTGG

AGGATTCGGAAACTGACTAGTACCACTAATATTAGGAGCACCAGACATAGCATTCCCACGAATAAACAACATAAGATTCTGATTATTACCACCATCATTAACTCTTCTTCTCACT

AGTAGAACAGTAGAAAGTGGTGCAGGAACAGGATGAACAGTATACCCACCCCTTGCAAGAGGAATTGCCCACGCCGGAGCATCAGTAGATCTAGCCATCTTCTCATTACACTT

AGCAGGAGTATCATCCATCCTAGGAGCAGTAAACTTCATCTCAACAACAATCAACATGAAACCAAAAAACATAAAACCCGAACGAATCCCCTTATTCGTATGATCAGTTGCCAT

CACGGCTCTCCTACTCCTCCTATCACTACCAGTACTAGCAGGAGCAATCACAATACTATTAACTGACCGAAACCTAAACACATCCTTCTTTGATCCAGCAGGAGGTGGAGACCC

AATCCTATACCAACACTTATTCTGATTCTTCGGACACCCAGAAGTATATATTTTAATCCTACCAGGATTTGGTATAATTTCCCACATTATTTGTCACGAAAGAGGTAAAAAGGAA

GCCTTCGGAAACCTAGGAATAATTTTCGCCATATTAGCAATCGGATTACTAGGATTTGTAGTATGAGCACACCATATGTTCACAGTAGGAATAGACGTTGACACACGAGCTTAC

TTTACATCAGCAACAATAATCATTGCAGTACCTACGGGGATTAAAATCTTCAGATGACTTGCAACAATATACGGAACCCGAATAACTTATAGAGCAGCATGCCTATGAGCCCTA

GGATTTGTA--

>G1_Macrotermes_subhyalinus_70 Macrotermes subhyalinus isolate TM41 cytochrome oxidase subunit I (COI) gene, partial cds; mitochondrial

----

AACAGAACTTGGACAACCAGGATCCTTAATCGGGGACGACCAAATCTACAACGTCATCGTCACAGCTCACGCTTTCGTAATGATCTTCTTCATAGTAATACCAATTATGATTGG

AGGATTCGGAAACTGACTAGTACCACTAATATTAGGAGCACCAGACATAGCATTCCCACGAATAAACAACATAAGATTCTGATTATTACCACCATCATTAACTCTTCTTCTCACT

AGTAGAACAGTAGAAAGTGGTGCAGGAACAGGATGAACAGTATACCCACCCCTTGCAAGAGGAATTGCCCACGCCGGAGCATCAGTAGATCTAGCCATCTTCTCATTACACTT

AGCAGGAGTATCATCCATCCTAGGAGCAGTAAACTTCATCTCAACAACAATCAACATGAAACCAAAAAACATAAAACCCGAACGAATCCCCTTATTCGTATGATCAGTTGCCAT

CACGGCTCTCCTACTCCTCCTATCACTACCAGTACTAGCAGGAGCAATCACAATACTATTAACTGACCGAAACCTAAACACATCCTTCTTTGATCCAGCAGGAGGTGGAGACCC

AATCCTATACCAACACTTATTCTGATTCTTCGGACACCCAGAAGTATATATTTTAATCCTACCAGGATTTGGTATAATTTCCCACATTATTTGTCACGAAAGAGGTAAAAAGGAA

GCCTTCGGAAACCTAGGAATAATTTTCGCCATATTAGCAATCGGATTACTAGGATTTGTAGTATGAGCACACCATATGTTCACAGTAGGAATAGACGTTGACACACGAGCTTAC

TTTACATCAGCAACAATAATCATTGCAGTACCTACGGGGATTAAAATCTTCAGATGACTTGCAACAATATACGGAACCCGAATAACTTATAGAGCAGCATGCCTATGAGCCCTA
GGATTTGTA--

>G1_Macrotermes_subhyalinus_71 Macrotermes subhyalinus isolate TM42 cytochrome oxidase subunit I (COI) gene, partial cds; mitochondrial

----
AACAGAACTTGGACAACCAGGATCCTTAATCGGGGACGACCAAATCTACAACGTCATCGTCACAGCTCACGCTTTCGTAATGATCTTCTTCATAGTAATACCAATTATGATTGG
AGGATTCGGAAACTGACTAGTACCACTAATATTAGGAGCACCAGACATAGCATTCCCACGAATAAACAACATAAGATTCTGATTATTACCACCATCATTAACTCTTCTTCTCACT
AGTAGAACAGTAGAAAGTGGTGCAGGAACAGGATGAACAGTATACCCACCCCTTGCAAGAGGAATTGCCCACGCCGGAGCATCAGTAGATCTAGCCATCTTCTCATTACACTT
AGCAGGAGTATCATCCATCCTAGGAGCAGTAAACTTCATCTCAACAACAATCAACATGAAACCAAAAAACATAAAACCCGAACGAATCCCCTTATTCGTATGATCAGTTGCCAT
CACGGCTCTCCTACTCCTCCTATCACTACCAGTACTAGCAGGAGCAATCACAATACTATTAACTGACCGAAACCTAAACACATCCTTCTTTGATCCAGCAGGAGGTGGAGACCC
AATCCTATACCAACACTTATTCTGATTCTTCGGACACCCAGAAGTATATATTTTAATCCTACCAGGATTTGGTATAATTTCCCACATTATTTGTCACGAAAGAGGTAAAAAGGAA
GCCTTCGGAAACCTAGGAATAATTTTCGCCATATTAGCAATCGGATTACTAGGATTTGTAGTATGAGCACACCATATGTTCACAGTAGGAATAGACGTTGACACACGAGCTTAC
TTTACATCAGCAACAATAATCATTGCAGTACCTACGGGGATTAAAATCTTCAGATGACTTGCAACAATATACGGAACCCGAATAACTTATAGAGCAGCATGCCTATGAGCCCTA
GGATTTGTA--

>G1_Macrotermes_subhyalinus_72 Macrotermes subhyalinus isolate TB02 cytochrome oxidase subunit I (COI) gene, partial cds; mitochondrial

----

AACAGAACTTGGACAACCAGGATCCTTAATCGGGGACGACCAAATCTACAACGTCATCGTCACAGCTCACGCTTTCGTAATGATCTTCTTCATAGTAATACCAATTATGATTGG
AGGATTCGGAAACTGACTAGTACCACTAATATTAGGAGCACCAGACATAGCATTCCCACGAATAAACAACATAAGATTCTGATTATTACCACCATCATTAACTCTTCTTCTCACT
AGTAGAACAGTAGAAAGTGGTGCAGGAACAGGATGAACAGTATACCCACCCCTTGCAAGAGGAATTGCCCACGCCGGAGCATCAGTAGATCTAGCCATCTTCTCATTACACTT
AGCAGGAGTATCATCCATCCTAGGAGCAGTAAACTTCATCTCAACAACAATCAACATGAAACCAAAAAACATAAAACCCGAACGAATCCCCTTATTCGTATGATCAGTTGCCAT
CACGGCTCTCCTACTCCTCCTATCACTACCAGTACTAGCAGGAGCAATCACAATACTATTAACTGACCGAAACCTAAACACATCCTTCTTTGATCCAGCAGGAGGTGGAGACCC
AATCCTATACCAACACTTATTCTGATTCTTCGGACACCCAGAAGTATATATTTTAATCCTACCAGGATTTGGTATAATTTCCCACATTATTTGTCACGAAAGAGGTAAAAAGGAA
GCCTTCGGAAACCTAGGAATAATTTTCGCCATATTAGCAATCGGATTACTAGGATTTGTAGTATGAGCACACCATATGTTCACAGTAGGAATAGACGTTGACACACGAGCTTAC
TTTACATCAGCAACAATAATCATTGCAGTACCTACGGGGATTAAAATCTTCAGATGACTTGCAACAATATACGGAACCCGAATAACTTATAGAGCAGCATGCCTATGAGCCCTA
GGATTTGTA---

>G1_Macrotermes_subhyalinus_73 Macrotermes subhyalinus isolate TB03 cytochrome oxidase subunit I (COI) gene, partial cds; mitochondrial

----

AACAGAACTTGGACAACCAGGATCCTTAATCGGGGACGACCAAATCTACAACGTCATCGTCACAGCTCACGCTTTCGTAATGATCTTCTTCATAGTAATACCAATTATGATTGG
AGGATTCGGAAACTGACTAGTACCACTAATATTAGGAGCACCAGACATAGCATTCCCACGAATAAACAACATAAGATTCTGATTATTACCACCATCATTAACTCTTCTTCTCACT
AGTAGAACAGTAGAAAGTGGTGCAGGAACAGGATGAACAGTATACCCACCCCTTGCAAGAGGAATTGCCCACGCCGGAGCATCAGTAGATCTAGCCATCTTCTCATTACACTT
AGCAGGAGTATCATCCATCCTAGGAGCAGTAAACTTCATCTCAACAACAATCAACATGAAACCAAAAAACATAAAACCCGAACGAATCCCCTTATTCGTATGATCAGTTGCCAT
CACGGCTCTCCTACTCCTCCTATCACTACCAGTACTAGCAGGAGCAATCACAATACTATTAACTGACCGAAACCTAAACACATCCTTCTTTGATCCAGCAGGAGGTGGAGACCC
AATCCTATACCAACACTTATTCTGATTCTTCGGACACCCAGAAGTATATATTTTAATCCTACCAGGATTTGGTATAATTTCCCACATTATTTGTCACGAAAGAGGTAAAAAGGAA
GCCTTCGGAAACCTAGGAATAATTTTCGCCATATTAGCAATCGGATTACTAGGATTTGTAGTATGAGCACACCATATGTTCACAGTAGGAATAGACGTTGACACACGAGCTTAC

TTTACATCAGCAACAATAATCATTGCAGTACCTACGGGGATTAAAATCTTCAGATGACTTGCAACAATATACGGAACCCGAATAACTTATAGAGCAGCATGCCTATGAGCCCTA

GGATTTGTA---

>G1_Macrotermes_subhyalinus_74 Macrotermes subhyalinus isolate TB04 cytochrome oxidase subunit I (COI) gene, partial cds; mitochondrial

----

AACAGAACTTGGACAACCAGGATCCTTAATCGGGGACGACCAAATCTACAACGTCATCGTCACAGCTCACGCTTTCGTAATGATCTTCTTCATAGTAATACCAATTATGATTGG

AGGATTCGGAAACTGACTAGTACCACTAATATTAGGAGCACCAGACATAGCATTCCCACGAATAAACAACATAAGATTCTGATTATTACCACCATCATTAACTCTTCTTCTCACT

AGTAGAACAGTAGAAAGTGGTGCAGGAACAGGATGAACAGTATACCCACCCCTTGCAAGAGGAATTGCCCACGCCGGAGCATCAGTAGATCTAGCCATCTTCTCATTACACTT

AGCAGGAGTATCATCCATCCTAGGAGCAGTAAACTTCATCTCAACAACAATCAACATGAAACCAAAAAACATAAAACCCGAACGAATCCCCTTATTCGTATGATCAGTTGCCAT

CACGGCTCTCCTACTCCTCCTATCACTACCAGTACTAGCAGGAGCAATCACAATACTATTAACTGACCGAAACCTAAACACATCCTTCTTTGATCCAGCAGGAGGTGGAGACCC

AATCCTATACCAACACTTATTCTGATTCTTCGGACACCCAGAAGTATATATTTTAATCCTACCAGGATTTGGTATAATTTCCCACATTATTTGTCACGAAAGAGGTAAAAAGGAA

GCCTTCGGAAACCTAGGAATAATTTTCGCCATATTAGCAATCGGATTACTAGGATTTGTAGTATGAGCACACCATATGTTCACAGTAGGAATAGACGTTGACACACGAGCTTAC

TTTACATCAGCAACAATAATCATTGCAGTACCTACGGGGATTAAAATCTTCAGATGACTTGCAACAATATACGGAACCCGAATAACTTATAGAGCAGCATGCCTATGAGCCCTA

GGATTTGTA---

>G1_Macrotermes_subhyalinus_75 Macrotermes subhyalinus isolate TB17 cytochrome oxidase subunit I (COI) gene, partial cds; mitochondrial

----

AACAGAACTTGGACAACCAGGATCCTTAATCGGGGACGACCAAATCTACAACGTCATCGTCACAGCTCACGCTTTCGTAATGATCTTCTTCATAGTAATACCAATTATGATTGG

AGGATTCGGAAACTGACTAGTACCACTAATATTAGGAGCACCAGACATAGCATTCCCACGAATAAACAACATAAGATTCTGATTATTACCACCATCATTAACTCTTCTTCTCACT

AGTAGAACAGTAGAAAGTGGTGCAGGAACAGGATGAACAGTATACCCACCCCTTGCAAGAGGAATTGCCCACGCCGGAGCATCAGTAGATCTAGCCATCTTCTCATTACACTT

AGCAGGAGTATCATCCATCCTAGGAGCAGTAAACTTCATCTCAACAACAATCAACATGAAACCAAAAAACATAAAACCCGAACGAATCCCCTTATTCGTATGATCAGTTGCCAT

CACGGCTCTCCTACTCCTCCTATCACTACCAGTACTAGCAGGAGCAATCACAATACTATTAACTGACCGAAACCTAAACACATCCTTCTTTGATCCAGCAGGAGGTGGAGACCC

AATCCTATACCAACACTTATTCTGATTCTTCGGACACCCAGAAGTATATATTTTAATCCTACCAGGATTTGGTATAATTTCCCACATTATTTGTCACGAAAGAGGTAAAAAGGAA

GCCTTCGGAAACCTAGGAATAATTTTCGCCATATTAGCAATCGGATTACTAGGATTTGTAGTATGAGCACACCATATGTTCACAGTAGGAATAGACGTTGACACACGAGCTTAC

TTTACATCAGCAACAATAATCATTGCAGTACCTACGGGGATTAAAATCTTCAGATGACTTGCAACAATATACGGAACCCGAATAACTTATAGAGCAGCATGCCTATGAGCCCTA

GGATTTGTA---

>G1_Macrotermes_subhyalinus_76 Macrotermes subhyalinus isolate TB19 cytochrome oxidase subunit I (COI) gene, partial cds; mitochondrial

----

AACAGAACTTGGACAACCAGGATCCTTAATCGGGGACGACCAAATCTACAACGTCATCGTCACAGCTCACGCTTTCGTAATGATCTTCTTCATAGTAATACCAATTATGATTGG

AGGATTCGGAAACTGACTAGTACCACTAATATTAGGAGCACCAGACATAGCATTCCCACGAATAAACAACATAAGATTCTGATTATTACCACCATCATTAACTCTTCTTCTCACT

AGTAGAACAGTAGAAAGTGGTGCAGGAACAGGATGAACAGTATACCCACCCCTTGCAAGAGGAATTGCCCACGCCGGAGCATCAGTAGATCTAGCCATCTTCTCATTACACTT

AGCAGGAGTATCATCCATCCTAGGAGCAGTAAACTTCATCTCAACAACAATCAACATGAAACCAAAAAACATAAAACCCGAACGAATCCCCTTATTCGTATGATCAGTTGCCAT

CACGGCTCTCCTACTCCTCCTATCACTACCAGTACTAGCAGGAGCAATCACAATACTATTAACTGACCGAAACCTAAACACATCCTTCTTTGATCCAGCAGGAGGTGGAGACCC

AATCCTATACCAACACTTATTCTGATTCTTCGGACACCCAGAAGTATATATTTTAATCCTACCAGGATTTGGTATAATTTCCCACATTATTTGTCACGAAAGAGGTAAAAAGGAA

GCCTTCGGAAACCTAGGAATAATTTTCGCCATATTAGCAATCGGATTACTAGGATTTGTAGTATGAGCACACCATATGTTCACAGTAGGAATAGACGTTGACACACGAGCTTAC

TTTACATCAGCAACAATAATCATTGCAGTACCTACGGGGATTAAAATCTTCAGATGACTTGCAACAATATACGGAACCCGAATAACTTATAGAGCAGCATGCCTATGAGCCCTA

GGATTTGTA--

>G1_Macrotermes_subhyalinus_77 Macrotermes subhyalinus isolate TB22 cytochrome oxidase subunit I (COI) gene, partial cds; mitochondrial

----

AACAGAACTTGGACAACCAGGATCCTTAATCGGGGACGACCAAATCTACAACGTCATCGTCACAGCTCACGCTTTCGTAATGATCTTCTTCATAGTAATACCAATTATGATTGG

AGGATTCGGAAACTGACTAGTACCACTAATATTAGGAGCACCAGACATAGCATTCCCACGAATAAACAACATAAGATTCTGATTATTACCACCATCATTAACTCTTCTTCTCACT

AGTAGAACAGTAGAAAGTGGTGCAGGAACAGGATGAACAGTATACCCACCCCTTGCAAGAGGAATTGCCCACGCCGGAGCATCAGTAGATCTAGCCATCTTCTCATTACACTT

AGCAGGAGTATCATCCATCCTAGGAGCAGTAAACTTCATCTCAACAACAATCAACATGAAACCAAAAAACATAAAACCCGAACGAATCCCCTTATTCGTATGATCAGTTGCCAT

CACGGCTCTCCTACTCCTCCTATCACTACCAGTACTAGCAGGAGCAATCACAATACTATTAACTGACCGAAACCTAAACACATCCTTCTTTGATCCAGCAGGAGGTGGAGACCC

AATCCTATACCAACACTTATTCTGATTCTTCGGACACCCAGAAGTATATATTTTAATCCTACCAGGATTTGGTATAATTTCCCACATTATTTGTCACGAAAGAGGTAAAAAGGAA

GCCTTCGGAAACCTAGGAATAATTTTCGCCATATTAGCAATCGGATTACTAGGATTTGTAGTATGAGCACACCATATGTTCACAGTAGGAATAGACGTTGACACACGAGCTTAC

TTTACATCAGCAACAATAATCATTGCAGTACCTACGGGGATTAAAATCTTCAGATGACTTGCAACAATATACGGAACCCGAATAACTTATAGAGCAGCATGCCTATGAGCCCTA

GGATTTGTA--

>G1_Macrotermes_subhyalinus_78 Macrotermes subhyalinus isolate TB23 cytochrome oxidase subunit I (COI) gene, partial cds; mitochondrial

----

AACAGAACTTGGACAACCAGGATCCTTAATCGGGGACGACCAAATCTACAACGTCATCGTCACAGCTCACGCTTTCGTAATGATCTTCTTCATAGTAATACCAATTATGATTGG

AGGATTCGGAAACTGACTAGTACCACTAATATTAGGAGCACCAGACATAGCATTCCCACGAATAAACAACATAAGATTCTGATTATTACCACCATCATTAACTCTTCTTCTCACT

AGTAGAACAGTAGAAAGTGGTGCAGGAACAGGATGAACAGTATACCCACCCCTTGCAAGAGGAATTGCCCACGCCGGAGCATCAGTAGATCTAGCCATCTTCTCATTACACTT

AGCAGGAGTATCATCCATCCTAGGAGCAGTAAACTTCATCTCAACAACAATCAACATGAAACCAAAAAACATAAAACCCGAACGAATCCCCTTATTCGTATGATCAGTTGCCAT

CACGGCTCTCCTACTCCTCCTATCACTACCAGTACTAGCAGGAGCAATCACAATACTATTAACTGACCGAAACCTAAACACATCCTTCTTTGATCCAGCAGGAGGTGGAGACCC

AATCCTATACCAACACTTATTCTGATTCTTCGGACACCCAGAAGTATATATTTTAATCCTACCAGGATTTGGTATAATTTCCCACATTATTTGTCACGAAAGAGGTAAAAAGGAA

GCCTTCGGAAACCTAGGAATAATTTTCGCCATATTAGCAATCGGATTACTAGGATTTGTAGTATGAGCACACCATATGTTCACAGTAGGAATAGACGTTGACACACGAGCTTAC

TTTACATCAGCAACAATAATCATTGCAGTACCTACGGGGATTAAAATCTTCAGATGACTTGCAACAATATACGGAACCCGAATAACTTATAGAGCAGCATGCCTATGAGCCCTA

GGATTTGTA--

>G1_Macrotermes_subhyalinus_79 Macrotermes subhyalinus isolate TB24 cytochrome oxidase subunit I (COI) gene, partial cds; mitochondrial

----

AACAGAACTTGGACAACCAGGATCCTTAATCGGGGACGACCAAATCTACAACGTCATCGTCACAGCTCACGCTTTCGTAATGATCTTCTTCATAGTAATACCAATTATGATTGG

AGGATTCGGAAACTGACTAGTACCACTAATATTAGGAGCACCAGACATAGCATTCCCACGAATAAACAACATAAGATTCTGATTATTACCACCATCATTAACTCTTCTTCTCACT

AGTAGAACAGTAGAAAGTGGTGCAGGAACAGGATGAACAGTATACCCACCCCTTGCAAGAGGAATTGCCCACGCCGGAGCATCAGTAGATCTAGCCATCTTCTCATTACACTT

AGCAGGAGTATCATCCATCCTAGGAGCAGTAAACTTCATCTCAACAACAATCAACATGAAACCAAAAAACATAAAACCCGAACGAATCCCCTTATTCGTATGATCAGTTGCCAT

CACGGCTCTCCTACTCCTCCTATCACTACCAGTACTAGCAGGAGCAATCACAATACTATTAACTGACCGAAACCTAAACACATCCTTCTTTGATCCAGCAGGAGGTGGAGACCC

AATCCTATACCAACACTTATTCTGATTCTTCGGACACCCAGAAGTATATATTTTAATCCTACCAGGATTTGGTATAATTTCCCACATTATTTGTCACGAAAGAGGTAAAAAGGAA

GCCTTCGGAAACCTAGGAATAATTTTCGCCATATTAGCAATCGGATTACTAGGATTTGTAGTATGAGCACACCATATGTTCACAGTAGGAATAGACGTTGACACACGAGCTTAC

TTTACATCAGCAACAATAATCATTGCAGTACCTACGGGGATTAAAATCTTCAGATGACTTGCAACAATATACGGAACCCGAATAACTTATAGAGCAGCATGCCTATGAGCCCTA

GGATTTGTA--

>G1_Macrotermes_subhyalinus_80 Macrotermes subhyalinus isolate TB25 cytochrome oxidase subunit I (COI) gene, partial cds; mitochondrial

----

AACAGAACTTGGACAACCAGGATCCTTAATCGGGGACGACCAAATCTACAACGTCATCGTCACAGCTCACGCTTTCGTAATGATCTTCTTCATAGTAATACCAATTATGATTGG

AGGATTCGGAAACTGACTAGTACCACTAATATTAGGAGCACCAGACATAGCATTCCCACGAATAAACAACATAAGATTCTGATTATTACCACCATCATTAACTCTTCTTCTCACT

AGTAGAACAGTAGAAAGTGGTGCAGGAACAGGATGAACAGTATACCCACCCCTTGCAAGAGGAATTGCCCACGCCGGAGCATCAGTAGATCTAGCCATCTTCTCATTACACTT

AGCAGGAGTATCATCCATCCTAGGAGCAGTAAACTTCATCTCAACAACAATCAACATGAAACCAAAAAACATAAAACCCGAACGAATCCCCTTATTCGTATGATCAGTTGCCAT

CACGGCTCTCCTACTCCTCCTATCACTACCAGTACTAGCAGGAGCAATCACAATACTATTAACTGACCGAAACCTAAACACATCCTTCTTTGATCCAGCAGGAGGTGGAGACCC

AATCCTATACCAACACTTATTCTGATTCTTCGGACACCCAGAAGTATATATTTTAATCCTACCAGGATTTGGTATAATTTCCCACATTATTTGTCACGAAAGAGGTAAAAAGGAA

GCCTTCGGAAACCTAGGAATAATTTTCGCCATATTAGCAATCGGATTACTAGGATTTGTAGTATGAGCACACCATATGTTCACAGTAGGAATAGACGTTGACACACGAGCTTAC

TTTACATCAGCAACAATAATCATTGCAGTACCTACGGGGATTAAAATCTTCAGATGACTTGCAACAATATACGGAACCCGAATAACTTATAGAGCAGCATGCCTATGAGCCCTA

GGATTTGTA--

>G1_Macrotermes_subhyalinus_81 Macrotermes subhyalinus isolate TB33 cytochrome oxidase subunit I (COI) gene, partial cds; mitochondrial

----

AACAGAACTTGGACAACCAGGATCCTTAATCGGGGACGACCAAATCTACAACGTCATCGTCACAGCTCACGCTTTCGTAATGATCTTCTTCATAGTAATACCAATTATGATTGG

AGGATTCGGAAACTGACTAGTACCACTAATATTAGGAGCACCAGACATAGCATTCCCACGAATAAACAACATAAGATTCTGATTATTACCACCATCATTAACTCTTCTTCTCACT

AGTAGAACAGTAGAAAGTGGTGCAGGAACAGGATGAACAGTATACCCACCCCTTGCAAGAGGAATTGCCCACGCCGGAGCATCAGTAGATCTAGCCATCTTCTCATTACACTT

AGCAGGAGTATCATCCATCCTAGGAGCAGTAAACTTCATCTCAACAACAATCAACATGAAACCAAAAAACATAAAACCCGAACGAATCCCCTTATTCGTATGATCAGTTGCCAT

CACGGCTCTCCTACTCCTCCTATCACTACCAGTACTAGCAGGAGCAATCACAATACTATTAACTGACCGAAACCTAAACACATCCTTCTTTGATCCAGCAGGAGGTGGAGACCC

AATCCTATACCAACACTTATTCTGATTCTTCGGACACCCAGAAGTATATATTTTAATCCTACCAGGATTTGGTATAATTTCCCACATTATTTGTCACGAAAGAGGTAAAAAGGAA

GCCTTCGGAAACCTAGGAATAATTTTCGCCATATTAGCAATCGGATTACTAGGATTTGTAGTATGAGCACACCATATGTTCACAGTAGGAATAGACGTTGACACACGAGCTTAC

TTTACATCAGCAACAATAATCATTGCAGTACCTACGGGGATTAAAATCTTCAGATGACTTGCAACAATATACGGAACCCGAATAACTTATAGAGCAGCATGCCTATGAGCCCTA

GGATTTGTA--

>G1_Macrotermes_subhyalinus_82 Macrotermes subhyalinus isolate TB35 cytochrome oxidase subunit I (COI) gene, partial cds; mitochondrial

----

AACAGAACTTGGACAACCAGGATCCTTAATCGGGGACGACCAAATCTACAACGTCATCGTCACAGCTCACGCTTTCGTAATGATCTTCTTCATAGTAATACCAATTATGATTGG

AGGATTCGGAAACTGACTAGTACCACTAATATTAGGAGCACCAGACATAGCATTCCCACGAATAAACAACATAAGATTCTGATTATTACCACCATCATTAACTCTTCTTCTCACT

AGTAGAACAGTAGAAAGTGGTGCAGGAACAGGATGAACAGTATACCCACCCCTTGCAAGAGGAATTGCCCACGCCGGAGCATCAGTAGATCTAGCCATCTTCTCATTACACTT

AGCAGGAGTATCATCCATCCTAGGAGCAGTAAACTTCATCTCAACAACAATCAACATGAAACCAAAAAACATAAAACCCGAACGAATCCCCTTATTCGTATGATCAGTTGCCAT

CACGGCTCTCCTACTCCTCCTATCACTACCAGTACTAGCAGGAGCAATCACAATACTATTAACTGACCGAAACCTAAACACATCCTTCTTTGATCCAGCAGGAGGTGGAGACCC

AATCCTATACCAACACTTATTCTGATTCTTCGGACACCCAGAAGTATATATTTTAATCCTACCAGGATTTGGTATAATTTCCCACATTATTTGTCACGAAAGAGGTAAAAAGGAA

GCCTTCGGAAACCTAGGAATAATTTTCGCCATATTAGCAATCGGATTACTAGGATTTGTAGTATGAGCACACCATATGTTCACAGTAGGAATAGACGTTGACACACGAGCTTAC

TTTACATCAGCAACAATAATCATTGCAGTACCTACGGGGATTAAAATCTTCAGATGACTTGCAACAATATACGGAACCCGAATAACTTATAGAGCAGCATGCCTATGAGCCCTA
GGATTTGTA---

>G1_Macrotermes_subhyalinus_83 Macrotermes subhyalinus isolate TB36 cytochrome oxidase subunit I (COI) gene, partial cds; mitochondrial

----
AACAGAACTTGGACAACCAGGATCCTTAATCGGGGACGACCAAATCTACAACGTCATCGTCACAGCTCACGCTTTCGTAATGATCTTCTTCATAGTAATACCAATTATGATTGG
AGGATTCGGAAACTGACTAGTACCACTAATATTAGGAGCACCAGACATAGCATTCCCACGAATAAACAACATAAGATTCTGATTATTACCACCATCATTAACTCTTCTTCTCACT
AGTAGAACAGTAGAAAGTGGTGCAGGAACAGGATGAACAGTATACCCACCCCTTGCAAGAGGAATTGCCCACGCCGGAGCATCAGTAGATCTAGCCATCTTCTCATTACACTT
AGCAGGAGTATCATCCATCCTAGGAGCAGTAAACTTCATCTCAACAACAATCAACATGAAACCAAAAAACATAAAACCCGAACGAATCCCCTTATTCGTATGATCAGTTGCCAT
CACGGCTCTCCTACTCCTCCTATCACTACCAGTACTAGCAGGAGCAATCACAATACTATTAACTGACCGAAACCTAAACACATCCTTCTTTGATCCAGCAGGAGGTGGAGACCC
AATCCTATACCAACACTTATTCTGATTCTTCGGACACCCAGAAGTATATATTTTAATCCTACCAGGATTTGGTATAATTTCCCACATTATTTGTCACGAAAGAGGTAAAAAGGAA
GCCTTCGGAAACCTAGGAATAATTTTCGCCATATTAGCAATCGGATTACTAGGATTTGTAGTATGAGCACACCATATGTTCACAGTAGGAATAGACGTTGACACACGAGCTTAC
TTTACATCAGCAACAATAATCATTGCAGTACCTACGGGGATTAAAATCTTCAGATGACTTGCAACAATATACGGAACCCGAATAACTTATAGAGCAGCATGCCTATGAGCCCTA
GGATTTGTA---

>G1_Macrotermes_subhyalinus_84 Macrotermes subhyalinus isolate TB37 cytochrome oxidase subunit I (COI) gene, partial cds; mitochondrial

----

AACAGAACTTGGACAACCAGGATCCTTAATCGGGGACGACCAAATCTACAACGTCATCGTCACAGCTCACGCTTTCGTAATGATCTTCTTCATAGTAATACCAATTATGATTGG

AGGATTCGGAAACTGACTAGTACCACTAATATTAGGAGCACCAGACATAGCATTCCCACGAATAAACAACATAAGATTCTGATTATTACCACCATCATTAACTCTTCTTCTCACT

AGTAGAACAGTAGAAAGTGGTGCAGGAACAGGATGAACAGTATACCCACCCCTTGCAAGAGGAATTGCCCACGCCGGAGCATCAGTAGATCTAGCCATCTTCTCATTACACTT

AGCAGGAGTATCATCCATCCTAGGAGCAGTAAACTTCATCTCAACAACAATCAACATGAAACCAAAAAACATAAAACCCGAACGAATCCCCTTATTCGTATGATCAGTTGCCAT

CACGGCTCTCCTACTCCTCCTATCACTACCAGTACTAGCAGGAGCAATCACAATACTATTAACTGACCGAAACCTAAACACATCCTTCTTTGATCCAGCAGGAGGTGGAGACCC

AATCCTATACCAACACTTATTCTGATTCTTCGGACACCCAGAAGTATATATTTTAATCCTACCAGGATTTGGTATAATTTCCCACATTATTTGTCACGAAAGAGGTAAAAAGGAA

GCCTTCGGAAACCTAGGAATAATTTTCGCCATATTAGCAATCGGATTACTAGGATTTGTAGTATGAGCACACCATATGTTCACAGTAGGAATAGACGTTGACACACGAGCTTAC

TTTACATCAGCAACAATAATCATTGCAGTACCTACGGGGATTAAAATCTTCAGATGACTTGCAACAATATACGGAACCCGAATAACTTATAGAGCAGCATGCCTATGAGCCCTA

GGATTTGTA---

>G1_Macrotermes_subhyalinus_85 Macrotermes subhyalinus isolate TFA11 cytochrome oxidase subunit I (COI) gene, partial cds; mitochondrial

----

AACAGAACTTGGACAACCAGGATCCTTAATCGGGGACGACCAAATCTACAACGTCATCGTCACAGCTCACGCTTTCGTAATGATCTTCTTCATAGTAATACCAATTATGATTGG

AGGATTCGGAAACTGACTAGTACCACTAATATTAGGAGCACCAGACATAGCATTCCCACGAATAAACAACATAAGATTCTGATTATTACCACCATCATTAACTCTTCTTCTCACT

AGTAGAACAGTAGAAAGTGGTGCAGGAACAGGATGAACAGTATACCCACCCCTTGCAAGAGGAATTGCCCACGCCGGAGCATCAGTAGATCTAGCCATCTTCTCATTACACTT

AGCAGGAGTATCATCCATCCTAGGAGCAGTAAACTTCATCTCAACAACAATCAACATGAAACCAAAAAACATAAAACCCGAACGAATCCCCTTATTCGTATGATCAGTTGCCAT

CACGGCTCTCCTACTCCTCCTATCACTACCAGTACTAGCAGGAGCAATCACAATACTATTAACTGACCGAAACCTAAACACATCCTTCTTTGATCCAGCAGGAGGTGGAGACCC

AATCCTATACCAACACTTATTCTGATTCTTCGGACACCCAGAAGTATATATTTTAATCCTACCAGGATTTGGTATAATTTCCCACATTATTTGTCACGAAAGAGGTAAAAAGGAA

GCCTTCGGAAACCTAGGAATAATTTTCGCCATATTAGCAATCGGATTACTAGGATTTGTAGTATGAGCACACCATATGTTCACAGTAGGAATAGACGTTGACACACGAGCTTAC

TTTACATCAGCAACAATAATCATTGCAGTACCTACGGGGATTAAAATCTTCAGATGACTTGCAACAATATACGGAACCCGAATAACTTATAGAGCAGCATGCCTATGAGCCCTA

GGATTTGTA--

>G1_Macrotermes_subhyalinus_86 Macrotermes subhyalinus isolate TFA12 cytochrome oxidase subunit I (COI) gene, partial cds; mitochondrial

----

AACAGAACTTGGACAACCAGGATCCTTAATCGGGGACGACCAAATCTACAACGTCATCGTCACAGCTCACGCTTTCGTAATGATCTTCTTCATAGTAATACCAATTATGATTGG

AGGATTCGGAAACTGACTAGTACCACTAATATTAGGAGCACCAGACATAGCATTCCCACGAATAAACAACATAAGATTCTGATTATTACCACCATCATTAACTCTTCTTCTCACT

AGTAGAACAGTAGAAAGTGGTGCAGGAACAGGATGAACAGTATACCCACCCCTTGCAAGAGGAATTGCCCACGCCGGAGCATCAGTAGATCTAGCCATCTTCTCATTACACTT

AGCAGGAGTATCATCCATCCTAGGAGCAGTAAACTTCATCTCAACAACAATCAACATGAAACCAAAAAACATAAAACCCGAACGAATCCCCTTATTCGTATGATCAGTTGCCAT

CACGGCTCTCCTACTCCTCCTATCACTACCAGTACTAGCAGGAGCAATCACAATACTATTAACTGACCGAAACCTAAACACATCCTTCTTTGATCCAGCAGGAGGTGGAGACCC

AATCCTATACCAACACTTATTCTGATTCTTCGGACACCCAGAAGTATATATTTTAATCCTACCAGGATTTGGTATAATTTCCCACATTATTTGTCACGAAAGAGGTAAAAAGGAA

GCCTTCGGAAACCTAGGAATAATTTTCGCCATATTAGCAATCGGATTACTAGGATTTGTAGTATGAGCACACCATATGTTCACAGTAGGAATAGACGTTGACACACGAGCTTAC

TTTACATCAGCAACAATAATCATTGCAGTACCTACGGGGATTAAAATCTTCAGATGACTTGCAACAATATACGGAACCCGAATAACTTATAGAGCAGCATGCCTATGAGCCCTA

GGATTTGTA--

>G1_Macrotermes_subhyalinus_87 Macrotermes subhyalinus isolate TFA13 cytochrome oxidase subunit I (COI) gene, partial cds; mitochondrial

----

AACAGAACTTGGACAACCAGGATCCTTAATCGGGGACGACCAAATCTACAACGTCATCGTCACAGCTCACGCTTTCGTAATGATCTTCTTCATAGTAATACCAATTATGATTGG

AGGATTCGGAAACTGACTAGTACCACTAATATTAGGAGCACCAGACATAGCATTCCCACGAATAAACAACATAAGATTCTGATTATTACCACCATCATTAACTCTTCTTCTCACT

AGTAGAACAGTAGAAAGTGGTGCAGGAACAGGATGAACAGTATACCCACCCCTTGCAAGAGGAATTGCCCACGCCGGAGCATCAGTAGATCTAGCCATCTTCTCATTACACTT

AGCAGGAGTATCATCCATCCTAGGAGCAGTAAACTTCATCTCAACAACAATCAACATGAAACCAAAAAACATAAAACCCGAACGAATCCCCTTATTCGTATGATCAGTTGCCAT

CACGGCTCTCCTACTCCTCCTATCACTACCAGTACTAGCAGGAGCAATCACAATACTATTAACTGACCGAAACCTAAACACATCCTTCTTTGATCCAGCAGGAGGTGGAGACCC

AATCCTATACCAACACTTATTCTGATTCTTCGGACACCCAGAAGTATATATTTTAATCCTACCAGGATTTGGTATAATTTCCCACATTATTTGTCACGAAAGAGGTAAAAAGGAA

GCCTTCGGAAACCTAGGAATAATTTTCGCCATATTAGCAATCGGATTACTAGGATTTGTAGTATGAGCACACCATATGTTCACAGTAGGAATAGACGTTGACACACGAGCTTAC

TTTACATCAGCAACAATAATCATTGCAGTACCTACGGGGATTAAAATCTTCAGATGACTTGCAACAATATACGGAACCCGAATAACTTATAGAGCAGCATGCCTATGAGCCCTA

GGATTTGTA---

>G1_Macrotermes_subhyalinus_88 Macrotermes subhyalinus isolate TFA14 cytochrome oxidase subunit I (COI) gene, partial cds; mitochondrial

----

AACAGAACTTGGACAACCAGGATCCTTAATCGGGGACGACCAAATCTACAACGTCATCGTCACAGCTCACGCTTTCGTAATGATCTTCTTCATAGTAATACCAATTATGATTGG

AGGATTCGGAAACTGACTAGTACCACTAATATTAGGAGCACCAGACATAGCATTCCCACGAATAAACAACATAAGATTCTGATTATTACCACCATCATTAACTCTTCTTCTCACT

AGTAGAACAGTAGAAAGTGGTGCAGGAACAGGATGAACAGTATACCCACCCCTTGCAAGAGGAATTGCCCACGCCGGAGCATCAGTAGATCTAGCCATCTTCTCATTACACTT

AGCAGGAGTATCATCCATCCTAGGAGCAGTAAACTTCATCTCAACAACAATCAACATGAAACCAAAAAACATAAAACCCGAACGAATCCCCTTATTCGTATGATCAGTTGCCAT

CACGGCTCTCCTACTCCTCCTATCACTACCAGTACTAGCAGGAGCAATCACAATACTATTAACTGACCGAAACCTAAACACATCCTTCTTTGATCCAGCAGGAGGTGGAGACCC

AATCCTATACCAACACTTATTCTGATTCTTCGGACACCCAGAAGTATATATTTTAATCCTACCAGGATTTGGTATAATTTCCCACATTATTTGTCACGAAAGAGGTAAAAAGGAA

GCCTTCGGAAACCTAGGAATAATTTTCGCCATATTAGCAATCGGATTACTAGGATTTGTAGTATGAGCACACCATATGTTCACAGTAGGAATAGACGTTGACACACGAGCTTAC

TTTACATCAGCAACAATAATCATTGCAGTACCTACGGGGATTAAAATCTTCAGATGACTTGCAACAATATACGGAACCCGAATAACTTATAGAGCAGCATGCCTATGAGCCCTA

GGATTTGTA--

>G1_Macrotermes_subhyalinus_89 Macrotermes subhyalinus isolate TFA21 cytochrome oxidase subunit I (COI) gene, partial cds; mitochondrial

----

AACAGAACTTGGACAACCAGGATCCTTAATCGGGGACGACCAAATCTACAACGTCATCGTCACAGCTCACGCTTTCGTAATGATCTTCTTCATAGTAATACCAATTATGATTGG

AGGATTCGGAAACTGACTAGTACCACTAATATTAGGAGCACCAGACATAGCATTCCCACGAATAAACAACATAAGATTCTGATTATTACCACCATCATTAACTCTTCTTCTCACT

AGTAGAACAGTAGAAAGTGGTGCAGGAACAGGATGAACAGTATACCCACCCCTTGCAAGAGGAATTGCCCACGCCGGAGCATCAGTAGATCTAGCCATCTTCTCATTACACTT

AGCAGGAGTATCATCCATCCTAGGAGCAGTAAACTTCATCTCAACAACAATCAACATGAAACCAAAAAACATAAAACCCGAACGAATCCCCTTATTCGTATGATCAGTTGCCAT

CACGGCTCTCCTACTCCTCCTATCACTACCAGTACTAGCAGGAGCAATCACAATACTATTAACTGACCGAAACCTAAACACATCCTTCTTTGATCCAGCAGGAGGTGGAGACCC

AATCCTATACCAACACTTATTCTGATTCTTCGGACACCCAGAAGTATATATTTTAATCCTACCAGGATTTGGTATAATTTCCCACATTATTTGTCACGAAAGAGGTAAAAAGGAA

GCCTTCGGAAACCTAGGAATAATTTTCGCCATATTAGCAATCGGATTACTAGGATTTGTAGTATGAGCACACCATATGTTCACAGTAGGAATAGACGTTGACACACGAGCTTAC

TTTACATCAGCAACAATAATCATTGCAGTACCTACGGGGATTAAAATCTTCAGATGACTTGCAACAATATACGGAACCCGAATAACTTATAGAGCAGCATGCCTATGAGCCCTA

GGATTTGTA--

>G1_Macrotermes_subhyalinus_90 Macrotermes subhyalinus isolate TFA28 cytochrome oxidase subunit I (COI) gene, partial cds; mitochondrial

----

AACAGAACTTGGACAACCAGGATCCTTAATCGGGGACGACCAAATCTACAACGTCATCGTCACAGCTCACGCTTTCGTAATGATCTTCTTCATAGTAATACCAATTATGATTGG

AGGATTCGGAAACTGACTAGTACCACTAATATTAGGAGCACCAGACATAGCATTCCCACGAATAAACAACATAAGATTCTGATTATTACCACCATCATTAACTCTTCTTCTCACT

AGTAGAACAGTAGAAAGTGGTGCAGGAACAGGATGAACAGTATACCCACCCCTTGCAAGAGGAATTGCCCACGCCGGAGCATCAGTAGATCTAGCCATCTTCTCATTACACTT

AGCAGGAGTATCATCCATCCTAGGAGCAGTAAACTTCATCTCAACAACAATCAACATGAAACCAAAAAACATAAAACCCGAACGAATCCCCTTATTCGTATGATCAGTTGCCAT

CACGGCTCTCCTACTCCTCCTATCACTACCAGTACTAGCAGGAGCAATCACAATACTATTAACTGACCGAAACCTAAACACATCCTTCTTTGATCCAGCAGGAGGTGGAGACCC

AATCCTATACCAACACTTATTCTGATTCTTCGGACACCCAGAAGTATATATTTTAATCCTACCAGGATTTGGTATAATTTCCCACATTATTTGTCACGAAAGAGGTAAAAAGGAA

GCCTTCGGAAACCTAGGAATAATTTTCGCCATATTAGCAATCGGATTACTAGGATTTGTAGTATGAGCACACCATATGTTCACAGTAGGAATAGACGTTGACACACGAGCTTAC

TTTACATCAGCAACAATAATCATTGCAGTACCTACGGGGATTAAAATCTTCAGATGACTTGCAACAATATACGGAACCCGAATAACTTATAGAGCAGCATGCCTATGAGCCCTA

GGATTTGTA--

>G1_Macrotermes_subhyalinus_91 Macrotermes subhyalinus isolate TFA35 cytochrome oxidase subunit I (COI) gene, partial cds; mitochondrial

----

AACAGAACTTGGACAACCAGGATCCTTAATCGGGGACGACCAAATCTACAACGTCATCGTCACAGCTCACGCTTTCGTAATGATCTTCTTCATAGTAATACCAATTATGATTGG

AGGATTCGGAAACTGACTAGTACCACTAATATTAGGAGCACCAGACATAGCATTCCCACGAATAAACAACATAAGATTCTGATTATTACCACCATCATTAACTCTTCTTCTCACT

AGTAGAACAGTAGAAAGTGGTGCAGGAACAGGATGAACAGTATACCCACCCCTTGCAAGAGGAATTGCCCACGCCGGAGCATCAGTAGATCTAGCCATCTTCTCATTACACTT

AGCAGGAGTATCATCCATCCTAGGAGCAGTAAACTTCATCTCAACAACAATCAACATGAAACCAAAAAACATAAAACCCGAACGAATCCCCTTATTCGTATGATCAGTTGCCAT

CACGGCTCTCCTACTCCTCCTATCACTACCAGTACTAGCAGGAGCAATCACAATACTATTAACTGACCGAAACCTAAACACATCCTTCTTTGATCCAGCAGGAGGTGGAGACCC

AATCCTATACCAACACTTATTCTGATTCTTCGGACACCCAGAAGTATATATTTTAATCCTACCAGGATTTGGTATAATTTCCCACATTATTTGTCACGAAAGAGGTAAAAAGGAA

GCCTTCGGAAACCTAGGAATAATTTTCGCCATATTAGCAATCGGATTACTAGGATTTGTAGTATGAGCACACCATATGTTCACAGTAGGAATAGACGTTGACACACGAGCTTAC

TTTACATCAGCAACAATAATCATTGCAGTACCTACGGGGATTAAAATCTTCAGATGACTTGCAACAATATACGGAACCCGAATAACTTATAGAGCAGCATGCCTATGAGCCCTA

GGATTTGTA--

>G1_Macrotermes_subhyalinus_92 Macrotermes subhyalinus isolate TFA49 cytochrome oxidase subunit I (COI) gene, partial cds; mitochondrial

----

AACAGAACTTGGACAACCAGGATCCTTAATCGGGGACGACCAAATCTACAACGTCATCGTCACAGCTCACGCTTTCGTAATGATCTTCTTCATAGTAATACCAATTATGATTGG

AGGATTCGGAAACTGACTAGTACCACTAATATTAGGAGCACCAGACATAGCATTCCCACGAATAAACAACATAAGATTCTGATTATTACCACCATCATTAACTCTTCTTCTCACT

AGTAGAACAGTAGAAAGTGGTGCAGGAACAGGATGAACAGTATACCCACCCCTTGCAAGAGGAATTGCCCACGCCGGAGCATCAGTAGATCTAGCCATCTTCTCATTACACTT

AGCAGGAGTATCATCCATCCTAGGAGCAGTAAACTTCATCTCAACAACAATCAACATGAAACCAAAAAACATAAAACCCGAACGAATCCCCTTATTCGTATGATCAGTTGCCAT

CACGGCTCTCCTACTCCTCCTATCACTACCAGTACTAGCAGGAGCAATCACAATACTATTAACTGACCGAAACCTAAACACATCCTTCTTTGATCCAGCAGGAGGTGGAGACCC

AATCCTATACCAACACTTATTCTGATTCTTCGGACACCCAGAAGTATATATTTTAATCCTACCAGGATTTGGTATAATTTCCCACATTATTTGTCACGAAAGAGGTAAAAAGGAA

GCCTTCGGAAACCTAGGAATAATTTTCGCCATATTAGCAATCGGATTACTAGGATTTGTAGTATGAGCACACCATATGTTCACAGTAGGAATAGACGTTGACACACGAGCTTAC

TTTACATCAGCAACAATAATCATTGCAGTACCTACGGGGATTAAAATCTTCAGATGACTTGCAACAATATACGGAACCCGAATAACTTATAGAGCAGCATGCCTATGAGCCCTA

GGATTTGTA--

>G1_Macrotermes_subhyalinus_93 Macrotermes subhyalinus isolate TFB20 cytochrome oxidase subunit I (COI) gene, partial cds; mitochondrial

----

AACAGAACTTGGACAACCAGGATCCTTAATCGGGGACGACCAAATCTACAACGTCATCGTCACAGCTCACGCTTTCGTAATGATCTTCTTCATAGTAATACCAATTATGATTGG

AGGATTCGGAAACTGACTAGTACCACTAATATTAGGAGCACCAGACATAGCATTCCCACGAATAAACAACATAAGATTCTGATTATTACCACCATCATTAACTCTTCTTCTCACT

AGTAGAACAGTAGAAAGTGGTGCAGGAACAGGATGAACAGTATACCCACCCCTTGCAAGAGGAATTGCCCACGCCGGAGCATCAGTAGATCTAGCCATCTTCTCATTACACTT

AGCAGGAGTATCATCCATCCTAGGAGCAGTAAACTTCATCTCAACAACAATCAACATGAAACCAAAAAACATAAAACCCGAACGAATCCCCTTATTCGTATGATCAGTTGCCAT

CACGGCTCTCCTACTCCTCCTATCACTACCAGTACTAGCAGGAGCAATCACAATACTATTAACTGACCGAAACCTAAACACATCCTTCTTTGATCCAGCAGGAGGTGGAGACCC

AATCCTATACCAACACTTATTCTGATTCTTCGGACACCCAGAAGTATATATTTTAATCCTACCAGGATTTGGTATAATTTCCCACATTATTTGTCACGAAAGAGGTAAAAAGGAA

GCCTTCGGAAACCTAGGAATAATTTTCGCCATATTAGCAATCGGATTACTAGGATTTGTAGTATGAGCACACCATATGTTCACAGTAGGAATAGACGTTGACACACGAGCTTAC

TTTACATCAGCAACAATAATCATTGCAGTACCTACGGGGATTAAAATCTTCAGATGACTTGCAACAATATACGGAACCCGAATAACTTATAGAGCAGCATGCCTATGAGCCCTA

GGATTTGTA---

>G1_Macrotermes_subhyalinus_94 Macrotermes subhyalinus isolate TFB34 cytochrome oxidase subunit I (COI) gene, partial cds; mitochondrial

----

AACAGAACTTGGACAACCAGGATCCTTAATCGGGGACGACCAAATCTACAACGTCATCGTCACAGCTCACGCTTTCGTAATGATCTTCTTCATAGTAATACCAATTATGATTGG

AGGATTCGGAAACTGACTAGTACCACTAATATTAGGAGCACCAGACATAGCATTCCCACGAATAAACAACATAAGATTCTGATTATTACCACCATCATTAACTCTTCTTCTCACT

AGTAGAACAGTAGAAAGTGGTGCAGGAACAGGATGAACAGTATACCCACCCCTTGCAAGAGGAATTGCCCACGCCGGAGCATCAGTAGATCTAGCCATCTTCTCATTACACTT

AGCAGGAGTATCATCCATCCTAGGAGCAGTAAACTTCATCTCAACAACAATCAACATGAAACCAAAAAACATAAAACCCGAACGAATCCCCTTATTCGTATGATCAGTTGCCAT

CACGGCTCTCCTACTCCTCCTATCACTACCAGTACTAGCAGGAGCAATCACAATACTATTAACTGACCGAAACCTAAACACATCCTTCTTTGATCCAGCAGGAGGTGGAGACCC

AATCCTATACCAACACTTATTCTGATTCTTCGGACACCCAGAAGTATATATTTTAATCCTACCAGGATTTGGTATAATTTCCCACATTATTTGTCACGAAAGAGGTAAAAAGGAA

GCCTTCGGAAACCTAGGAATAATTTTCGCCATATTAGCAATCGGATTACTAGGATTTGTAGTATGAGCACACCATATGTTCACAGTAGGAATAGACGTTGACACACGAGCTTAC

TTTACATCAGCAACAATAATCATTGCAGTACCTACGGGGATTAAAATCTTCAGATGACTTGCAACAATATACGGAACCCGAATAACTTATAGAGCAGCATGCCTATGAGCCCTA

GGATTTGTA---

>G1_Macrotermes_subhyalinus_95 Macrotermes subhyalinus isolate TFB50 cytochrome oxidase subunit I (COI) gene, partial cds; mitochondrial

----

AACAGAACTTGGACAACCAGGATCCTTAATCGGGGACGACCAAATCTACAACGTCATCGTCACAGCTCACGCTTTCGTAATGATCTTCTTCATAGTAATACCAATTATGATTGG

AGGATTCGGAAACTGACTAGTACCACTAATATTAGGAGCACCAGACATAGCATTCCCACGAATAAACAACATAAGATTCTGATTATTACCACCATCATTAACTCTTCTTCTCACT

AGTAGAACAGTAGAAAGTGGTGCAGGAACAGGATGAACAGTATACCCACCCCTTGCAAGAGGAATTGCCCACGCCGGAGCATCAGTAGATCTAGCCATCTTCTCATTACACTT

AGCAGGAGTATCATCCATCCTAGGAGCAGTAAACTTCATCTCAACAACAATCAACATGAAACCAAAAAACATAAAACCCGAACGAATCCCCTTATTCGTATGATCAGTTGCCAT

CACGGCTCTCCTACTCCTCCTATCACTACCAGTACTAGCAGGAGCAATCACAATACTATTAACTGACCGAAACCTAAACACATCCTTCTTTGATCCAGCAGGAGGTGGAGACCC

AATCCTATACCAACACTTATTCTGATTCTTCGGACACCCAGAAGTATATATTTTAATCCTACCAGGATTTGGTATAATTTCCCACATTATTTGTCACGAAAGAGGTAAAAAGGAA

GCCTTCGGAAACCTAGGAATAATTTTCGCCATATTAGCAATCGGATTACTAGGATTTGTAGTATGAGCACACCATATGTTCACAGTAGGAATAGACGTTGACACACGAGCTTAC

TTTACATCAGCAACAATAATCATTGCAGTACCTACGGGGATTAAAATCTTCAGATGACTTGCAACAATATACGGAACCCGAATAACTTATAGAGCAGCATGCCTATGAGCCCTA

GGATTTGTA---

>G1_Macrotermes_subhyalinus_96 Macrotermes subhyalinus isolate TA06 cytochrome oxidase subunit I (COI) gene, partial cds; mitochondrial

----

AACAGAACTTGGACAACCAGGATCCTTAATCGGGGACGACCAAATCTACAACGTCATCGTCACAGCTCACGCTTTCGTAATGATCTTCTTCATAGTAATACCAATTATGATTGG

AGGATTCGGAAACTGACTAGTACCACTAATATTAGGAGCACCAGACATAGCATTCCCACGAATAAACAACATAAGATTCTGATTATTACCACCATCATTAACTCTTCTTCTCACT

AGTAGAACAGTAGAAAGTGGTGCAGGAACAGGATGAACAGTATACCCACCCCTTGCAAGAGGAATTGCCCACGCCGGAGCATCAGTAGATCTAGCCATCTTCTCATTACACTT

AGCAGGAGTATCATCCATCCTAGGAGCAGTAAACTTCATCTCAACAACAATCAACATGAAACCAAAAAACATAAAACCCGAACGAATCCCCTTATTCGTATGATCAGTTGCCAT

CACGGCTCTCCTACTCCTCCTATCACTACCAGTACTAGCAGGAGCAATCACAATACTATTAACTGACCGAAACCTAAACACATCCTTCTTTGATCCAGCAGGAGGTGGAGACCC

AATCCTATACCAACACTTATTCTGATTCTTCGGACACCCAGAAGTATATATTTTAATCCTACCAGGATTTGGTATAATTTCCCACATTATTTGTCACGAAAGAGGTAAAAAGGAA

GCCTTCGGAAACCTAGGAATAATTTTCGCCATATTAGCAATCGGATTACTAGGATTTGTAGTATGAGCACACCATATGTTCACAGTAGGAATAGACGTTGACACACGAGCTTAC

TTTACATCAGCAACAATAATCATTGCAGTACCTACGGGGATTAAAATCTTCAGATGACTTGCAACAATATACGGAACCCGAATAACTTATAGAGCAGCATGCCTATGAGCCCTA

GGATTTGTA---

>G1_Macrotermes_subhyalinus_97 Macrotermes subhyalinus isolate TA11 cytochrome oxidase subunit I (COI) gene, partial cds; mitochondrial

----

AACAGAACTTGGACAACCAGGATCCTTAATCGGGGACGACCAAATCTACAACGTCATCGTCACAGCTCACGCTTTCGTAATGATCTTCTTCATAGTAATACCAATTATGATTGG

AGGATTCGGAAACTGACTAGTACCACTAATATTAGGAGCACCAGACATAGCATTCCCACGAATAAACAACATAAGATTCTGATTATTACCACCATCATTAACTCTTCTTCTCACT

AGTAGAACAGTAGAAAGTGGTGCAGGAACAGGATGAACAGTATACCCACCCCTTGCAAGAGGAATTGCCCACGCCGGAGCATCAGTAGATCTAGCCATCTTCTCATTACACTT

AGCAGGAGTATCATCCATCCTAGGAGCAGTAAACTTCATCTCAACAACAATCAACATGAAACCAAAAAACATAAAACCCGAACGAATCCCCTTATTCGTATGATCAGTTGCCAT

CACGGCTCTCCTACTCCTCCTATCACTACCAGTACTAGCAGGAGCAATCACAATACTATTAACTGACCGAAACCTAAACACATCCTTCTTTGATCCAGCAGGAGGTGGAGACCC

AATCCTATACCAACACTTATTCTGATTCTTCGGACACCCAGAAGTATATATTTTAATCCTACCAGGATTTGGTATAATTTCCCACATTATTTGTCACGAAAGAGGTAAAAAGGAA

GCCTTCGGAAACCTAGGAATAATTTTCGCCATATTAGCAATCGGATTACTAGGATTTGTAGTATGAGCACACCATATGTTCACAGTAGGAATAGACGTTGACACACGAGCTTAC

TTTACATCAGCAACAATAATCATTGCAGTACCTACGGGGATTAAAATCTTCAGATGACTTGCAACAATATACGGAACCCGAATAACTTATAGAGCAGCATGCCTATGAGCCCTA

GGATTTGTA--

>G1_Macrotermes_subhyalinus_98 Macrotermes subhyalinus isolate TA12 cytochrome oxidase subunit I (COI) gene, partial cds; mitochondrial

----

AACAGAACTTGGACAACCAGGATCCTTAATCGGGGACGACCAAATCTACAACGTCATCGTCACAGCTCACGCTTTCGTAATGATCTTCTTCATAGTAATACCAATTATGATTGG

AGGATTCGGAAACTGACTAGTACCACTAATATTAGGAGCACCAGACATAGCATTCCCACGAATAAACAACATAAGATTCTGATTATTACCACCATCATTAACTCTTCTTCTCACT

AGTAGAACAGTAGAAAGTGGTGCAGGAACAGGATGAACAGTATACCCACCCCTTGCAAGAGGAATTGCCCACGCCGGAGCATCAGTAGATCTAGCCATCTTCTCATTACACTT

AGCAGGAGTATCATCCATCCTAGGAGCAGTAAACTTCATCTCAACAACAATCAACATGAAACCAAAAAACATAAAACCCGAACGAATCCCCTTATTCGTATGATCAGTTGCCAT

CACGGCTCTCCTACTCCTCCTATCACTACCAGTACTAGCAGGAGCAATCACAATACTATTAACTGACCGAAACCTAAACACATCCTTCTTTGATCCAGCAGGAGGTGGAGACCC

AATCCTATACCAACACTTATTCTGATTCTTCGGACACCCAGAAGTATATATTTTAATCCTACCAGGATTTGGTATAATTTCCCACATTATTTGTCACGAAAGAGGTAAAAAGGAA

GCCTTCGGAAACCTAGGAATAATTTTCGCCATATTAGCAATCGGATTACTAGGATTTGTAGTATGAGCACACCATATGTTCACAGTAGGAATAGACGTTGACACACGAGCTTAC

TTTACATCAGCAACAATAATCATTGCAGTACCTACGGGGATTAAAATCTTCAGATGACTTGCAACAATATACGGAACCCGAATAACTTATAGAGCAGCATGCCTATGAGCCCTA

GGATTTGTA--

>G1_Macrotermes_subhyalinus_99 Macrotermes subhyalinus isolate TA19 cytochrome oxidase subunit I (COI) gene, partial cds; mitochondrial

----

AACAGAACTTGGACAACCAGGATCCTTAATCGGGGACGACCAAATCTACAACGTCATCGTCACAGCTCACGCTTTCGTAATGATCTTCTTCATAGTAATACCAATTATGATTGG

AGGATTCGGAAACTGACTAGTACCACTAATATTAGGAGCACCAGACATAGCATTCCCACGAATAAACAACATAAGATTCTGATTATTACCACCATCATTAACTCTTCTTCTCACT

AGTAGAACAGTAGAAAGTGGTGCAGGAACAGGATGAACAGTATACCCACCCCTTGCAAGAGGAATTGCCCACGCCGGAGCATCAGTAGATCTAGCCATCTTCTCATTACACTT

AGCAGGAGTATCATCCATCCTAGGAGCAGTAAACTTCATCTCAACAACAATCAACATGAAACCAAAAAACATAAAACCCGAACGAATCCCCTTATTCGTATGATCAGTTGCCAT

CACGGCTCTCCTACTCCTCCTATCACTACCAGTACTAGCAGGAGCAATCACAATACTATTAACTGACCGAAACCTAAACACATCCTTCTTTGATCCAGCAGGAGGTGGAGACCC

AATCCTATACCAACACTTATTCTGATTCTTCGGACACCCAGAAGTATATATTTTAATCCTACCAGGATTTGGTATAATTTCCCACATTATTTGTCACGAAAGAGGTAAAAAGGAA

GCCTTCGGAAACCTAGGAATAATTTTCGCCATATTAGCAATCGGATTACTAGGATTTGTAGTATGAGCACACCATATGTTCACAGTAGGAATAGACGTTGACACACGAGCTTAC

TTTACATCAGCAACAATAATCATTGCAGTACCTACGGGGATTAAAATCTTCAGATGACTTGCAACAATATACGGAACCCGAATAACTTATAGAGCAGCATGCCTATGAGCCCTA

GGATTTGTA--

>G1_Macrotermes_subhyalinus_100 Macrotermes subhyalinus isolate TA21 cytochrome oxidase subunit I (COI) gene, partial cds; mitochondrial

----

AACAGAACTTGGACAACCAGGATCCTTAATCGGGGACGACCAAATCTACAACGTCATCGTCACAGCTCACGCTTTCGTAATGATCTTCTTCATAGTAATACCAATTATGATTGG

AGGATTCGGAAACTGACTAGTACCACTAATATTAGGAGCACCAGACATAGCATTCCCACGAATAAACAACATAAGATTCTGATTATTACCACCATCATTAACTCTTCTTCTCACT

AGTAGAACAGTAGAAAGTGGTGCAGGAACAGGATGAACAGTATACCCACCCCTTGCAAGAGGAATTGCCCACGCCGGAGCATCAGTAGATCTAGCCATCTTCTCATTACACTT

AGCAGGAGTATCATCCATCCTAGGAGCAGTAAACTTCATCTCAACAACAATCAACATGAAACCAAAAAACATAAAACCCGAACGAATCCCCTTATTCGTATGATCAGTTGCCAT

CACGGCTCTCCTACTCCTCCTATCACTACCAGTACTAGCAGGAGCAATCACAATACTATTAACTGACCGAAACCTAAACACATCCTTCTTTGATCCAGCAGGAGGTGGAGACCC

AATCCTATACCAACACTTATTCTGATTCTTCGGACACCCAGAAGTATATATTTTAATCCTACCAGGATTTGGTATAATTTCCCACATTATTTGTCACGAAAGAGGTAAAAAGGAA

GCCTTCGGAAACCTAGGAATAATTTTCGCCATATTAGCAATCGGATTACTAGGATTTGTAGTATGAGCACACCATATGTTCACAGTAGGAATAGACGTTGACACACGAGCTTAC

TTTACATCAGCAACAATAATCATTGCAGTACCTACGGGGATTAAAATCTTCAGATGACTTGCAACAATATACGGAACCCGAATAACTTATAGAGCAGCATGCCTATGAGCCCTA
GGATTTGTA--

>G1_Macrotermes_subhyalinus_101 Macrotermes subhyalinus isolate TA29 cytochrome oxidase subunit I (COI) gene, partial cds; mitochondrial

----
AACAGAACTTGGACAACCAGGATCCTTAATCGGGGACGACCAAATCTACAACGTCATCGTCACAGCTCACGCTTTCGTAATGATCTTCTTCATAGTAATACCAATTATGATTGG
AGGATTCGGAAACTGACTAGTACCACTAATATTAGGAGCACCAGACATAGCATTCCCACGAATAAACAACATAAGATTCTGATTATTACCACCATCATTAACTCTTCTTCTCACT
AGTAGAACAGTAGAAAGTGGTGCAGGAACAGGATGAACAGTATACCCACCCCTTGCAAGAGGAATTGCCCACGCCGGAGCATCAGTAGATCTAGCCATCTTCTCATTACACTT
AGCAGGAGTATCATCCATCCTAGGAGCAGTAAACTTCATCTCAACAACAATCAACATGAAACCAAAAAACATAAAACCCGAACGAATCCCCTTATTCGTATGATCAGTTGCCAT
CACGGCTCTCCTACTCCTCCTATCACTACCAGTACTAGCAGGAGCAATCACAATACTATTAACTGACCGAAACCTAAACACATCCTTCTTTGATCCAGCAGGAGGTGGAGACCC
AATCCTATACCAACACTTATTCTGATTCTTCGGACACCCAGAAGTATATATTTTAATCCTACCAGGATTTGGTATAATTTCCCACATTATTTGTCACGAAAGAGGTAAAAAGGAA
GCCTTCGGAAACCTAGGAATAATTTTCGCCATATTAGCAATCGGATTACTAGGATTTGTAGTATGAGCACACCATATGTTCACAGTAGGAATAGACGTTGACACACGAGCTTAC
TTTACATCAGCAACAATAATCATTGCAGTACCTACGGGGATTAAAATCTTCAGATGACTTGCAACAATATACGGAACCCGAATAACTTATAGAGCAGCATGCCTATGAGCCCTA
GGATTTGTA--

>G1_Macrotermes_subhyalinus_102 Macrotermes subhyalinus isolate TA30 cytochrome oxidase subunit I (COI) gene, partial cds; mitochondrial

----

AACAGAACTTGGACAACCAGGATCCTTAATCGGGGACGACCAAATCTACAACGTCATCGTCACAGCTCACGCTTTCGTAATGATCTTCTTCATAGTAATACCAATTATGATTGG

AGGATTCGGAAACTGACTAGTACCACTAATATTAGGAGCACCAGACATAGCATTCCCACGAATAAACAACATAAGATTCTGATTATTACCACCATCATTAACTCTTCTTCTCACT

AGTAGAACAGTAGAAAGTGGTGCAGGAACAGGATGAACAGTATACCCACCCCTTGCAAGAGGAATTGCCCACGCCGGAGCATCAGTAGATCTAGCCATCTTCTCATTACACTT

AGCAGGAGTATCATCCATCCTAGGAGCAGTAAACTTCATCTCAACAACAATCAACATGAAACCAAAAAACATAAAACCCGAACGAATCCCCTTATTCGTATGATCAGTTGCCAT

CACGGCTCTCCTACTCCTCCTATCACTACCAGTACTAGCAGGAGCAATCACAATACTATTAACTGACCGAAACCTAAACACATCCTTCTTTGATCCAGCAGGAGGTGGAGACCC

AATCCTATACCAACACTTATTCTGATTCTTCGGACACCCAGAAGTATATATTTTAATCCTACCAGGATTTGGTATAATTTCCCACATTATTTGTCACGAAAGAGGTAAAAAGGAA

GCCTTCGGAAACCTAGGAATAATTTTCGCCATATTAGCAATCGGATTACTAGGATTTGTAGTATGAGCACACCATATGTTCACAGTAGGAATAGACGTTGACACACGAGCTTAC

TTTACATCAGCAACAATAATCATTGCAGTACCTACGGGGATTAAAATCTTCAGATGACTTGCAACAATATACGGAACCCGAATAACTTATAGAGCAGCATGCCTATGAGCCCTA

GGATTTGTA--

>G1_Macrotermes_subhyalinus_103 Macrotermes subhyalinus isolate TA32 cytochrome oxidase subunit I (COI) gene, partial cds; mitochondrial

----

AACAGAACTTGGACAACCAGGATCCTTAATCGGGGACGACCAAATCTACAACGTCATCGTCACAGCTCACGCTTTCGTAATGATCTTCTTCATAGTAATACCAATTATGATTGG

AGGATTCGGAAACTGACTAGTACCACTAATATTAGGAGCACCAGACATAGCATTCCCACGAATAAACAACATAAGATTCTGATTATTACCACCATCATTAACTCTTCTTCTCACT

AGTAGAACAGTAGAAAGTGGTGCAGGAACAGGATGAACAGTATACCCACCCCTTGCAAGAGGAATTGCCCACGCCGGAGCATCAGTAGATCTAGCCATCTTCTCATTACACTT

AGCAGGAGTATCATCCATCCTAGGAGCAGTAAACTTCATCTCAACAACAATCAACATGAAACCAAAAAACATAAAACCCGAACGAATCCCCTTATTCGTATGATCAGTTGCCAT

CACGGCTCTCCTACTCCTCCTATCACTACCAGTACTAGCAGGAGCAATCACAATACTATTAACTGACCGAAACCTAAACACATCCTTCTTTGATCCAGCAGGAGGTGGAGACCC

AATCCTATACCAACACTTATTCTGATTCTTCGGACACCCAGAAGTATATATTTTAATCCTACCAGGATTTGGTATAATTTCCCACATTATTTGTCACGAAAGAGGTAAAAAGGAA

GCCTTCGGAAACCTAGGAATAATTTTCGCCATATTAGCAATCGGATTACTAGGATTTGTAGTATGAGCACACCATATGTTCACAGTAGGAATAGACGTTGACACACGAGCTTAC

TTTACATCAGCAACAATAATCATTGCAGTACCTACGGGGATTAAAATCTTCAGATGACTTGCAACAATATACGGAACCCGAATAACTTATAGAGCAGCATGCCTATGAGCCCTA
GGATTTGTA--

>G1_Macrotermes_subhyalinus_104 Macrotermes subhyalinus isolate TY01 cytochrome oxidase subunit I (COI) gene, partial cds; mitochondrial

----
AACAGAACTTGGACAACCAGGATCCTTAATCGGGGACGACCAAATCTACAACGTCATCGTCACAGCTCACGCTTTCGTAATGATCTTCTTCATAGTAATACCAATTATGATTGG
AGGATTCGGAAACTGACTAGTACCACTAATATTAGGAGCACCAGACATAGCATTCCCACGAATAAACAACATAAGATTCTGATTATTACCACCATCATTAACTCTTCTTCTCACT
AGTAGAACAGTAGAAAGTGGTGCAGGAACAGGATGAACAGTATACCCACCCCTTGCAAGAGGAATTGCCCACGCCGGAGCATCAGTAGATCTAGCCATCTTCTCATTACACTT
AGCAGGAGTATCATCCATCCTAGGAGCAGTAAACTTCATCTCAACAACAATCAACATGAAACCAAAAAACATAAAACCCGAACGAATCCCCTTATTCGTATGATCAGTTGCCAT
CACGGCTCTCCTACTCCTCCTATCACTACCAGTACTAGCAGGAGCAATCACAATACTATTAACTGACCGAAACCTAAACACATCCTTCTTTGATCCAGCAGGAGGTGGAGACCC
AATCCTATACCAACACTTATTCTGATTCTTCGGACACCCAGAAGTATATATTTTAATCCTACCAGGATTTGGTATAATTTCCCACATTATTTGTCACGAAAGAGGTAAAAAGGAA
GCCTTCGGAAACCTAGGAATAATTTTCGCCATATTAGCAATCGGATTACTAGGATTTGTAGTATGAGCACACCATATGTTCACAGTAGGAATAGACGTTGACACACGAGCTTAC
TTTACATCAGCAACAATAATCATTGCAGTACCTACGGGGATTAAAATCTTCAGATGACTTGCAACAATATACGGAACCCGAATAACTTATAGAGCAGCATGCCTATGAGCCCTA
GGATTTGTA--

>G1_Macrotermes_subhyalinus_105 Macrotermes subhyalinus isolate TY02 cytochrome oxidase subunit I (COI) gene, partial cds; mitochondrial

----

AACAGAACTTGGACAACCAGGATCCTTAATCGGGGACGACCAAATCTACAACGTCATCGTCACAGCTCACGCTTTCGTAATGATCTTCTTCATAGTAATACCAATTATGATTGG

AGGATTCGGAAACTGACTAGTACCACTAATATTAGGAGCACCAGACATAGCATTCCCACGAATAAACAACATAAGATTCTGATTATTACCACCATCATTAACTCTTCTTCTCACT

AGTAGAACAGTAGAAAGTGGTGCAGGAACAGGATGAACAGTATACCCACCCCTTGCAAGAGGAATTGCCCACGCCGGAGCATCAGTAGATCTAGCCATCTTCTCATTACACTT

AGCAGGAGTATCATCCATCCTAGGAGCAGTAAACTTCATCTCAACAACAATCAACATGAAACCAAAAAACATAAAACCCGAACGAATCCCCTTATTCGTATGATCAGTTGCCAT

CACGGCTCTCCTACTCCTCCTATCACTACCAGTACTAGCAGGAGCAATCACAATACTATTAACTGACCGAAACCTAAACACATCCTTCTTTGATCCAGCAGGAGGTGGAGACCC

AATCCTATACCAACACTTATTCTGATTCTTCGGACACCCAGAAGTATATATTTTAATCCTACCAGGATTTGGTATAATTTCCCACATTATTTGTCACGAAAGAGGTAAAAAGGAA

GCCTTCGGAAACCTAGGAATAATTTTCGCCATATTAGCAATCGGATTACTAGGATTTGTAGTATGAGCACACCATATGTTCACAGTAGGAATAGACGTTGACACACGAGCTTAC

TTTACATCAGCAACAATAATCATTGCAGTACCTACGGGGATTAAAATCTTCAGATGACTTGCAACAATATACGGAACCCGAATAACTTATAGAGCAGCATGCCTATGAGCCCTA

GGATTTGTA---

>G1_Macrotermes_subhyalinus_106 Macrotermes subhyalinus isolate TY17 cytochrome oxidase subunit I (COI) gene, partial cds; mitochondrial

----

AACAGAACTTGGACAACCAGGATCCTTAATCGGGGACGACCAAATCTACAACGTCATCGTCACAGCTCACGCTTTCGTAATGATCTTCTTCATAGTAATACCAATTATGATTGG

AGGATTCGGAAACTGACTAGTACCACTAATATTAGGAGCACCAGACATAGCATTCCCACGAATAAACAACATAAGATTCTGATTATTACCACCATCATTAACTCTTCTTCTCACT

AGTAGAACAGTAGAAAGTGGTGCAGGAACAGGATGAACAGTATACCCACCCCTTGCAAGAGGAATTGCCCACGCCGGAGCATCAGTAGATCTAGCCATCTTCTCATTACACTT

AGCAGGAGTATCATCCATCCTAGGAGCAGTAAACTTCATCTCAACAACAATCAACATGAAACCAAAAAACATAAAACCCGAACGAATCCCCTTATTCGTATGATCAGTTGCCAT

CACGGCTCTCCTACTCCTCCTATCACTACCAGTACTAGCAGGAGCAATCACAATACTACTAACTGACCGAAACCTAAACACATCCTTCTTTGATCCAGCAGGAGGTGGAGACCC

AATCCTATACCAACACTTATTCTGATTCTTCGGACACCCAGAAGTATATATTTTAATCCTACCAGGATTTGGTATAATTTCCCACATTATTTGTCACGAAAGAGGTAAAAAGGAA

GCCTTCGGAAACCTAGGAATAATTTTCGCCATATTAGCAATCGGATTACTAGGATTTGTAGTATGAGCACACCATATGTTCACAGTAGGAATAGACGTTGACACACGAGCTTAC

TTTACATCAGCAACAATAATCATTGCAGTACCTACGGGGATTAAAATCTTCAGATGACTTGCAACAATATACGGAACCCGAATAACTTATAGAGCAGCATGCCTATGAGCCCTA

GGATTTGTA---

>G1_Macrotermes_subhyalinus_107 Macrotermes subhyalinus isolate TA02 cytochrome oxidase subunit I (COI) gene, partial cds; mitochondrial

----

AACAGAACTTGGACAACCAGGATCCTTAATCGGGGACGACCAAATCTACAACGTCATCGTCACAGCTCACGCTTTCGTAATGATCTTCTTCATAGTAATACCAATTATGATTGG

AGGATTCGGAAACTGACTAGTACCACTAATATTAGGAGCACCAGACATAGCATTCCCACGAATAAACAACATAAGATTCTGATTATTACCACCATCATTAACTCTTCTTCTCACT

AGTAGAACAGTAGAAAGTGGTGCAGGAACAGGATGAACAGTATACCCACCCCTTGCAAGAGGAATTGCCCACGCCGGAGCATCAGTAGATCTAGCCATCTTCTCATTACACTT

AGCAGGAGTATCATCCATCCTAGGAGCAGTAAACTTCATCTCAACAACAATCAACATGAAACCAAAAAACATAAAACCCGAACGAATCCCCTTATTCGTATGATCAGTTGCCAT

CACGGCTCTCCTACTCCTCCTATCACTACCAGTACTAGCAGGAGCAATCACAATACTACTAACTGACCGAAACCTAAACACATCCTTCTTTGATCCAGCAGGAGGTGGAGACCC

AATCCTATACCAACACTTATTCTGATTCTTCGGACACCCAGAAGTATATATTTTAATCCTACCAGGATTTGGTATAATTTCCCACATTATTTGTCACGAAAGAGGTAAAAAGGAA

GCCTTCGGAAACCTAGGAATAATTTTCGCCATATTAGCAATCGGATTACTAGGATTTGTAGTATGAGCACACCATATGTTCACAGTAGGAATAGACGTTGACACACGAGCTTAC

TTTACATCAGCAACAATAATCATTGCAGTACCTACGGGGATTAAAATCTTCAGATGACTTGCAACAATATACGGAACCCGAATAACTTATAGAGCAGCATGCCTATGAGCCCTA

GGATTTGTA---

>G1_Macrotermes_subhyalinus_108 Macrotermes subhyalinus isolate TA26 cytochrome oxidase subunit I (COI) gene, partial cds; mitochondrial

----

AACAGAACTTGGACAACCAGGATCCTTAATCGGGGACGACCAAATCTACAACGTCATCGTCACAGCTCACGCTTTCGTAATGATCTTCTTCATAGTAATACCAATTATGATTGG

AGGATTCGGAAACTGACTAGTACCACTAATATTAGGAGCACCAGACATAGCATTCCCACGAATAAACAACATAAGATTCTGATTATTACCACCATCATTAACTCTTCTTCTCACT

AGTAGAACAGTAGAAAGTGGTGCAGGAACAGGATGAACAGTATACCCACCCCTTGCAAGAGGAATTGCCCACGCCGGAGCATCAGTAGATCTAGCCATCTTCTCATTACACTT

AGCAGGAGTATCATCCATCCTAGGAGCAGTAAACTTCATCTCAACAACAATCAACATGAAACCAAAAAACATAAAACCCGAACGAATCCCCTTATTCGTATGATCAGTTGCCAT

CACGGCTCTCCTACTCCTCCTATCACTACCAGTACTAGCAGGAGCAATCACAATACTACTAACTGACCGAAACCTAAACACATCCTTCTTTGATCCAGCAGGAGGTGGAGACCC

AATCCTATACCAACACTTATTCTGATTCTTCGGACACCCAGAAGTATATATTTTAATCCTACCAGGATTTGGTATAATTTCCCACATTATTTGTCACGAAAGAGGTAAAAAGGAA

GCCTTCGGAAACCTAGGAATAATTTTCGCCATATTAGCAATCGGATTACTAGGATTTGTAGTATGAGCACACCATATGTTCACAGTAGGAATAGACGTTGACACACGAGCTTAC

TTTACATCAGCAACAATAATCATTGCAGTACCTACGGGGATTAAAATCTTCAGATGACTTGCAACAATATACGGAACCCGAATAACTTATAGAGCAGCATGCCTATGAGCCCTA

GGATTTGTA--

>G1_Macrotermes_subhyalinus_109 Macrotermes subhyalinus isolate TK02 cytochrome oxidase subunit I (COI) gene, partial cds; mitochondrial

----

AACAGAACTTGGACAACCAGGATCCTTAATCGGGGACGACCAAATCTACAACGTCATCGTCACAGCTCACGCTTTCGTAATGATCTTCTTCATAGTAATACCAATTATGATTGG

AGGATTCGGAAACTGACTAGTACCACTAATATTAGGAGCACCAGACATAGCATTCCCACGAATAAATAACATAAGATTCTGATTATTACCACCATCATTAACTCTTCTTCTCACT

AGTAGAACAGTAGAAAGTGGTGCAGGAACAGGATGAACAGTATACCCACCCCTTGCAAGAGGAATTGCCCACGCCGGAGCATCAGTAGATCTAGCCATCTTCTCATTACACTT

AGCAGGAGTATCATCCATCCTAGGAGCAGTAAACTTCATCTCAACAACAATCAACATGAAACCAAAAAACATAAAACCCGAACGAATCCCCTTATTCGTATGATCAGTTGCCAT

CACGGCTCTCCTACTCCTCCTATCACTACCAGTACTAGCAGGAGCAATCACAATACTATTAACTGACCGAAACCTAAACACATCCTTCTTTGATCCAGCAGGAGGTGGAGACCC

AATCCTATACCAACACTTATTCTGATTCTTCGGACACCCAGAAGTATATATTTTAATCCTACCAGGATTTGGTATAATTTCCCACATTATTTGTCACGAAAGAGGTAAAAAGGAA

GCCTTCGGAAACCTAGGAATAATTTTCGCCATATTAGCAATCGGATTACTAGGATTTGTAGTATGAGCACACCATATGTTCACAGTAGGAATAGACGTTGACACACGAGCTTAC

TTTACATCAGCAACAATAATCATTGCAGTACCTACGGGGATTAAAATCTTCAGATGACTTGCAACAATATACGGAACCCGAATAACTTATAGAGCAGCATGCCTATGAGCCCTA

GGATTTGTA---

>G1_Macrotermes_subhyalinus_110 Macrotermes subhyalinus isolate TK06 cytochrome oxidase subunit I (COI) gene, partial cds; mitochondrial

----

AACAGAACTTGGACAACCAGGATCCTTAATTGGGGACGACCAAATCTACAACGTCATCGTCACAGCTCACGCTTTCGTAATGATCTTCTTCATAGTAATACCAATTATGATTGG

AGGATTCGGAAACTGACTAGTACCACTAATATTAGGAGCACCAGACATAGCATTCCCACGAATAAACAACATAAGATTCTGATTATTACCACCATCATTAACTCTTCTTCTCACT

AGTAGAACAGTAGAAAGTGGTGCAGGAACAGGATGAACAGTATACCCACCCCTTGCAAGAGGAATTGCCCACGCCGGAGCATCAGTAGATCTAGCCATCTTCTCATTACACTT

AGCAGGAGTATCATCCATCCTAGGAGCAGTAAACTTCATCTCAACAACAATCAACATGAAACCAAAAAACATAAAACCCGAACGAATCCCCTTATTCGTATGATCAGTTGCCAT

CACGGCTCTCCTACTCCTCCTATCACTACCAGTACTAGCAGGAGCAATCACAATACTATTAACTGACCGAAACCTAAACACATCCTTCTTTGATCCAGCAGGAGGTGGAGACCC

AATCCTATACCAACACTTATTCTGATTCTTCGGACACCCAGAAGTATATATTTTAATCCTACCAGGATTTGGTATAATTTCCCACATTATTTGTCACGAAAGAGGTAAAAAGGAA

GCCTTCGGAAACCTAGGAATAATTTTCGCCATATTAGCAATCGGATTACTAGGATTTGTAGTATGAGCACACCATATGTTCACAGTAGGAATAGACGTTGACACACGAGCTTAC

TTTACATCAGCAACAATAATCATTGCAGTACCTACGGGGATTAAAATCTTCAGATGACTTGCAACAATATACGGAACCCGAATAACTTATAGAGCAGCATGCCTATGAGCCCTA

GGATTTGTA---

>G1_Macrotermes_subhyalinus_111 Macrotermes subhyalinus isolate TK10 cytochrome oxidase subunit I (COI) gene, partial cds; mitochondrial

----

AACAGAACTTGGACAACCAGGATCCTTAATTGGGGACGACCAAATCTACAACGTCATCGTCACAGCTCACGCTTTCGTAATGATCTTCTTCATAGTAATACCAATTATGATTGG

AGGATTCGGAAACTGACTAGTACCACTAATATTAGGAGCACCAGACATAGCATTCCCACGAATAAACAACATAAGATTCTGATTATTACCACCATCATTAACTCTTCTTCTCACT

AGTAGAACAGTAGAAAGTGGTGCAGGAACAGGATGAACAGTATACCCACCCCTTGCAAGAGGAATTGCCCACGCCGGAGCATCAGTAGATCTAGCCATCTTCTCATTACACTT

AGCAGGAGTATCATCCATCCTAGGAGCAGTAAACTTCATCTCAACAACAATCAACATGAAACCAAAAAACATAAAACCCGAACGAATCCCCTTATTCGTATGATCAGTTGCCAT

CACGGCTCTCCTACTCCTCCTATCACTACCAGTACTAGCAGGAGCAATCACAATACTATTAACTGACCGAAACCTAAACACATCCTTCTTTGATCCAGCAGGAGGTGGAGACCC

AATCCTATACCAACACTTATTCTGATTCTTCGGACACCCAGAAGTATATATTTTAATCCTACCAGGATTTGGTATAATTTCCCACATTATTTGTCACGAAAGAGGTAAAAAGGAA

GCCTTCGGAAACCTAGGAATAATTTTCGCCATATTAGCAATCGGATTACTAGGATTTGTAGTATGAGCACACCATATGTTCACAGTAGGAATAGACGTTGACACACGAGCTTAC

TTTACATCAGCAACAATAATCATTGCAGTACCTACGGGGATTAAAATCTTCAGATGACTTGCAACAATATACGGAACCCGAATAACTTATAGAGCAGCATGCCTATGAGCCCTA

GGATTTGTA---

>G1_Macrotermes_subhyalinus_112 Macrotermes subhyalinus isolate TK14 cytochrome oxidase subunit I (COI) gene, partial cds; mitochondrial

----

AACAGAACTTGGACAACCAGGATCCTTAATTGGGGACGACCAAATCTACAACGTCATCGTCACAGCTCACGCTTTCGTAATGATCTTCTTCATAGTAATACCAATTATGATTGG

AGGATTCGGAAACTGACTAGTACCACTAATATTAGGAGCACCAGACATAGCATTCCCACGAATAAACAACATAAGATTCTGATTATTACCACCATCATTAACTCTTCTTCTCACT

AGTAGAACAGTAGAAAGTGGTGCAGGAACAGGATGAACAGTATACCCACCCCTTGCAAGAGGAATTGCCCACGCCGGAGCATCAGTAGATCTAGCCATCTTCTCATTACACTT

AGCAGGAGTATCATCCATCCTAGGAGCAGTAAACTTCATCTCAACAACAATCAACATGAAACCAAAAAACATAAAACCCGAACGAATCCCCTTATTCGTATGATCAGTTGCCAT

CACGGCTCTCCTACTCCTCCTATCACTACCAGTACTAGCAGGAGCAATCACAATACTATTAACTGACCGAAACCTAAACACATCCTTCTTTGATCCAGCAGGAGGTGGAGACCC

AATCCTATACCAACACTTATTCTGATTCTTCGGACACCCAGAAGTATATATTTTAATCCTACCAGGATTTGGTATAATTTCCCACATTATTTGTCACGAAAGAGGTAAAAAGGAA

GCCTTCGGAAACCTAGGAATAATTTTCGCCATATTAGCAATCGGATTACTAGGATTTGTAGTATGAGCACACCATATGTTCACAGTAGGAATAGACGTTGACACACGAGCTTAC

TTTACATCAGCAACAATAATCATTGCAGTACCTACGGGGATTAAAATCTTCAGATGACTTGCAACAATATACGGAACCCGAATAACTTATAGAGCAGCATGCCTATGAGCCCTA
GGATTTGTA--

>G1_Macrotermes_subhyalinus_113 Macrotermes subhyalinus isolate TM06 cytochrome oxidase subunit I (COI) gene, partial cds; mitochondrial

----
AACAGAACTTGGACAACCAGGATCCTTAATCGGGGACGACCAAATCTACAACGTCATCGTCACAGCTCACGCTTTCGTAATGATCTTCTTCATAGTAATACCAATTATGATTGG
AGGATTCGGAAACTGACTAGTACCACTAATATTAGGAGCACCAGACATAGCATTCCCACGAATAAACAACATAAGATTCTGATTATTACCACCATCATTAACTCTTCTTCTCACT
AGTAGAACAGTAGAAAGTGGTGCAGGAACAGGATGAACAGTATACCCACCCCTTGCAAGAGGAATTGCCCACGCCGGAGCATCAGTAGATCTAGCCATCTTCTCATTACACTT
AGCAGGAGTATCATCCATCCTAGGAGCAGTAAACTTCATCTCAACAACAATCAACATGAAACCAAAAAACATAAAACCCGAACGAATCCCCTTATTCGTATGATCAGTTGCCAT
CACGGCTCTCCTACTCCTCCTATCACTACCAGTACTAGCAGGAGCAATCACAATACTATTAACTGACCGAAACCTAAACACATCCTTCTTTGATCCAGCAGGAGGTGGAGACCC
AATCCTATACCAACACTTATTCTGATTCTTCGGACACCCAGAAGTATATATTTTAATCCTACCAGGATTTGGTATAATTTCCCACATTATTTGTCACGAAAGAGGTAAAAAGGAA
GCCTTCGGAAACCTAGGAATAATTTTCGCCATATTAGCAATCGGATTACTAGGATTTGTAGTATGAGCACACCATATGTTCACAGTAGGAATAGACGTTGACACACGAGCTTAC
TTTACATCAGCAACAATAATCATTGCAGTACCTACGGGGATTAAAATCTTCAGATGACTTGCAACAATATACGGAACCCGAATAACTTATAGAGCAGCAKGCCTATGAGCCCTA
GGATTTGTA--

>G1_Macrotermes_subhyalinus_114 Macrotermes subhyalinus isolate TK05 cytochrome oxidase subunit I (COI) gene, partial cds; mitochondrial

----

AACAGAACTTGGACAACCAGGATCCTTAATCGGGGACGACCAAATCTACAACGTCATCGTCACAGCTCACGCTTTCGTAATGATCTTCTTCATAGTAATACCAATTATGATTGG

AGGATTCGGAAACTGACTAGTACCACTAATATTAGGAGCACCAGACATAGCATTCCCACGAATAAACAACATAAGATTCTGATTATTACCACCATCATTAACTCTTCTTCTCACT

AGTAGAACAGTAGAAAGTGGTGCAGGAACAGGATGAACAGTATACCCACCCCTTGCAAGAGGAATTGCCCACGCCGGAGCATCAGTAGATCTAGCCATCTTCTCATTACACTT

AGCAGGAGTATCATCCATCCTAGGAGCAGTAAACTTCATCTCAACAACAATCAACATGAAACCAAAAAACATAAAACCCGAACGAATCCCCTTATTCGTATGATCAGTTGCCAT

CACGGCTCTCCTACTCCTCCTATCACTACCAGTACTAGCAGGAGCAATCACAATACTATTAACTGACCGAAACCTAAACACATCCTTCTTTGATCCAGCAGGAGGTGGAGACCC

AATCCTATACCAACACTTATTCTGATTCTTCGGACACCCAGAAGTATATATTTTAATCCTACCAGGATTTGGTATAATTTCCCACATTATTTGTCACGAAAGAGGTAAAAAGGAA

GCCTTCGGAAACCTAGGAATAATTTTCGCCATATTAGCAATCGGATTACTAGGATTTGTAGTATGAGCACATCATATGTTCACAGTAGGAATAGACGTTGACACACGAGCTTAC

TTTACATCAGCAACAATAATCATTGCAGTACCTACGGGGATTAAAATCTTCAGATGACTTGCAACAATATACGGAACCCGAATAACTTATAGAGCAGCATGCCTATGAGCCCTA

GGATTTGTA--

>G1_Macrotermes_subhyalinus_115 Macrotermes subhyalinus isolate TK09 cytochrome oxidase subunit I (COI) gene, partial cds; mitochondrial

----

AACAGAACTTGGACAACCAGGATCCTTAATCGGGGACGACCAAATCTACAACGTCATCGTCACAGCTCACGCTTTCGTAATGATCTTCTTCATAGTAATACCAATTATGATTGG

AGGATTCGGAAACTGACTAGTACCACTAATATTAGGAGCACCAGACATAGCATTCCCACGAATAAACAACATAAGATTCTGATTATTACCACCATCATTAACTCTTCTTCTCACT

AGTAGAACAGTAGAAAGTGGTGCAGGAACAGGATGAACAGTATACCCACCCCTTGCAAGAGGAATTGCCCACGCCGGAGCATCAGTAGATCTAGCCATCTTCTCATTACACTT

AGCAGGAGTATCATCCATCCTAGGAGCAGTAAACTTCATCTCAACAACAATCAACATGAAACCAAAAAACATAAAACCCGAACGAATCCCCTTATTCGTATGATCAGTTGCCAT

CACGGCTCTCCTACTCCTCCTATCACTACCAGTACTAGCAGGAGCAATCACAATACTATTAACTGACCGAAACCTAAACACATCCTTCTTTGATCCAGCAGGAGGTGGAGACCC

AATCCTATACCAACACTTATTCTGATTCTTCGGACACCCAGAAGTATATATTTTAATCCTACCAGGATTTGGTATAATTTCCCACATTATTTGTCACGAAAGAGGTAAAAAGGAA

GCCTTCGGAAACCTAGGAATAATTTTCGCCATATTAGCAATCGGATTACTAGGATTTGTAGTATGAGCACATCATATGTTCACAGTAGGAATAGACGTTGACACACGAGCTTAC

TTTACATCAGCAACAATAATCATTGCAGTACCTACGGGGATTAAAATCTTCAGATGACTTGCAACAATATACGGAACCCGAATAACTTATAGAGCAGCATGCCTATGAGCCCTA
GGATTTGTA---

>G1_Macrotermes_subhyalinus_116 Macrotermes subhyalinus isolate TK11 cytochrome oxidase subunit I (COI) gene, partial cds; mitochondrial

----
AACAGAACTTGGACAACCAGGATCCTTAATCGGGGACGACCAAATCTACAACGTCATCGTCACAGCTCACGCTTTCGTAATGATCTTCTTCATAGTAATACCAATTATGATTGG
AGGATTCGGAAACTGACTAGTACCACTAATATTAGGAGCACCAGACATAGCATTCCCACGAATAAACAACATAAGATTCTGATTATTACCACCATCATTAACTCTTCTTCTCACT
AGTAGAACAGTAGAAAGTGGTGCAGGAACAGGATGAACAGTATACCCACCCCTTGCAAGAGGAATTGCCCACGCCGGAGCATCAGTAGATCTAGCCATCTTCTCATTACACTT
AGCAGGAGTATCATCCATCCTAGGAGCAGTAAACTTCATCTCAACAACAATCAACATGAAACCAAAAAACATAAAACCCGAACGAATCCCCTTATTCGTATGATCAGTTGCCAT
CACGGCTCTCCTACTCCTCCTATCACTACCAGTACTAGCAGGAGCAATCACAATACTATTAACTGACCGAAACCTAAACACATCCTTCTTTGATCCAGCAGGAGGTGGAGACCC
AATCCTATACCAACACTTATTCTGATTCTTCGGACACCCAGAAGTATATATTTTAATCCTACCAGGATTTGGTATAATTTCCCACATTATTTGTCACGAAAGAGGTAAAAAGGAA
GCCTTCGGAAACCTAGGAATAATTTTCGCCATATTAGCAATCGGATTACTAGGATTTGTAGTATGAGCACATCATATGTTCACAGTAGGAATAGACGTTGACACACGAGCTTAC
TTTACATCAGCAACAATAATCATTGCAGTACCTACGGGGATTAAAATCTTCAGATGACTTGCAACAATATACGGAACCCGAATAACTTATAGAGCAGCATGCCTATGAGCCCTA
GGATTTGTA---

>G1_Macrotermes_subhyalinus_117 Macrotermes subhyalinus isolate TR10 cytochrome oxidase subunit I (COI) gene, partial cds; mitochondrial

----

AACAGAACTTGGACAACCAGGATCCTTAATCGGGGACGACCAAATCTACAACGTCATCGTCACAGCTCACGCTTTCGTAATGATCTTCTTCATAGTAATACCAATTATGATTGG

AGGATTCGGAAACTGACTAGTACCACTAATATTAGGAGCACCAGACATAGCATTCCCACGAATAAACAACATAAGATTCTGATTATTACCACCATCATTAACTCTTCTTCTCACT

AGTAGAACAGTAGAAAGTGGTGCAGGAACAGGATGAACAGTATACCCACCCCTTGCAAGAGGAATTGCCCACGCCGGAGCATCAGTAGATCTAGCCATCTTCTCATTACACTT

AGCAGGAGTATCATCCATCCTAGGAGCAGTAAACTTCATCTCAACAACAATCAACATGAAACCAAAAAACATAAAACCCGAACGAATCCCCTTATTCGTATGATCAGTTGCCAT

CACGGCTCTCCTACTCCTCCTATCACTACCAGTACTAGCAGGAGCAATCACAATACTATTAACTGACCGAAACCTAAACACATCCTTCTTTGATCCAGCAGGAGGTGGAGACCC

AATCCTATACCAACACTTATTCTGATTCTTCGGACACCCAGAAGTATATATTTTAATCCTACCAGGATTTGGTATAATTTCCCACATTATTTGTCACGAAAGAGGTAAAAAGGAA

GCCTTCGGAAACCTAGGAATAATTTTCGCCATATTAGCAATCGGATTACTAGGATTTGTAGTATGAGCACATCATATGTTCACAGTAGGAATAGACGTTGACACACGAGCTTAC

TTTACATCAGCAACAATAATCATTGCAGTACCTACGGGGATTAAAATCTTCAGATGACTTGCAACAATATACGGAACCCGAATAACTTATAGAGCAGCATGCCTATGAGCCCTA

GGATTTGTA--

>G1_Macrotermes_subhyalinus_118 Macrotermes subhyalinus isolate TR154 cytochrome oxidase subunit I (COI) gene, partial cds; mitochondrial

----

AACAGAACTTGGACAACCAGGATCCTTAATCGGGGACGACCAAATCTACAACGTCATCGTCACAGCTCACGCTTTCGTAATGATCTTCTTCATAGTAATACCAATTATGATTGG

AGGATTCGGAAACTGACTAGTACCACTAATATTAGGAGCACCAGACATAGCATTCCCACGAATAAACAACATAAGATTCTGATTATTACCACCATCATTAACTCTTCTTCTCACT

AGTAGAACAGTAGAAAGTGGTGCAGGAACAGGATGAACAGTATACCCACCCCTTGCAAGAGGAATTGCCCACGCCGGAGCATCAGTAGATCTAGCCATCTTCTCATTACACTT

AGCAGGAGTATCATCCATCCTAGGAGCAGTAAACTTCATCTCAACAACAATCAACATGAAACCAAAAAACATAAAACCCGAACGAATCCCCTTATTCGTATGATCAGTTGCCAT

CACGGCTCTCCTACTCCTCCTATCACTACCAGTACTAGCAGGAGCAATCACAATACTATTAACTGACCGAAACCTAAACACATCCTTCTTTGATCCAGCAGGAGGTGGAGACCC

AATCCTATACCAACACTTATTCTGATTCTTCGGACACCCAGAAGTATATATTTTAATCCTACCAGGATTTGGTATAATTTCCCACATTATTTGTCACGAAAGAGGTAAAAAGGAA

GCCTTCGGAAACCTAGGAATAATTTTCGCCATATTAGCAATCGGATTACTAGGATTTGTAGTATGAGCACATCATATGTTCACAGTAGGAATAGACGTTGACACACGAGCTTAC

TTTACATCAGCAACAATAATCATTGCAGTACCTACGGGGATTAAAATCTTCAGATGACTTGCAACAATATACGGAACCCGAATAACTTATAGAGCAGCATGCCTATGAGCCCTA

GGATTTGTA--

>G1_Macrotermes_subhyalinus_119 Macrotermes subhyalinus isolate TR156 cytochrome oxidase subunit I (COI) gene, partial cds; mitochondrial

----

AACAGAACTTGGACAACCAGGATCCTTAATCGGGGACGACCAAATCTACAACGTCATCGTCACAGCTCACGCTTTCGTAATGATCTTCTTCATAGTAATACCAATTATGATTGG

AGGATTCGGAAACTGACTAGTACCACTAATATTAGGAGCACCAGACATAGCATTCCCACGAATAAACAACATAAGATTCTGATTATTACCACCATCATTAACTCTTCTTCTCACT

AGTAGAACAGTAGAAAGTGGTGCAGGAACAGGATGAACAGTATACCCACCCCTTGCAAGAGGAATTGCCCACGCCGGAGCATCAGTAGATCTAGCCATCTTCTCATTACACTT

AGCAGGAGTATCATCCATCCTAGGAGCAGTAAACTTCATCTCAACAACAATCAACATGAAACCAAAAAACATAAAACCCGAACGAATCCCCTTATTCGTATGATCAGTTGCCAT

CACGGCTCTCCTACTCCTCCTATCACTACCAGTACTAGCAGGAGCAATCACAATACTATTAACTGACCGAAACCTAAACACATCCTTCTTTGATCCAGCAGGAGGTGGAGACCC

AATCCTATACCAACACTTATTCTGATTCTTCGGACACCCAGAAGTATATATTTTAATCCTACCAGGATTTGGTATAATTTCCCACATTATTTGTCACGAAAGAGGTAAAAAGGAA

GCCTTCGGAAACCTAGGAATAATTTTCGCCATATTAGCAATCGGATTACTAGGATTTGTAGTATGAGCACATCATATGTTCACAGTAGGAATAGACGTTGACACACGAGCTTAC

TTTACATCAGCAACAATAATCATTGCAGTACCTACGGGGATTAAAATCTTCAGATGACTTGCAACAATATACGGAACCCGAATAACTTATAGAGCAGCATGCCTATGAGCCCTA

GGATTTGTA--

>G1_Macrotermes_subhyalinus_120 Macrotermes subhyalinus isolate TR161 cytochrome oxidase subunit I (COI) gene, partial cds; mitochondrial

----

AACAGAACTTGGACAACCAGGATCCTTAATCGGGGACGACCAAATCTACAACGTCATCGTCACAGCTCACGCTTTCGTAATGATCTTCTTCATAGTAATACCAATTATGATTGG

AGGATTCGGAAACTGACTAGTACCACTAATATTAGGAGCACCAGACATAGCATTCCCACGAATAAACAACATAAGATTCTGATTATTACCACCATCATTAACTCTTCTTCTCACT

AGTAGAACAGTAGAAAGTGGTGCAGGAACAGGATGAACAGTATACCCACCCCTTGCAAGAGGAATTGCCCACGCCGGAGCATCAGTAGATCTAGCCATCTTCTCATTACACTT

AGCAGGAGTATCATCCATCCTAGGAGCAGTAAACTTCATCTCAACAACAATCAACATGAAACCAAAAAACATAAAACCCGAACGAATCCCCTTATTCGTATGATCAGTTGCCAT

CACGGCTCTCCTACTCCTCCTATCACTACCAGTACTAGCAGGAGCAATCACAATACTATTAACTGACCGAAACCTAAACACATCCTTCTTTGATCCAGCAGGAGGTGGAGACCC

AATCCTATACCAACACTTATTCTGATTCTTCGGACACCCAGAAGTATATATTTTAATCCTACCAGGATTTGGTATAATTTCCCACATTATTTGTCACGAAAGAGGTAAAAAGGAA

GCCTTCGGAAACCTAGGAATAATTTTCGCCATATTAGCAATCGGATTACTAGGATTTGTAGTATGAGCACATCATATGTTCACAGTAGGAATAGACGTTGACACACGAGCTTAC

TTTACATCAGCAACAATAATCATTGCAGTACCTACGGGGATTAAAATCTTCAGATGACTTGCAACAATATACGGAACCCGAATAACTTATAGAGCAGCATGCCTATGAGCCCTA

GGATTTGTA--

>G1_Macrotermes_subhyalinus_121 Macrotermes subhyalinus isolate TR167 cytochrome oxidase subunit I (COI) gene, partial cds; mitochondrial

----

AACAGAACTTGGACAACCAGGATCCTTAATCGGGGACGACCAAATCTACAACGTCATCGTCACAGCTCACGCTTTCGTAATGATCTTCTTCATAGTAATACCAATTATGATTGG

AGGATTCGGAAACTGACTAGTACCACTAATATTAGGAGCACCAGACATAGCATTCCCACGAATAAACAACATAAGATTCTGATTATTACCACCATCATTAACTCTTCTTCTCACT

AGTAGAACAGTAGAAAGTGGTGCAGGAACAGGATGAACAGTATACCCACCCCTTGCAAGAGGAATTGCCCACGCCGGAGCATCAGTAGATCTAGCCATCTTCTCATTACACTT

AGCAGGAGTATCATCCATCCTAGGAGCAGTAAACTTCATCTCAACAACAATCAACATGAAACCAAAAAACATAAAACCCGAACGAATCCCCTTATTCGTATGATCAGTTGCCAT

CACGGCTCTCCTACTCCTCCTATCACTACCAGTACTAGCAGGAGCAATCACAATACTATTAACTGACCGAAACCTAAACACATCCTTCTTTGATCCAGCAGGAGGTGGAGACCC

AATCCTATACCAACACTTATTCTGATTCTTCGGACACCCAGAAGTATATATTTTAATCCTACCAGGATTTGGTATAATTTCCCACATTATTTGTCACGAAAGAGGTAAAAAGGAA

GCCTTCGGAAACCTAGGAATAATTTTCGCCATATTAGCAATCGGATTACTAGGATTTGTAGTATGAGCACATCATATGTTCACAGTAGGAATAGACGTTGACACACGAGCTTAC

TTTACATCAGCAACAATAATCATTGCAGTACCTACGGGGATTAAAATCTTCAGATGACTTGCAACAATATACGGAACCCGAATAACTTATAGAGCAGCATGCCTATGAGCCCTA
GGATTTGTA--

>G1_Macrotermes_subhyalinus_122 Macrotermes subhyalinus isolate TR168 cytochrome oxidase subunit I (COI) gene, partial cds; mitochondrial

----
AACAGAACTTGGACAACCAGGATCCTTAATCGGGGACGACCAAATCTACAACGTCATCGTCACAGCTCACGCTTTCGTAATGATCTTCTTCATAGTAATACCAATTATGATTGG
AGGATTCGGAAACTGACTAGTACCACTAATATTAGGAGCACCAGACATAGCATTCCCACGAATAAACAACATAAGATTCTGATTATTACCACCATCATTAACTCTTCTTCTCACT
AGTAGAACAGTAGAAAGTGGTGCAGGAACAGGATGAACAGTATACCCACCCCTTGCAAGAGGAATTGCCCACGCCGGAGCATCAGTAGATCTAGCCATCTTCTCATTACACTT
AGCAGGAGTATCATCCATCCTAGGAGCAGTAAACTTCATCTCAACAACAATCAACATGAAACCAAAAAACATAAAACCCGAACGAATCCCCTTATTCGTATGATCAGTTGCCAT
CACGGCTCTCCTACTCCTCCTATCACTACCAGTACTAGCAGGAGCAATCACAATACTATTAACTGACCGAAACCTAAACACATCCTTCTTTGATCCAGCAGGAGGTGGAGACCC
AATCCTATACCAACACTTATTCTGATTCTTCGGACACCCAGAAGTATATATTTTAATCCTACCAGGATTTGGTATAATTTCCCACATTATTTGTCACGAAAGAGGTAAAAAGGAA
GCCTTCGGAAACCTAGGAATAATTTTCGCCATATTAGCAATCGGATTACTAGGATTTGTAGTATGAGCACATCATATGTTCACAGTAGGAATAGACGTTGACACACGAGCTTAC
TTTACATCAGCAACAATAATCATTGCAGTACCTACGGGGATTAAAATCTTCAGATGACTTGCAACAATATACGGAACCCGAATAACTTATAGAGCAGCATGCCTATGAGCCCTA
GGATTTGTA--

>G1_Macrotermes_subhyalinus_123 Macrotermes subhyalinus isolate TM01 cytochrome oxidase subunit I (COI) gene, partial cds; mitochondrial

----

AACAGAACTTGGACAACCAGGATCCTTAATCGGGGACGACCAAATCTACAACGTCATCGTCACAGCTCACGCTTTCGTAATGATCTTCTTCATAGTAATACCAATTATGATTGG

AGGATTCGGAAACTGACTAGTACCACTAATATTAGGAGCACCAGACATAGCATTCCCACGAATAAACAACATAAGATTCTGATTATTACCACCATCATTAACTCTTCTTCTCACT

AGTAGAACAGTAGAAAGTGGTGCAGGAACAGGATGAACAGTATACCCACCCCTTGCAAGAGGAATTGCCCACGCCGGAGCATCAGTAGATCTAGCCATCTTCTCATTACACTT

AGCAGGAGTATCATCCATCCTAGGAGCAGTAAACTTCATCTCAACAACAATCAACATGAAACCAAAAAACATAAAACCCGAACGAATCCCCTTATTCGTATGATCAGTTGCCAT

CACGGCTCTCCTACTCCTCCTATCACTACCAGTACTAGCAGGAGCAATCACAATACTATTAACTGACCGAAACCTAAACACATCCTTCTTTGATCCAGCAGGAGGTGGAGACCC

AATCCTATACCAACACTTATTCTGATTCTTCGGACACCCAGAAGTATATATTTTAATCCTACCAGGATTTGGTATAATTTCCCACATTATTTGTCACGAAAGAGGTAAAAAGGAA

GCCTTCGGAAACCTAGGAATAATTTTCGCCATATTAGCAATCGGATTACTAGGATTTGTAGTATGAGCACATCATATGTTCACAGTAGGAATAGACGTTGACACACGAGCTTAC

TTTACATCAGCAACAATAATCATTGCAGTACCTACGGGGATTAAAATCTTCAGATGACTTGCAACAATATACGGAACCCGAATAACTTATAGAGCAGCATGCCTATGAGCCCTA

GGATTTGTA--

>G1_Macrotermes_subhyalinus_124 Macrotermes subhyalinus isolate TM10 cytochrome oxidase subunit I (COI) gene, partial cds; mitochondrial

----

AACAGAACTTGGACAACCAGGATCCTTAATCGGGGACGACCAAATCTACAACGTCATCGTCACAGCTCACGCTTTCGTAATGATCTTCTTCATAGTAATACCAATTATGATTGG

AGGATTCGGAAACTGACTAGTACCACTAATATTAGGAGCACCAGACATAGCATTCCCACGAATAAACAACATAAGATTCTGATTATTACCACCATCATTAACTCTTCTTCTCACT

AGTAGAACAGTAGAAAGTGGTGCAGGAACAGGATGAACAGTATACCCACCCCTTGCAAGAGGAATTGCCCACGCCGGAGCATCAGTAGATCTAGCCATCTTCTCATTACACTT

AGCAGGAGTATCATCCATCCTAGGAGCAGTAAACTTCATCTCAACAACAATCAACATGAAACCAAAAAACATAAAACCCGAACGAATCCCCTTATTCGTATGATCAGTTGCCAT

CACGGCTCTCCTACTCCTCCTATCACTACCAGTACTAGCAGGAGCAATCACAATACTATTAACTGACCGAAACCTAAACACATCCTTCTTTGATCCAGCAGGAGGTGGAGACCC

AATCCTATACCAACACTTATTCTGATTCTTCGGACACCCAGAAGTATATATTTTAATCCTACCAGGATTTGGTATAATTTCCCACATTATTTGTCACGAAAGAGGTAAAAAGGAA

GCCTTCGGAAACCTAGGAATAATTTTCGCCATATTAGCAATCGGATTACTAGGATTTGTAGTATGAGCACATCATATGTTCACAGTAGGAATAGACGTTGACACACGAGCTTAC

TTTACATCAGCAACAATAATCATTGCAGTACCTACGGGGATTAAAATCTTCAGATGACTTGCAACAATATACGGAACCCGAATAACTTATAGAGCAGCATGCCTATGAGCCCTA
GGATTTGTA--

>G1_Macrotermes_subhyalinus_125 Macrotermes subhyalinus isolate TM16 cytochrome oxidase subunit I (COI) gene, partial cds; mitochondrial

----
AACAGAACTTGGACAACCAGGATCCTTAATCGGGGACGACCAAATCTACAACGTCATCGTCACAGCTCACGCTTTCGTAATGATCTTCTTCATAGTAATACCAATTATGATTGG
AGGATTCGGAAACTGACTAGTACCACTAATATTAGGAGCACCAGACATAGCATTCCCACGAATAAACAACATAAGATTCTGATTATTACCACCATCATTAACTCTTCTTCTCACT
AGTAGAACAGTAGAAAGTGGTGCAGGAACAGGATGAACAGTATACCCACCCCTTGCAAGAGGAATTGCCCACGCCGGAGCATCAGTAGATCTAGCCATCTTCTCATTACACTT
AGCAGGAGTATCATCCATCCTAGGAGCAGTAAACTTCATCTCAACAACAATCAACATGAAACCAAAAAACATAAAACCCGAACGAATCCCCTTATTCGTATGATCAGTTGCCAT
CACGGCTCTCCTACTCCTCCTATCACTACCAGTACTAGCAGGAGCAATCACAATACTATTAACTGACCGAAACCTAAACACATCCTTCTTTGATCCAGCAGGAGGTGGAGACCC
AATCCTATACCAACACTTATTCTGATTCTTCGGACACCCAGAAGTATATATTTTAATCCTACCAGGATTTGGTATAATTTCCCACATTATTTGTCACGAAAGAGGTAAAAAGGAA
GCCTTCGGAAACCTAGGAATAATTTTCGCCATATTAGCAATCGGATTACTAGGATTTGTAGTATGAGCACATCATATGTTCACAGTAGGAATAGACGTTGACACACGAGCTTAC
TTTACATCAGCAACAATAATCATTGCAGTACCTACGGGGATTAAAATCTTCAGATGACTTGCAACAATATACGGAACCCGAATAACTTATAGAGCAGCATGCCTATGAGCCCTA
GGATTTGTA--

>G1_Macrotermes_subhyalinus_126 Macrotermes subhyalinus isolate TM19 cytochrome oxidase subunit I (COI) gene, partial cds; mitochondrial

----

AACAGAACTTGGACAACCAGGATCCTTAATCGGGGACGACCAAATCTACAACGTCATCGTCACAGCTCACGCTTTCGTAATGATCTTCTTCATAGTAATACCAATTATGATTGG

AGGATTCGGAAACTGACTAGTACCACTAATATTAGGAGCACCAGACATAGCATTCCCACGAATAAACAACATAAGATTCTGATTATTACCACCATCATTAACTCTTCTTCTCACT

AGTAGAACAGTAGAAAGTGGTGCAGGAACAGGATGAACAGTATACCCACCCCTTGCAAGAGGAATTGCCCACGCCGGAGCATCAGTAGATCTAGCCATCTTCTCATTACACTT

AGCAGGAGTATCATCCATCCTAGGAGCAGTAAACTTCATCTCAACAACAATCAACATGAAACCAAAAAACATAAAACCCGAACGAATCCCCTTATTCGTATGATCAGTTGCCAT

CACGGCTCTCCTACTCCTCCTATCACTACCAGTACTAGCAGGAGCAATCACAATACTATTAACTGACCGAAACCTAAACACATCCTTCTTTGATCCAGCAGGAGGTGGAGACCC

AATCCTATACCAACACTTATTCTGATTCTTCGGACACCCAGAAGTATATATTTTAATCCTACCAGGATTTGGTATAATTTCCCACATTATTTGTCACGAAAGAGGTAAAAAGGAA

GCCTTCGGAAACCTAGGAATAATTTTCGCCATATTAGCAATCGGATTACTAGGATTTGTAGTATGAGCACATCATATGTTCACAGTAGGAATAGACGTTGACACACGAGCTTAC

TTTACATCAGCAACAATAATCATTGCAGTACCTACGGGGATTAAAATCTTCAGATGACTTGCAACAATATACGGAACCCGAATAACTTATAGAGCAGCATGCCTATGAGCCCTA

GGATTTGTA--

>G1_Macrotermes_subhyalinus_127 Macrotermes subhyalinus isolate TM39 cytochrome oxidase subunit I (COI) gene, partial cds; mitochondrial

----

AACAGAACTTGGACAACCAGGATCCTTAATCGGGGACGACCAAATCTACAACGTCATCGTCACAGCTCACGCTTTCGTAATGATCTTCTTCATAGTAATACCAATTATGATTGG

AGGATTCGGAAACTGACTAGTACCACTAATATTAGGAGCACCAGACATAGCATTCCCACGAATAAACAACATAAGATTCTGATTATTACCACCATCATTAACTCTTCTTCTCACT

AGTAGAACAGTAGAAAGTGGTGCAGGAACAGGATGAACAGTATACCCACCCCTTGCAAGAGGAATTGCCCACGCCGGAGCATCAGTAGATCTAGCCATCTTCTCATTACACTT

AGCAGGAGTATCATCCATCCTAGGAGCAGTAAACTTCATCTCAACAACAATCAACATGAAACCAAAAAACATAAAACCCGAACGAATCCCCTTATTCGTATGATCAGTTGCCAT

CACGGCTCTCCTACTCCTCCTATCACTACCAGTACTAGCAGGAGCAATCACAATACTATTAACTGACCGAAACCTAAACACATCCTTCTTTGATCCAGCAGGAGGTGGAGACCC

AATCCTATACCAACACTTATTCTGATTCTTCGGACACCCAGAAGTATATATTTTAATCCTACCAGGATTTGGTATAATTTCCCACATTATTTGTCACGAAAGAGGTAAAAAGGAA

GCCTTCGGAAACCTAGGAATAATTTTCGCCATATTAGCAATCGGATTACTAGGATTTGTAGTATGAGCACATCATATGTTCACAGTAGGAATAGACGTTGACACACGAGCTTAC

TTTACATCAGCAACAATAATCATTGCAGTACCTACGGGGATTAAAATCTTCAGATGACTTGCAACAATATACGGAACCCGAATAACTTATAGAGCAGCATGCCTATGAGCCCTA
GGATTTGTA--

>G1_Macrotermes_subhyalinus_128 Macrotermes subhyalinus isolate TB05 cytochrome oxidase subunit I (COI) gene, partial cds; mitochondrial

----
AACAGAACTTGGACAACCAGGATCCTTAATCGGGGACGACCAAATCTACAACGTCATCGTCACAGCTCACGCTTTCGTAATGATCTTCTTCATAGTAATACCAATTATGATTGG
AGGATTCGGAAACTGACTAGTACCACTAATATTAGGAGCACCAGACATAGCATTCCCACGAATAAACAACATAAGATTCTGATTATTACCACCATCATTAACTCTTCTTCTCACT
AGTAGAACAGTAGAAAGTGGTGCAGGAACAGGATGAACAGTATACCCACCCCTTGCAAGAGGAATTGCCCACGCCGGAGCATCAGTAGATCTAGCCATCTTCTCATTACACTT
AGCAGGAGTATCATCCATCCTAGGAGCAGTAAACTTCATCTCAACAACAATCAACATGAAACCAAAAAACATAAAACCCGAACGAATCCCCTTATTCGTATGATCAGTTGCCAT
CACGGCTCTCCTACTCCTCCTATCACTACCAGTACTAGCAGGAGCAATCACAATACTATTAACTGACCGAAACCTAAACACATCCTTCTTTGATCCAGCAGGAGGTGGAGACCC
AATCCTATACCAACACTTATTCTGATTCTTCGGACACCCAGAAGTATATATTTTAATCCTACCAGGATTTGGTATAATTTCCCACATTATTTGTCACGAAAGAGGTAAAAAGGAA
GCCTTCGGAAACCTAGGAATAATTTTCGCCATATTAGCAATCGGATTACTAGGATTTGTAGTATGAGCACATCATATGTTCACAGTAGGAATAGACGTTGACACACGAGCTTAC
TTTACATCAGCAACAATAATCATTGCAGTACCTACGGGGATTAAAATCTTCAGATGACTTGCAACAATATACGGAACCCGAATAACTTATAGAGCAGCATGCCTATGAGCCCTA
GGATTTGTA--

>G1_Macrotermes_subhyalinus_129 Macrotermes subhyalinus isolate TB06 cytochrome oxidase subunit I (COI) gene, partial cds; mitochondrial

----

AACAGAACTTGGACAACCAGGATCCTTAATCGGGGACGACCAAATCTACAACGTCATCGTCACAGCTCACGCTTTCGTAATGATCTTCTTCATAGTAATACCAATTATGATTGG
AGGATTCGGAAACTGACTAGTACCACTAATATTAGGAGCACCAGACATAGCATTCCCACGAATAAACAACATAAGATTCTGATTATTACCACCATCATTAACTCTTCTTCTCACT
AGTAGAACAGTAGAAAGTGGTGCAGGAACAGGATGAACAGTATACCCACCCCTTGCAAGAGGAATTGCCCACGCCGGAGCATCAGTAGATCTAGCCATCTTCTCATTACACTT
AGCAGGAGTATCATCCATCCTAGGAGCAGTAAACTTCATCTCAACAACAATCAACATGAAACCAAAAAACATAAAACCCGAACGAATCCCCTTATTCGTATGATCAGTTGCCAT
CACGGCTCTCCTACTCCTCCTATCACTACCAGTACTAGCAGGAGCAATCACAATACTATTAACTGACCGAAACCTAAACACATCCTTCTTTGATCCAGCAGGAGGTGGAGACCC
AATCCTATACCAACACTTATTCTGATTCTTCGGACACCCAGAAGTATATATTTTAATCCTACCAGGATTTGGTATAATTTCCCACATTATTTGTCACGAAAGAGGTAAAAAGGAA
GCCTTCGGAAACCTAGGAATAATTTTCGCCATATTAGCAATCGGATTACTAGGATTTGTAGTATGAGCACATCATATGTTCACAGTAGGAATAGACGTTGACACACGAGCTTAC
TTTACATCAGCAACAATAATCATTGCAGTACCTACGGGGATTAAAATCTTCAGATGACTTGCAACAATATACGGAACCCGAATAACTTATAGAGCAGCATGCCTATGAGCCCTA
GGATTTGTA---

>G1_Macrotermes_subhyalinus_130 Macrotermes subhyalinus isolate TB07 cytochrome oxidase subunit I (COI) gene, partial cds; mitochondrial

----

AACAGAACTTGGACAACCAGGATCCTTAATCGGGGACGACCAAATCTACAACGTCATCGTCACAGCTCACGCTTTCGTAATGATCTTCTTCATAGTAATACCAATTATGATTGG
AGGATTCGGAAACTGACTAGTACCACTAATATTAGGAGCACCAGACATAGCATTCCCACGAATAAACAACATAAGATTCTGATTATTACCACCATCATTAACTCTTCTTCTCACT
AGTAGAACAGTAGAAAGTGGTGCAGGAACAGGATGAACAGTATACCCACCCCTTGCAAGAGGAATTGCCCACGCCGGAGCATCAGTAGATCTAGCCATCTTCTCATTACACTT
AGCAGGAGTATCATCCATCCTAGGAGCAGTAAACTTCATCTCAACAACAATCAACATGAAACCAAAAAACATAAAACCCGAACGAATCCCCTTATTCGTATGATCAGTTGCCAT
CACGGCTCTCCTACTCCTCCTATCACTACCAGTACTAGCAGGAGCAATCACAATACTATTAACTGACCGAAACCTAAACACATCCTTCTTTGATCCAGCAGGAGGTGGAGACCC
AATCCTATACCAACACTTATTCTGATTCTTCGGACACCCAGAAGTATATATTTTAATCCTACCAGGATTTGGTATAATTTCCCACATTATTTGTCACGAAAGAGGTAAAAAGGAA
GCCTTCGGAAACCTAGGAATAATTTTCGCCATATTAGCAATCGGATTACTAGGATTTGTAGTATGAGCACATCATATGTTCACAGTAGGAATAGACGTTGACACACGAGCTTAC

TTTACATCAGCAACAATAATCATTGCAGTACCTACGGGGATTAAAATCTTCAGATGACTTGCAACAATATACGGAACCCGAATAACTTATAGAGCAGCATGCCTATGAGCCCTA

GGATTTGTA--

>G1_Macrotermes_subhyalinus_131 Macrotermes subhyalinus isolate TB18 cytochrome oxidase subunit I (COI) gene, partial cds; mitochondrial

----

AACAGAACTTGGACAACCAGGATCCTTAATCGGGGACGACCAAATCTACAACGTCATCGTCACAGCTCACGCTTTCGTAATGATCTTCTTCATAGTAATACCAATTATGATTGG

AGGATTCGGAAACTGACTAGTACCACTAATATTAGGAGCACCAGACATAGCATTCCCACGAATAAACAACATAAGATTCTGATTATTACCACCATCATTAACTCTTCTTCTCACT

AGTAGAACAGTAGAAAGTGGTGCAGGAACAGGATGAACAGTATACCCACCCCTTGCAAGAGGAATTGCCCACGCCGGAGCATCAGTAGATCTAGCCATCTTCTCATTACACTT

AGCAGGAGTATCATCCATCCTAGGAGCAGTAAACTTCATCTCAACAACAATCAACATGAAACCAAAAAACATAAAACCCGAACGAATCCCCTTATTCGTATGATCAGTTGCCAT

CACGGCTCTCCTACTCCTCCTATCACTACCAGTACTAGCAGGAGCAATCACAATACTATTAACTGACCGAAACCTAAACACATCCTTCTTTGATCCAGCAGGAGGTGGAGACCC

AATCCTATACCAACACTTATTCTGATTCTTCGGACACCCAGAAGTATATATTTTAATCCTACCAGGATTTGGTATAATTTCCCACATTATTTGTCACGAAAGAGGTAAAAAGGAA

GCCTTCGGAAACCTAGGAATAATTTTCGCCATATTAGCAATCGGATTACTAGGATTTGTAGTATGAGCACATCATATGTTCACAGTAGGAATAGACGTTGACACACGAGCTTAC

TTTACATCAGCAACAATAATCATTGCAGTACCTACGGGGATTAAAATCTTCAGATGACTTGCAACAATATACGGAACCCGAATAACTTATAGAGCAGCATGCCTATGAGCCCTA

GGATTTGTA--

>G1_Macrotermes_subhyalinus_132 Macrotermes subhyalinus isolate TB26 cytochrome oxidase subunit I (COI) gene, partial cds; mitochondrial

----

AACAGAACTTGGACAACCAGGATCCTTAATCGGGGACGACCAAATCTACAACGTCATCGTCACAGCTCACGCTTTCGTAATGATCTTCTTCATAGTAATACCAATTATGATTGG

AGGATTCGGAAACTGACTAGTACCACTAATATTAGGAGCACCAGACATAGCATTCCCACGAATAAACAACATAAGATTCTGATTATTACCACCATCATTAACTCTTCTTCTCACT

AGTAGAACAGTAGAAAGTGGTGCAGGAACAGGATGAACAGTATACCCACCCCTTGCAAGAGGAATTGCCCACGCCGGAGCATCAGTAGATCTAGCCATCTTCTCATTACACTT

AGCAGGAGTATCATCCATCCTAGGAGCAGTAAACTTCATCTCAACAACAATCAACATGAAACCAAAAAACATAAAACCCGAACGAATCCCCTTATTCGTATGATCAGTTGCCAT

CACGGCTCTCCTACTCCTCCTATCACTACCAGTACTAGCAGGAGCAATCACAATACTATTAACTGACCGAAACCTAAACACATCCTTCTTTGATCCAGCAGGAGGTGGAGACCC

AATCCTATACCAACACTTATTCTGATTCTTCGGACACCCAGAAGTATATATTTTAATCCTACCAGGATTTGGTATAATTTCCCACATTATTTGTCACGAAAGAGGTAAAAAGGAA

GCCTTCGGAAACCTAGGAATAATTTTCGCCATATTAGCAATCGGATTACTAGGATTTGTAGTATGAGCACATCATATGTTCACAGTAGGAATAGACGTTGACACACGAGCTTAC

TTTACATCAGCAACAATAATCATTGCAGTACCTACGGGGATTAAAATCTTCAGATGACTTGCAACAATATACGGAACCCGAATAACTTATAGAGCAGCATGCCTATGAGCCCTA

GGATTTGTA--

>G1_Macrotermes_subhyalinus_133 Macrotermes subhyalinus isolate TFA18 cytochrome oxidase subunit I (COI) gene, partial cds; mitochondrial

----

AACAGAACTTGGACAACCAGGATCCTTAATCGGGGACGACCAAATCTACAACGTCATCGTCACAGCTCACGCTTTCGTAATGATCTTCTTCATAGTAATACCAATTATGATTGG

AGGATTCGGAAACTGACTAGTACCACTAATATTAGGAGCACCAGACATAGCATTCCCACGAATAAACAACATAAGATTCTGATTATTACCACCATCATTAACTCTTCTTCTCACT

AGTAGAACAGTAGAAAGTGGTGCAGGAACAGGATGAACAGTATACCCACCCCTTGCAAGAGGAATTGCCCACGCCGGAGCATCAGTAGATCTAGCCATCTTCTCATTACACTT

AGCAGGAGTATCATCCATCCTAGGAGCAGTAAACTTCATCTCAACAACAATCAACATGAAACCAAAAAACATAAAACCCGAACGAATCCCCTTATTCGTATGATCAGTTGCCAT

CACGGCTCTCCTACTCCTCCTATCACTACCAGTACTAGCAGGAGCAATCACAATACTATTAACTGACCGAAACCTAAACACATCCTTCTTTGATCCAGCAGGAGGTGGAGACCC

AATCCTATACCAACACTTATTCTGATTCTTCGGACACCCAGAAGTATATATTTTAATCCTACCAGGATTTGGTATAATTTCCCACATTATTTGTCACGAAAGAGGTAAAAAGGAA

GCCTTCGGAAACCTAGGAATAATTTTCGCCATATTAGCAATCGGATTACTAGGATTTGTAGTATGAGCACATCATATGTTCACAGTAGGAATAGACGTTGACACACGAGCTTAC

TTTACATCAGCAACAATAATCATTGCAGTACCTACGGGGATTAAAATCTTCAGATGACTTGCAACAATATACGGAACCCGAATAACTTATAGAGCAGCATGCCTATGAGCCCTA
GGATTTGTA--

>G1_Macrotermes_subhyalinus_134 Macrotermes subhyalinus isolate TFA48 cytochrome oxidase subunit I (COI) gene, partial cds; mitochondrial

----
AACAGAACTTGGACAACCAGGATCCTTAATCGGGGACGACCAAATCTACAACGTCATCGTCACAGCTCACGCTTTCGTAATGATCTTCTTCATAGTAATACCAATTATGATTGG
AGGATTCGGAAACTGACTAGTACCACTAATATTAGGAGCACCAGACATAGCATTCCCACGAATAAACAACATAAGATTCTGATTATTACCACCATCATTAACTCTTCTTCTCACT
AGTAGAACAGTAGAAAGTGGTGCAGGAACAGGATGAACAGTATACCCACCCCTTGCAAGAGGAATTGCCCACGCCGGAGCATCAGTAGATCTAGCCATCTTCTCATTACACTT
AGCAGGAGTATCATCCATCCTAGGAGCAGTAAACTTCATCTCAACAACAATCAACATGAAACCAAAAAACATAAAACCCGAACGAATCCCCTTATTCGTATGATCAGTTGCCAT
CACGGCTCTCCTACTCCTCCTATCACTACCAGTACTAGCAGGAGCAATCACAATACTATTAACTGACCGAAACCTAAACACATCCTTCTTTGATCCAGCAGGAGGTGGAGACCC
AATCCTATACCAACACTTATTCTGATTCTTCGGACACCCAGAAGTATATATTTTAATCCTACCAGGATTTGGTATAATTTCCCACATTATTTGTCACGAAAGAGGTAAAAAGGAA
GCCTTCGGAAACCTAGGAATAATTTTCGCCATATTAGCAATCGGATTACTAGGATTTGTAGTATGAGCACATCATATGTTCACAGTAGGAATAGACGTTGACACACGAGCTTAC
TTTACATCAGCAACAATAATCATTGCAGTACCTACGGGGATTAAAATCTTCAGATGACTTGCAACAATATACGGAACCCGAATAACTTATAGAGCAGCATGCCTATGAGCCCTA
GGATTTGTA--

>G1_Macrotermes_subhyalinus_135 Macrotermes subhyalinus isolate TK13 cytochrome oxidase subunit I (COI) gene, partial cds; mitochondrial

----

AACAGAACTTGGACAACCAGGATCCTTAATCGGGGACGACCAAATCTACAACGTCATCGTCACAGCTCACGCTTTCGTAATGATCTTCTTCATAGTAATACCAATTATGATTGG

AGGATTCGGAAACTGACTAGTACCACTAATATTAGGAGCACCAGACATAGCATTCCCACGAATAAACAACATAAGATTCTGATTATTACCACCATCATTAACTCTTCTTCTCACT

AGTAGAACAGTAGAAAGTGGTGCAGGAACAGGATGAACAGTATACCCACCCCTTGCAAGAGGAATTGCTCACGCCGGAGCATCAGTAGATCTAGCCATCTTCTCATTACACTT

AGCAGGAGTATCATCCATCCTAGGAGCAGTAAACTTCATCTCAACAACAATCAACATGAAACCAAAAAACATAAAACCCGAACGAATCCCCTTATTCGTATGATCAGTTGCCAT

CACGGCTCTCCTACTCCTCCTATCACTACCAGTACTAGCAGGAGCAATCACAATACTATTAACTGACCGAAACCTAAACACATCCTTCTTTGATCCAGCAGGAGGTGGAGACCC

AATCCTATACCAACACTTATTCTGATTCTTCGGACACCCAGAAGTATATATTTTAATCCTACCAGGATTTGGTATAATTTCCCACATTATTTGTCACGAAAGAGGTAAAAAGGAA

GCCTTCGGAAACCTAGGAATAATTTTCGCCATATTAGCAATCGGATTACTAGGATTTGTAGTATGAGCACATCATATGTTCACAGTAGGAATAGACGTTGACACACGAGCTTAC

TTTACATCAGCAACAATAATCATTGCAGTACCTACGGGGATTAAAATCTTCAGATGACTTGCAACAATATACGGAACCCGAATAACTTATAGAGCAGCATGCCTATGAGCCCTA

GGATTTGTA--

>G1_Macrotermes_subhyalinus_136 Macrotermes subhyalinus isolate TK15 cytochrome oxidase subunit I (COI) gene, partial cds; mitochondrial

----

AACAGAACTTGGACAACCAGGATCCTTAATCGGGGACGACCAAATCTACAACGTCATCGTCACAGCTCACGCTTTCGTAATGATCTTCTTCATAGTAATACCAATTATGATTGG

AGGATTCGGAAACTGACTAGTACCACTAATATTAGGAGCACCAGACATAGCATTCCCACGAATAAACAACATAAGATTCTGATTATTACCACCATCATTAACTCTTCTTCTCACT

AGTAGAACAGTAGAAAGTGGTGCAGGAACAGGATGAACAGTATACCCACCCCTTGCAAGAGGAATTGCTCACGCCGGAGCATCAGTAGATCTAGCCATCTTCTCATTACACTT

AGCAGGAGTATCATCCATCCTAGGAGCAGTAAACTTCATCTCAACAACAATCAACATGAAACCAAAAAACATAAAACCCGAACGAATCCCCTTATTCGTATGATCAGTTGCCAT

CACGGCTCTCCTACTCCTCCTATCACTACCAGTACTAGCAGGAGCAATCACAATACTATTAACTGACCGAAACCTAAACACATCCTTCTTTGATCCAGCAGGAGGTGGAGACCC

AATCCTATACCAACACTTATTCTGATTCTTCGGACACCCAGAAGTATATATTTTAATCCTACCAGGATTTGGTATAATTTCCCACATTATTTGTCACGAAAGAGGTAAAAAGGAA

GCCTTCGGAAACCTAGGAATAATTTTCGCCATATTAGCAATCGGATTACTAGGATTTGTAGTATGAGCACATCATATGTTCACAGTAGGAATAGACGTTGACACACGAGCTTAC

TTTACATCAGCAACAATAATCATTGCAGTACCTACGGGGATTAAAATCTTCAGATGACTTGCAACAATATACGGAACCCGAATAACTTATAGAGCAGCATGCCTATGAGCCCTA

GGATTTGTA---

>G1_Macrotermes_subhyalinus_137 Macrotermes subhyalinus isolate TK16 cytochrome oxidase subunit I (COI) gene, partial cds; mitochondrial

----

AACAGAACTTGGACAACCAGGATCCTTAATCGGGGACGACCAAATCTACAACGTCATCGTCACAGCTCACGCTTTCGTAATGATCTTCTTCATAGTAATACCAATTATGATTGG

AGGATTCGGAAACTGACTAGTACCACTAATATTAGGAGCACCAGACATAGCATTCCCACGAATAAACAACATAAGATTCTGATTATTACCACCATCATTAACTCTTCTTCTCACT

AGTAGAACAGTAGAAAGTGGTGCAGGAACAGGATGAACAGTATACCCACCCCTTGCAAGAGGAATTGCTCACGCCGGAGCATCAGTAGATCTAGCCATCTTCTCATTACACTT

AGCAGGAGTATCATCCATCCTAGGAGCAGTAAACTTCATCTCAACAACAATCAACATGAAACCAAAAAACATAAAACCCGAACGAATCCCCTTATTCGTATGATCAGTTGCCAT

CACGGCTCTCCTACTCCTCCTATCACTACCAGTACTAGCAGGAGCAATCACAATACTATTAACTGACCGAAACCTAAACACATCCTTCTTTGATCCAGCAGGAGGTGGAGACCC

AATCCTATACCAACACTTATTCTGATTCTTCGGACACCCAGAAGTATATATTTTAATCCTACCAGGATTTGGTATAATTTCCCACATTATTTGTCACGAAAGAGGTAAAAAGGAA

GCCTTCGGAAACCTAGGAATAATTTTCGCCATATTAGCAATCGGATTACTAGGATTTGTAGTATGAGCACATCATATGTTCACAGTAGGAATAGACGTTGACACACGAGCTTAC

TTTACATCAGCAACAATAATCATTGCAGTACCTACGGGGATTAAAATCTTCAGATGACTTGCAACAATATACGGAACCCGAATAACTTATAGAGCAGCATGCCTATGAGCCCTA

GGATTTGTA---

>G1_Macrotermes_subhyalinus_138 Macrotermes subhyalinus isolate TY11 cytochrome oxidase subunit I (COI) gene, partial cds; mitochondrial

----

AACAGAACTTGGACAACCAGGATCCTTAATCGGGGACGACCAAATCTACAACGTCATCGTCACAGCTCACGCTTTCGTAATGATCTTCTTCATAGTAATACCAATTATGATTGG
AGGATTCGGAAACTGACTAGTACCACTAATATTAGGAGCACCAGACATAGCATTCCCACGAATAAACAACATAAGATTCTGATTATTACCACCATCATTAACTCTTCTTCTCACT
AGTAGAACAGTAGAAAGTGGTGCAGGAACAGGATGAACAGTATACCCACCCCTTGCAAGAGGAATTGCCCACGCCGGAGCATCAGTAGATCTAGCCATCTTCTCATTACACTT
AGCAGGAGTATCATCCATCCTAGGAGCAGTAAACTTCATCTCAACAACAATCAACATGAAACCAAAAAACATAAAACCCGAACGAATCCCCTTATTCGTATGATCAGTTGCCAT
CACGGCTCTCCTACTCCTCCTATCACTACCAGTACTAGCAGGAGCAATCACAATACTATTAACTGACCGAAACCTAAACACATCCTTCTTTGATCCAGCAGGAGGTGGAGACCC
AATCCTATACCAACACTTATTCTGATTCTTCGGACACCCCGAAGTATATATTTTAATCCTACCAGGATTTGGTATAATTTCCCACATTATTTGTCACGAAAGAGGTAAAAAGGAA
GCCTTCGGAAACCTAGGAATAATTTTCGCCATATTAGCAATCGGATTACTAGGATTTGTAGTATGAGCACACCATATGTTCACAGTAGGAATAGACGTTGACACACGAGCTTAC
TTTACATCAGCAACAATAATCATTGCAGTACCTACGGGGATTAAAATCTTCAGATGACTTGCAACAATATACGGAACCCGAATAACTTATAGAGCAGCATGCCTATGAGCCCTA
GGATTTGTA---

>G1_Macrotermes_subhyalinus_139 Macrotermes subhyalinus isolate TY12 cytochrome oxidase subunit I (COI) gene, partial cds; mitochondrial

----

AACAGAACTTGGACAACCAGGATCCTTAATCGGGGACGACCAAATCTACAACGTCATCGTCACAGCTCACGCTTTCGTAATGATCTTCTTCATAGTAATACCAATTATGATTGG
AGGATTCGGAAACTGACTAGTACCACTAATATTAGGAGCACCAGACATAGCATTCCCACGAATAAACAACATAAGATTCTGATTATTACCACCATCATTAACTCTTCTTCTCACT
AGTAGAACAGTAGAAAGTGGTGCAGGAACAGGATGAACAGTATACCCACCCCTTGCAAGAGGAATTGCCCACGCCGGAGCATCAGTAGATCTAGCCATCTTCTCATTACACTT
AGCAGGAGTATCATCCATCCTAGGAGCAGTAAACTTCATCTCAACAACAATCAACATGAAACCAAAAAACATAAAACCCGAACGAATCCCCTTATTCGTATGATCAGTTGCCAT
CACGGCTCTCCTACTCCTCCTATCACTACCAGTACTAGCAGGAGCAATCACAATACTATTAACTGACCGAAACCTAAACACATCCTTCTTTGATCCAGCAGGAGGTGGAGACCC
AATCCTATACCAACACTTATTCTGATTCTTCGGACACCCCGAAGTATATATTTTAATCCTACCAGGATTTGGTATAATTTCCCACATTATTTGTCACGAAAGAGGTAAAAAGGAA
GCCTTCGGAAACCTAGGAATAATTTTCGCCATATTAGCAATCGGATTACTAGGATTTGTAGTATGAGCACACCATATGTTCACAGTAGGAATAGACGTTGACACACGAGCTTAC

TTTACATCAGCAACAATAATCATTGCAGTACCTACGGGGATTAAAATCTTCAGATGACTTGCAACAATATACGGAACCCGAATAACTTATAGAGCAGCATGCCTATGAGCCCTA

GGATTTGTA--

>G1_Macrotermes_subhyalinus_140 Macrotermes subhyalinus isolate TA01 cytochrome oxidase subunit I (COI) gene, partial cds; mitochondrial

----

AACAGAACTTGGACAACCAGGATCCTTAATCGGGGACGACCAAATCTACAACGTCATCGTCACAGCTCACGCTTTCGTAATGATCTTCTTCATAGTAATACCAATTATGATTGG

AGGATTCGGAAACTGACTAGTACCACTAATATTAGGAGCACCAGACATAGCATTCCCACGAATAAACAACATAAGATTCTGATTATTACCACCATCATTAACTCTTCTTCTCACT

AGTAGAACAGTAGAAAGTGGTGCAGGAACAGGATGAACAGTATACCCACCCCTTGCAAGAGGAATTGCCCACGCCGGAGCATCAGTAGATCTAGCCATCTTCTCATTACACTT

AGCAGGAGTATCATCCATCCTAGGAGCAGTAAACTTCATCTCAACAACAATCAACATGAAACCAAAAAACATAAAACCCGAACGAATCCCCTTATTCGTATGATCAGTTGCCAT

CACGGCTCTCCTACTCCTCCTATCACTACCAGTACTAGCAGGAGCAATCACAATACTATTAACTGACCGAAACCTAAACACATCCTTCTTTGATCCAGCAGGAGGTGGAGACCC

AATCCTATACCAACACTTATTCTGATTCTTCGGACACCCCGAAGTATATATTTTAATCCTACCAGGATTTGGTATAATTTCCCACATTATTTGTCACGAAAGAGGTAAAAAGGAA

GCCTTCGGAAACCTAGGAATAATTTTCGCCATATTAGCAATCGGATTACTAGGATTTGTAGTATGAGCACACCATATGTTCACAGTAGGAATAGACGTTGACACACGAGCTTAC

TTTACATCAGCAACAATAATCATTGCAGTACCTACGGGGATTAAAATCTTCAGATGACTTGCAACAATATACGGAACCCGAATAACTTATAGAGCAGCATGCCTATGAGCCCTA

GGATTTGTA--

>G1_Macrotermes_subhyalinus_141 Macrotermes subhyalinus isolate TA05 cytochrome oxidase subunit I (COI) gene, partial cds; mitochondrial

----

AACAGAACTTGGACAACCAGGATCCTTAATCGGGGACGACCAAATCTACAACGTCATCGTCACAGCTCACGCTTTCGTAATGATCTTCTTCATAGTAATACCAATTATGATTGG

AGGATTCGGAAACTGACTAGTACCACTAATATTAGGAGCACCAGACATAGCATTCCCACGAATAAACAACATAAGATTCTGATTATTACCACCATCATTAACTCTTCTTCTCACT

AGTAGAACAGTAGAAAGTGGTGCAGGAACAGGATGAACAGTATACCCACCCCTTGCAAGAGGAATTGCCCACGCCGGAGCATCAGTAGATCTAGCCATCTTCTCATTACACTT

AGCAGGAGTATCATCCATCCTAGGAGCAGTAAACTTCATCTCAACAACAATCAACATGAAACCAAAAAACATAAAACCCGAACGAATCCCCTTATTCGTATGATCAGTTGCCAT

CACGGCTCTCCTACTCCTCCTATCACTACCAGTACTAGCAGGAGCAATCACAATACTATTAACTGACCGAAACCTAAACACATCCTTCTTTGATCCAGCAGGAGGTGGAGACCC

AATCCTATACCAACACTTATTCTGATTCTTCGGACACCCCGAAGTATATATTTTAATCCTACCAGGATTTGGTATAATTTCCCACATTATTTGTCACGAAAGAGGTAAAAAGGAA

GCCTTCGGAAACCTAGGAATAATTTTCGCCATATTAGCAATCGGATTACTAGGATTTGTAGTATGAGCACACCATATGTTCACAGTAGGAATAGACGTTGACACACGAGCTTAC

TTTACATCAGCAACAATAATCATTGCAGTACCTACGGGGATTAAAATCTTCAGATGACTTGCAACAATATACGGAACCCGAATAACTTATAGAGCAGCATGCCTATGAGCCCTA

GGATTTGTA--

>G1_Macrotermes_subhyalinus_142 Macrotermes subhyalinus isolate TA22 cytochrome oxidase subunit I (COI) gene, partial cds; mitochondrial

----

AACAGAACTTGGACAACCAGGATCCTTAATCGGGGACGACCAAATCTACAACGTCATCGTCACAGCTCACGCTTTCGTAATGATCTTCTTCATAGTAATACCAATTATGATTGG

AGGATTCGGAAACTGACTAGTACCACTAATATTAGGAGCACCAGACATAGCATTCCCACGAATAAACAACATAAGATTCTGATTATTACCACCATCATTAACTCTTCTTCTCACT

AGTAGAACAGTAGAAAGTGGTGCAGGAACAGGATGAACAGTATACCCACCCCTTGCAAGAGGAATTGCCCACGCCGGAGCATCAGTAGATCTAGCCATCTTCTCATTACACTT

AGCAGGAGTATCATCCATCCTAGGAGCAGTAAACTTCATCTCAACAACAATCAACATGAAACCAAAAAACATAAAACCCGAACGAATCCCCTTATTCGTATGATCAGTTGCCAT

CACGGCTCTCCTACTCCTCCTATCACTACCAGTACTAGCAGGAGCAATCACAATACTATTAACTGACCGAAACCTAAACACATCCTTCTTTGATCCAGCAGGAGGTGGAGACCC

AATCCTATACCAACACTTATTCTGATTCTTCGGACACCCCGAAGTATATATTTTAATCCTACCAGGATTTGGTATAATTTCCCACATTATTTGTCACGAAAGAGGTAAAAAGGAA

GCCTTCGGAAACCTAGGAATAATTTTCGCCATATTAGCAATCGGATTACTAGGATTTGTAGTATGAGCACACCATATGTTCACAGTAGGAATAGACGTTGACACACGAGCTTAC

TTTACATCAGCAACAATAATCATTGCAGTACCTACGGGGATTAAAATCTTCAGATGACTTGCAACAATATACGGAACCCGAATAACTTATAGAGCAGCATGCCTATGAGCCCTA

GGATTTGTA---

>G1_Macrotermes_subhyalinus_143 Macrotermes subhyalinus isolate TA31 cytochrome oxidase subunit I (COI) gene, partial cds; mitochondrial

----

AACAGAACTTGGACAACCAGGATCCTTAATCGGGGACGACCAAATCTACAACGTCATCGTCACAGCTCACGCTTTCGTAATGATCTTCTTCATAGTAATACCAATTATGATTGG

AGGATTCGGAAACTGACTAGTACCACTAATATTAGGAGCACCAGACATAGCATTCCCACGAATAAACAACATAAGATTCTGATTATTACCACCATCATTAACTCTTCTTCTCACT

AGTAGAACAGTAGAAAGTGGTGCAGGAACAGGATGAACAGTATACCCACCCCTTGCAAGAGGAATTGCCCACGCCGGAGCATCAGTAGATCTAGCCATCTTCTCATTACACTT

AGCAGGAGTATCATCCATCCTAGGAGCAGTAAACTTCATCTCAACAACAATCAACATGAAACCAAAAAACATAAAACCCGAACGAATCCCCTTATTCGTATGATCAGTTGCCAT

CACGGCTCTCCTACTCCTCCTATCACTACCAGTACTAGCAGGAGCAATCACAATACTATTAACTGACCGAAACCTAAACACATCCTTCTTTGATCCAGCAGGAGGTGGAGACCC

AATCCTATACCAACACTTATTCTGATTCTTCGGACACCCCGAAGTATATATTTTAATCCTACCAGGATTTGGTATAATTTCCCACATTATTTGTCACGAAAGAGGTAAAAAGGAA

GCCTTCGGAAACCTAGGAATAATTTTCGCCATATTAGCAATCGGATTACTAGGATTTGTAGTATGAGCACACCATATGTTCACAGTAGGAATAGACGTTGACACACGAGCTTAC

TTTACATCAGCAACAATAATCATTGCAGTACCTACGGGGATTAAAATCTTCAGATGACTTGCAACAATATACGGAACCCGAATAACTTATAGAGCAGCATGCCTATGAGCCCTA

GGATTTGTA---

>G1_Macrotermes_subhyalinus_144 Macrotermes subhyalinus isolate TA36 cytochrome oxidase subunit I (COI) gene, partial cds; mitochondrial

----

AACAGAACTTGGACAACCAGGATCCTTAATCGGGGACGACCAAATCTACAACGTCATCGTCACAGCTCACGCTTTCGTAATGATCTTCTTCATAGTAATACCAATTATGATTGG

AGGATTCGGAAACTGACTAGTACCACTAATATTAGGAGCACCAGACATAGCATTCCCACGAATAAACAACATAAGATTCTGATTATTACCACCATCATTAACTCTTCTTCTCACT

AGTAGAACAGTAGAAAGTGGTGCAGGAACAGGATGAACAGTATACCCACCCCTTGCAAGAGGAATTGCCCACGCCGGAGCATCAGTAGATCTAGCCATCTTCTCATTACACTT

AGCAGGAGTATCATCCATCCTAGGAGCAGTAAACTTCATCTCAACAACAATCAACATGAAACCAAAAAACATAAAACCCGAACGAATCCCCTTATTCGTATGATCAGTTGCCAT

CACGGCTCTCCTACTCCTCCTATCACTACCAGTACTAGCAGGAGCAATCACAATACTATTAACTGACCGAAACCTAAACACATCCTTCTTTGATCCAGCAGGAGGTGGAGACCC

AATCCTATACCAACACTTATTCTGATTCTTCGGACACCCCGAAGTATATATTTTAATCCTACCAGGATTTGGTATAATTTCCCACATTATTTGTCACGAAAGAGGTAAAAAGGAA

GCCTTCGGAAACCTAGGAATAATTTTCGCCATATTAGCAATCGGATTACTAGGATTTGTAGTATGAGCACACCATATGTTCACAGTAGGAATAGACGTTGACACACGAGCTTAC

TTTACATCAGCAACAATAATCATTGCAGTACCTACGGGGATTAAAATCTTCAGATGACTTGCAACAATATACGGAACCCGAATAACTTATAGAGCAGCATGCCTATGAGCCCTA

GGATTTGTA---

>G1_Macrotermes_subhyalinus_145 Macrotermes subhyalinus isolate TY04 cytochrome oxidase subunit I (COI) gene, partial cds; mitochondrial

----

AACAGAACTTGGACAACCAGGATCCTTAATCGGGGACGACCAAATCTACAACGTCATCGTCACAGCTCACGCTTTCGTAATGATCTTCTTCATAGTAATACCAATTATGATTGG

AGGATTCGGAAACTGACTAGTACCACTAATATTAGGAGCACCAGACATAGCATTCCCACGAATAAACAACATAAGATTCTGATTATTACCACCATCATTAACTCTTCTTCTCACT

AGTAGAACAGTAGAAAGTGGTGCAGGAACAGGATGAACAGTATACCCACCCCTTGCAAGAGGAATTGCCCACGCCGGAGCATCAGTAGATCTAGCCATCTTCTCATTACACTT

AGCAGGAGTATCATCCATCCTAGGAGCAGTAAACTTCATCTCAACAACAATCAACATGAAACCAAAAAACATAAAACCCGAACGAATCCCCTTATTCGTATGATCAGTTGCCAT

CACGGCTCTCCTACTCCTCCTATCACTACCAGTACTAGCAGGAGCAATCACAATACTATTAACTGACCGAAACCTAAACACATCCTTCTTTGATCCAGCAGGAGGTGGAGACCC

AATCCTATACCAACACTTATTCTGATTCTTCGGACACCCCGAAGTATATATTTTAATCCTACCAGGATTTGGTATAATTTCCCACATTATTTGTCACGAAAGAGGTAAAAAGGAA

GCCTTCGGAAACCTAGGAATAATTTTCGCCATATTAGCAATCGGATTACTAGGATTTGTAGTATGAGCACACCATATGTTCACAGTAGGAATAGACGTTGACACACGAGCTTAC

TTTACATCAGCAACAATAATCATTGCAGTACCTACGGGGATTAAAATCTTCAGATGACTTGCAACAATATACGGAACCCGAATAACTTATAGAGCAGCATGCCTATGAGCCCTA

GGATTTGTA--

>G1_Macrotermes_subhyalinus_146 Macrotermes subhyalinus isolate TFA43 cytochrome oxidase subunit I (COI) gene, partial cds; mitochondrial

----

AACAGAACTTGGACAACCAGGATCCTTAATCGGGGACGACCAAATCTACAACGTCATCGTCACAGCTCACGCTTTCGTAATGATCTTCTTCATAGTAATACCAATTATGATTGG

AGGATTCGGAAACTGACTAGTACCACTAATATTAGGAGCACCAGACATAGCATTCCCACGAATAAACAACATAAGATTCTGATTATTACCACCATCATTAACTCTTCTTCTCACT

AGTAGAACAGTAGAAAGTGGTGCAGGAACAGGATGAACAGTATACCCACCCCTTGCAAGAGGAATTGCCCACGCCGGAGCATCAGTAGATCTAGCCATCTTCTCATTACACTT

AGCAGGAGTATCATCCATCCTAGGAGCAGTAAACTTCATCTCAACAACAATCAACATGAAACCAAAAAACATAAAACCCGAACGAATCCCCTTATTCGTATGATCAGTTGCCAT

CACGGCTCTCCTACTCCTCCTATCACTACCAGTACTAGCAGGAGCAATCACAATACTATTAACTGACCGAAACCTAAACACATCCTTCTTTGATCCAGCAGGAGGTGGAGACCC

AATCCTATACCAACACTTATTCTGATTCTTCGGACACCCAGAAGTATATATTTTAATCCTACCAGGATTTGGTATAATTTCCCACATTATTTGTCACGAAAGAGGTAAAAAGGAA

GCCTTCGGAAACCTAGGAATAATTTTCGCCATATTAGCAATCGGATTACTAGGATTTGTAGTATGAGCACACCATATGTTCACAGTAGGAATAGACGTTGACACACGAGCTTAC

TTTACATCAGCAACAATAATCATTGCAGTACCTACGGGGATTAAAATCTTCAGATGACTTGCAACAATATACGGAACCCGAATAACTTATAGAGCTGCATGCCTATGAGCCCTA

GGATTTGTA--

>G1_Macrotermes_subhyalinus_147 Macrotermes subhyalinus isolate TM04 cytochrome oxidase subunit I (COI) gene, partial cds; mitochondrial

----

AACAGAACTTGGACAACCAGGATCCTTAATTGGGGACGACCAAATCTACAACGTCATCGTCACAGCTCACGCTTTCGTAATGATCTTCTTCATAGTAATACCAATTATGATTGG

AGGATTCGGAAACTGACTAGTACCACTAATATTAGGAGCACCAGACATAGCATTCCCACGAATAAACAACATAAGATTCTGATTATTACCACCATCATTAACTCTTCTTCTCACT

AGTAGAACAGTAGAAAGTGGTGCAGGAACAGGATGAACAGTATACCCACCCCTTGCAAGAGGAATTGCCCACGCCGGAGCATCAGTAGATCTAGCCATCTTCTCATTACACTT

AGCAGGAGTATCATCCATCCTAGGAGCAGTAAACTTCATCTCAACAACAATCAACATGAAACCAAAAAACATAAAACCCGAACGAATCCCCTTATTCGTATGATCAGTTGCCAT

CACGGCTCTCCTACTCCTCCTATCACTACCAGTTCTAGCAGGAGCAATCACAATACTATTAACTGACCGAAACCTAAACACATCCTTCTTTGATCCAGCAGGAGGTGGAGACCCA

ATCCTATACCAACACTTATTCTGATTCTTCGGACACCCCGAAGTATATATTTTAATCCTACCAGGATTTGGTATAATTTCCCACATTATTTGTCACGAAAGAGGTAAAAAGGAAG

CCTTCGGAAACCTAGGAATAATTTTCGCCATATTAGCAATCGGATTACTAGGATTTGTAGTATGAGCACACCATATGTTCACAGTAGGAATAGACGTTGACACACGAGCTTACT

TTACATCAGCAACAATAATCATTGCAGTACCTACGGGGATTAAAATCTTCAGATGACTTGCAACAATATACGGAACCCGAATAACTTATAGAGCAGCATGCCTATGAGCCCTAG

GATTTGTA--

>G1_Macrotermes_subhyalinus_148 Macrotermes subhyalinus isolate TM14 cytochrome oxidase subunit I (COI) gene, partial cds; mitochondrial

----

AACAGAACTTGGACAACCAGGATCCTTAATTGGGGACGACCAAATCTACAACGTCATCGTCACAGCTCACGCTTTCGTAATGATCTTCTTCATAGTAATACCAATTATGATTGG

AGGATTCGGAAACTGACTAGTACCACTAATATTAGGAGCACCAGACATAGCATTCCCACGAATAAACAACATAAGATTCTGATTATTACCACCATCATTAACTCTTCTTCTCACT

AGTAGAACAGTAGAAAGTGGTGCAGGAACAGGATGAACAGTATACCCACCCCTTGCAAGAGGAATTGCCCACGCCGGAGCATCAGTAGATCTAGCCATCTTCTCATTACACTT

AGCAGGAGTATCATCCATCCTAGGAGCAGTAAACTTCATCTCAACAACAATCAACATGAAACCAAAAAACATAAAACCCGAACGAATCCCCTTATTCGTATGATCAGTTGCCAT

CACGGCTCTCCTACTCCTCCTATCACTACCAGTTCTAGCAGGAGCAATCACAATACTATTAACTGACCGAAACCTAAACACATCCTTCTTTGATCCAGCAGGAGGTGGAGACCCA

ATCCTATACCAACACTTATTCTGATTCTTCGGACACCCCGAAGTATATATTTTAATCCTACCAGGATTTGGTATAATTTCCCACATTATTTGTCACGAAAGAGGTAAAAAGGAAG

CCTTCGGAAACCTAGGAATAATTTTCGCCATATTAGCAATCGGATTACTAGGATTTGTAGTATGAGCACACCATATGTTCACAGTAGGAATAGACGTTGACACACGAGCTTACT

TTACATCAGCAACAATAATCATTGCAGTACCTACGGGGATTAAAATCTTCAGATGACTTGCAACAATATACGGAACCCGAATAACTTATAGAGCAGCATGCCTATGAGCCCTAG
GATTTGTA--

>G4_Macrotermes_michaelseni_1 Macrotermes michaelseni isolate TR149 cytochrome oxidase subunit I (COI) gene, partial cds; mitochondrial

----
AACAGAACTTGGACAACCAGGATCCTTAATTGGAGACGACCAAATCTACAACGTCATCGTCACAGCTCACGCTTTCGTAATGATCTTCTTCATAGTAATACCAATCATGATTGG
AGGATTCGGAAACTGACTAGTACCGCTAATATTAGGAGCACCAGACATAGCATTCCCACGAATAAACAACATAAGATTCTGATTATTACCACCATCATTAACTCTTCTTCTCACT
AGTAGAACAGTAGAAAGTGGTGCAGGAACAGGATGAACAGTATACCCACCCCTTGCAAGAGGAATTGCTCACGCCGGAGCATCAGTAGATCTAGCCATCTTCTCATTACACTT
AGCAGGAGTATCATCCATCCTAGGAGCAGTAAACTTCATTTCAACAACAATCAACATGAAACCAAAAAACATAAAACCCGAACGAATCCCATTATTCGTATGATCAGTTGCCAT
CACGGCTCTCCTACTCCTCCTATCACTACCAGTACTAGCAGGAGCAATCACAATACTATTAACTGACCGAAACCTAAACACATCCTTCTTCGATCCAGCAGGAGGTGGAGACCC
AATCCTATACCAACACTTATTCTGATTCTTCGGACACCCCGAAGTATATATTTTAATTCTACCAGGATTTGGTATAATTTCCCACATTATTTGTCACGAAAGAGGTAAAAAGGAA
GCCTTCGGAAACCTGGGAATAATTTTTGCCATATTAGCAATCGGATTACTAGGATTTGTAGTATGAGCACACCATATGTTCACAGTAGGAATAGACGTTGATACACGAGCTTAC
TTTACATCAGCAACAATAATCATTGCAGTACCTACGGGGATTAAAATCTTCAGATGACTTGCAACAATATACGGAACACGAATAACTTATAGAGCAGCATGCCTATGAGCCCTA
GGATTTGTA---

>G4_Macrotermes_michaelseni_2 Macrotermes michaelseni isolate TR159 cytochrome oxidase subunit I (COI) gene, partial cds; mitochondrial

----

AACAGAACTTGGACAACCAGGATCCTTAATTGGAGACGACCAAATCTACAACGTCATCGTCACAGCTCACGCTTTCGTAATGATCTTCTTCATAGTAATACCAATCATGATTGG

AGGATTCGGAAACTGACTAGTACCGCTAATATTAGGAGCACCAGACATAGCATTCCCACGAATAAACAACATAAGATTCTGATTATTACCACCATCATTAACTCTTCTTCTCACT

AGTAGAACAGTAGAAAGTGGTGCAGGAACAGGATGAACAGTATACCCACCCCTTGCAAGAGGAATTGCTCACGCCGGAGCATCAGTAGATCTAGCCATCTTCTCATTACACTT

AGCAGGAGTATCATCCATCCTAGGAGCAGTAAACTTCATTTCAACAACAATCAACATGAAACCAAAAAACATAAAACCCGAACGAATCCCATTATTCGTATGATCAGTTGCCAT

CACGGCTCTCCTACTCCTCCTATCACTACCAGTACTAGCAGGAGCAATCACAATACTATTAACTGACCGAAACCTAAACACATCCTTCTTCGATCCAGCAGGAGGTGGAGACCC

AATCCTATACCAACACTTATTCTGATTCTTCGGACACCCCGAAGTATATATTTTAATTCTACCAGGATTTGGTATAATTTCCCACATTATTTGTCACGAAAGAGGTAAAAAGGAA

GCCTTCGGAAACCTGGGAATAATTTTTGCCATATTAGCAATCGGATTACTAGGATTTGTAGTATGAGCACACCATATGTTCACAGTAGGAATAGACGTTGATACACGAGCTTAC

TTTACATCAGCAACAATAATCATTGCAGTACCTACGGGGATTAAAATCTTCAGATGACTTGCAACAATATACGGAACACGAATAACTTATAGAGCAGCATGCCTATGAGCCCTA

GGATTTGTA--

>G4_Macrotermes_michaelseni_3 Macrotermes michaelseni isolate TR160 cytochrome oxidase subunit I (COI) gene, partial cds; mitochondrial

----

AACAGAACTTGGACAACCAGGATCCTTAATTGGAGACGACCAAATCTACAACGTCATCGTCACAGCTCACGCTTTCGTAATGATCTTCTTCATAGTAATACCAATCATGATTGG

AGGATTCGGAAACTGACTAGTACCGCTAATATTAGGAGCACCAGACATAGCATTCCCACGAATAAACAACATAAGATTCTGATTATTACCACCATCATTAACTCTTCTTCTCACT

AGTAGAACAGTAGAAAGTGGTGCAGGAACAGGATGAACAGTATACCCACCCCTTGCAAGAGGAATTGCTCACGCCGGAGCATCAGTAGATCTAGCCATCTTCTCATTACACTT

AGCAGGAGTATCATCCATCCTAGGAGCAGTAAACTTCATTTCAACAACAATCAACATGAAACCAAAAAACATAAAACCCGAACGAATCCCATTATTCGTATGATCAGTTGCCAT

CACGGCTCTCCTACTCCTCCTATCACTACCAGTACTAGCAGGAGCAATCACAATACTATTAACTGACCGAAACCTAAACACATCCTTCTTCGATCCAGCAGGAGGTGGAGACCC

AATCCTATACCAACACTTATTCTGATTCTTCGGACACCCCGAAGTATATATTTTAATTCTACCAGGATTTGGTATAATTTCCCACATTATTTGTCACGAAAGAGGTAAAAAGGAA

GCCTTCGGAAACCTGGGAATAATTTTTGCCATATTAGCAATCGGATTACTAGGATTTGTAGTATGAGCACACCATATGTTCACAGTAGGAATAGACGTTGATACACGAGCTTAC

TTTACATCAGCAACAATAATCATTGCAGTACCTACGGGGATTAAAATCTTCAGATGACTTGCAACAATATACGGAACACGAATAACTTATAGAGCAGCATGCCTATGAGCCCTA
GGATTTGTA--

>G4_Macrotermes_michaelseni_4 Macrotermes michaelseni isolate TR164 cytochrome oxidase subunit I (COI) gene, partial cds; mitochondrial

----
AACAGAACTTGGACAACCAGGATCCTTAATTGGAGACGACCAAATCTACAACGTCATCGTCACAGCTCACGCTTTCGTAATGATCTTCTTCATAGTAATACCAATCATGATTGG
AGGATTCGGAAACTGACTAGTACCGCTAATATTAGGAGCACCAGACATAGCATTCCCACGAATAAACAACATAAGATTCTGATTATTACCACCATCATTAACTCTTCTTCTCACT
AGTAGAACAGTAGAAAGTGGTGCAGGAACAGGATGAACAGTATACCCACCCCTTGCAAGAGGAATTGCTCACGCCGGAGCATCAGTAGATCTAGCCATCTTCTCATTACACTT
AGCAGGAGTATCATCCATCCTAGGAGCAGTAAACTTCATTTCAACAACAATCAACATGAAACCAAAAAACATAAAACCCGAACGAATCCCATTATTCGTATGATCAGTTGCCAT
CACGGCTCTCCTACTCCTCCTATCACTACCAGTACTAGCAGGAGCAATCACAATACTATTAACTGACCGAAACCTAAACACATCCTTCTTCGATCCAGCAGGAGGTGGAGACCC
AATCCTATACCAACACTTATTCTGATTCTTCGGACACCCCGAAGTATATATTTTAATTCTACCAGGATTTGGTATAATTTCCCACATTATTTGTCACGAAAGAGGTAAAAAGGAA
GCCTTCGGAAACCTGGGAATAATTTTTGCCATATTAGCAATCGGATTACTAGGATTTGTAGTATGAGCACACCATATGTTCACAGTAGGAATAGACGTTGATACACGAGCTTAC
TTTACATCAGCAACAATAATCATTGCAGTACCTACGGGGATTAAAATCTTCAGATGACTTGCAACAATATACGGAACACGAATAACTTATAGAGCAGCATGCCTATGAGCCCTA
GGATTTGTA--

>G4_Macrotermes_michaelseni_5 Macrotermes michaelseni isolate TR166 cytochrome oxidase subunit I (COI) gene, partial cds; mitochondrial

----

AACAGAACTTGGACAACCAGGATCCTTAATTGGAGACGACCAAATCTACAACGTCATCGTCACAGCTCACGCTTTCGTAATGATCTTCTTCATAGTAATACCAATCATGATTGG

AGGATTCGGAAACTGACTAGTACCGCTAATATTAGGAGCACCAGACATAGCATTCCCACGAATAAACAACATAAGATTCTGATTATTACCACCATCATTAACTCTTCTTCTCACT

AGTAGAACAGTAGAAAGTGGTGCAGGAACAGGATGAACAGTATACCCACCCCTTGCAAGAGGAATTGCTCACGCCGGAGCATCAGTAGATCTAGCCATCTTCTCATTACACTT

AGCAGGAGTATCATCCATCCTAGGAGCAGTAAACTTCATTTCAACAACAATCAACATGAAACCAAAAAACATAAAACCCGAACGAATCCCATTATTCGTATGATCAGTTGCCAT

CACGGCTCTCCTACTCCTCCTATCACTACCAGTACTAGCAGGAGCAATCACAATACTATTAACTGACCGAAACCTAAACACATCCTTCTTCGATCCAGCAGGAGGTGGAGACCC

AATCCTATACCAACACTTATTCTGATTCTTCGGACACCCCGAAGTATATATTTTAATTCTACCAGGATTTGGTATAATTTCCCACATTATTTGTCACGAAAGAGGTAAAAAGGAA

GCCTTCGGAAACCTGGGAATAATTTTTGCCATATTAGCAATCGGATTACTAGGATTTGTAGTATGAGCACACCATATGTTCACAGTAGGAATAGACGTTGATACACGAGCTTAC

TTTACATCAGCAACAATAATCATTGCAGTACCTACGGGGATTAAAATCTTCAGATGACTTGCAACAATATACGGAACACGAATAACTTATAGAGCAGCATGCCTATGAGCCCTA

GGATTTGTA---

>G4_Macrotermes_michaelseni_6 Macrotermes michaelseni isolate TR173 cytochrome oxidase subunit I (COI) gene, partial cds; mitochondrial

----

AACAGAACTTGGACAACCAGGATCCTTAATTGGAGACGACCAAATCTACAACGTCATCGTCACAGCTCACGCTTTCGTAATGATCTTCTTCATAGTAATACCAATCATGATTGG

AGGATTCGGAAACTGACTAGTACCGCTAATATTAGGAGCACCAGACATAGCATTCCCACGAATAAACAACATAAGATTCTGATTATTACCACCATCATTAACTCTTCTTCTCACT

AGTAGAACAGTAGAAAGTGGTGCAGGAACAGGATGAACAGTATACCCACCCCTTGCAAGAGGAATTGCTCACGCCGGAGCATCAGTAGATCTAGCCATCTTCTCATTACACTT

AGCAGGAGTATCATCCATCCTAGGAGCAGTAAACTTCATTTCAACAACAATCAACATGAAACCAAAAAACATAAAACCCGAACGAATCCCATTATTCGTATGATCAGTTGCCAT

CACGGCTCTCCTACTCCTCCTATCACTACCAGTACTAGCAGGAGCAATCACAATACTATTAACTGACCGAAACCTAAACACATCCTTCTTCGATCCAGCAGGAGGTGGAGACCC

AATCCTATACCAACACTTATTCTGATTCTTCGGACACCCCGAAGTATATATTTTAATTCTACCAGGATTTGGTATAATTTCCCACATTATTTGTCACGAAAGAGGTAAAAAGGAA

GCCTTCGGAAACCTGGGAATAATTTTTGCCATATTAGCAATCGGATTACTAGGATTTGTAGTATGAGCACACCATATGTTCACAGTAGGAATAGACGTTGATACACGAGCTTAC

TTTACATCAGCAACAATAATCATTGCAGTACCTACGGGGATTAAAATCTTCAGATGACTTGCAACAATATACGGAACACGAATAACTTATAGAGCAGCATGCCTATGAGCCCTA
GGATTTGTA--

>G4_Macrotermes_michaelseni_7 Macrotermes michaelseni isolate TB08 cytochrome oxidase subunit I (COI) gene, partial cds; mitochondrial

----
AACAGAACTTGGACAACCAGGATCCTTAATTGGAGACGACCAAATCTACAACGTCATCGTCACAGCTCACGCTTTCGTAATGATCTTCTTCATAGTAATACCAATCATGATTGG
AGGATTCGGAAACTGACTAGTACCGCTAATATTAGGAGCACCAGACATAGCATTCCCACGAATAAACAACATAAGATTCTGATTATTACCACCATCATTAACTCTTCTTCTCACT
AGTAGAACAGTAGAAAGTGGTGCAGGAACAGGATGAACAGTATACCCACCCCTTGCAAGAGGAATTGCTCACGCCGGAGCATCAGTAGATCTAGCCATCTTCTCATTACACTT
AGCAGGAGTATCATCCATCCTAGGAGCAGTAAACTTCATTTCAACAACAATCAACATGAAACCAAAAAACATAAAACCCGAACGAATCCCATTATTCGTATGATCAGTTGCCAT
CACGGCTCTCCTACTCCTCCTATCACTACCAGTACTAGCAGGAGCAATCACAATACTATTAACTGACCGAAACCTAAACACATCCTTCTTCGATCCAGCAGGAGGTGGAGACCC
AATCCTATACCAACACTTATTCTGATTCTTCGGACACCCCGAAGTATATATTTTAATTCTACCAGGATTTGGTATAATTTCCCACATTATTTGTCACGAAAGAGGTAAAAAGGAA
GCCTTCGGAAACCTGGGAATAATTTTTGCCATATTAGCAATCGGATTACTAGGATTTGTAGTATGAGCACACCATATGTTCACAGTAGGAATAGACGTTGATACACGAGCTTAC
TTTACATCAGCAACAATAATCATTGCAGTACCTACGGGGATTAAAATCTTCAGATGACTTGCAACAATATACGGAACACGAATAACTTATAGAGCAGCATGCCTATGAGCCCTA
GGATTTGTA--

>G4_Macrotermes_michaelseni_8 Macrotermes michaelseni isolate TS01 cytochrome oxidase subunit I (COI) gene, partial cds; mitochondrial

----

AACAGAACTTGGACAACCAGGATCCTTAATTGGAGACGACCAAATCTACAACGTCATCGTCACAGCTCACGCTTTCGTAATGATCTTCTTCATAGTAATACCAATCATGATTGG

AGGATTCGGAAACTGACTAGTACCGCTAATATTAGGAGCACCAGACATAGCATTCCCACGAATAAACAACATAAGATTCTGATTATTACCACCATCATTAACTCTTCTTCTCACT

AGTAGAACAGTAGAAAGTGGTGCAGGAACAGGATGAACAGTATACCCACCCCTTGCAAGAGGAATTGCTCACGCCGGAGCATCAGTAGATCTAGCCATCTTCTCATTACACTT

AGCAGGAGTATCATCCATCCTAGGAGCAGTAAACTTCATTTCAACAACAATCAACATGAAACCAAAAAACATAAAACCCGAACGAATCCCATTATTCGTATGATCAGTTGCCAT

CACGGCTCTCCTACTCCTCCTATCACTACCAGTACTAGCAGGAGCAATCACAATACTATTAACTGACCGAAACCTAAACACATCCTTCTTCGATCCAGCAGGAGGTGGAGACCC

AATCCTATACCAACACTTATTCTGATTCTTCGGACACCCCGAAGTATATATTTTAATTCTACCAGGATTTGGTATAATTTCCCACATTATTTGTCACGAAAGAGGTAAAAAGGAA

GCCTTCGGAAACCTGGGAATAATTTTTGCCATATTAGCAATCGGATTACTAGGATTTGTAGTATGAGCACACCATATGTTCACAGTAGGAATAGACGTTGATACACGAGCTTAC

TTTACATCAGCAACAATAATCATTGCAGTACCTACGGGGATTAAAATCTTCAGATGACTTGCAACAATATACGGAACACGAATAACTTATAGAGCAGCATGCCTATGAGCCCTA

GGATTTGTA--

>G4_Macrotermes_michaelseni_9 Macrotermes michaelseni isolate TS03 cytochrome oxidase subunit I (COI) gene, partial cds; mitochondrial

----

AACAGAACTTGGACAACCAGGATCCTTAATTGGAGACGACCAAATCTACAACGTCATCGTCACAGCTCACGCTTTCGTAATGATCTTCTTCATAGTAATACCAATCATGATTGG

AGGATTCGGAAACTGACTAGTACCGCTAATATTAGGAGCACCAGACATAGCATTCCCACGAATAAACAACATAAGATTCTGATTATTACCACCATCATTAACTCTTCTTCTCACT

AGTAGAACAGTAGAAAGTGGTGCAGGAACAGGATGAACAGTATACCCACCCCTTGCAAGAGGAATTGCTCACGCCGGAGCATCAGTAGATCTAGCCATCTTCTCATTACACTT

AGCAGGAGTATCATCCATCCTAGGAGCAGTAAACTTCATTTCAACAACAATCAACATGAAACCAAAAAACATAAAACCCGAACGAATCCCATTATTCGTATGATCAGTTGCCAT

CACGGCTCTCCTACTCCTCCTATCACTACCAGTACTAGCAGGAGCAATCACAATACTATTAACTGACCGAAACCTAAACACATCCTTCTTCGATCCAGCAGGAGGTGGAGACCC

AATCCTATACCAACACTTATTCTGATTCTTCGGACACCCCGAAGTATATATTTTAATTCTACCAGGATTTGGTATAATTTCCCACATTATTTGTCACGAAAGAGGTAAAAAGGAA

GCCTTCGGAAACCTGGGAATAATTTTTGCCATATTAGCAATCGGATTACTAGGATTTGTAGTATGAGCACACCATATGTTCACAGTAGGAATAGACGTTGATACACGAGCTTAC

TTTACATCAGCAACAATAATCATTGCAGTACCTACGGGGATTAAAATCTTCAGATGACTTGCAACAATATACGGAACACGAATAACTTATAGAGCAGCATGCCTATGAGCCCTA
GGATTTGTA--

>G4_Macrotermes_michaelseni_10 Macrotermes michaelseni isolate TS12 cytochrome oxidase subunit I (COI) gene, partial cds; mitochondrial

----
AACAGAACTTGGACAACCAGGATCCTTAATTGGAGACGACCAAATCTACAACGTCATCGTCACAGCTCACGCTTTCGTAATGATCTTCTTCATAGTAATACCAATCATGATTGG
AGGATTCGGAAACTGACTAGTACCGCTAATATTAGGAGCACCAGACATAGCATTCCCACGAATAAACAACATAAGATTCTGATTATTACCACCATCATTAACTCTTCTTCTCACT
AGTAGAACAGTAGAAAGTGGTGCAGGAACAGGATGAACAGTATACCCACCCCTTGCAAGAGGAATTGCTCACGCCGGAGCATCAGTAGATCTAGCCATCTTCTCATTACACTT
AGCAGGAGTATCATCCATCCTAGGAGCAGTAAACTTCATTTCAACAACAATCAACATGAAACCAAAAAACATAAAACCCGAACGAATCCCATTATTCGTATGATCAGTTGCCAT
CACGGCTCTCCTACTCCTCCTATCACTACCAGTACTAGCAGGAGCAATCACAATACTATTAACTGACCGAAACCTAAACACATCCTTCTTCGATCCAGCAGGAGGTGGAGACCC
AATCCTATACCAACACTTATTCTGATTCTTCGGACACCCCGAAGTATATATTTTAATTCTACCAGGATTTGGTATAATTTCCCACATTATTTGTCACGAAAGAGGTAAAAAGGAA
GCCTTCGGAAACCTGGGAATAATTTTTGCCATATTAGCAATCGGATTACTAGGATTTGTAGTATGAGCACACCATATGTTCACAGTAGGAATAGACGTTGATACACGAGCTTAC
TTTACATCAGCAACAATAATCATTGCAGTACCTACGGGGATTAAAATCTTCAGATGACTTGCAACAATATACGGAACACGAATAACTTATAGAGCAGCATGCCTATGAGCCCTA
GGATTTGTA--

>G4_Macrotermes_michaelseni_11 Macrotermes michaelseni isolate TS16 cytochrome oxidase subunit I (COI) gene, partial cds; mitochondrial

----

AACAGAACTTGGACAACCAGGATCCTTAATTGGAGACGACCAAATCTACAACGTCATCGTCACAGCTCACGCTTTCGTAATGATCTTCTTCATAGTAATACCAATCATGATTGG

AGGATTCGGAAACTGACTAGTACCGCTAATATTAGGAGCACCAGACATAGCATTCCCACGAATAAACAACATAAGATTCTGATTATTACCACCATCATTAACTCTTCTTCTCACT

AGTAGAACAGTAGAAAGTGGTGCAGGAACAGGATGAACAGTATACCCACCCCTTGCAAGAGGAATTGCTCACGCCGGAGCATCAGTAGATCTAGCCATCTTCTCATTACACTT

AGCAGGAGTATCATCCATCCTAGGAGCAGTAAACTTCATTTCAACAACAATCAACATGAAACCAAAAAACATAAAACCCGAACGAATCCCATTATTCGTATGATCAGTTGCCAT

CACGGCTCTCCTACTCCTCCTATCACTACCAGTACTAGCAGGAGCAATCACAATACTATTAACTGACCGAAACCTAAACACATCCTTCTTCGATCCAGCAGGAGGTGGAGACCC

AATCCTATACCAACACTTATTCTGATTCTTCGGACACCCCGAAGTATATATTTTAATTCTACCAGGATTTGGTATAATTTCCCACATTATTTGTCACGAAAGAGGTAAAAAGGAA

GCCTTCGGAAACCTGGGAATAATTTTTGCCATATTAGCAATCGGATTACTAGGATTTGTAGTATGAGCACACCATATGTTCACAGTAGGAATAGACGTTGATACACGAGCTTAC

TTTACATCAGCAACAATAATCATTGCAGTACCTACGGGGATTAAAATCTTCAGATGACTTGCAACAATATACGGAACACGAATAACTTATAGAGCAGCATGCCTATGAGCCCTA

GGATTTGTA---

>G4_Macrotermes_michaelseni_12 Macrotermes michaelseni isolate TS18 cytochrome oxidase subunit I (COI) gene, partial cds; mitochondrial

----

AACAGAACTTGGACAACCAGGATCCTTAATTGGAGACGACCAAATCTACAACGTCATCGTCACAGCTCACGCTTTCGTAATGATCTTCTTCATAGTAATACCAATCATGATTGG

AGGATTCGGAAACTGACTAGTACCGCTAATATTAGGAGCACCAGACATAGCATTCCCACGAATAAACAACATAAGATTCTGATTATTACCACCATCATTAACTCTTCTTCTCACT

AGTAGAACAGTAGAAAGTGGTGCAGGAACAGGATGAACAGTATACCCACCCCTTGCAAGAGGAATTGCTCACGCCGGAGCATCAGTAGATCTAGCCATCTTCTCATTACACTT

AGCAGGAGTATCATCCATCCTAGGAGCAGTAAACTTCATTTCAACAACAATCAACATGAAACCAAAAAACATAAAACCCGAACGAATCCCATTATTCGTATGATCAGTTGCCAT

CACGGCTCTCCTACTCCTCCTATCACTACCAGTACTAGCAGGAGCAATCACAATACTATTAACTGACCGAAACCTAAACACATCCTTCTTCGATCCAGCAGGAGGTGGAGACCC

AATCCTATACCAACACTTATTCTGATTCTTCGGACACCCCGAAGTATATATTTTAATTCTACCAGGATTTGGTATAATTTCCCACATTATTTGTCACGAAAGAGGTAAAAAGGAA

GCCTTCGGAAACCTGGGAATAATTTTTGCCATATTAGCAATCGGATTACTAGGATTTGTAGTATGAGCACACCATATGTTCACAGTAGGAATAGACGTTGATACACGAGCTTAC

TTTACATCAGCAACAATAATCATTGCAGTACCTACGGGGATTAAAATCTTCAGATGACTTGCAACAATATACGGAACACGAATAACTTATAGAGCAGCATGCCTATGAGCCCTA
GGATTTGTA--

>G4_Macrotermes_michaelseni_13 Macrotermes michaelseni isolate TS22 cytochrome oxidase subunit I (COI) gene, partial cds; mitochondrial

----
AACAGAACTTGGACAACCAGGATCCTTAATTGGAGACGACCAAATCTACAACGTCATCGTCACAGCTCACGCTTTCGTAATGATCTTCTTCATAGTAATACCAATCATGATTGG
AGGATTCGGAAACTGACTAGTACCGCTAATATTAGGAGCACCAGACATAGCATTCCCACGAATAAACAACATAAGATTCTGATTATTACCACCATCATTAACTCTTCTTCTCACT
AGTAGAACAGTAGAAAGTGGTGCAGGAACAGGATGAACAGTATACCCACCCCTTGCAAGAGGAATTGCTCACGCCGGAGCATCAGTAGATCTAGCCATCTTCTCATTACACTT
AGCAGGAGTATCATCCATCCTAGGAGCAGTAAACTTCATTTCAACAACAATCAACATGAAACCAAAAAACATAAAACCCGAACGAATCCCATTATTCGTATGATCAGTTGCCAT
CACGGCTCTCCTACTCCTCCTATCACTACCAGTACTAGCAGGAGCAATCACAATACTATTAACTGACCGAAACCTAAACACATCCTTCTTCGATCCAGCAGGAGGTGGAGACCC
AATCCTATACCAACACTTATTCTGATTCTTCGGACACCCCGAAGTATATATTTTAATTCTACCAGGATTTGGTATAATTTCCCACATTATTTGTCACGAAAGAGGTAAAAAGGAA
GCCTTCGGAAACCTGGGAATAATTTTTGCCATATTAGCAATCGGATTACTAGGATTTGTAGTATGAGCACACCATATGTTCACAGTAGGAATAGACGTTGATACACGAGCTTAC
TTTACATCAGCAACAATAATCATTGCAGTACCTACGGGGATTAAAATCTTCAGATGACTTGCAACAATATACGGAACACGAATAACTTATAGAGCAGCATGCCTATGAGCCCTA
GGATTTGTA--

>G4_Macrotermes_michaelseni_14 Macrotermes michaelseni isolate TR151 cytochrome oxidase subunit I (COI) gene, partial cds; mitochondrial

----

AACAGAACTTGGACAACCAGGATCCTTAATTGGAGACGACCAAATCTACAACGTCATCGTCACAGCTCACGCTTTCGTAATGATCTTCTTCATAGTAATACCAATCATGATTGG
AGGATTCGGAAACTGACTAGTACCGCTAATATTAGGAGCACCAGACATAGCATTCCCACGAATAAACAACATAAGATTCTGATTATTACCACCATCATTAACTCTTCTTCTCACT
AGTAGAACAGTAGAAAGTGGTGCAGGAACAGGATGAACAGTATACCCACCCCTTGCAAGAGGAATTGCTCACGCCGGAGCATCAGTAGATCTAGCCATCTTCTCATTACACTT
AGCAGGAGTATCATCCATCCTAGGAGCAGTAAACTTCATTTCAACAACAATCAACATGAAACCAAAAAACATAAAACCCGAACGAATCCCATTATTCGTATGATCAGTTGCCAT
CACGGCTCTCCTACTCCTCCTATCACTACCAGTACTAGCAGGAGCAATCACAATACTATTAACTGACCGAAACCTAAACACATCCTTCTTCGATCCAGCAGGAGGTGGAGACCC
AATCCTATACCAACACTTATTCTGATTCTTCGGACACCCCGAAGTATATATTTTAATTCTACCAGGATTTGGTATAATTTCCCACATTATTTGTCACGAAAGAGGTAAAAAGGAA
GCCTTCGGAAACCTGGGAATAATTTTTGCCATATTAGCAATCGGATTACTAGGATTTGTAGTATGAGCACACCATATGTTCACAGTAGGAATAGACGTTGATACACGAGCTTAC
TTTACATCAGCAACAATAATCATTGCAGTACCTACGGGAATTAAAATCTTCAGATGACTTGCAACAATATACGGAACACGAATAACTTATAGAGCAGCATGCCTATGAGCCCTA
GGATTTGTA---

>G4_Macrotermes_michaelseni_15 Macrotermes michaelseni isolate TS02 cytochrome oxidase subunit I (COI) gene, partial cds; mitochondrial

----

AACAGAACTTGGACAACCAGGATCCTTAATTGGAGACGACCAAATCTACAACGTCATCGTCACAGCTCACGCTTTCGTAATGATCTTCTTCATAGTAATACCAATCATGATTGG
AGGATTCGGAAACTGACTAGTACCGCTAATATTAGGAGCACCAGACATAGCATTCCCACGAATAAACAACATAAGATTCTGATTATTACCACCATCATTAACTCTTCTTCTCACT
AGTAGAACAGTAGAAAGTGGTGCAGGAACAGGATGAACAGTATACCCACCCCTTGCAAGAGGAATTGCTCACGCCGGAGCATCAGTAGATCTAGCCATCTTCTCATTACACTT
AGCAGGAGTATCATCCATCCTAGGAGCAGTAAACTTCATTTCAACAACAATCAACATGAAACCAAAAAACATAAAACCCGAACGAATCCCATTATTCGTATGATCAGTTGCCAT
CACGGCTCTCCTACTCCTCCTATCACTACCAGTACTAGCAGGAGCAATCACAATACTATTAACTGACCGAAACCTAAACACATCCTTCTTCGATCCAGCAGGAGGTGGAGACCC
AATCCTATACCAACACTTATTCTGATTCTTCGGACACCCCGAAGTATATATTTTAATTCTACCAGGATTTGGTATAATTTCCCACATTATTTGTCACGAAAGAGGTAAAAAGGAA
GCCTTCGGAAACCTGGGAATAATTTTTGCCATATTAGCAATCGGATTACTAGGATTTGTAGTATGAGCACACCATATGTTCACAGTAGGAATAGACGTTGATACACGAGCTTAC

TTTACATCAGCAACAATAATCATTGCAGTACCTACGGGAATTAAAATCTTCAGATGACTTGCAACAATATACGGAACACGAATAACTTATAGAGCAGCATGCCTATGAGCCCTA
GGATTTGTA--

>G4_Macrotermes_michaelseni_16 Macrotermes michaelseni isolate TS04 cytochrome oxidase subunit I (COI) gene, partial cds; mitochondrial

----
AACAGAACTTGGACAACCAGGATCCTTAATTGGAGACGACCAAATCTACAACGTCATCGTCACAGCTCACGCTTTCGTAATGATCTTCTTCATAGTAATACCAATCATGATTGG
AGGATTCGGAAACTGACTAGTACCGCTAATATTAGGAGCACCAGACATAGCATTCCCACGAATAAACAACATAAGATTCTGATTATTACCACCATCATTAACTCTTCTTCTCACT
AGTAGAACAGTAGAAAGTGGTGCAGGAACAGGATGAACAGTATACCCACCCCTTGCAAGAGGAATTGCTCACGCCGGAGCATCAGTAGATCTAGCCATCTTCTCATTACACTT
AGCAGGAGTATCATCCATCCTAGGAGCAGTAAACTTCATTTCAACAACAATCAACATGAAACCAAAAAACATAAAACCCGAACGAATCCCATTATTCGTATGATCAGTTGCCAT
CACGGCTCTCCTACTCCTCCTATCACTACCAGTACTAGCAGGAGCAATCACAATACTATTAACTGACCGAAACCTAAACACATCCTTCTTCGATCCAGCAGGAGGTGGAGACCC
AATCCTATACCAACACTTATTCTGATTCTTCGGACACCCCGAAGTATATATTTTAATTCTACCAGGATTTGGTATAATTTCCCACATTATTTGTCACGAAAGAGGTAAAAAGGAA
GCCTTCGGAAACCTGGGAATAATTTTTGCCATATTAGCAATCGGATTACTAGGATTTGTAGTATGAGCACACCATATGTTCACAGTAGGAATAGACGTTGATACACGAGCTTAC
TTTACATCAGCAACAATAATCATTGCAGTACCTACGGGAATTAAAATCTTCAGATGACTTGCAACAATATACGGAACACGAATAACTTATAGAGCAGCATGCCTATGAGCCCTA
GGATTTGTA--

>G4_Macrotermes_michaelseni_17 Macrotermes michaelseni isolate TS15 cytochrome oxidase subunit I (COI) gene, partial cds; mitochondrial

----

AACAGAACTTGGACAACCAGGATCCTTAATTGGAGACGACCAAATCTACAACGTCATCGTCACAGCTCACGCTTTCGTAATGATCTTCTTCATAGTAATACCAATCATGATTGG

AGGATTCGGAAACTGACTAGTACCGCTAATATTAGGAGCACCAGACATAGCATTCCCACGAATAAACAACATAAGATTCTGATTATTACCACCATCATTAACTCTTCTTCTCACT

AGTAGAACAGTAGAAAGTGGTGCAGGAACAGGATGAACAGTATACCCACCCCTTGCAAGAGGAATTGCTCACGCCGGAGCATCAGTAGATCTAGCCATCTTCTCATTACACTT

AGCAGGAGTATCATCCATCCTAGGAGCAGTAAACTTCATTTCAACAACAATCAACATGAAACCAAAAAACATAAAACCCGAACGAATCCCATTATTCGTATGATCAGTTGCCAT

CACGGCTCTCCTACTCCTCCTATCACTACCAGTACTAGCAGGAGCAATCACAATACTATTAACTGACCGAAACCTAAACACATCCTTCTTCGATCCAGCAGGAGGTGGAGACCC

AATCCTATACCAACACTTATTCTGATTCTTCGGACACCCCGAAGTATATATTTTAATTCTACCAGGATTTGGTATAATTTCCCACATTATTTGTCACGAAAGAGGTAAAAAGGAA

GCCTTCGGAAACCTGGGAATAATTTTTGCCATATTAGCAATCGGATTACTAGGATTTGTAGTATGAGCACACCATATGTTCACAGTAGGAATAGACGTTGATACACGAGCTTAC

TTTACATCAGCAACAATAATCATTGCAGTACCTACGGGAATTAAAATCTTCAGATGACTTGCAACAATATACGGAACACGAATAACTTATAGAGCAGCATGCCTATGAGCCCTA

GGATTTGTA---

>G4_Macrotermes_michaelseni_18 Macrotermes michaelseni isolate TS19 cytochrome oxidase subunit I (COI) gene, partial cds; mitochondrial

----

AACAGAACTTGGACAACCAGGATCCTTAATTGGAGACGACCAAATCTACAACGTCATCGTCACAGCTCACGCTTTCGTAATGATCTTCTTCATAGTAATACCAATCATGATTGG

AGGATTCGGAAACTGACTAGTACCGCTAATATTAGGAGCACCAGACATAGCATTCCCACGAATAAACAACATAAGATTCTGATTATTACCACCATCATTAACTCTTCTTCTCACT

AGTAGAACAGTAGAAAGTGGTGCAGGAACAGGATGAACAGTATACCCACCCCTTGCAAGAGGAATTGCTCACGCCGGAGCATCAGTAGATCTAGCCATCTTCTCATTACACTT

AGCAGGAGTATCATCCATCCTAGGAGCAGTAAACTTCATTTCAACAACAATCAACATGAAACCAAAAAACATAAAACCCGAACGAATCCCATTATTCGTATGATCAGTTGCCAT

CACGGCTCTCCTACTCCTCCTATCACTACCAGTACTAGCAGGAGCAATCACAATACTATTAACTGACCGAAACCTAAACACATCCTTCTTCGATCCAGCAGGAGGTGGAGACCC

AATCCTATACCAACACTTATTCTGATTCTTCGGACACCCCGAAGTATATATTTTAATTCTACCAGGATTTGGTATAATTTCCCACATTATTTGTCACGAAAGAGGTAAAAAGGAA

GCCTTCGGAAACCTGGGAATAATTTTTGCCATATTAGCAATCGGATTACTAGGATTTGTAGTATGAGCACACCATATGTTCACAGTAGGAATAGACGTTGATACACGAGCTTAC

TTTACATCAGCAACAATAATCATTGCAGTACCTACGGGAATTAAAATCTTCAGATGACTTGCAACAATATACGGAACACGAATAACTTATAGAGCAGCATGCCTATGAGCCCTA

GGATTTGTA---

>G4_Macrotermes_michaelseni_19 Macrotermes michaelseni isolate TR175 cytochrome oxidase subunit I (COI) gene, partial cds; mitochondrial

----

AACAGAACTTGGACAACCAGGATCCTTAATTGGAGACGACCAAATCTACAACGTCATCGTCACAGCTCACGCTTTCGTAATGATCTTCTTCATAGTAATACCAATCATGATTGG

AGGATTCGGAAACTGACTAGTACCGCTAATATTAGGAGCACCAGACATAGCATTCCCACGAATAAACAACATAAGATTCTGATTATTACCACCATCATTAACTCTTCTTCTCACT

AGTAGAACAGTAGAAAGTGGTGCAGGAACAGGATGAACAGTATACCCACCCCTTGCAAGAGGAATTGCTCACGCCGGAGCATCAGTAGATCTAGCCATCTTCTCATTACACTT

AGCAGGAGTATCATCCATCCTAGGAGCAGTAAACTTCATTTCAACAACAATCAACATGAAACCAAAAAACATAAAACCCGAACGAATCCCATTATTCGTATGATCAGTTGCCAT

CACGGCTCTCCTACTCCTCCTATCACTACCAGTACTAGCAGGAGCAATCACAATACTATTAACTGACCGAAACCTAAACACATCCTTCTTCGATCCAGCAGGAGGTGGAGACCC

AATCCTATACCAACACTTATTCTGATTCTTCGGACACCCCGAAGTATATATTTTAATTCTACCAGGATTTGGTATAATTTCCCACATTATTTGTCACGAAAGAGGTAAAAAGGAA

GCCTTCGGAAACCTGGGGATAATTTTTGCCATATTAGCAATCGGATTACTAGGATTTGTAGTATGAGCACACCATATGTTCACAGTAGGAATAGACGTTGATACACGAGCTTAC

TTTACATCAGCAACAATAATCATTGCAGTACCTACGGGAATTAAAATCTTCAGATGACTTGCAACAATATACGGAACACGAATAACTTATAGAGCAGCATGCCTATGAGCCCTA

GGATTTGTA---

>G4_Macrotermes_jeanneli_3 Macrotermes jeanneli isolate MJ2625 cytocrome oxidase subunit I (COI) gene, partial cds; mitochondrial

----

CTAATCCGAACAGAACTTGGACAACCAGGATCCTTAATCGGAGACGACCAAATCTACAACGTCATCGTTACAGCTCACGCTTTCGTAATGATCTTCTTCATAGTAATACCAATCA

TGATTGGAGGATTCGGAAACTGACTAGTACCGCTAATATTAGGAGCACCAGACATAGCATTCCCACGAATAAACAACATAAGATTCTGATTATTACCACCATCATTAACTCTTC

TTCTCACTAGTAGAACAGTAGAAAGTGGTGCAGGAACAGGATGAACAGTATACCCACCCCTTGCAAGAGGAATTGCTCACGCCGGAGCATCAGTAGATCTAGCAATCTTCTCA

TTACACTTAGCAGGAGTATCATCCATCCTAGGAGCAGTAAACTTTATTTCAACAACAATCAACATGAAACCAAAAAACATAAAACCCGAACGAATCCCCTTATTCGTATGATCA

GTTGCCATCACGGCTCTCCTACTCCTCCTATCACTACCAGTACTAGCAGGAGCAATCACAATACTATTAACTGACCGAAACCTAAACACATCCTTCTTTGATCCAGCAGGAGGTG

GAGACCCAATCCTATACCAACACTTATTTTGATTCTTCGGACACCCTGAAGTATATATCTTAATCCTACCAGGATTCGGTATAATTTCCCACATTATTTGTCACGAAAGAGGTAAA

AAGGAAGCCTTCGGAAACCTGGGAATAATTTTCGCCATATTAGCAATCGGATTACTAGGATTTGTAGTATGAGCACACCATATGTTCACAGTAGGAATAGACGTTGATACACG

AGCCTACTTTACATCAGCAACAATAATCATTGCAGTACCTACGGGGATTAAAATCTTCAGATGACTTGCAACAATATACGGAACCCGAATAACTTATAGAGCAGCATGCCTATG

AGCCCTAGGATTTGTATTCCT--

>G2_Macrotermes_falciger_1 Macrotermes falciger isolate Mf01 cytochrome oxidase subunit I (COI) gene, partial cds; mitochondrial

----

AACACTATACTTNGTATTNGGAGCCTGATCAGGAATGGTTGGAACATCCCTAAGAATACTAATCCGAACAGAACTTGGACAACCAGGATCCTTAATTGGGGACGATCAAATCT

ACAACGTCATCGTCACAGCTCATGCTTTCGTAATAATCTTCTTCATAGTAATACCAATCATGATTNGAGGGTTCGGAAACTGACTAGTACCGCTAATATTAGGAGCACCAGACA

TAGCATTCCCACGAATAAACAACATAAGATTCTGATTACTACCACAATCACTAACTCTTCTTCTCACTAGTAGAACAGTAGAAAGTGGTGCAGGAACAGGATGAACAGTATACC

CTCCCCTTGCAAGAGGAATTGCCCACGCCGGAGCATCGGTAGATCTAGCCATCTTCTCACTACACTTAGCAGGAGTATCATCAATCCTAGGAGCAGTAAATTTCATCTCAACAA

CAATCAACATGAAACCAAAAAACATAAAGCCCGAACGAATTCCCCTATTCNTATGATCAGTTGCCATCACGGCCCTCCTATTCCTCCTATCACTACCAGTACTAGCAGGAGCAAT

CACAATACTATTAACCGACCGAAACCTAAACACATCCTTCTTCGATCCAGCAGGAGGTGGAGACCCAATCCTATATCAACACTTATTT--

----

>G2_Macrotermes_falciger_2 Macrotermes falciger isolate Mf04 cytochrome oxidase subunit I (COI) gene, partial cds; mitochondrial

----

AACACTATACTTCGTATTCGGAGCCTGATCAGGAATGGTTGGAACATCCCTAAGAATACTAATCCGAACAGAACTTGGACAACCAGGATCCTTAATTGGGGACGATCAAATCT
ACAACGTCATCGTCACAGCTCATGCTTTCGTAATAATCTTCTTCATAGTAATACCAATCATGATTNGAGGGTTCGGAAACTGACTAGTACCGCTAATATTAGGAGCACCAGACA
TAGCATTCCCACGAATAAACAACATAAGATTCTGATTACTACCACAATCACTAACTCTTCTTCTCACTAGTAGAACAGTAGAAAGTGGTGCAGGAACAGGATGAACAGTATACC
CTCCCCTTGCAAGAGGAATTGCCCACGCCGGAGCATCGGTAGATCTAGCCATCTTCTCACTACACTTAGCAGGAGTATCATCAATCCTAGGAGCAGTAAATTTCATCTCAACAA
CAATCAACATGAAACCAAAAAACATAAAGCCCGAACGAATTCCCCTATTCATATGATCAGTTGCCATCACGGCCCTCCTATTCCTCCTATCACTACCAGTACTAGCAGGAGCAAT
CACAATACTATTAACCGACCGAAACCTAAACACATCCTTCTTCGATCCAGCAGGAGGTGGAGACCCAATCCTATATCAACACTTATTT--
----

>G2_Macrotermes_falciger_3 Macrotermes falciger isolate Mf02 cytochrome oxidase subunit I (COI) gene, partial cds; mitochondrial

----

AACNCTATACTTCGTATTCGGAGCCTGATCAGGAATGGTTGGAACATCCCTAAGAATACTAATCCGAACAGAACTTGGACAACCAGGATCCTTAATTGGGGACGATCAAATCT
ACAACGTCATCGTCACAGCTCATGCTTTCGTAATAATCTTCTTCATAGTAATACCAATCATGATTNGAGGGTTCGGAAACTGACTAGTACCGCTAATATTAGGAGCACCAGACA

TAGCATTCCCACGAATAAACAACATAAGATTCTGATTACTACCACAATCACTAACTCTTCTTCTCACTAGTAGAACAGTAGAAAGTGGTGCAGGAACAGGATGAACAGTATACC

CTCCCCTTGCAAGAGGAATTGCCCACGCCGGAGCATCGGTAGATCTAGCCATCTTCTCACTACACTTAGCAGGAGTATCATCAATCCTAGGAGCAGTAAATTTCATCTCAACAA

CAATCAACATGAAACCAAAAAACATAAAGCCCGAACGAATTCCCCTATTCATATGATCAGTTGCCATCACGGCCCTCCTATTCCTCCTATCACTACCAGTACTAGCAGGAGCAAT

CACAATACTATTAACCGACCGAAACCTAAACACATCCTTCTTCGATCCAGCAGGAGGTGGAGACCCAATCCTATATCAACACTTATTT--

----

>G2_Macrotermes_falciger_4 Macrotermes falciger isolate Mf03 cytochrome oxidase subunit I (COI) gene, partial cds; mitochondrial

----

AACNCTATACTTCGTATTNGGAGCNTGATCAGGAATGGTTGGAACATCCCTAAGAATANTAATCCGAACAGAACTTGGACAACCAGGATCNTTAATTGGGGACGATCAAATC

TACAACGTCATCGTCACAGCTCATGCTTTCGTAATAATCTTCTTCATAGTAATACCAATCATGATTGGAGGNTTCGGAAACTGACTAGTACCGCTAATATTAGGAGCACCAGAC

ATAGCATTCCCACGAATAAACAACATAAGATTCTGATTACTACCACNATCACTAACTCTTCTTCTCACTAGTAGAACAGTAGAAAGTGGTGCAGGAACAGGATGAACAGTATAC

CCTCCCCTTGCAAGAGGAATTGCCCACGCCGGAGCATCNGTAGATCTAGCCATCTTCTCACTACACTTAGCAGGAGTATCATCAATCCTAGGAGCAGTAAATTTCATCTCAACA

ACAATCAACATGAAACCAAAAAACATAAAGCCCGAACGAATTCCCCTATTCNTATGATCAGTTGCCATCACNGCCCTCCTANTCCTCCTATCACTACCAGTACTAGCAGGAGCA

ATCACAATANTATTAACCGACCGAAACCTAAACACATCCTTCTTCGATCCAGCAGGAGGTGGAGACCCAATCCTATANCAACACNTATTT---

----

>G2_Macrotermes_falciger_5 Macrotermes falciger isolate Mf05 cytochrome oxidase subunit I (COI) gene, partial cds; mitochondrial

----

AACACTATANTTCGTNTTTGGAGCNTGATCAGGAATGGTTGGAACATCCCTAAGAATACTAATCCGAACAGAACTTGGACAACCAGGATCCTTAATTGGGGACGANCAAATCT

ACAACGTCATCGTCACAGCTCATGCTTTCGTAATAATCTTCTTCATAGTAATACCAATCATGATTGGAGGGTTCGGAAACTGACTAGTACCGCTAATATTAGGAGCACCAGACA

TAGCATTCCCACGAATAAACAACATAAGATTCTGATTACTACCACNATCACTAACTCTTCTTCTCACTAGTAGAACAGTAGAAAGTGGTGCAGGAACAGGATGAACAGTATACC

CTCCCCTTGCAAGAGGAATTGCCCACGCCGGAGCATCNGTAGATCTAGCCATCTTCTCACTACACTTAGCAGGAGTATCATCAATCCTAGGAGCAGTAAATTTCATCTCAACAA

CAATCAACATGAAACCAAAAAACATAAAGCCCGAACGAATTCCCCTATTCNTATGATCAGTTGCCATCACNGCCCTCCTANTCCTCCTATCACTACCAGTACTAGCAGGAGCAA

TCACAATANTATTAACCGACCGAAACCTAAACACATCCTTCTTCGATCCAGCAGGAGGTGGAGACCCAATCCTATATCAACACTTATTT--

----

>G2_Macrotermes_subhyalinus_1 Macrotermes subhyalinus isolate Ms14 cytochrome oxidase subunit I (COI) gene, partial cds; mitochondrial

----

AACACTATACTTCGTATTNGGAGCCTGATCAGGAATGGTTGGAACATCCCTAAGAATACTAATCCGAACAGAACTTGGACAACCAGGATCCTTAATTGGGGACGATCAAATCT

ACAACGTCATCGTCACAGCTCATGCTTTCGTAATGATCTTCTTCATAGTAATACCAATCATGATTGGAGGGTTCGGAAACTGACTAGTACCGCTAATATTAGGAGCACCAGACA

TAGCATTCCCACGAATAAACAACATAAGATTCTGATTACTACCACNATCACTAACTCTTCTTCTCACTAGTAGAACAGTAGAAAGTGGTGCAGGAACAGGATGAACAGTATACC

CTCCCCTTGCAAGAGGAATTGCCCACGCTGGAGCATCGGTAGATCTAGCCATCTTCTCACTACACTTAGCAGGAGTATCATCAATCCTAGGAGCAGTAAATTTCATCTCAACAA

CAATCAACATGAAACCAAAAAACATAAAGCCCGAACGAATTCCCCTATTCATATGATCAGTTGCCATCACGGCCCTCCTACTCCTCCTATCACTACCAGTACTAGCAGGAGCAAT

CACAATACTATTAACCGACCGAAACCTAAACACATCCTTCTTNGATCCAGCAGGAGGTGGAGACCCAATCCTATATCAACACTTATT---

----

>G2_Macrotermes_subhyalinus_2 Macrotermes subhyalinus isolate Ms11 cytochrome oxidase subunit I (COI) gene, partial cds; mitochondrial

----

AACACTATACTTCGTATTTGGAGCCTGATCAGGAATGGTTGGAACATCCCTAAGAATACTAATCCGAACAGAACTTGGACAACCAGGATCCTTAATCGGGGACGATCAAATCT

ACAACGTCATCGTCACAGCTCANGCTTTCGTAATGATCTTCTTCATAGTAATACCAATCATGATTGGAGGATTCGGAAACTGACTAGTACCGCTAATATTAGGAGCACCAGACA

TAGCATTCCCACGAATAAACAACATAAGATTCTGATTANTACCACCATCANTAACTCTTCTTCTCACTAGTAGAACAGTAGAAAGTGGTGCAGGAACAGGATGAACAGTATACC

CTCCCCTTGCAAGAGGAATTGCCCACGCNGGAGCATCGGTAGATCTAGCCATCTTCTCACTACACTTAGCAGGAGTATCATCCATCCTAGGAGCAGTAAATTTCATCTCAACAA

CAATCAACATGAAACCAAAAAACATAAANCCCGAACGAATTCCCCTATTCGTATGATCAGTTGCCATCACGGCNCTCCTACTCCTCCTATCACTACCAGTACTAGCAGGAGCAA

TCACAATACTATTAACCGACCGAAACCTAAACACATCCTTCTTTGATCCAGCAGGAGGTGGAGACCCAATCCTATATCAACACTTATTC---

----

>G2_Macrotermes_subhyalinus_3 Macrotermes subhyalinus isolate Ms13 cytochrome oxidase subunit I (COI) gene, partial cds; mitochondrial

----

AACACTATACTTCGTATTTGGAGCCTGATCAGGAATGGTTGGAACATCCCTAAGAATACTAATCCGAACAGAACTTGGACAACCAGGATCCTTAATNGGGGACGATCAAATCT

ACAACGTCATCGTCACAGCTCANGCTTTCGTAATGATCTTCTTCATAGTAATACCAATNATGATTGGAGGGTTCGGAAACTGACTAGTACCGCTAATATTAGGAGCACCAGACA

TAGCATTCCCACGAATAAACAACATAAGATTCTGATTATTACCACCATCATTAACTCTTCTTCTCACTAGTAGAACAGTAGAAAGTGGTGCAGGAACAGGATGAACAGTATACC

CTCCCCTTGCAAGAGGAATTGCCCACGCNGGAGCATCGGTAGATCTAGCCATCTTCTCANTACACTTAGCAGGAGTATCATCCATCCTAGGAGCAGTAAATTTCATCTCAACAA

CAATCAACATGAAACCAAAAAACATAAAGCCCGAACGAATTCCCNTATTCGTATGATCAGTTGCCATCACGGCNCTCCTACTCCTCCTATCACTACCAGTACTAGCAGGAGCAA

TCACAATACTATTAACCGACCGAAACCTAAACACATCCTTCTTTGATCCAGCAGGAGGTGGAGACCCAATCCTATANCAACACTTATTC---

----

>G2_Macrotermes_subhyalinus_4 Macrotermes subhyalinus isolate Ms15 cytochrome oxidase subunit I (COI) gene, partial cds; mitochondrial

----

AACACTATACTTCGTATTTGGAGCCTGATCAGGAATGGTTGGAACATCCCTAAGAATACTAATCCGAACAGAACTTGGACAACCAGGATCCTTAATNGGGGACGATCAAATCT

ACAACGTCATCGTCACAGCTCANGCTTTCGTAATGATCTTCTTCATAGTAATACCAATCATGATTGGAGGNTTCGGAAACTGACTAGTACCGCTAATATTAGGAGCACCAGACA

TAGCATTCCCACGAATAAACAACATAAGATTCTGATTATTACCACCATCATTAACTCTTCTTCTCACTAGTAGAACAGTAGAAAGTGGTGCAGGAACAGGATGAACAGTATACC

CTCCCCTTGCAAGAGGAATTGCCCACGCNGGAGCATCNGTAGATCTAGCCATCTTCTCANTACACTTAGCAGGAGTATCATCNATCCTAGGAGCAGTAAATTTCATCTCAACAA

CAATCAACATGAAACCAAAAAACATAAAACCCGAACGAATTCCCNTATTCATATGATCAGTTGCCATCACGGCNCTCCTACTCCTCCTATCACTACCAGTACTAGCAGGAGCAA

TCACAATACTATTAACCGACCGAAACCTAAACACATCCTTCTTNGATCCAGCAGGAGGTGGAGACCCAATCCTATANCAACACTTATTC---

----

>G2_Macrotermes_subhyalinus_5 Macrotermes subhyalinus isolate Ms12 cytochrome oxidase subunit I (COI) gene, partial cds; mitochondrial

----

AACACTATACTTNGTATTTGGAGCATGATCAGGAATGGTTGGAACATCCCTAAGAATACTAATCCGAACAGAACTTGGACAACCAGGATCCTTAATCGGGGACGATCAAATCT

ACAACGTCATCGTCACAGCTCATGCTTTCGTAATGATCTTCTTCATAGTAATACCAATCATGATTGGAGGATTCGGAAACTGACTAGTACCGCTAATATTAGGAGCACCAGACA

TAGCATTCCCACGAATAAACAACATAAGATTCTGATTACTACCACCATCANTAACTCTTCTTCTCACTAGTAGAACAGTAGAAAGTGGTGCAGGAACAGGATGAACAGTATACC

CTCCCCTTGCAAGAGGAATTGCCCACGCNGGAGCATCGGTAGATCTAGCCATCTTCTCACTACACTTAGCAGGAGTATCATCAATCCTAGGAGCAGTAAATTTCATCTCAACAA

CAATCAACATGAAACCAAAAAACATAAAACCCGAACGAATTCCCCTATTCGTATGATCAGTTGCCATCACGGCCCTCCTACTCCTCCTATCACTACCAGTACTAGCAGGAGCAAT

CACAATACTATTAACCGACCGAAACCTAAACACATCCTTCTTTGATCCAGCAGGAGGTGGAGACCCAATCCTATANCAACACTTATTC---

----

>G2_Macrotermes_natalensis_1 Macrotermes natalensis isolate NDT27 cytochrome oxidase subunit 1 (COI) gene, partial cds; mitochondrial

----

GGAGCCTGATCAGGAATGGTTGGAACATCCCTAAGAATACTAATCCGAACAGAACTTGGACAACCAGGATCCTTAATTGGGGACGATCAAATCTACAACGTCATCATCACAGC

TCATGCTTTCGTAATAATCTTCTTCATAGTAATACCAATCATGATTGGAGGGTTCGGAAACTGACTAGTACCGCTAATATTAGGAGCACCAGACATAGCATTCCCACGAATAAA

CAACATAAGATTCTGATTACTACCACCATCACTAACTCTTCTTCTCACTAGTAGAACAGTAGAAAGTGGTGCAGGAACAGGATGAACAGTATACCCTCCCCTTGCAAGAAGAAT

TGCCCACGCCGGAGCATCGGTAGATCTAGCCATCTTCTCACTACACTTAGCAGGAGTATCATCAATCCTAGGAGCAGTAAATTTCATCTCAACAACAATCAACATGAAACCAAA

AAACATAAAGCCCGAACGAATTCCCCTATTCGTATGATCAGTTGCCATCACGGCCCTCCTACTCCTCCTATCACTACCAGTACTAGCAGGAGCAATCACAATACTATTAACCGAC

CGAAACCTAAACACATCCTTCTTCGATCCAGCAGGAGGTGGAGACCCA---

>G4_Macrotermes_jeanneli_1 Macrotermes jeanneli isolate Mj01 cytochrome oxidase subunit I (COI) gene, partial cds; mitochondrial

----

AACACTATACTTCGTATTTGGAGCCTGATCAGGAATGGTTGGAACATCCCTAAGAATACTAATCCGAACAGAACTTGGACAACCAGGATCCTTAATCGGAGACGACCAAATCT
ACAACGTCATCGTTACAGCTCACGCTTTCGTAATGATCTTCTTCATAGTAATACCAATCATGATTGGAGGATTCGGAAACTGACTAGTACCGCTAATATTAGGAGCACCAGACA
TAGCATTCCCACGAATAAACAACATAAGATTCTGATTATTACCACCATCATTAACTCTTCTTCTCACTAGTAGAACAGTAGAAAGTGGTGCAGGAACAGGATGAACAGTATACC
CACCCCTTGCAAGAGGAATTGCTCACGCCGGAGCATCAGTAGATCTAGCAATCTTCTCATTACACTTAGCAGGAGTATCATCCATCCTAGGAGCAGTAAACTTTATTTCAACAA
CAATCAACATGAAACCAAAAAACATAAAACCCGAACGAATCCCCTTATTCGTATGATCAGTTGCCATCACGGCTCTCCTACTCCTCCTATCACTACCAGTACTAGCAGGAGCAAT
CACAATACTATTAACTGACCGAAACCTAAACACATCCTTCTTTGATCCAGCAGGAGGTGGAGACCCAATCCTATACCAACACTTATTT---
----

>G4_Macrotermes_jeanneli_2 Macrotermes jeanneli isolate Mj03 cytochrome oxidase subunit I (COI) gene, partial cds; mitochondrial

----

AACACTATACTTCGTATTTGGAGCCTGATCAGGAATGGTTGGAACATCCCTAAGAATACTAATCCGAACAGAACTTGGACAACCAGGATCCTTAATCGGAGACGACCAAATCT

ACAACGTCATCGTTACAGCTCACGCTTTCGTAATGATCTTCTTCATAGTAATACCAATCATGATTGGAGGATTCGGAAACTGACTAGTACCGCTAATATTAGGAGCACCAGACA
TAGCATTCCCACGAATAAACAACATAAGATTCTGATTATTACCACCATCATTAACTCTTCTTCTCACTAGTAGAACAGTAGAAAGTGGTGCAGGAACAGGATGAACAGTATACC
CACCCCTTGCAAGAGGAATTGCTCACGCCGGAGCATCAGTAGATCTAGCAATCTTCTCATTACACTTAGCAGGAGTATCATCCATCCTAGGAGCAGTAAACTTTATTTCAACAA
CAATCAACATGAAACCAAAAAACATAAAACCCGAACGAATCCCCTTATTCGTATGATCAGTTGCCATCACGGCTCTCCTACTCCTCCTATCACTACCAGTACTAGCAGGAGCAAT
CACAATACTATTAACTGACCGAAACCTAAACACATCCTTCTTTGATCCAGCAGGAGGTGGAGACCCAATCCTATACCAACACTTATTT--
----

>G8_Macrotermes_herus_4 Macrotermes herus isolate Mh04 cytochrome oxidase subunit I (COI) gene, partial cds; mitochondrial

----
AACACTATACTTCGTATTCGGAGCCTGATCAGGAATGGTTGGAACATCCCTAAGAATATTAATCCGAACAGAACTTGGACAACCAGGATCCCTAATTGGAGATGATCAAATCT
ACAACGTCATCGTCACAGCCCATGCTTTCGTAATAATCTTCTTCATAGTAATACCAATCATGATTGGAGGATTCGGAAACTGACTAGTACCACTAATATTAGGAGCACCAGACA
TAGCATTCCCACGAATAAACAACATAAGATTTTGATTATTACCACCATCACTAACTCTTCTTCTTACTAGTAGAACAGTAGAAAGTGGTGCAGGAACAGGATGAACAGTGTACC
CTCCCCTTGCAAGAGGAATTGCTCACGCCGGAGCATCAGTAGACCTAGCCATCTTCTCACTACACTTAGCAGGAGTATCATCAATCCTAGGAGCAGTAAACTTCATCTCAACAA
CAATCAACATGAAACCAAAAAACATAAAACCCGAACGAATCCCCCTATTCGTATGATCAGTTGCCATCACGGCCCTCCTGCTCCTCCTATCACTACCAGTACTAGCAGGAGCAA
TCACAATGCTACTAACCGACCGAAACCTAAACACATCCTTCTTCGACCCAGCAGGAGGAGGAGACCCAATTCTATATCAACACTTATTT---
----

>G8_Macrotermes_herus_5 Macrotermes herus isolate Mh05 cytochrome oxidase subunit I (COI) gene, partial cds; mitochondrial

----

AACACTATACTTCGTATTCGGAGCCTGATCAGGAATGGTTGGAACATCCCTAAGAATATTAATCCGAACAGAACTTGGACAACCAGGATCCCTAATTGGAGATGATCAAATCT

ACAACGTCATCGTCACAGCCCACGCTTTCGTAATAATCTTCTTCATAGTAATACCAATCATGATTGGAGGATTCGGAAACTGACTAGTACCACTAATATTAGGAGCACCAGACA

TAGCATTCCCACGAATAAACAACATAAGATTTTGATTATTACCACCATCACTAACTCTTCTTCTTACTAGTAGAACAGTAGAAAGTGGTGCAGGAACAGGATGAACAGTGTACC

CTCCCCTTGCAAGAGGAATTGCTCACGCCGGAGCATCAGTAGACCTAGCCATCTTCTCACTACACTTAGCAGGAGTATCATCAATCCTAGGAGCAGTAAACTTCATCTCAACAA

CAATCAACATGAAACCAAAAAACATAAAACCCGAACGAATCCCCCTATTCGTATGATCAGTTGCCATCACGGCCCTCCTGCTCCTCCTATCACTACCAGTACTAGCAGGAGCAA

TCACAATGCTACTAACCGACCGAAACCTAAACACATCCTTCTTCGACCCAGCAGGAGGAGGAGACCCAATTCTATATCAACACTTATTT---

----

>G10_Macrotermes_herus_1 Macrotermes herus isolate Mh01 cytochrome oxidase subunit I (COI) gene, partial cds; mitochondrial

----

AACACTATACTTCGTATTCGGAGCCTGATCAGGAATGGTTGGAACATCCCTAAGAATACTAATCCGAACAGAACTTGGACAACCAGGATCCCTAATTGGAGACGATCAAATCT

ACAACGTCATCGTCACAGCCCATGCTTTCGTAATGATCTTCTTCATAGTAATACCAATCATGATTGGAGGATTCGGAAACTGATTAGTACCGCTAATATTAGGAGCACCAGACA

TAGCATTCCCACGAATAAACAACATAAGATTTTGATTATTACCACCATCACTAACTCTTCTTCTTACTAGTAGAACAGTAGAAAGTGGTGCAGGAACAGGATGAACAGTGTACC

CTCCCCTTGCAAGAGGAATTGCCCACGCCGGAGCATCAGTAGACCTAGCCATCTTCTCACTACACTTAGCAGGAGTATCATCCATCTTAGGAGCAGTAAATTTCATTTCAACAA

CAATCAACATGAAACCAAAAAGCATAAAGCCTGAACGAATCCCCCTATTCGTATGATCAGTTGCCATCACGGCCCTCTTACTCCTCCTATCACTACCAGTACTAGCAGGAGCAA

TCACAATACTACTAACCGACCGAAACCTAAACACATCCTTCTTCGACCCAGCAGGAGGTGGAGACCCAATCCTATATCAACACCTATTT--

----

>G10_Macrotermes_herus_2 Macrotermes herus isolate Mh03 cytochrome oxidase subunit I (COI) gene, partial cds; mitochondrial

----

AACACTATACTTCGTATTCGGAGCCTGATCAGGAATGGTTGGAACATCCCTAAGAATACTAATCCGAACAGAACTTGGACAACCAGGATCCCTAATTGGAGACGATCAAATCT

ACAACGTCATCGTCACAGCCCATGCTTTCGTAATGATCTTCTTCATAGTAATACCAATCATGATTGGAGGATTCGGAAACTGATTAGTACCGCTAATATTAGGAGCACCAGACA

TAGCATTCCCACGAATAAACAACATAAGATTTTGATTATTACCACCATCACTAACTCTTCTTCTTACTAGTAGAACAGTAGAAAGTGGTGCAGGAACAGGATGAACAGTGTACC

CTCCCCTTGCAAGAGGAATTGCCCACGCCGGAGCATCAGTAGACCTAGCCATCTTCTCACTACACTTAGCAGGAGTATCATCCATCCTAGGAGCAGTAAATTTCATTTCAACAA

CAATCAACATGAAACCAAAAAGCATAAAGCCTGAACGAATCCCCCTATTCGTATGATCAGTTGCCATCACGGCCCTCTTACTCCTCCTATCACTACCAGTACTAGCAGGAGCAA

TCACAATACTACTAACCGACCGAAACCTAAACACATCCTTTTTCGACCCAGCAGGAGGTGGAGACCCAATCCTATATCAACACCTATTT--

----

>G11_Macrotermes_subhyalinus_6 Macrotermes subhyalinus voucher P3To07 cytochrome oxidase subunit I (COI) gene, partial cds; mitochondrial

----

AAAGATATTGGAACACTATACTTCGTATTCGGGGCCTGATCAGGAATGGTTGGAACATCCCTAAGAATACTAATCCGAACAGAACTTGGACAACCAGGATCCCTAATTGGAGA

CGATCAAATCTACAACGTCATTGTCACAGCCCATGCTTTCGTAATGATCTTCTTCATAGTAATACCAATCATGATTGGAGGATTCGGGAACTGATTAGTACCGCTAATATTAGGA

GCACCAGACATAGCATTCCCACGAATAAACAACATAAGATTTTGATTATTACCACCATCACTAGCTCTTCTTCTTACTAGTAGAACAGTAGAAAGTGGTGCAGGAACAGGATGA

ACAGTGTACCCTCCCCTTGCAAGAGGAATTGCTCACGCTGGAGCATCAGTAGACCTAGCCATCTTCTCGCTACACTTAGCAGGAGTATCATCCATCCTAGGAGCAGTAAATTTC

ATTTCAACAACAATCAACATGAAACCAAAAAGCATAAAGCCCGAACGAATCCCCCTATTCGTATGATCAGTTGCCATCACAGCCCTGCTGCTCCTCCTATCACTACCAGTACTAG

CAGGAGCAATCACAATACTATTAACCGACCGAAACCTAAACACATCCTTCTTCGACCCAGCAGGAGGTGGA---

----

>G11_Macrotermes_subhyalinus_7 Macrotermes subhyalinus voucher Tri1Ms13 cytochrome oxidase subunit I (COI) gene, partial cds; mitochondrial

----

AAAGATATTGGAACACTATACTTCGTATTCGGGGCCTGATCAGGAATGGTTGGAACATCCCTAAGAATACTAATCCGAACAGAACTTGGACAACCAGGATCCCTAATTGGAGA

CGATCAAATCTACAACGTCATTGTCACAGCCCATGCTTTCGTAATGATCTTCTTCATAGTAATACCAATCATGATTGGAGGATTCGGGAACTGATTAGTACCGCTAATATTAGGA

GCACCAGACATAGCATTCCCACGAATAAACAACATAAGATTTTGATTATTACCACCATCACTAACTCTTCTTCTTACTAGTAGAACAGTAGAAAGTGGTGCAGGAACAGGATGA

ACAGTGTACCCTCCCCTTGCAAGAGGAATTGCTCACGCTGGAGCATCAGTAGACCTAGCCATCTTCTCGCTACACTTAGCAGGAGTATCATCCATCCTAGGAGCAGTAAATTTC

ATTTCAACAACAATCAACATGAAACCAAAAAGCATAAAGCCCGAACGAATCCCCCTATTCGTATGATCAGTTGCCATCACAGCCCTGCTGCTCCTCCTATCACTACCAGTACTAG

CAGGAGCAATCACAATACTATTAACCGACCGAAACCTAAACACATCCTTCTTCGACCCAGCAGGAGGTGGAGA--

----

>G11_Macrotermes_subhyalinus_8 Macrotermes subhyalinus voucher Tri1Ms28 cytochrome oxidase subunit I (COI) gene, partial cds; mitochondrial

----

AAAGATATTGGAACACTATACTTCGTATTCGGGGCCTGATCAGGAATGGTTGGAACATCCCTAAGAATACTAATCCGAACAGAACTTGGACAACCAGGATCCCTAATTGGAGA

CGATCAAATCTACAACGTCATTGTCACAGCCCATGCTTTCGTAATGATCTTCTTCATAGTAATACCAATCATGATTGGAGGATTCGGGAACTGATTAGTACCGCTAATATTAGGA

GCACCAGACATAGCATTCCCACGAATAAACAACATAAGATTTTGATTATTACCACCATCACTAACTCTTCTTCTTACTAGTAGAACAGTAGAAAGTGGTGCAGGAACAGGATGA

ACAGTGTACCCTCCCCTTGCAAGAGGAATTGCTCACGCTGGAGCATCAGTAGACCTAGCCATCTTCTCGCTACACTTAGCAGGAGTATCATCCATCCTAGGAGCAGTAAATTTC

ATTTCAACAACAATCAACATGAAACCAAAAAGCATAAAGCCCGAACGAATCCCCCTATTCGTATGATCAGTTGCCATCACAGCCCTGCTGCTCCTCCTATCACTACCAGTACTAG

CAGGAGCAATCACAATACTATTAACCGACCGAAACCTAAACACATCCTTCTTCGACCCAGCAGGAGGTGGAGA--

----

>G11_Macrotermes_subhyalinus_9 Macrotermes subhyalinus voucher Tri1Ms18 cytochrome oxidase subunit I (COI) gene, partial cds; mitochondrial

----

AAGATATTGGAACACTATACTTCGTATTCGGAGCCTGATCAGGAATGGTTGGAACATCCCTAAGAATACTAATCCGAACAGAACTTGGACAACCAGGATCCCTAATTGGAGAC

GATCAAATCTACAACGTCATCGTCACAGCCCATGCTTTCGTAATGATCTTCTTCATAGTAATACCAATCATGATTGGAGGATTCGGAAACTGATTAGTACCGCTAATATTAGGAG

CACCAGACATAGCATTCCCACGAATAAACAACATAAGATTTTGATTATTACCACCATCACTAACTCTTCTTCTTACTAGTAGAACAGTAGAAAGTGGTGCAGGAACAGGATGAA

CAGTGTACCCTCCCCTTGCAAGAGGAATTGCCCACGCCGGAGCATCAGTAGACCTAGCCATCTTCTCGCTACACTTAGCAGGAGTATCATCCATCCTAGGAGCAGTAAATTTCA

TTTCAACAACAATCAACATGAAACCAAAAAGCATAAAGCCTGAACGAATCCCCCTATTCGTATGATCAGTTGCCATCACGGCCCTGCTGCTCCTCCTATCACTACCAGTACTAGC

AGGAGCAATCACAATACTATTAACCGACCGAAACCTAAACACATCCTTCTTCGACCCAGCAGGAGGTGGAGA---

----

>G11_Macrotermes_subhyalinus_12 Macrotermes subhyalinus voucher BYU IGC IS95 cytochrome oxidase subunit I (COI) gene, partial cds; mitochondrial

----

AACACTATACTTCGTATTCGGAGCCTGATCAGGAATGGTTGGAACATCCCTAAGAATACTAATCCGAACAGAACTTGGACAACCAGGATCCCTAATTGGAGACGATCAAATCT

ACAACGTCATCGTCACAGCCCATGCTTTCGTAATGATCTTCTTCATAGTAATACCAATCATGATTGGAGGATTCGGAAACTGATTAGTACCGCTAATATTAGGAGCACCAGACA

TAGCATTCCCACGAATAAACAACATAAGATTTTGATTATTACCACCATCACTAACTCTTCTTCTTACTAGTAGAACAGTAGAAAGTGGTGCAGGAACAGGATGAACAGTGTACC

CTCCCCTTGCAAGAGGAATTGCTCACGCCGGAGCATCAGTAGACCTAGCCATCTTCTCGCTACACTTAGCAGGAGTATCATCCATCCTAGGAGCAGTAAATTTCATTTCAACAA

CAATCAACATGAAACCAAAAAGCATAAAGCCCGAACGAATCCCCCTATTCGTATGATCAGTTGCCATCACGGCTCTGCTGCTCCTTCTATCACTACCAGTACTAGCAGGAGCAA

TCACAATACTATTAACCGACCGAAACTTAAACACATCCTTCTTCGACCCAGCAGGAGGTGGAGACCCAATCCTATATCAACACTTATTTTGATTCTTCGGACACCCTGAAGTATA

CATTTTAATCCTACCAGGATTTGGTATAATCTCCCACATTATTTGTCACGAAAGAGGTAAAAAGGAAGCCTTTGGAAACCTGGGAATAATCTTTGCCATACTAGCAATTGGACT

ACTAGGATTTGTAGTATGAGCACATCACATGTTCACAGTAGGTATAGACGTTGATACACGAGCCTACTTTACATCAGCAACAATAATTATCGCAGTACCAACGGGAATTAAAAT

CTTCAGATGACTTGCAACCATATACGGAACTCGAATAACTTACAGAGCAGCATGCCTATGGGCCCTAGGATTTGTATTCCTATTCACAATAGGAGGTCTCACAGGGGTAGTCCT

AGCAAACTCATCAATCGAC--

>G11_Macrotermes_subhyalinus_mtgenome_-_COX1 Macrotermes subhyalinus mitochondrion, complete genome

----

TCACCCGATGATTTTTCTCAACTAATCACAAAGACATTGGAACACTATACTTCGTATTCGGAGCCTGATCAGGAATGGTTGGAACATCCCTAAGAATACTAATCCGAACAGAAC

TTGGACAACCAGGATCCCTAATTGGAGACGATCAAATCTACAACGTCATCGTCACAGCCCATGCTTTCGTAATGATCTTCTTCATAGTAATACCAATCATGATTGGAGGATTCG

GAAACTGATTAGTACCGCTAATATTAGGAGCACCAGACATAGCATTCCCACGAATAAACAACATAAGATTTTGATTATTACCACCATCACTAACTCTTCTTCTTACTAGTAGAAC

AGTAGAAAGTGGTGCAGGAACAGGATGAACAGTGTACCCTCCCCTTGCAAGAGGAATTGCTCACGCCGGAGCATCAGTAGACCTAGCCATCTTCTCGCTACACTTAGCAGGA

GTATCATCCATCCTAGGAGCAGTAAATTTCATTTCAACAACAATCAACATGAAACCAAAAAGCATAAAGCCCGAACGAATCCCCCTATTCGTATGATCAGTTGCCATCACGGCT

CTGCTGCTCCTTCTATCACTACCAGTACTAGCAGGAGCAATCACAATACTATTAACCGACCGAAACTTAAACACATCCTTCTTCGACCCAGCAGGAGGTGGAGACCCAATCCTA

TATCAACACTTATTTTGATTCTTCGGACACCCTGAAGTATACATTTTAATCCTACCAGGATTTGGTATAATCTCCCACATTATTTGTCACGAAAGAGGTAAAAAGGAAGCCTTTG

GAAACCTGGGAATAATCTTTGCCATACTAGCAATTGGACTACTAGGATTTGTAGTATGAGCACATCACATGTTCACAGTAGGTATAGACGTTGATACACGAGCCTACTTTACAT

CAGCAACAATAATTATCGCAGTACCAACGGGAATTAAAATCTTCAGATGACTTGCAACCATATACGGAACTCGAATAACTTACAGAGCAGCATGCCTATGGGCCCTAGGATTT

GTATTCCTATTCACAATAGGAGGTCTCACAGGGGTAGTCCTAGCAAACTCATCAATCGAC--

----

>G11_Macrotermes_subhyalinus_26 Macrotermes subhyalinus isolate IS095 cytochrome oxidase subunit I (COI) gene, partial cds; mitochondrial

----

AGAATACTAATCCGAACAGAACTTNNNNNNNNNNGGATCCCTAATTGGAGACGATCAAATCTACMMCGTCATCGTCACAGCCCATGCTTTCGTAATGATCTTCTTCAWAGTA

ATACCAATCATGATTGGAGGATTCGGAAACTGATTAGTACCGCTAATATTAGGAGCACCAGACATAGCATTCCCACGAATAAACAACATAAGATTTTGATTATTACCACCATCA

CTAACTCTTCTTCTTACTAGTAGAACAGTAGAAAGTGGTGCAGGAACAGGATGAACAGTGTACCCTCCCCTTGCAAGAGGAATTGCTCACGCCGGAGCATCAGTAGACCTAGC

CATCTTCTCGCTACACTTAGCAGGAGTATCATCCATCCTAGGAGCAGTAAATTTCATTTCAACAACAATCAACATGAAACCAAAAAGCATAAAGCCCGAACGAATCCCCCTATTC

GTATGATCAGTTGCCATCACGGCTCTGCTGCTCCTTCTATCACTACCAGTACTAGCAGGAGCAATCACAATACTATTAACCGACCGAAACTTAAACACATCCTTCTTCGACCCAG

CAGGAGGTGGAGACCCAATCCTATATCAACACTTATTTTGATTCTTCGGACACCCTGAAGTATACATTTTAATCCTACCAGGATTTGGTATAATCTCCCACATTATTTGTCACGA

AAGAGGTAAAAAGGAAGCCTTTGGAAACCTGGGAATAATCTTTGCCATACTAGCAATTGGACTACTAGGATTTGTAGTATGAGCACATCACATGTTCACAGTAGGTATAGACG
TTGATACACGAGCCTACTTTACATCAGCAACAATAATTATCGCAGTACCAACGGGAATTAAAATCTTCAGATGACTTGCAACCATATACGGAACTCGAATAACTTACAGAGCAG
CATGCCTATGGGCCCTAGGATTTGTATTCCTATTCACAATAGGAGGTCTCACAGGGGTAGTCCTAGCAAACTCATCAATCGAC--
----

>G11_Macrotermes_subhyalinus_23 Macrotermes subhyalinus voucher Tri4S51 cytochrome oxidase subunit I (COI) gene, partial cds; mitochondrial

----
AAAGATATAGGAACACTATACTTCGTATTCGGAGCCTGATCAGGAATGGCTGGAACATCCCTAAGAATACTAATCCGAACAGAACTTGGACAACCAGGATCCCTAATTGGAGA
CGATCAAATCTACAACGTCATCGTCACAGCCCATGCTTTCGTAATGATCTTCTTCATAGTAATACCAATCATGATTGGAGGATTCGGAAACTGATTAGTACCGCTAATATTAGGA
GCACCAGACATAGCATTCCCACGAATAAACAACATAAGATTTTGATTATTACCACCATCACTAACTCTTCTTCTTACTAGTAGAACAGTAGAAAGTGGTGCAGGAACAGGATGA
ACAGTGTACCCTCCCCTTGCAAGAGGAATTGCTCACGCCGGAGCATCAGTAGACCTAGCCATCTTCTCGCTACACTTAGCAGGAGTATCATCCATCCTAGGAGCAGTAAATTTC
ATTTCAACAACAATCAACATGAAACCAAAAAGCATAAAGCCCGAACGAATCCCCCTATTCGTATGATCAGTTGCCATCACGGCTCTGCTGCTCCTTCTATCACTACCAGTACTAG
CAGGAGCAATCACAATACTATTAACCGACCGAAACCTAAACACATCCTTCTTCGACCCAGCAGGAGGTGGAGACCCAATCCTATACCAACACTTATTTTGATTCTTCGGACACC
CCGAAGTATACATTTTAATCCTACCAGGATTTGGTATAATCTCCCACATTATTTGTCACGAAAGAGGTAAAAAGGAAGCCTTTGGAAACCTGGGAATAATCTTTGCCATACTAG
CAATTGGACTACTAGGATTTGTAGTATGAGCACATCACATGTTCACAGTAGGTATAGACGTTGATACACGAGCCTACTTTACATCAGCAACAATAATTATCGCAGTACCAACGG
GAATTAAAATCTTCAGATGACTTGCAACCATATACGGAACTCGAATAACTTACAGAGCAGCATGCCTATGGGCCCTAGGATTTGTATTCCTATTCACAATAGGAGGTCTCACAG
GGGTAGTCCTAGCAAACTCATCAATCGAC---

>G11_Macrotermes_subhyalinus_13 Macrotermes subhyalinus isolate Ms01 cytochrome oxidase subunit I (COI) gene, partial cds; mitochondrial

----

AACACTATACTTCGTATTCGGAGCCTGATCAGGAATGGTTGGAACATCCCTAAGAATACTAATCCGAACAGAACTTGGACAACCAGGATCCCTAATTGGAGACGACCAAATCT

ACAACGTCATCGTCACAGCCCATGCTTTCGTAATGATCTTCTTCATAGTAATACCAATCATGATTGGAGGATTCGGAAACTGATTAGTACCGCTAATATTAGGAGCACCAGACA

TAGCATTCCCACGAATAAACAACATAAGATTTTGATTATTACCACCATCACTAACTCTTCTTCTTACTAGTAGAACAGTAGAAAGTGGTGCAGGAACAGGATGAACAGTATACC

CTCCCCTTGCAAGAGGAATTGCTCACGCCGGAGCATCAGTAGACCTAGCCATCTTCTCGCTACACTTAGCAGGAGTATCATCCATCCTAGGAGCAGTAAATTTCATTTCAACAA

CAATCAACATGAAACCAAAAAGCATAAAGCCCGAACGAATCCCCCTATTCGTATGATCAGTTGCCATCACGGCCCTGCTGCTCCTCCTATCACTACCAGTACTAGCAGGAGCAA

TCACAATACTATTAACCGACCGAAACCTAAACACATCCTTCTTCGACCCAGCAGGAGGTGGAGACCCAATCCTATATCAACACTTATTT--

----

>G11_Macrotermes_subhyalinus_14 Macrotermes subhyalinus isolate Ms02 cytochrome oxidase subunit I (COI) gene, partial cds; mitochondrial

----

AACACTATACTTCGTATTCGGAGCCTGATCAGGAATGGTTGGAACATCCCTAAGAATACTAATCCGAACAGAACTTGGACAACCAGGATCCCTAATTGGAGACGACCAAATCT

ACAACGTCATCGTCACAGCCCATGCTTTCGTAATGATCTTCTTCATAGTAATACCAATCATGATTGGAGGATTCGGAAACTGATTAGTACCGCTAATATTAGGAGCACCAGACA

TAGCATTCCCACGAATAAACAACATAAGATTTTGATTATTACCACCATCACTAACTCTTCTTCTTACTAGTAGAACAGTAGAAAGTGGTGCAGGAACAGGATGAACAGTGTACC

CTCCCCTTGCAAGAGGAATTGCTCACGCCGGAGCATCAGTAGACCTAGCCATCTTCTCGCTACACTTAGCAGGAGTATCATCCATCCTAGGAGCAGTAAATTTCATTTCAACAA

CAATCAACATGAAACCAAAAAGCATAAAGCCCGAACGAATCCCCCTATTCGTATGATCAGTTGCCATCACGGCCCTGCTGCTCCTCCTATCACTACCAGTACTAGCAGGAGCAA

TCACAATACTATTAACCGACCGAAACCTAAACACATCCTTCTTCGACCCAGCAGGAGGTGGAGACCCAATCCTATATCAACACTTATTT--

----

>G11_Macrotermes_subhyalinus_15 Macrotermes subhyalinus isolate Ms03 cytochrome oxidase subunit I (COI) gene, partial cds; mitochondrial

----
AACACTATACTTCGTATTCGGAGCCTGATCAGGAATGGTTGGAACATCCCTAAGAATACTAATCCGAACAGAACTTGGACAACCAGGATCCCTAATTGGAGACGACCAAATCT
ACAACGTCATCGTCACAGCCCATGCTTTCGTAATGATCTTCTTCATAGTAATACCAATCATGATTGGAGGATTCGGAAACTGATTAGTACCGCTAATATTAGGAGCACCAGACA
TAGCATTCCCACGAATAAACAACATAAGATTTTGATTATTACCACCATCACTAACTCTTCTTCTTACTAGTAGAACAGTAGAAAGTGGTGCAGGAACAGGATGAACAGTGTACC
CTCCCCTTGCAAGAGGAATTGCTCACGCCGGAGCATCAGTAGACCTAGCCATCTTCTCGCTACACTTAGCAGGAGTATCATCCATCCTAGGAGCAGTAAATTTCATTTCAACAA
CAATCAACATGAAACCAAAAAGCATAAAGCCCGAACGAATCCCCCTATTCGTATGATCAGTTGCCATCACGGCCCTGCTGCTCCTCCTATCACTACCAGTACTAGCAGGAGCAA
TCACAATACTATTAACCGACCGAAACCTAAACACATCCTTCTTCGACCCAGCAGGAGGTGGAGACCCAATCCTATATCAACACTTATTT--
----

>G11_Macrotermes_subhyalinus_16 Macrotermes subhyalinus isolate Ms05 cytochrome oxidase subunit I (COI) gene, partial cds; mitochondrial

----
AACACTATACTTCGTATTCGGAGCCTGATCAGGAATGGTTGGAACATCCCTAAGAATACTAATCCGAACAGAACTTGGACAACCAGGATCCCTAATTGGAGACGACCAAATCT
ACAACGTCATCGTCACAGCCCATGCTTTCGTAATGATCTTCTTCATAGTAATACCAATCATGATTGGAGGATTCGGAAACTGATTAGTACCGCTAATATTAGGAGCACCAGACA
TAGCATTCCCACGAATAAACAACATAAGATTTTGATTATTACCACCATCACTAACTCTTCTTCTTACTAGTAGAACAGTAGAAAGTGGTGCAGGAACAGGATGAACAGTGTACC

CTCCCCTTGCAAGAGGAATTGCTCACGCCGGAGCATCAGTAGACCTAGCCATCTTCTCGCTACACTTAGCAGGAGTATCATCCATCCTAGGAGCAGTAAATTTCATTTCAACAA

CAATCAACATGAAACCAAAAAGCATAAAGCCCGAACGAATCCCCCTATTCGTATGATCAGTTGCCATCACGGCCCTGCTGCTCCTCCTATCACTACCAGTACTAGCAGGAGCAA

TCACAATACTATTAACCGACCGAAACCTAAACACATCCTTCTTCGACCCAGCAGGAGGTGGAGACCCAATCCTATATCAACACTTATTT--

----

>G11_Macrotermes_subhyalinus_17 Macrotermes subhyalinus isolate Ms06 cytochrome oxidase subunit I (COI) gene, partial cds; mitochondrial

----

AACACTATACTTCGTATTCGGAGCCTGATCAGGAATGGTTGGAACATCCCTAAGAATACTAATCCGAACAGAACTTGGACAACCAGGATCCCTAATTGGAGACGACCAAATCT

ACAACGTCATCGTCACAGCCCATGCTTTCGTAATGATCTTCTTCATAGTAATACCAATCATGATTGGAGGATTCGGAAACTGATTAGTACCGCTAATATTAGGAGCACCAGACA

TAGCATTCCCACGAATAAACAACATAAGATTTTGATTATTACCACCATCACTAACTCTTCTTCTTACTAGTAGAACAGTAGAAAGTGGTGCAGGAACAGGATGAACAGTGTACC

CTCCCCTTGCAAGAGGAATTGCTCACGCCGGAGCATCAGTAGACCTAGCCATCTTCTCGCTACACTTAGCAGGAGTATCATCCATCCTAGGAGCAGTAAATTTCATTTCAACAA

CAATCAACATGAAACCAAAAAGCATAAAGCCCGAACGAATCCCCCTATTCGTATGATCAGTTGCCATCACGGCCCTGCTGCTCCTCCTATCACTACCAGTACTAGCAGGAGCAA

TCACAATACTATTAACCGACCGAAACCTAAACACATCCTTCTTCGACCCAGCAGGAGGTGGAGACCCAATCCTATATCAACACTTATTT--

----

>G11_Macrotermes_subhyalinus_18 Macrotermes subhyalinus isolate Ms07 cytochrome oxidase subunit I (COI) gene, partial cds; mitochondrial

----

AACACTATACTTCGTATTCGGAGCCTGATCAGGAATGGTTGGAACATCCCTAAGAATACTAATCCGAACAGAACTTGGACAACCAGGATCCCTAATTGGAGACGACCAAATCT

ACAACGTCATCGTCACAGCCCATGCTTTCGTAATGATCTTCTTCATAGTAATACCAATCATGATTGGAGGATTCGGAAACTGATTAGTACCGCTAATATTAGGAGCACCAGACA

TAGCATTCCCACGAATAAACAACATAAGATTTTGATTATTACCACCATCACTAACTCTTCTTCTTACTAGTAGAACAGTAGAAAGTGGTGCAGGAACAGGATGAACAGTGTACC

CTCCCCTTGCAAGAGGAATTGCTCACGCCGGAGCATCAGTAGACCTAGCCATCTTCTCGCTACACTTAGCAGGAGTATCATCCATCCTAGGAGCAGTAAATTTCATTTCAACAA

CAATCAACATGAAACCAAAAAGCATAAAGCCCGAACGAATCCCCCTATTCGTATGATCAGTTGCCATCACGGCCCTGCTGCTCCTCCTATCACTACCAGTACTAGCAGGAGCAA

TCACAATACTATTAACCGACCGAAACCTAAACACATCCTTCTTCGACCCAGCAGGAGGTGGAGACCCAATCCTATATCAACACTTATTT---

----

>G11_Macrotermes_subhyalinus_19 Macrotermes subhyalinus isolate Ms09 cytochrome oxidase subunit I (COI) gene, partial cds; mitochondrial

----

AACACTATACTTCGTATTCGGAGCCTGATCAGGAATGGTTGGAACATCCCTAAGAATACTAATCCGAACAGAACTTGGACAACCAGGATCCCTAATTGGAGACGACCAAATCT

ACAACGTCATCGTCACAGCCCATGCTTTCGTAATGATCTTCTTCATAGTAATACCAATCATGATTGGAGGATTCGGAAACTGATTAGTACCGCTAATATTAGGAGCACCAGACA

TAGCATTCCCACGAATAAACAACATAAGATTTTGATTATTACCACCATCACTAACTCTTCTTCTTACTAGTAGAACAGTAGAAAGTGGTGCAGGAACAGGATGAACAGTGTACC

CTCCCCTTGCAAGAGGAATTGCTCACGCCGGAGCATCAGTAGACCTAGCCATCTTCTCGCTACACTTAGCAGGAGTATCATCCATCCTAGGAGCAGTAAATTTCATTTCAACAA

CAATCAACATGAAACCAAAAAGCATAAAGCCCGAACGAATCCCCCTATTCGTATGATCAGTTGCCATCACGGCCCTGCTGCTCCTCCTATCACTACCAGTACTAGCAGGAGCAA

TCACAATACTATTAACCGACCGAAACCTAAACACATCCTTCTTCGACCCAGCAGGAGGTGGAGACCCAATCCTATATCAACACTTATTT---

----

>G11_Macrotermes_subhyalinus_20 Macrotermes subhyalinus isolate Ms08 cytochrome oxidase subunit I (COI) gene, partial cds; mitochondrial

----

ANCACTATACTTNGTATTCGGAGCNTGATCAGGAANGGTTGGAACATCCCTAAGAATACTAATCCGAACAGAACTTGGACAACCAGGATCCCTAATTGGAGACGACCAAATC

TACAACGTCATCGTCACAGCCCATGCTTTCGTAATGATCTTCTTCATAGTAATACCAATCATGATTGGAGGATTCGGAAACTGATTAGTACCGCTAATATTAGGAGCACCAGAC

ATAGCATTCCCACGAATAAACAACATAAGATTTTGATTATTACCACCATCACTAACTCTTCTTCTTACTAGTAGAACAGTAGAAAGTGGTGCAGGAACAGGATGAACAGTGTAC

CCTCCCCTTGCAAGAGGAATTGCTCACGCCGGAGCATCAGTAGACCTAGCCATCTTCTCGCTACACTTAGCAGGAGTATCATCCATCCTAGGAGCAGTAAATTTCATTTCAACA

ACAATCAACATGAAACCAAAAAGCATAAAGCCCGAACGAATCCCCCTATTCGTATGATCAGTTGCCATCACGGCCCTGCTGCTCCTCCTATCACTACCAGTACTAGCAGGAGCA

ATCACAATACTATTAACCGACCGAAACCTAAACACATCCTTCTTCGACCCAGCAGGAGGTGGAGACCCAATCCTATATCAACACTTATTT---

----

>G11_Macrotermes_subhyalinus_21 Macrotermes subhyalinus isolate Ms04 cytochrome oxidase subunit I (COI) gene, partial cds; mitochondrial

----

AACACTATACTTCGTATTCGGAGCCTGATCAGGAATGGTTGGAACATCCCTAAGAATACTAATCCGAACAGAACTTGGACAACCAGGATCCCTAATTGGAGACGACCAAATCT

ACAACGTCATCGTCACAGCCCATGCTTTCGTAATGATCTTCTTCATAGTAATACCAATCATGATTGGAGGATTCGGAAACTGATTAGTACCGCTAATATTAGGAGCACCAGACA

TAGCATTCCCACGAATAAACAACATAAGATTTTGATTATTACCACCATCACTAACTCTTCTTCTTACTAGTAGAACAGTAGAAAGTGGTGCAGGAACAGGATGAACAGTGTACC

CTCCCCTTGCAAGAGGAATTGCTCACGCCGGAGCATCAGTAGACCTAGCCATCTTCTCGCTACACTTAGCAGGAGTATCATCCATCCTAGGAGCAGTAAATTTCATTTCAACAA

CAATCAACATGAAACCAAAAAACATAAAGCCCGAACGAATCCCCCTATTCGTATGATCAGTTGCCATCACGGCCCTGCTGCTCCTCCTATCACTACCAGTACTAGCAGGAGCAA

TCACAATACTATTAACCGACCGAAACCTAAACACATCCTTCTTCGACCCAGCAGGAGGTGGAGACCCAATCCTATATCAACACTTATTT--

----

>G11_Macrotermes_subhyalinus_22 Macrotermes subhyalinus isolate Ms10 cytochrome oxidase subunit I (COI) gene, partial cds; mitochondrial

----

AACACTATACTTCGTATTCGGAGCCTGATCAGGAATGGTTGGAACATCCCTAAGAATACTAATCCGAACAGAACTTGGACAACCAGGATCCCTAATTGGAGACGACCAAATCT

ACAACGTCATCGTCACAGCCCATGCTTTCGTAATGATCTTCTTCATAGTAATACCAATCATGATTGGAGGATTCGGAAACTGATTAGTACCGCTAATATTAGGAGCACCAGACA

TAGCATTCCCACGAATAAACAACATAAGATTTTGATTATTACCACCATCACTAACTCTTCTTCTTACTAGTAGAACAGTAGAAAGTGGTGCAGGAACAGGATGAACAGTGTACC

CTCCCCTTGCAAGAGGAATTGCTCACGCCGGAGCATCAGTAGACCTAGCCATCTTCTCGCTACACTTAGCAGGAGTATCATCCATCCTAGGAGCAGTAAATTTCATTTCAACAA

CAATCAACATGAAACCAAAAAACATAAAGCCCGAACGAATCCCCCTATTCGTATGATCAGTTGCCATCACGGCCCTGCTGCTCCTCCTATCACTACCAGTACTAGCAGGAGCAA

TCACAATACTATTAACCGACCGAAACCTAAACACATCCTTCTTCGACCCAGCAGGAGGTGGAGACCCAATCCTATATCAACACTTATTT--

----

>G10_Macrotermes_herus_3 Macrotermes herus isolate Mh02 cytochrome oxidase subunit I (COI) gene, partial cds; mitochondrial

----

AACCCTATACTTCGTATTTGGAGCATGATCAGGAATGGTNGGAACATCCCTAAGAATACTAATCCGAACAGAACTTGGACAACCAGGATCCCTAATTGGAGACGATCAAATCT

ACAACGTCATCGTCACAGCCCATGCTTTCGTAATGATCTTCTTCATAGTNATGCCAATCATGATTGGAGGATTCGGAAACTGATTAGTACCGNTAATATTAGGAGCACCAGACA

TAGCATTCCCACGAATAAACAACATAAGATTTTGATTATTACCGCCCTCACTAACTCTTCTTCTTNCTAGTAGAACAGTAGAAAGAGGTGCAGGAACAGGATGAACAGTGTANC

CTCCCCTTGCAAGAGGAATTGCCCACGCCGGAGCATCAGTAGACCTAGCCATCTTCTCCCTACACTTAGCAGGAGTATCATCCATCTTAGGAGCAGTAAATTTCATTTCAACAAC

AATCAACATGAAACCCAAAAGCATAAAGCCTGAACGAATCCCCCTATTCGTATGATCAGTTGCCATCACGGCCCTCTTACTCCTCCTATCACTACCAGTACTAGCAGGAGCAATC

ACAATACTACTAACCGACCGAAACCTAAACACATCCTTCTTCGANCCAGCAGGAGGNGGAGACCCAATCCTATATCAACACCTATTT--

----

>G7_FT3

TGATCAGGAATGGTTGGAACATCCCTAAGAATACTAATCCGAACAGAACTTGGACAACCAGGATCCTTAATTGGGGACGATCAAATCTACAACGTCATCGTCACAGCTCATGC

TTTCGTAATAATCTTCTTCATAGTAATACCAATCATGATTGGAGGATTCGGAAACTGACTAGTACCACTAATATTAGGAGCACCAGACATAGCATTCCCACGAATAAACAACAT

AAGATTCTGATTACTACCACCATCGCTAACTCTTCTTCTCACTAGTAGAACAGTAGAAAGTGGTGCAGGAACAGGATGAACAGTATACCCACCCCTTGCAAGAGGAATTGCTCA

CGCCGGAGCATCAGTAGACCTAGCCATCTTCTCATTACACTTAGCAGGAGTATCATCAATCCTAGGAGCAGTAAATTTCATTTCAACAACAATTAATATGAAACCAAAAAACAT

AAAGCCCGAACGAATCCCCCTATTCGTATGATCAGTCGCCATCACAGCCCTCCTACTCCTACTATCACTACCAGTACTAGCAGGAGCAATCACAATACTATTAACCGACCGAAAC

CTAAACACATCCTTCTTCGACCCAGCAGGAGGTGGAGATCCAATCCTATATCAACACTTATTCTGATTCTTCGGACACCC---

----

>G7_FT5

GGAATGGTTGGAACATCCCTAAGAATACTAATCCGAACAGAACTTGGACAACCAGGATCCTTAATTGGGGACGATCAAATCTACAACGTCATCGTCACAGCTCATGCTTTCGT

AATAATCTTCTTCATAGTAATACCAATCATGATTGGAGGATTCGGAAACTGACTAGTACCACTAATATTAGGAGCACCAGACATAGCATTCCCACGAATAAACAACATAAGATT

CTGATTACTACCACCATCGCTAACTCTTCTTCTCACTAGTAGAACAGTAGAAAGTGGTGCAGGAACAGGATGAACAGTATACCCACCCCTTGCAAGAGGAATTGCTCACGCCG

GAGCATCAGTAGACCTAGCCATCTTCTCATTACACTTAGCAGGAGTATCATCAATCCTAGGAGCAGTAAATTTCATTTCAACAACAATTAATATGAAACCAAAAAACATAAAGC

CCGAACGAATCCCCCTATTCGTATGATCAGTCGCCATCACAGCCCTCCTACTCCTACTATCACTACCAGTACTAGCAGGAGCAATCACAATACTATTAACCGACCGAAACCTAAA

CACATCCTTCTTCGACCCAGCAGGAGGTGGAGATCCAATCCTATATCAACACTTATTCTGATTCTTCGGACACC--

----

>G7_MF49

ATGGTTGGAACATCCCTAAGAATACTAATCCGAACAGAACTTGGACAACCAGGATCCTTAATTGGGGACGATCAAATCTACAACGTCATCGTCACAGCTCATGCTTTCGTAATA

ATCTTCTTCATAGTAATACCAATCATGATTGGAGGATTCGGAAACTGACTAGTACCACTAATATTAGGAGCACCAGACATAGCATTCCCACGAATAAACAACATAAGATTCTGA

TTACTACCACCATCGCTAACTCTTCTTCTCACTAGTAGAACAGTAGAAAGTGGTGCAGGAACAGGATGAACAGTATACCCACCCCTTGCAAGAGGAATTGCTCACGCCGGAGC

ATCAGTAGACCTAGCCATCTTCTCATTACACTTAGCAGGAGTATCATCAATCCTAGGAGCAGTAAATTTCATTTCAACAACAATTAATATGAAACCAAAAAACATAAAGCCCGA

ACGAATCCCCCTATTCGTATGATCAGTCGCCATCACAGCCCTCCTACTCCTACTATCACTACCAGTACTAGCAGGAGCAATCACAATACTATTAACCGACCGAAACCTAAACACA

TCCTTCTTCGACCCAGCAGGAGGTGGAGATCCAATCCTATATCAACACTTATTCTGATTCTTCGGACACCC--

----

>G7_MF51

ATGGTTGGAACATCCCTAAGAATACTAATCCGAACAGAACTTGGACAACCAGGATCCTTAATTGGGGACGATCAAATCTACAACGTCATCGTCACAGCTCATGCTTTCGTAATA

ATCTTCTTCATAGTAATACCAATCATGATTGGAGGATTCGGAAACTGACTAGTACCACTAATATTAGGAGCACCAGACATAGCATTCCCACGAATAAACAACATAAGATTCTGA

TTACTACCACCATCGCTAACTCTTCTTCTCACTAGTAGAACAGTAGAAAGTGGTGCAGGAACAGGATGAACAGTATACCCACCCCTTGCAAGAGGAATTGCTCACGCCGGAGC

ATCAGTAGACCTAGCCATCTTCTCATTACACTTAGCAGGAGTATCATCAATCCTAGGAGCAGTAAATTTCATTTCAACAACAATTAATATGAAACCAAAAAACATAAAGCCCGA

ACGAATCCCCCTATTCGTATGATCAGTCGCCATCACAGCCCTCCTACTCCTACTATCACTACCAGTACTAGCAGGAGCAATCACAATACTATTAACCGACCGAAACCTAAACACA

TCCTTCTTCGACCCAGCAGGAGGTGGAGATCCAATCCTATATCAACACTTATTCTGATTCTTCGGACACCC--

----

>G7_MF52

ATGGTTGGAACATCCCTAAGAATACTAATCCGAACAGAACTTGGACAACCAGGATCCTTAATTGGGGACGATCAAATCTACAACGTCATCGTCACAGCTCATGCTTTCGTAATA

ATCTTCTTCATAGTAATACCAATCATGATTGGAGGATTCGGAAACTGACTAGTACCACTAATATTAGGAGCACCAGACATAGCATTCCCACGAATAAACAACATAAGATTCTGA

TTACTACCACCATCGCTAACTCTTCTTCTCACTAGTAGAACAGTAGAAAGTGGTGCAGGAACAGGATGAACAGTATACCCACCCCTTGCAAGAGGAATTGCTCACGCCGGAGC

ATCAGTAGACCTAGCCATCTTCTCATTACACTTAGCAGGAGTATCATCAATCCTAGGAGCAGTAAATTTCATTTCAACAACAATTAATATGAAACCAAAAAACATAAAGCCCGA

ACGAATCCCCCTATTCGTATGATCAGTCGCCATCACAGCCCTCCTACTCCTACTATCACTACCAGTACTAGCAGGAGCAATCACAATACTATTAACCGACCGAAACCTAAACACA

TCCTTCTTCGACCCAGCAGGAGGTGGAGATCCAATCCTATATCAACACTTATTCTGATTCTTCGGACACCC--

----

>G7_MF50

GGAATGGTTGGAACATCCCTAAGAATACTAATCCGAACAGAACTTGGACAACCAGGATCCTTAATTGGGGACGATCAAATCTACAACGTCATCGTCACAGCTCATGCTTTCGT

AATAATCTTCTTCATAGTAATACCAATCATGATTGGAGGATTCGGAAACTGACTAGTACCACTAATATTAGGAGCACCAGACATAGCATTCCCACGAATAAACAACATAAGATT

CTGATTACTACCACCATCGYTAACTCTTCTTCTCACTAGTAGAACAGTAGAAAGTGGTGCAGGAACAGGATGAACAGTATACCCACCCCTTGCAAGAGGAATTGCTCACGCCG

GAGCATCAGTAGACCTAGCCATCTTCTCATTACACTTAGCAGGAGTATCATCAATCCTAGGAGCAGTAAATTTCATTTCAACAACAATTAATATGAAACCAAAAAACATAAAGC

CCGAACGAATCCCCCTATTCGTATGATCAGTCGCCATCACAGCCCTCCTACTCCTACTATCACTACCAGTACTAGCAGGAGCAATCACAATACTATTAACCGACCGAAACCTAAA

CACATCCTTCTTCGACCCAGCAGGAGGTGGAGATCCAATCCTATATCAACACTTATTCTGATTCTTCGGACACC---

----

>G7_Macrotermes_natalensis_2 Macrotermes natalensis strain za1 cytocrome oxidase subunit I gene, partial cds; mitochondrial

----
ATACTAATCCGAACAGAACTTGGACAACCAGGATCCTTAATTGGAGACGACCAAATCTACAACGTCATCGTCACAGCCCATGCTTTCGTAATAATCTTCTTCATAGTAATACCAA
TCATGATTGGAGGATTCGGAAACTGACTAGTACCGCTAATATTAGGAGCACCAGACATAGCATTCCCACGAATAAACAACATAAGATTCTGATTATTACCACCATCGCTAACTC
TTCTTCTCACTAGTAGAACAGTAGAAAGTGGTGCAGGAACAGGATGAACAGTATACCCACCCCTTGCAAGAGGAATTGCTCACGCCGGAGCATCAGTAGACCTAGCCATCTTC
TCATTACACTTAGCAGGAGTATCATCAATCCTAGGAGCAGTAAATTTCATTTCAACAACAATCAACATGAAGCCAAAAAACATAAAGCCCGAACGAATCCCCCTATTCGTATGA
TCAGTCGCCATCACGGCCCTCCTACTCCTACTATCACTACCAGTACTAGCAGGAGCAATCACAATACTACTAACCGACCGAAACCTAAACACATCCTTCTTCGACCCAGCAGGA
GGTGGTGACCCAATCCTATATCAACACTTATTTTGATTCTTCGGACACCCTGAAGTATACATCTTAATCCTACCAGGATTTGGTATAATCTCCCACATTATTTGTCACGAAAGAG
GTAAAAAGGAAGCCTTTGGAAACCTGGGAATAATCTTTGCCATACTAGCAATTGGATTACTAGGATTTGTAGTATGAGCACACCACATGTTCACAGTAGGAATAGACGTTGAT
ACACGAGCTTACTTTACATCAGCAACAATAATCATTGCAGTACCAACAGGAATCAAAATCTTCAGATGACTTGCAACCATATACGGAACTCGAATAACCTATAGAGCAGCATGC
CTATGGGCTCTAGGATTTGTATTCC--

>G7_Macrotermes_natalensis_mtgenome_-_COX1 Macrotermes natalensis mitochondrion, complete genome

----
TCACCCGATGATTTTTCTCAACTAATCACAAAGACATTGGAACACTATACTTTGTATTTGGAGCCTGATCAGGAATGGTTGGAACATCCCTAAGAATACTAATCCGAACAGAAC
TTGGACAACCAGGATCCTTAATTGGAGACGATCAAATCTACAACGTCATCGTCACAGCCCATGCTTTCGTAATAATCTTCTTCATAGTAATACCAATCATGATTGGAGGATTCGG

AAACTGACTAGTACCGCTAATATTAGGAGCACCAGACATAGCATTCCCACGAATAAACAACATAAGATTCTGATTATTACCACCATCGCTAACTCTTCTTCTCACTAGTAGAACA

GTAGAAAGTGGTGCAGGAACAGGATGAACAGTATACCCACCCCTTGCAAGAGGAATTGCTCACGCCGGAGCATCAGTAGACCTAGCCATCTTCTCATTACACTTAGCAGGAG

TATCATCAATCCTAGGAGCAGTAAATTTCATTTCAACAACAATCAACATGAAGCCAAAAAACATAAAGCCCGAACGAATCCCCCTATTCGTATGATCAGTCGCCATCACGGCCC

TCCTACTCCTACTATCACTACCAGTACTAGCAGGAGCAATCACAATACTACTAACCGACCGAAACCTAAACACATCCTTCTTCGACCCAGCAGGAGGTGGTGACCCAATCCTAT

ATCAACACTTATTTTGATTCTTCGGACACCCTGAAGTATATATCTTAATCCTACCAGGATTTGGTATAATCTCCCACATTATTTGTCACGAAAGAGGTAAAAAGGAAGCCTTTGG

AAACCTGGGAATAATCTTTGCCATACTAGCAATTGGATTACTAGGATTTGTAGTATGAGCACACCACATGTTCACAGTAGGAATAGACGTTGATACACGAGCTTACTTTACATC

AGCAACAATAATCATTGCAGTACCAACAGGAATCAAAATCTTCAGATGACTTGCAACCATATACGGAACTCGAATAACCTATAGAGCAGCATGCCTATGGGCTCTAGGATTTG

TATTCCTATTCACAATAGGAGGTCTCACAGGGGTAGTACTAGCAAATTCATCAATTGAT--

----

>G7_Macrotermes_natalensis_3 Macrotermes natalensis strain za136 cytocrome oxidase subunit I gene, partial cds; mitochondrial

----

ATACTAATCCGAACAGAACTTGGACAACCAGGATCCTTAATTGGGGACGATCAAATCTACAACGTCATCGTCACAGCTCATGCTTTCGTAATAATCTTCTTCATAGTAATACCAA

TCATGATTGGAGGATTCGGAAACTGACTAGTACCACTAATATTAGGAGCACCAGACATAGCATTCCCACGAATAAACAACATAAGATTCTGATTACTACCACCATCACTAACTC

TTCTTCTCACTAGTAGAACAGTAGAAAGTGGTGCAGGAACAGGATGAACAGTATACCCACCCCTTGCAAGAGGAATTGCTCACGCCGGAGCATCAGTAGACCTAGCCATCTTC

TCATTACACTTAGCAGGAGTATCATCAATCCTAGGAGCAGTAAATTTCATTTCAACAACAATTAATATGAAACCAAAAAACATAAAGCCCGAACGAATCCCCCTATTCGTATGAT

CAGTCGCCATCACAGCCCTCCTACTCCTACTATCACTACCAGTACTAGCAGGAGCAATCACAATACTATTAACCGACCGAAACCTAAACACATCCTTCTTCGACCCAGCAGGAG

GTGGAGACCCAATCCTATATCAACACTTATTCTGATTCTTCGGACACCCTGAAGTATATATCTTAATCCTACCAGGATTTGGTATAATCTCCCACATTATTTGTCACGAAAGAGG

TAAAAAGGAAGCCTTTGGAAACCTGGGAATAATCTTTGCCATACTAGCAATTGGATTACTAGGATTTGTAGTATGAGCACACCACATGTTCACAGTAGGAATAGACGTTGATA

CACGAGCTTACTTTACATCAGCAACAATAATCATTGCAGTACCAACGGGAATCAAAATCTTCAGATGACTTGCAACCATATACGGAACTCGAATAACTTATAGAGCAGCATGCC

TATGGGCCCTAGGATTTGTATTCC--

>G9_Macrotermes_subhyalinus_10 Macrotermes subhyalinus isolate dka5 cytocrome oxidase subunit I (COI) gene, partial cds; mitochondrial

----

ATACTAATCCGAACAGAACTTGGACAACCAGGATCCCTAATTGGAGACGATCAAATCTACAACGTCATCGTCACAGCCCATGCCTTCGTAATGATCTTCTTTATAGTAATACCAA

TCATGATTGGAGGATTCGGAAACTGACTAGTACCGCTAATATTAGGAGCACCAGACATAGCATTCCCCCGAATAAACAACATAAGATTTTGATTATTACCACCATCACTAACTC

TTCTTCTTACTAGTAGAACAGTAGAAAGTGGTGCAGGAACAGGATGAACAGTGTACCCTCCCCTTGCAAGAGGAATTGCTCACGCCGGAGCATCAGTAGACCTAGCCATCTTC

TCACTACACTTAGCAGGAGTATCATCCATCCTAGGAGCAGTAAATTTCATTTCAACAACAATCAACATGAAACCAAAAAACATAAAACCCGAACGAATCCCCCTATTCGTATGA

TCAGTTGCCATCACGGCCCTCCTACTCCTCCTATCACTACCAGTACTAGCAGGAGCAATTACAATACTATTAACCGACCGAAACCTAAACACATCCTTCTTCGACCCAGCAGGAG

GTGGAGACCCAATCCTATATCAACACTTATTTTGATTCTTCGGACACCCTGAAGTATACATTTTAATCCTACCAGGATTTGGTATAATCTCCCACATTATTTGTCACGAAAGAGGT

AAAAAGGAAGCCTTTGGAAACCTGGGAATAATCTTTGCCATACTAGCAATCGGACTACTAGGATTTGTAGTATGAGCACATCACATGTTCACAGTAGGGATAGACGTTGATAC

ACGAGCCTACTTTACATCAGCAACAATAATCATTGCAGTACCAACGGGAATTAAAATCTTCAGATGACTTGCAACCATATACGGAACCCGAATAACCTATAGAGCAGCATGTCT

ATGGGCCCTAGGATTTGTATTCC---

>G9_Macrotermes_subhyalinus_11 Macrotermes subhyalinus isolate dka64 cytocrome oxidase subunit I (COI) gene, partial cds; mitochondrial

----

ATACTAATCCGAACAGAACTTGGACAACCAGGATCCCTAATTGGAGACGATCAAATCTACAACGTCATCGTCACAGCCCATGCCTTCGTAATGATCTTCTTTATAGTAATACCAA

TCATGATTGGAGGATTCGGAAACTGACTAGTACCGCTAATATTAGGAGCACCAGACATAGCATTCCCCCGAATAAACAACATAAGATTTTGATTATTACCACCATCACTAACTC

TTCTTCTTACTAGTAGAACAGTAGAAAGTGGTGCAGGAACAGGATGAACAGTGTACCCTCCCCTTGCAAGAGGAATTGCTCACGCCGGAGCATCAGTAGACCTAGCCATCTTC

TCACTACACTTAGCAGGAGTATCATCCATCCTAGGAGCAGTAAATTTCATTTCAACAACAATCAACATGAAACCAAAAAACATAAAACCCGAACGAATCCCCCTATTCGTATGA

TCAGTTGCCATCACGGCCCTCCTACTCCTCCTATCACTACCAGTACTAGCAGGAGCAATTACAATACTATTAACCGACCGAAACCTAAACACATCCTTCTTCGACCCAGCAGGAG

GTGGAGACCCAATCCTATATCAACACTTATTTTGATTCTTCGGACACCCTGAAGTATACATTTTAATCCTACCAGGATTTGGTATAATCTCCCACATTATTTGTCACGAAAGAGGT

AAAAAGGAAGCCTTTGGAAACCTGGGAATAATCTTTGCCATACTAGCAATCGGACTACTAGGATTTGTAGTATGAGCACATCACATGTTCACAGTAGGGATAGACGTTGATAC

ACGAGCCTACTTTACATCAGCAACAATAATCATTGCAGTACCAACGGGAATTAAAATCTTCAGATGACTTGCAACCATATACGGAACCCGAATAACCTATAGAGCAGCATGTCT

ATGGGCCCTAGGATTTGTATTCC--

>G11_Macrotermes_subhyalinus_24 Macrotermes subhyalinus isolate N2m cytochrome oxidase subunit I (COI) gene, partial cds; mitochondrial

----

CCAGGATCCCTAATTGGAGACGACCAAATCTACAACGTCATCGTCACAGCCCATGCTTTCGTAATGATCTTCTTCATAGTAATACCAATCATGATTGGAGGATTCGGAAACTGA

TTAGTACCGCTAATATTAGGAGCACCAGACATAGCATTCCCACGAATAAACAACATAAGATTTTGATTATTACCACCATCACTAACTCTTCTTCTTACTAGTAGAACAGTAGAAA

GTGGTGCAGGAACAGGATGAACAGTGTACCCTCCCCTTGCAAGAGGAATCGCTCACGCCGGAGCATCAGTAGACCTAGCCATCTTCTCGCTACACTTAGCAGGAGTATCATCC

ATCCTAGGAGCAGTAAATTTCATTTCAACAACAATCAACATGAAACCAAAAAGCATAAAGCCCGAACGAATCCCCCTATTCGTATGATCAGTTGCCATCACGGCCCTGCTGCTC

CTCCTATCACTACCAGTACTAGCAGGAGCAATCACAATACTATTAACCGACCGAAACCTAAACACATCCTTCTTCGACCCAGCAGGAGGTGGAGACCCAATCCTATATCAACAC

TTATTTTGATTCTTCGGACACCCTGAAGTATACATTTTAATCCTACCAGGATTTGGTATAATCTCCCACATTATTTGTCACGAAAGAGGTAAAAAGGAAGCCTTTGGAAACCTGG

GAATAATCTTTGCCATACTAGCAATTGGACTACTAGGATTTGTAGTATGAGCACATCACATGTTCACAGTAGGTATAGACGTTGATACACGAGCCTACTTTACATCAGCAACAA

TAATTATTGCAGTACCAACGGGAATTAAAATCTTCAGATGACTTGCAACCATATACGGAACTCGAATAACTTATAGAGCAGCATGCCTATGAGCCCTAGGATTTGTATT----------

----

>G11_Macrotermes_subhyalinus_25 Macrotermes subhyalinus isolate K2m cytochrome oxidase subunit I (COI) gene, partial cds; mitochondrial

----

CCAGGATCCCTAATTGGAGACGATCAAATCTACAACGTCATTGTCACAGCCCATGCTTTCGTAATGATCTTCTTCATAGTAATACCAATCATGATTGGAGGATTCGGAAACTGAT
TAGTACCGCTAATATTAGGAGCACCAGACATAGCATTCCCACGAATAAACAACATAAGATTTTGATTATTACCACCATCACTAACTCTTCTTCTTACTAGTAGAACAGTAGAAAG
TGGTGCAGGAACAGGATGAACAGTGTACCCTCCCCTTGCAAGAGGAATTGCTCACGCTGGAGCATCAGTAGACCTAGCCATCTTCTCGCTACACTTAGCAGGAGTATCATCCA
TCCTAGGAGCAGTAAACTTCATTTCAACAACAATCAACATGAAACCAAAAAGCATAAAGCCCGAACGAATCCCCCTATTCGTATGATCAGTTGCCATCACAGCCCTGCTGCTCC
TCCTATCACTACCAGTACTAGCAGGAGCAATCACAATACTATTAACCGACCGAAACCTAAACACATCCTTCTTCGACCCAGCAGGAGGTGGAGACCCAATCCTATATCAACACT
TATTTTGATTCTTCGGACACCCTGAAGTATACATTTTAATCCTACCAGGATTTGGTATAATCTCCCACATTATTTGTCACGAAAGAGGTAAAAAGGAAGCCTTTGGAAACCTAGG
AATAATCTTTGCCATACTAGCAATTGGACTACTAGGATTTGTAGTATGAGCACATCACATGTTCACAGTAGGTATAGACGTTGATACACGAGCCTACTTTACATCAGCAACAAT
AATTATTGCAGTACCAACGGGAATTAAAATCTTCAGATGACTTGCAACCATATACGGAACTCGAATAACTTATAGAGCAGCATGCCTATGGGCCCTAGGATTTGTATT-----------
----

>G3_FT1

GATCAGGAATGGTTGGAACATCCCTAAGAATATTAATCCGAACAGAACTTGGACAACCAGGATCATTAATTGGGGACGACCAAATCTACAACGTCATCGTCACAGCTCACGCT
TTCGTAATAATCTTCTTCATAGTAATACCAATCATGATTGGAGGATTCGGAAACTGACTAGTACCGCTAATATTAGGAGCACCAGACATAGCATTCCCTCGAATAAACAACATA

AGATTCTGATTATTACCACCATCATTAACTCTTCTTCTCACTAGTAGAACAGTAGAAAGTGGTGCAGGAACAGGATGAACAGTATACCCACCCCTTGCAAGAGGAATTGCTCAC

GCCGGAGCATCAGTAGATCTAGCCATCTTCTCATTACACTTAGCAGGAGTATCATCAATCCTAGGAGCAGTAAACTTCATTTCAACAACAATCAACATGAAACCAAAAAACATA

AAGCCCGAACGAATCCCCTTATTCGTATGATCAGTTGCCATCACAGCTCTCCTACTCCTCCTATCACTACCAGTACTAGCAGGAGCAATCACAATATTATTAACTGACCGAAACC

TAAATACATCCTTCTTCGATCCAGCAGGAGGTGGAGACCCAATTCTATACCAACACCTATTTTGATTCTTCGGACACCC---

----

>G3_MF39

GATCAGGAATGGTTGGAACATCCCTAAGAATATTAATCCGAACAGAACTTGGACAACCAGGATCATTAATTGGGGACGACCAAATCTACAACGTCATCGTCACAGCTCACGCT

TTCGTAATAATCTTCTTCATAGTAATACCAATCATGATTGGAGGATTCGGAAACTGACTAGTACCGCTAATATTAGGAGCACCAGACATAGCATTCCCTCGAATAAACAACATA

AGATTCTGATTATTACCACCATCATTAACTCTTCTTCTCACTAGTAGAACAGTAGAAAGTGGTGCAGGAACAGGATGAACAGTATACCCACCCCTTGCAAGAGGAATTGCTCAC

GCCGGAGCATCAGTAGATCTAGCCATCTTCTCATTACACTTAGCAGGAGTATCATCAATCCTAGGAGCAGTAAACTTCATTTCAACAACAATCAACATGAAACCAAAAAACATA

AAGCCCGAACGAATCCCCTTATTCGTATGATCAGTTGCCATCACAGCTCTCCTACTCCTCCTATCACTACCAGTACTAGCAGGAGCAATCACAATATTATTAACTGACCGAAACC

TAAATACATCCTTCTTCGATCCAGCAGGAGGTGGAGACCCAATTCTATACCAACACCTATTTTGATTCTTCGGACACCC---

----

>G3_MF42

GATCAGGAATGGTTGGAACATCCCTAAGAATATTAATCCGAACAGAACTTGGACAACCAGGATCATTAATTGGGGACGACCAAATCTACAACGTCATCGTCACAGCTCACGCT

TTCGTAATAATCTTCTTCATAGTAATACCAATCATGATTGGAGGATTCGGAAACTGACTAGTACCGCTAATATTAGGAGCACCAGACATAGCATTCCCTCGAATAAACAACATA

AGATTCTGATTATTACCACCATCATTAACTCTTCTTCTCACTAGTAGAACAGTAGAAAGTGGTGCAGGAACAGGATGAACAGTATACCCACCCCTTGCAAGAGGAATTGCTCAC

GCCGGAGCATCAGTAGATCTAGCCATCTTCTCATTACACTTAGCAGGAGTATCATCAATCCTAGGAGCAGTAAACTTCATTTCAACAACAATCAACATGAAACCAAAAAACATA

AAGCCCGAACGAATCCCCTTATTCGTATGATCAGTTGCCATCACAGCTCTCCTACTCCTCCTATCACTACCAGTACTAGCAGGAGCAATCACAATATTATTAACTGACCGAAACC

TAAATACATCCTTCTTCGATCCAGCAGGAGGTGGAGACCCAATTCTATACCAACACCTATTTTGATTCTTCGGACACCC--

----

>G3_MF43

GATCAGGAATGGTTGGAACATCCCTAAGAATATTAATCCGAACAGAACTTGGACAACCAGGATCATTAATTGGGGACGACCAAATCTACAACGTCATCGTCACAGCTCACGCT

TTCGTAATAATCTTCTTCATAGTAATACCAATCATGATTGGAGGATTCGGAAACTGACTAGTACCGCTAATATTAGGAGCACCAGACATAGCATTCCCTCGAATAAACAACATA

AGATTCTGATTATTACCACCATCATTAACTCTTCTTCTCACTAGTAGAACAGTAGAAAGTGGTGCAGGAACAGGATGAACAGTATACCCACCCCTTGCAAGAGGAATTGCTCAC

GCCGGAGCATCAGTAGATCTAGCCATCTTCTCATTACACTTAGCAGGAGTATCATCAATCCTAGGAGCAGTAAACTTCATTTCAACAACAATCAACATGAAACCAAAAAACATA

AAGCCCGAACGAATCCCCTTATTCGTATGATCAGTTGCCATCACAGCTCTCCTACTCCTCCTATCACTACCAGTACTAGCAGGAGCAATCACAATATTATTAACTGACCGAAACC

TAAATACATCCTTCTTCGATCCAGCAGGAGGTGGAGACCCAATTCTATACCAACACCTATTTTGATTCTTCGGACACCC--

----

>G3_Macrotermes_falciger_mtgenome_-_COX1_ Macrotermes falciger mitochondrion, complete genome

----

TCACCCGATGATTTTTCTCAACTAATCATAAAGACATTGGAACACTATACTTCGTATTTGGAGCCTGATCAGGAATGGTTGGAACATCCCTAAGAATATTAATCCGAACAGAACT

TGGACAACCAGGATCATTAATTGGGGACGACCAAATCTACAACGTCATCGTCACAGCTCACGCTTTCGTAATAATCTTCTTCATAGTAATACCAATCATGATTGGAGGATTCGG

AAACTGACTAGTACCGCTAATATTAGGAGCACCAGACATAGCATTCCCTCGAATAAACAACATAAGATTCTGATTATTACCACCATCATTAACTCTTCTTCTCACTAGTAGAACA

GTAGAAAGTGGTGCAGGAACAGGATGAACAGTATACCCACCCCTTGCAAGAGGAATTGCTCACGCCGGAGCATCAGTAGATCTAGCCATCTTCTCATTACACTTAGCAGGAGT

ATCATCAATCCTAGGAGCAGTAAACTTCATTTCAACAACAATCAACATGAAACCAAAAAACATAAAGCCCGAACGAATCCCCTTATTCGTATGATCAGTTGCCATCACAGCTCTC

CTACTCCTCCTATCACTACCAGTACTAGCAGGAGCAATCACAATATTATTAACTGACCGAAACCTAAATACATCCTTCTTCGATCCAGCAGGAGGTGGAGACCCAATTCTATACC

AACACCTATTTTGATTCTTCGGACACCCCGAAGTATATATTTTAATCCTACCAGGATTTGGTATAATTTCCCACATTATTTGCCACGAAAGAGGTAAAAAGGAAGCCTTCGGAAA

CCTGGGAATAATTTTTGCCATATTAGCAATCGGATTATTAGGATTTGTAGTATGAGCACACCACATGTTCACAGTAGGAATAGATGTTGACACACGAGCTTACTTTACATCAGC

AACAATAATTATCGCAGTACCTACAGGGATTAAAATCTTCAGATGACTTGCAACAATATACGGAACCCGAATAACTTATAGAGCAGCATGCCTATGGGCCCTAGGATTTGTATT

CCTATTCACAATAGGAGGTCTTACAGGAGTAGTCCTAGCAAACTCATCAATTGAT---

>G3_MF2

ATGGTTGGAACATCCCTAAGAATATTAATCCGAACAGAACTTGGACAACCAGGATCATTAATTGGGGACGACCAAATCTACAACGTCATCGTCACAGCTCACGCTTTCGTAATA

ATCTTCTTCATAGTAATACCAATCATGATTGGAGGATTCGGAAACTGACTAGTACCGCTAATATTAGGAGCACCAGACATAGCATTCCCTCGAATAAACAACATAAGATTCTGA

TTATTACCACCATCATTAACTCTTCTTCTCACTAGTAGAACAGTAGAAAGTGGTGCAGGAACAGGATGAACAGTATACCCACCCCTTGCAAGAGGAATTGCTCACGCCGGAGC

ATCAGTAGATCTAGCCATCTTCTCATTACACTTAGCAGGAGTATCATCAATCCTAGGAGCAGTAAACTTCATTTCAACAACAATCAACATGAAACCAAAAAACATAAAGCCCGA

ACGAATCCCCTTATTCGTATGATCAGTTGCCATCACAGCTCTCCTACTCCTCCTATCACTACCAGTACTAGCAGGAGCAATCACAATATTATTAACTGACCGAAACCTAAATACAT

CCTTCTTCGATCCAGCAGGAGGTGGAGACCCAATTCTATACCAACACCTATTTTGATTCTTCGGACACC--

----

>G3_MF5

ATGGTTGGAACATCCCTAAGAATATTAATCCGAACAGAACTTGGACAACCAGGATCATTAATTGGGGACGACCAAATCTACAACGTCATCGTCACAGCTCACGCTTTCGTAATA

ATCTTCTTCATAGTAATACCAATCATGATTGGAGGATTCGGAAACTGACTAGTACCGCTAATATTAGGAGCACCAGACATAGCATTCCCTCGAATAAACAACATAAGATTCTGA

TTATTACCACCATCATTAACTCTTCTTCTCACTAGTAGAACAGTAGAAAGTGGTGCAGGAACAGGATGAACAGTATACCCACCCCTTGCAAGAGGAATTGCTCACGCCGGAGC

ATCAGTAGATCTAGCCATCTTCTCATTACACTTAGCAGGAGTATCATCAATCCTAGGAGCAGTAAACTTCATTTCAACAACAATCAACATGAAACCAAAAAACATAAAGCCCGA

ACGAATCCCCTTATTCGTATGATCAGTTGCCATCACAGCTCTCCTACTCCTCCTATCACTACCAGTACTAGCAGGAGCAATCACAATATTATTAACTGACCGAAACCTAAATACAT

CCTTCTTCGATCCAGCAGGAGGTGGAGACCCAATTCTATACCAACACCTATTTTGATTCTTCGGACACCC---

----

>G3_MF41

TGATCAGGAATGGTTGGAACATCCCTAAGAATATTAATCCGAACAGAACTTGGACAACCAGGATCATTAATTGGGGACGACCAAATCTACAACGTCATCGTCACAGCTCACGC

TTTCGTAATAATCTTCTTCATAGTAATACCAATCATGATTGGAGGATTCGGAAACTGACTAGTACCGCTAATATTAGGAGCACCAGACATAGCATTCCCTCGAATAAACAACAT

AAGATTCTGATTATTACCACCATCATTAACTCTTCTTCTCACTAGTAGAACAGTAGAAAGTGGTGCAGGAACAGGATGAACAGTATACCCACCCCTTGCAAGAGGAATTGCTCA

CGCCGGAGCATCAGTAGATCTAGCCATCTTCTCATTACACTTAGCAGGAGTATCATCAATCCTAGGAGCAGTAAACTTCATTTCAACAACAATCAACATGAAACCAAAAAACAT

AAAGCCCGAACGAATCCCCTTATTCGTATGATCAGTTGCCATCACAGCTCTCCTACTCCTCCTATCACTACCAGTACTAGCAGGAGCAATCACAATATTATTAACTGACCGAAAC

CTAAATACATCCTTCTTCGATCCAGCAGGAGGTGGAGACCCAATTCTATACCAACACCTATTTTGATTCTTCGGACACCC--

----

>G3_FT6

TGATCAGGAATGGTTGGAACATCCCTAAGAATATTAATCCGAACAGAACTTGGACAACCAGGATCATTAATTGGGGACGACCAAATCTACAACGTCATCGTCACAGCTCACGC

TTTCGTAATAATCTTCTTCATAGTAATACCAATCATGATTGGAGGATTCGGAAACTGACTAGTACCGCTAATATTAGGAGCACCAGACATAGCATTCCCTCGAATAAACAACAT

AAGATTCTGATTATTACCACCATCATTAACTCTTCTTCTCACTAGTAGAACAGTAGAAAGTGGTGCAGGAACAGGATGAACAGTATACCCACCCCTTGCAAGAGGAATTGCTCA

CGCCGGAGCATCAGTAGATCTAGCCATCTTCTCATTACACTTAGCAGGAGTATCATCAATCCTAGGAGCAGTAAACTTCATTTCAACAACAATCAACATGAAACCAAAAAACAT

AAAGCCCGAACGAATCCCCTTATTCGTATGATCAGTTGCCATCACAGCTCTCCTACTCCTCCTATCACTACCAGTACTAGCAGGAGCAATCACAATATTATTAACTGACCGAAAC

CTAAATACATCCTTCTTCGATCCAGCAGGAGGTGGAGACCCAATTCTATACCAACACCTATTTTGATTCTTCGGACAC---

----

>G3_MF40

TGATCAGGAATGGTTGGAACATCCCTAAGAATATTAATCCGAACAGAACTTGGACAACCAGGATCATTAATTGGGGACGACCAAATCTACAACGTCATCGTCACAGCTCACGC

TTTCGTAATAATCTTCTTCATAGTAATACCAATCATGATTGGAGGATTCGGAAACTGACTAGTACCGCTAATATTAGGAGCACCAGACATAGCATTCCCTCGAATAAACAACAT

AAGATTCTGATTATTACCACCATCATTAACTCTTCTTCTCACTAGTAGAACAGTAGAAAGTGGTGCAGGAACAGGATGAACAGTATACCCACCCCTTGCAAGAGGAATTGCTCA

CGCCGGAGCATCAGTAGATCTAGCCATCTTCTCATTACACTTAGCAGGAGTATCATCAATCCTAGGAGCAGTAAACTTCATTTCAACAACAATCAACATGAAACCAAAAAACAT

AAAGCCCGAACGAATCCCCTTATTCGTATGATCAGTTGCCATCACAGCTCTCCTACTCCTCCTATCACTACCAGTACTAGCAGGAGCAATCACAATATTATTAACTGACCGAAAC

CTAAATACATCCTTCTTCGATCCAGCAGGAGGTGGAGACCCAATTCTATACCAACACCTATTTTGATTCTTCGGACAC---

----

>G3_MF6

GATCAGGAATGGTTGGAACATCCCTAAGAATATTAATCCGAACAGAACTTGGACAACCAGGATCATTAATTGGGGACGACCAAATCTACAACGTCATCGTCACAGCTCACGCT

TTCGTAATAATCTTCTTCATAGTAATACCAATCATGATTGGAGGATTCGGAAACTGACTAGTACCGCTAATATTAGGAGCACCAGACATAGCATTCCCTCGAATAAACAACATA

AGATTCTGATTATTACCACCATCATTAACTCTTCTTCTCACTAGTAGAACAGTAGAAAGTGGTGCAGGAACAGGATGAACAGTATACCCACCCCTTGCAAGAGGAATTGCTCAC

GCCGGAGCATCAGTAGATCTAGCCATCTTCTCATTACACTTAGCAGGAGTATCATCAATCCTAGGAGCAGTAAACTTCATTTCAACAACAATCAACATGAAACCAAAAAACATA

AAGCCCGAACGAATCCCCTTATTCGTATGATCAGTTGCCATCACAGCTCTCCTACTCCTCCTATCACTACCAGTACTAGCAGGAGCAATCACAATATTATTAACTGACCGAAACC

TAAATACATCCTTCTTCGATCCAGCAGGAGGTGGAGACCCAATTCTATACCAACACCTATTTTGATTCTTCGGACA--

----

>G3_MF7

GGAGCTTGATCAGGAATGGTTGGAACATCCCTAAGAATATTAATCCGAACAGAACTTGGACAACCAGGATCATTAATTGGGGACGACCAAATCTACAACGTCATCGTCACAGC

TCACGCTTTCGTAATAATCTTCTTCATAGTAATACCAATCATGATTGGAGGATTCGGAAACTGACTAGTACCGCTAATATTAGGAGCACCAGACATAGCATTCCCTCGAATAAAC

AACATAAGATTCTGATTATTACCACCATCATTAACTCTTCTTCTCACTAGTAGAACAGTAGAAAGTGGTGCAGGAACAGGATGAACAGTATACCCACCCCTTGCAAGAGGAATT

GCTCACGCCGGAGCATCAGTAGATCTAGCCATCTTCTCATTACACTTAGCAGGAGTATCATCAATCCTAGGAGCAGTAAACTTCATTTCAACAACAATCAACATGAAACCAAAA

AACATAAAGCCCGAACGAATCCCCTTATTCGTATGATCAGTTGCCATCACAGCTCTCCTACTCCTCCTATCACTACCAGTACTAGCAGGAGCAATCACAATATTATTAACTGACC

GAAACCTAAATACATCCTTCTTCGATCCAGCAGGAGGTGGAGACCCAATTCTATACCAACACCTATTTTGATTCTTCGGACACCC--

----

>G3_MF8

GGAGCTTGATCAGGAATGGTTGGAACATCCCTAAGAATATTAATCCGAACAGAACTTGGACAACCAGGATCATTAATTGGGGACGACCAAATCTACAACGTCATCGTCACAGC

TCACGCTTTCGTAATAATCTTCTTCATAGTAATACCAATCATGATTGGAGGATTCGGAAACTGACTAGTACCGCTAATATTAGGAGCACCAGACATAGCATTCCCTCGAATAAAC

AACATAAGATTCTGATTATTACCACCATCATTAACTCTTCTTCTCACTAGTAGAACAGTAGAAAGTGGTGCAGGAACAGGATGAACAGTATACCCACCCCTTGCAAGAGGAATT

GCTCACGCCGGAGCATCAGTAGATCTAGCCATCTTCTCATTACACTTAGCAGGAGTATCATCAATCCTAGGAGCAGTAAACTTCATTTCAACAACAATCAACATGAAACCAAAA

AACATAAAGCCCGAACGAATCCCCTTATTCGTATGATCAGTTGCCATCACAGCTCTCCTACTCCTCCTATCACTACCAGTACTAGCAGGAGCAATCACAATATTATTAACTGACC

GAAACCTAAATACATCCTTCTTCGATCCAGCAGGAGGTGGAGACCCAATTCTATACCAACACCTATTTTGATTCTTCGGACACC--

----

>G3_MF1

GGAGCTTGATCAGGAATGGTTGGAACATCCCTAAGAATATTAATCCGAACAGAACTTGGACAACCAGGATCATTAATTGGGGACGACCAAATCTACAACGTCATCGTCACAGC

TCACGCTTTCGTAATAATCTTCTTCATAGTAATACCAATCATGATTGGAGGATTCGGAAACTGACTAGTACCGCTAATATTAGGAGCACCAGACATAGCATTCCCTCGAATAAAC

AACATAAGATTCTGATTATTACCACCATCATTAACTCTTCTTCTCACTAGTAGAACAGTAGAAAGTGGTGCAGGAACAGGATGAACAGTATACCCACCCCTTGCAAGAGGAATT

GCTCACGCCGGAGCATCAGTAGATCTAGCCATCTTCTCATTACACTTAGCAGGAGTATCATCAATCCTAGGAGCAGTAAACTTCATTTCAACAACAATCAACATGAAACCAAAA

AACATAAAGCCCGAACGAATCCCCTTATTCGTATGATCAGTTGCCATCACAGCTCTCCTACTCCTCCTATCACTACCAGTACTAGCAGGAGCAATCACAATATTATTAACTGACC

GAAACCTAAATACATCCTTCTTCGATCCAGCAGGAGGTGGAGACCCAATTCTATA---

>G3_MF9

GGAGCTTGATCAGGAATGGTTGGAACATCCCTAAGAATATTAATCCGAACAGAACTTGGACAACCAGGATCATTAATTGGGGACGACCAAATCTACAACGTCATCGTCACAGC

TCACGCTTTCGTAATAATCTTCTTCATAGTAATACCAATCATGATTGGAGGATTCGGAAACTGACTAGTACCGCTAATATTAGGAGCACCAGACATAGCATTCCCTCGAATAAAC

AACATAAGATTCTGATTATTACCACCATCATTAACTCTTCTTCTCACTAGTAGAACAGTAGAAAGTGGTGCAGGAACAGGATGAACAGTATACCCACCCCTTGCAAGAGGAATT

GCTCACGCCGGAGCATCAGTAGATCTAGCCATCTTCTCATTACACTTAGCAGGAGTATCATCAATCCTAGGAGCAGTAAACTTCATTTCAACAACAATCAACATGAAACCAAAA

AACATAAAGCCCGAACGAATCCCCTTATTCGTATGATCAGTTGCCATCACAGCTCTCCTACTCCTCCTATCACTACCAGTACTAGCAGGAGCAATCACAATATTATTAACTGACC

GAAACCTAAATACATCCTTCTTCGATCCAGCAGGAGGTGGAGACCCAATTCTATACCAACACCTATTTTGATTCTTCGGACA---

----

>G3_MF10

GGAGCTTGATCAGGAATGGTTGGAACATCCCTAAGAATATTAATCCGAACAGAACTTGGACAACCAGGATCATTAATTGGGGACGACCAAATCTACAACGTCATCGTCACAGC

TCACGCTTTCGTAATAATCTTCTTCATAGTAATACCAATCATGATTGGAGGATTCGGAAACTGACTAGTACCGCTAATATTAGGAGCACCAGACATAGCATTCCCTCGAATAAAC

AACATAAGATTCTGATTATTACCACCATCATTAACTCTTCTTCTCACTAGTAGAACAGTAGAAAGTGGTGCAGGAACAGGATGAACAGTATACCCACCCCTTGCAAGAGGAATT

GCTCACGCCGGAGCATCAGTAGATCTAGCCATCTTCTCATTACACTTAGCAGGAGTATCATCAATCCTAGGAGCAGTAAACTTCATTTCAACAACAATCAACATGAAACCAAAA

AACATAAAGCCCGAACGAATCCCCTTATTCGTATGATCAGTTGCCATCACAGCTCTCCTACTCCTCCTATCACTACCAGTACTAGCAGGAGCAATCACAATATTATTAACTGACC

GAAACCTAAATACATCCTTCTTCGATCCAGCAGGAGGTGGAGACCCAATTCTATACCAACACCTATTTTGATTCTTCGGAC--

----

>G3_MF3

ATGGTTGGAACATCCCTAAGAATATTAATCCGAACAGAACTTGGACAACCAGGATCATTAATTGGGGACGACCAAATCTACAACGTCATCGTCACAGCTCACGCTTTCGTAATA

ATCTTCTTCATAGTAATACCAATCATGATTGGAGGATTCGGAAACTGACTAGTACCGCTAATATTAGGAGCACCAGACATAGCATTCCCTCGAATAAACAACATAAGATTCTGA

TTATTACCACCATCATTAACTCTTCTTCTCACTAGTAGAACAGTAGAAAGTGGTGCAGGAACAGGATGAACAGTATACCCACCCCTTGCAAGAGGAATTGCTCACGCCGGAGC

ATCAGTAGATCTAGCCATCTTCTCATTACACTTAGCAGGAGTATCATCAATCCTAGGAGCAGTAAACTTCATTTCAACAACAATCAACATGAAACCAAAAAACATAAAGCCCGA

ACGAATCCCCTTATTCGTATGATCAGTTGCCATCACAGCTCTCCTACTCCTCCTATCACTACCAGTACTAGCAGGAGCAATCACAATATTATTAACTGACCGAAACCTAAATACAT

CCTTCTTCGATCCAGCAGGAGGTGGAGACCCAATTCTATACCAACACCTATTTTGATTCTTCGGACACCCCGAAGTATAA--

----

>G3_MF4

ATGGTTGGAACATCCCTAAGAATATTAATCCGAACAGAACTTGGACAACCAGGATCATTAATTGGGGACGACCAAATCTACAACGTCATCGTCACAGCTCACGCTTTCGTAATA
ATCTTCTTCATAGTAATACCAATCATGATTGGAGGATTCGGAAACTGACTAGTACCGCTAATATTAGGAGCACCAGACATAGCATTCCCTCGAATAAACAACATAAGATTCTGA
TTATTACCACCATCATTAACTCTTCTTCTCACTAGTAGAACAGTAGAAAGTGGTGCAGGAACAGGATGAACAGTATACCCACCCCTTGCAAGAGGAATTGCTCACGCCGGAGC
ATCAGTAGATCTAGCCATCTTCTCATTACACTTAGCAGGAGTATCATCAATCCTAGGAGCAGTAAACTTCATTTCAACAACAATCAACATGAAACCAAAAAACATAAAGCCCGA
ACGAATCCCCTTATTCGTATGATCAGTTGCCATCACAGCTCTCCTACTCCTCCTATCACTACCAGTACTAGCAGGAGCAATCACAATATTATTAACTGACCGAAACCTAAATACAT
CCTTCTTCGATCCAGCAGGAGGTGGAGACCCAATTCTATACCAACACCTATTTTGATTCTTCGGACACCCCGAAGTATAAA---
----

>G3_FT4

GGTTGGAACATCCCTAAGAATATTAATCCGAACAGAACTTGGACAACCAGGATCATTAATTGGGGACGACCAAATCTACAACGTCATCGTCACAGCTCACGCTTTCGTAATAAT
CTTCTTCATAGTAATACCAATCATGATTGGAGGATTCGGAAACTGACTAGTACCGCTAATATTAGGAGCACCAGACATAGCATTCCCTCGAATAAACAACATAAGATTCTGATT
ATTACCACCATCATTAACTCTTCTTCTCACTAGTAGAACAGTAGAAAGTGGTGCAGGAACAGGATGAACAGTATACCCACCCCTTGCAAGAGGAATTGCCCACGCCGGAGCAT

CAGTAGATCTAGCCATCTTCTCATTACACTTAGCAGGAGTATCATCAATCCTAGGAGCAGTAAACTTCATTTCAACAACAATCAACATGAAACCAAAAAACATAAAGCCCGAAC

GAATCCCCTTATTCGTATGATCAGTTGCCATCACAGCTCTCCTACTCCTCCTATCACTACCAGTACTAGCAGGAGCAATCACAATATTATTAACTGACCGAAACCTAAATACATCC

TTCTTCGATCCAGCAGGAGGTGGAGACCCAATTCTATACCAACACCTATTTTGATTCTTCGGACACCCCGAAGTATAA---

----

>G3_FT2

TGATCAGGAATGGTTGGAACATCCCTAAGAATATTAATCCGAACAGAACTTGGACAACCAGGATCATTAATTGGGGACGACCAAATCTACAACGTCATCGTCACAGCTCACGC

TTTCGTAATAATCTTCTTCATAGTAATACCAATCATGATTGGAGGATTCGGAAACTGACTAGTACCGCTAATATTAGGAGCACCAGACATAGCATTCCCTCGAATAAACAACAT

AAGATTCTGATTATTACCACCATCATTAACTCTTCTTCTCACTAGTAGAACAGTAGAAAGTGGTGCAGGAACAGGATGAACAGTATACCCACCCCTTGCAAGAGGAATTGCTCA

CGCCGGAGCATCAGTAGATCTAGCCATCTTCTCATTACACTTAGCAGGAGTATCATCAATCCTGGGAGCAGTAAACTTCATTTCAACAACAATCAACATGAAACCAAAAAACAT

AAAGCCCGAACGAATCCCCTTATTCGTATGATCAGTTGCCATCACAGCTCTCCTACTCCTCCTATCACTACCAGTACTAGCAGGAGCAATCACAATATTATTAACTGACCGAAAC

CTAAATACATCCTTCTTCGATCCAGCAGGAGGTGGAGACCCAATTCTATACCAACACCTATTTTGATTCTTCGGACACCC---

----

>G5_FT8

GGAATGGTTGGAACATCCCTAAGAATACTAATCCGAACAGAACTCGGACAACCAGGATCCTTAATTGGAGATGATCAAATCTACAACGTCATCGTCACAGCTCACGCTTTCGT

AATAATTTTCTTCATAGTAATACCAATTATGATTGGAGGATTCGGAAACTGACTAGTACCACTAATATTAGGAGCGCCAGACATAGCATTCCCACGAATAAACAACATAAGATT

CTGATTACTACCACCATCACTAACTCTTCTTCTCACTAGTAGTACAGTAGAAAGTGGTGCAGGAACAGGATGAACAGTATACCCACCCCTTGCAAGAGGGATTGCTCACGCCGG

GGCATCGGTAGACCTAGCCATCTTCTCATTACACTTAGCAGGAGTATCATCCATCTTAGGAGCAGTAAATTTTATTTCAACAACAATCAACATGAAACCAAAAAACATAAAGCC

CGAACGAATTCCGCTATTCGTATGATCAGTTGCCATCACGGCTCTCCTACTCCTCCTATCACTACCAGTACTAGCAGGAGCAATCACAATGCTATTAACCGACCGAAACCTAAAC

ACATCTTTCTTCGATCCGGCAGGAGGTGGAGACCCAATCCTATATCAACATTTATTTTGATTCTTCGGACACCC---

----

>G5_FT12

TGATCAGGAATGGTTGGAACATCCCTAAGAATACTAATCCGAACAGAACTCGGACAACCAGGATCCTTAATTGGAGATGATCAAATCTACAACGTCATCGTCACAGCTCACGC

TTTCGTAATAATTTTCTTCATAGTAATACCAATTATGATTGGAGGATTCGGAAACTGACTAGTACCACTAATATTAGGAGCGCCAGACATAGCATTCCCACGAATAAACAACAT

AAGATTCTGATTACTACCACCATCACTAACTCTTCTTCTCACTAGTAGTACAGTAGAAAGTGGTGCAGGAACAGGATGAACAGTATACCCACCCCTTGCAAGAGGGATTGCTCA

CGCCGGGGCATCGGTAGACCTAGCCATCTTCTCATTACACTTAGCAGGAGTATCATCCATCTTAGGAGCAGTAAATTTTATTTCAACAACAATCAACATGAAACCAAAAAACAT

AAAGCCCGAACGAATTCCGCTATTCGTATGATCAGTTGCCATCACGGCTCTCCTACTCCTCCTATCACTACCAGTACTAGCAGGAGCAATCACAATGCTATTAACCGACCGAAAC

CTAAACACATCTTTCTTCGATCCGGCAGGAGGTGGAGACCCAATCCTATATCAACATTTATTTTGATTCTTCGGACA--

----

>G5_FT13

TGATCAGGAATGGTTGGAACATCCCTAAGAATACTAATCCGAACAGAACTCGGACAACCAGGATCCTTAATTGGAGATGATCAAATCTACAACGTCATCGTCACAGCTCACGC

TTTCGTAATAATTTTCTTCATAGTAATACCAATTATGATTGGAGGATTCGGAAACTGACTAGTACCACTAATATTAGGAGCGCCAGACATAGCATTCCCACGAATAAACAACAT

AAGATTCTGATTACTACCACCATCACTAACTCTTCTTCTCACTAGTAGTACAGTAGAAAGTGGTGCAGGAACAGGATGAACAGTATACCCACCCCTTGCAAGAGGGATTGCTCA

CGCCGGGGCATCGGTAGACCTAGCCATCTTCTCATTACACTTAGCAGGAGTATCATCCATCTTAGGAGCAGTAAATTTTATTTCAACAACAATCAACATGAAACCAAAAAACAT

AAAGCCCGAACGAATTCCGCTATTCGTATGATCAGTTGCCATCACGGCTCTCCTACTCCTCCTATCACTACCAGTACTAGCAGGAGCAATCACAATGCTATTAACCGACCGAAAC

CTAAACACATCTTTCTTCGATCCGGCAGGAGGTGGAGACCCAATCCTATATCAACATTTATTTTGATTCTTCGGACACC---

----

>G6_FT7

GGAATGGTTGGAACATCCCTAAGAATACTAATTCGAACAGAACTCGGACAACCAGGATCCTTAATTGGGGACGATCAAATCTACAACGTCATCGTCACAGCTCACGCCTTCGT

AATGATCTTCTTTATAGTAATACCAATCATGATTGGAGGATTCGGAAACTGACTAGTACCGCTAATATTAGGAGCGCCAGACATAGCATTCCCACGAATAAACAACATAAGATT

CTGATTACTACCACCATCACTAACTCTTCTTCTCACTAGTAGAACAGTAGAAAGTGGTGCAGGAACAGGATGAACAGTATACCCACCCCTTGCAAGAGGGATTGCTCACGCCG

GAGCATCAGTAGACCTAGCCATCTTCTCACTACACTTAGCAGGAGTATCCTCCATCCTAGGAGCAGTAAATTTCATTTCAACAACAATCAACATGAAACCAAAAAACATAAAGC
CCGAACGAATCCCGCTATTCGTATGATCAGTTGCCATCACGGCCCTCCTACTCCTCCTATCACTACCAGTACTAGCAGGAGCAATCACAATGCTATTAACCGACCGAAACCTAAA
CACATCTTTCTTCGATCCGGCAGGAGGTGGAGACCCAATCCTATATCAACATTTATTTTGATTCTTCGGACACCC---
----

>G6_FT9

GATCAGGAATGGTTGGAACATCCCTAAGAATACTAATTCGAACAGAACTCGGACAACCAGGATCCTTAATTGGGGACGATCAAATCTACAACGTCATCGTCACAGCTCACGCC
TTCGTAATGATCTTCTTTATAGTAATACCAATCATGATTGGAGGATTCGGAAACTGACTAGTACCGCTAATATTAGGAGCGCCAGACATAGCATTCCCACGAATAAACAACATA
AGATTCTGATTACTACCACCATCACTAACTCTTCTTCTCACTAGTAGAACAGTAGAAAGTGGTGCAGGAACAGGATGAACAGTATACCCACCCCTTGCAAGAGGGATTGCTCAC
GCCGGAGCATCAGTAGACCTAGCCATCTTCTCACTACACTTAGCAGGAGTATCCTCCATCCTAGGAGCAGTAAATTTCATTTCAACAACAATCAACATGAAACCAAAAAACATA
AAGCCCGAACGAATCCCGCTATTCGTATGATCAGTTGCCATCACGGCCCTCCTACTCCTCCTATCACTACCAGTACTAGCAGGAGCAATCACAATGCTATTAACCGACCGAAAC
CTAAACACATCTTTCTTCGATCCGGCAGGAGGTGGAGACCCAATCCTATATCAACATTTATTTTGATTCTTCGGACACCC---
----

>G6_FT14

GATCAGGAATGGTTGGAACATCCCTAAGAATACTAATTCGAACAGAACTCGGACAACCAGGATCCTTAATTGGGGACGATCAAATCTACAACGTCATCGTCACAGCTCACGCC

TTCGTAATGATCTTCTTTATAGTAATACCAATCATGATTGGAGGATTCGGAAACTGACTAGTACCGCTAATATTAGGAGCGCCAGACATAGCATTCCCACGAATAAACAACATA

AGATTCTGATTACTACCACCATCACTAACTCTTCTTCTCACTAGTAGAACAGTAGAAAGTGGTGCAGGAACAGGATGAACAGTATACCCACCCCTTGCAAGAGGGATTGCTCAC

GCCGGAGCATCAGTAGACCTAGCCATCTTCTCACTACACTTAGCAGGAGTATCCTCCATCCTAGGAGCAGTAAATTTCATTTCAACAACAATCAACATGAAACCAAAAAACATA

AAGCCCGAACGAATCCCGCTATTCGTATGATCAGTTGCCATCACGGCCCTCCTACTCCTCCTATCACTACCAGTACTAGCAGGAGCAATCACAATGCTATTAACCGACCGAAAC

CTAAACACATCTTTCTTCGATCCGGCAGGAGGTGGAGACCCAATCCTATATCAACATTTATTTTGATTCTTCGGACACCC--

----

>G6_FT15

GATCAGGAATGGTTGGAACATCCCTAAGAATACTAATTCGAACAGAACTCGGACAACCAGGATCCTTAATTGGGGACGATCAAATCTACAACGTCATCGTCACAGCTCACGCC

TTCGTAATGATCTTCTTTATAGTAATACCAATCATGATTGGAGGATTCGGAAACTGACTAGTACCGCTAATATTAGGAGCGCCAGACATAGCATTCCCACGAATAAACAACATA

AGATTCTGATTACTACCACCATCACTAACTCTTCTTCTCACTAGTAGAACAGTAGAAAGTGGTGCAGGAACAGGATGAACAGTATACCCACCCCTTGCAAGAGGGATTGCTCAC

GCCGGAGCATCAGTAGACCTAGCCATCTTCTCACTACACTTAGCAGGAGTATCCTCCATCCTAGGAGCAGTAAATTTCATTTCAACAACAATCAACATGAAACCAAAAAACATA

AAGCCCGAACGAATCCCGCTATTCGTATGATCAGTTGCCATCACGGCCCTCCTACTCCTCCTATCACTACCAGTACTAGCAGGAGCAATCACAATGCTATTAACCGACCGAAAC

CTAAACACATCTTTCTTCGATCCGGCAGGAGGTGGAGACCCAATCCTATATCAACATTTATTTTGATTCTTCGGACACCC--

----

>G6_FT11

GATCAGGAATGGTTGGAACATCCCTAAGAATACTAATTCGAACAGAACTCGGACAACCAGGATCCTTAATTGGGGACGATCAAATCTACAACGTCATCGTCACAGCTCACGCC

TTCGTAATGATCTTCTTTATAGTAATACCAATCATGATTGGAGGATTCGGAAACTGACTAGTACCGCTAATATTAGGAGCGCCAGACATAGCATTCCCACGAATAAACAACATA

AGATTCTGATTACTACCACCATCACTAACTCTTCTTCTCACTAGTAGAACAGTAGAAAGTGGTGCAGGAACAGGATGAACAGTATACCCACCCCTTGCAAGAGGGATTGCTCAC

GCCGGAGCATCAGTAGACCTAGCCATCTTCTCACTACACTTAGCAGGAGTATCCTCCATCCTAGGAGCAGTAAATTTCATTTCAACAACAATCAACATGAAACCAAAAAACATA

AAGCCCGAACGAATCCCGCTATTCGTATGATCAGTTGCCATCACGGCCCTCCTACTCCTCCTATCACTACCAGTACTAGCAGGAGCAATCACAATGCTATTAACCGACCGAAAC

CTAAACACATCTTTCTTCGATCCGGCAGGAGGTGGAGACCCAATCCTATATCAACATTTATTTTGATTCTTCGGACACCCCGAAGTATAA--

----

>G6_FT10

GAATGGTTGGAACATCCCTAAGAATACTAATTCGAACAGAACTCGGACAACCAGGATCCTTAATTGGGGACGATCAAATCTACAACGTCATCGTCACAGCTCACGCCTTCGTA

ATGATCTTCTTTATAGTAATACCAATCATGATTGGAGGATTCGGAAACTGACTAGTACCGCTAATATTAGGAGCGCCAGACATAGCATTCCCACGAATAAACAACATAAGATTC

TGATTACTACCACCATCACTAACTCTTCTTCTCACTAGTAGAACAGTAGAAAGTGGTGCAGGAACAGGATGAACAGTATACCCACCCCTTGCAAGAGGGATTGCTCACGCCGG

AGCATCAGTAGACCTAGCCATCTTCTCACTACACTTAGCAGGAGTATCCTCCATCCTAGGAGCAGTAAATTTCATTTCAACAACAATCAACATGAAACCAAAAAACATAAAGCC
CGAACGAATCCCGCTATTCGTATGATCAGTTGCCATCACGGCCCTCCTACTCCTCCTATCACTACCAGTACTAGCAGGAGCAATCACAATGCTATTAACCGACCGAAACCTAAAC
ACATCTTTCTTCGATCCGGCAGGAGGTGGAGACCCAATCCTATATCAACATTTATTTTGATTCTTCGGACACCCCGAAGTATA---
----

>G12_Macrotermes_vitrialatus_mtgenome_-_COX1 Macrotermes vitrialatus mitochondrion, complete genome

ATCTTCACCCGATGATTCTTCTCAACTAATCACAAAGACATTGGAACACTATATTTTGTATTCGGAGCCTGATCAGGAATGGTCGGAACATCTCTTAGAATGCTAATCCGAACAG
AACTTGGACAACCAGGATCCCTAATTGGTGATGATCAAATCTATAATGTCATCGTCACCGCTCACGCCTTTGTAATAATTTTCTTTATAGTAATACCAATTATGATCGGGGGATT
CGGAAACTGACTAGTACCACTTATATTAGGAGCACCAGACATAGCATTCCCACGAATAAACAACATAAGATTTTGACTATTACCACCATCATTAACTCTTCTTCTCACTAGTAGA
ACGGTAGAAAGTGGTGCAGGAACAGGATGGACAGTATATCCCCCTCTTGCGAGAGGCATTGCCCATGCCGGAGCATCCGTAGACCTAGCTATCTTCTCCCTACACCTAGCAGG
TGTATCCTCCATCCTTGGAGCAGTAAACTTCATTTCAACAACAATCAACATGAAACCAAAAAGTATAAAACCTGAACGAATCCCACTATTCGTATGATCAGTTGCCATCACAGCC
CTCTTGCTACTTCTATCATTACCCGTACTAGCAGGAGCAATCACAATACTATTAACTGATCGAAACCTTAATACATCCTTCTTTGACCCAGCAGGAGGGGGAGACCCAATCCTAT
ATCAACACCTATTCTGATTCTTCGGACACCCAGAAGTGTATATCCTAATTCTACCAGGATTTGGTATAATCTCACACATCATTTGCCACGAAAGAGGTAAAAAGGAAGCCTTCG
GAAACTTGGGAATAATCTTTGCTATACTAGCAATTGGGTTACTAGGATTCGTAGTATGGGCACATCATATGTTCACAGTAGGAATAGATGTTGATACACGAGCCTACTTTACAT
CAGCAACAATAATTATTGCAGTACCAACCGGAATCAAAATCTTCAGATGACTCGCAACCATATATGGAACCCGAATAACCTATAGAGCAGCATGCTTGTGAGCTCTAGGATTTG
TATTTCTATTCACAATAGGAGGCCTCACAGGAGTAGTGTTAGCAAACTCATCAATCGACATTGTACTACACGACACATACTACGTAGTAGCCCACTTCCACTACGTACTATCAAT
AGGAGCAGTATTCGCAATCATAGGGGGATTCGTTCAATGATTCCCATTATTTACAGGACTAACTATAAACCCAAAATGACTAAAAGCCCAATTCGCCGTAATATTTACGGGAGT
AAATTTAACATTCTTCCCACAACACTTCCTTGGACTAGCAGGAATACCACGACGATATTCTGATTATCCAGACGCCTACACCACATGAAATATCATCTCATCAATGGGATCAACA

ATCTCATTTGTAAGAGTAATAATATTCATATTCATCATATGAGAAAGAATTGTATCAAACCGCCAAATCCTATTCCCGACACACACAAGAAATTCAGTAGAATGACTACAAAACT
TCCCACCAGCAGAGCACAGATATTCAGAACTACCAACCATCTCAATAACTAACTAA

>G13_Macrotermes_lilljeborgi Macrotermes lilljeborgi isolate dka143 cytocrome oxidase subunit I (COI) gene, partial cds; mitochondrial

----
ATATTAATCCGAACAGAACTTGGACAACCGGGATCCCTAATTGGAGATGACCAAATCTACAACGTCATCGTCACCGCTCATGCCTTTGTAATAATTTTCTTCATAGTAATACCAA
TCATGATTGGGGGATTCGGAAACTGACTAGTACCACTCATATTAGGAGCACCAGACATAGCTTTCCCACGAATAAACAACATAAGATTCTGATTACTACCACCATCACTGACCC
TTCTTCTCACTAGTAGAACAGTAGAAAGCGGTGCAGGAACAGGATGGACAGTATACCCCCCTCTCGCGAGAGGGATTGCCCATGCCGGAGCATCCGTAGATCTAGCCATCTTC
TCCCTACACCTAGCAGGTGTATCCTCCATCCTAGGAGCAGTAAATTTTATTTCAACAACAATCAACATGAAACCAAAAAGCATAAAACCTGAACGAATCCCTCTATTCGTATGAT
CAGTTGCCATCACAGCCCTACTACTGCTATTATCGCTACCCGTACTAGCAGGAGCAATCACAATACTACTAACTGACCGAAACCTCAATACATCCTTCTTCGACCCAGCAGGAG
GGGGAGACCCAATCCTATATCAACACTTATTTTGATTCTTCGGACACCCTGAAGTATATATCCTAATTCTACCTGGATTCGGTATAATCTCACACATCATTTGTCACGAAAGAGG
CAAAAAGGAAGCCTTCGGAAACCTAGGAATAATTTTTGCTATACTAGCAATCGGACTACTAGGTTTCGTAGTATGAGCACATCACATATTTACAGTAGGAATAGATGTTGACAC
ACGAGCCTATTTTACATCAGCAACAATAATCATCGCAGTACCAACAGGAATCAAAATCTTCAGATGACTCGCAACAATATACGGAACTCGAATAACCTACAGAGCAGCATGCCT
ATGAGCCCTAGGATTCGTATTTC--

>G15_Macrotermes_muelleri Macrotermes muelleri isolate 367551 cytocrome oxidase subunit I (COI) gene, partial cds; mitochondrial

----
ATATTAATCCGAACAGAACTCGGACAACCAGGATCCCTAATTGGAGACGACCAAATCTACAACGTCATCGTCACCGCCCATGCCTTTGTAATAATTTTCTTCATAGTAATACCAA
TCATGATTGGAGGATTCGGAAACTGACTAGTACCACTCATATTAGGAGCACCAGACATAGCATTCCCACGAATAAACAACATAAGATTCTGATTGCTACCACCATCACTGACTC

TTCTTCTCACTAGTAGAACAGTAGAAAGCGGTGCAGGAACAGGATGGACAGTATACCCACCTCTTGCGAGAGGTATTGCCCACGCCGGAGCATCCGTAGATCTAGCTATCTTC
TCCCTACACCTAGCAGGTGTATCCTCCATCCTAGGAGCAGTAAATTTTATTTCAACAACAATCAACATGAAACCAAAAAGCATAAAACCTGAACGAATCCCCCTATTCGTATGAT
CAGTTGCCATCACAGCCCTACTACTGCTCCTATCACTGCCCGTACTAGCAGGGGCAATCACAATACTACTAACTGACCGAAACCTCAATACATCCTTCTTCGACCCAGCAGGAG
GTGGAGACCCAATTCTATATCAACACTTATTTTGATTCTTCGGGCACCCTGAAGTGTATATCCTAATTCTACCCGGATTTGGTATAATCTCACACATCATTTGCCACGAAAGAGG
TAAAAAGGAAGCCTTCGGAAACCTAGGAATAATTTTTGCTATACTAGCAATCGGACTGCTAGGTTTCGTAGTATGAGCACATCACATATTTACAGTAGGAATAGATGTTGATAC
ACGAGCCTACTTTACATCAGCAACAATAATTATCGCAGTACCAACAGGAATTAAAATCTTCAGATGACTCGCAACAATATACGGAACTCGAATAACATACAGAGCGGCATGCCT
ATGGGCCCTAGGATTCGTATTTC---

>G15_Macrotermes_muelleri_mtgenome_-_COX1_ Macrotermes muelleri mitochondrion, complete genome

----
TCACTCGATGGTTCTTCTCAACTAATCACAAAGACATTGGAACACTATACTTTGTATTCGGAGCCTGATCAGGAATGGTTGGAACATCTCTCAGAATATTAATCCGAACAGAACT
CGGACAACCAGGATCCCTAATTGGAGACGACCAAATCTACAACGTCATCGTCACCGCCCATGCCTTTGTAATAATTTTCTTCATAGTAATACCAATCATGATTGGAGGATTCGG
AAACTGGCTAGTACCACTCATATTAGGAGCACCAGACATAGCATTCCCACGAATAAACAACATAAGATTCTGATTGCTACCACCATCACTGACTCTTCTTCTCACTAGTAGAACA
GTAGAAAGCGGTGCAGGAACAGGATGGACAGTATACCCCCCTCTCGCAAGAGGTATTGCTCACGCCGGAGCATCCGTAGATCTAGCCATCTTCTCCCTACACCTAGCAGGTGT
ATCCTCCATCCTAGGAGCAGTAAATTTTATTTCAACAACAATCAACATGAAACCAAAAAGCATAAAACCTGAACGAATCCCCCTATTCGTATGATCAGTTGCCATCACAGCCCTA
CTACTGCTCCTATCACTGCCCGTACTAGCAGGAGCAATCACAATACTACTAACTGACCGAAACCTCAATACATCCTTCTTCGACCCAGCAGGAGGTGGAGACCCAATTCTATATC
AACACTTATTTTGATTCTTCGGGCACCCTGAAGTGTATATCCTAATTCTACCCGGATTTGGTATAATCTCACACATCATTTGCCACGAAAGAGGTAAAAAGGAGGCCTTCGGAA
ACCTAGGAATAATTTTTGCCATACTAGCAATCGGACTGCTAGGTTTCGTAGTATGAGCACATCACATATTTACAGTAGGAATAGATGTTGATACACGAGCCTACTTTACATCAG
CAACAATAATTATCGCAGTACCAACAGGAATTAAAATCTTCAGATGACTCGCAACAATATACGGAACTCGAATAACCTACAGAGCGGCATGCCTATGGGCCCTAGGATTCGTA
TTTCTATTCACAATAGGTGGCCTCACAGGGGTGGTACTAGCAAATTCATCAATCGAC---

>G14_Macrotermes_nobilis Macrotermes nobilis isolate 367569 cytocrome oxidase subunit I (COI) gene, partial cds; mitochondrial

----

ATACTAATCCGAACAGAACTTGGACAGCCAGGGTCCCTAATTGGAGACGACCAAATCTACAACGTCATCGTCACCGCCCATGCCTTTGTAATAATTTTCTTCATAGTAATACCAA
TCATGATTGGGGGATTCGGAAACTGGCTAGTACCACTCATATTAGGAGCACCAGACATAGCATTCCCACGAATAAACAACATAAGATTCTGATTACTACCACCATCACTGACTC
TTCTTCTCACTAGTAGAACAGTAGAAAGCGGTGCAGGAACAGGGTGGACAGTATACCCCCCTCTCGCGAGAGGAATTGCCCATGCCGGAGCATCCGTAGACCTAGCCATCTTC
TCACTACACCTAGCAGGTGTATCCTCCATCCTAGGGGCAGTAAACTTTATTTCAACAACAATCAACATGAAACCAAAAAGCATAAAACCCGAACGAATCCCCCTATTCGTATGA
TCAGTTGCCATCACAGCCCTACTGCTGCTTCTATCACTACCCGTACTAGCAGGAGCAATCACAATACTACTAACTGATCGAAACCTCAATACATCCTTCTTCGACCCAGCAGGAG
GTGGAGACCCAATCCTATATCAACACTTATTTTGATTCTTTGGACACCCTGAAGTATATATCCTAATTCTACCTGGATTTGGTATAATTTCACACATCATTTGCCACGAAAGAGGT
AAAAAGGAAGCCTTCGGAAACCTAGGAATAATTTTTGCTATACTAGCAATCGGACTGCTGGGATTCGTAGTATGAGCACATCACATATTTACAGTAGGAATAGATGTTGATAC
ACGAGCCTACTTTACATCAGCAACAATAATTATCGCAGTGCCAACAGGAATCAAAATCTTCAGATGACTCGCAACAATATACGGAACTCGAATAACCTACAGAGCAGCATGCCT
ATGAGCTCTAGGATTTGTATTCC--

>G16_Macrotermes_bellicosus_3 Macrotermes bellicosus isolate Mb cytochrome oxidase subunit I (COI) gene, partial cds; mitochondrial

----

TTGGAGACGATCAAATCTAYAATGTCATCGTCAGAGCCCACGCTTTCGTCATGATYTTCTTCATGGTAATACCAATCATGATCGGAGGATTGGGAAACTGATTAGTACCACTTAT
ATTAGGAGCACCAGACATAGCATTTCCACGAATAAATAACATAAGATTCTGATTATTACCACCATCACTAACCCTTCTTCTCACTAGCAGGACGGTAGAAAGCGGTGCAGGAAC

AGGATGAACAGTGTACCCACCCCTAGCAAGAGGAATTGCACACGCTGGAGCATCCGTAGATCTAGCCATCTTCTCCCTACACCTAGCGGGTGTATCATCAATTTTAGGAGCAG

TAAACTTTATCTCAACAATAATCAACATAAAACCGAGAAGTATAAAACCTGAACGAATCCCCCTATTCGTATGATCAGTTGCCATCACAGCCCTTCTACTACTATTGTCACTACYG

GTACTAGCAGGAGCAATCACAATACTATTAACCGACYGAAACTTAAACACATCCTTCTTTGACCCGGCAGGAGGAGGGGATCCAATCCTATATCAACACCTATTTTGATTCTTC

GGACACCCCGTAGTATATATCTTAATTCTACCAGGATTTGGTATAATCTCTCATATTATCTGCCACGAAAGAGGTAAAAAGGAAGCCTTCGGAAAACTGGGAATAATCTTTGCT

ATACTAGCAATTGGATTACTAGGATTTGTAGTATGAGCACACCACATATTCACAGTAGGAATAGATGTTGATACACGAGCCTACTTCACATCAGCAACAATAATTATTGCAGTG

CCAACAGGAATTAAAATCTTCAGATGACTTGCAACCATATACGGAACCAGAATAACCTATCKAGCAGCATGCCTGTGAGCCCTAGGATTTGTATT---------------------------------

----

>G17_Macrotermes_bellicosus_1 Macrotermes bellicosus isolate dka19 cytocrome oxidase subunit I (COI) gene, partial cds; mitochondrial

----

ATATTAATTCGAACAGAACTAGGACAACCAGGATCCTTAATTGGAGACGATCAAATCTACAACGTCATCGTCACAGCTCACGCCTTCGTCATGATCTTCTTCATGGTAATACCAA

TTATGATCGGAGGGTTCGGAAACTGAYTAGTACCACTCATATTAGGAGCACCAGATATAGCATTCCCACGAATAAATAACATAAGATTCTGATTATTACCACCATCACTAACCC

TTCTTCTCACTAGCAGAACGGTAGAAAGTGGTGCAGGAACAGGATGAACGGTATACCCACCCCTAGCAAGAGGTATTGCACATGCTGGGGCATCTGTAGATCTTGCCATCTTT

TCCCTACACCTAGCGGGAGTATCATCTATCCTAGGAGCAGTAAACTTTATCTCAACAACTATCAACATAAAGCCAAGAAACATAAAACCAGAGCGAATTCCCCTATTCGTATGA

TCAGTAGCCATTACAGCCCTTCTCCTGTTATTATCACTACCAGTACTAGCAGGAGCAATCACAATACTACTAACTGATCGAAACCTGAACACATCCTTCTTTGACCCAGCAGGAG

GGGGAGACCCAATCCTATATCAACACCTATTCTGATTCTTCGGGCACCCCGAAGTATACATCTTAATTCTACCAGGATTTGGTATAATCTCCCACATTATCTGCCATGAAAGAGG

TAAAAAGGAAGCCTTCGGAAACCTGGGAATAATCTTTGCTATACTAGCAATTGGATTACTAGGATTTGTAGTATGAGCACACCACATATTCACAGTAGGGATAGATGTTGACA

CACGAGCCTACTTCACATCAGCAACAATAATTATTGCAGTACCAACAGGGATTAAAATCTTCAGATGACTTGCAACCATGTACGGAACCCAAATAACCTATAGAGCAGCATGCC

TTTGAGCCTTAGGGTTTGTATTCT--

>G17_Macrotermes_bellicosus_2 Macrotermes bellicosus isolate dka8 cytocrome oxidase subunit I (COI) gene, partial cds; mitochondrial

----

ATATTAATTCGAACAGAACTAGGACAACCAGGATCCTTAATTGGAGACGATCAAATCTACAACGTCATCGTCACAGCTCACGCCTTCGTCATGATCTTCTTCATGGTAATACCAA

TTATGATCGGAGGGTTCGGAAACTGATTAGTACCACTCATATTAGGAGCACCAGATATAGCATTCCCACGAATAAATAACATAAGATTCTGATTATTACCACCATCACTAACCC

TTCTTCTCACTAGCAGAACGGTAGAAAGTGGTGCAGGAACAGGATGAACGGTATACCCACCCCTAGCAAGAGGTATTGCACATGCTGGGGCATCTGTAGATCTTGCCATCTTT

TCCCTACACCTAGCGGGAGTATCATCTATCCTAGGAGCAGTAAACTTTATCTCAACAACTATCAACATAAAGCCAAGAAACATAAAACCAGAGCGAATTCCCCTATTCGTATGA

TCAGTAGCCATTACAGCCCTTCTCCTGTTATTATCACTACCAGTACTAGCAGGAGCAATCACAATACTACTAACTGATCGAAACCTGAACACATCCTTCTTTGACCCAGCAGGAG

GGGGAGACCCAATCCTATATCAACACCTATTCTGATTCTTCGGGCACCCCGAAGTATACATCTTAATTCTACCAGGATTTGGTATAATCTCCCACATTATCTGCCATGAAAGAGG

TAAAAAGGAAGCCTTCGGAAACCTGGGAATAATCTTTGCTATACTAGCAATTGGATTACTAGGATTTGTAGTATGAGCACACCACATATTCACAGTAGGGATAGATGTTGACA

CACGAGCCTACTTCACATCAGCAACAATAATTATTGCAGTACCAACAGGGATTAAAATCTTCAGATGACTTGCAACCATGTACGGAACCCGAATAACCTATAGAGCAGCATGCC

TTTGAGCCTTAGGGTTTGTATTCT--
